# Comparing the Performances of Force Fields in Conformational Searching of Hydrogen-Bond-Donating Catalysts

**DOI:** 10.1021/acs.joc.2c00066

**Published:** 2022-04-27

**Authors:** Toby Lewis-Atwell, Piers A. Townsend, Matthew N. Grayson

**Affiliations:** † Department of Computer Science, University of Bath, Claverton Down, Bath BA2 7AY, U.K.; ‡ Centre for Sustainable Chemical Technologies, University of Bath, Claverton Down, Bath BA2 7AY, U.K.; § Department of Chemistry, University of Bath, Claverton Down, Bath BA2 7AY, U.K.

## Abstract

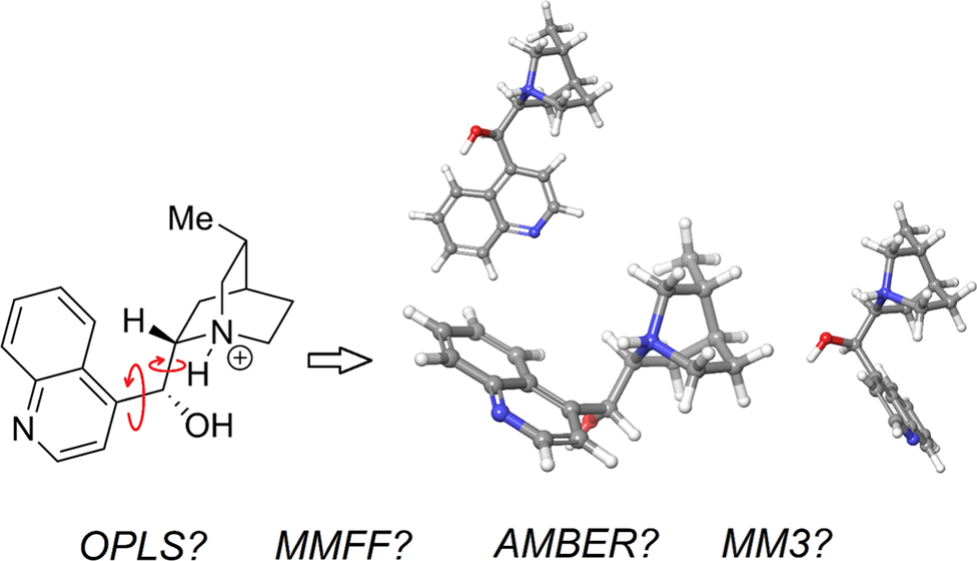

Here, we compare the relative performances of different force fields for conformational searching of hydrogen-bond-donating catalyst-like molecules. We assess the force fields by their predictions of conformer energies, geometries, low-energy, nonredundant conformers, and the maximum numbers of possible conformers. Overall, MM3, MMFFs, and OPLS3e had consistently strong performances and are recommended for conformationally searching molecules structurally similar to those in this study.

## 1 Introduction

Conformational searching involves changing a molecule’s geometry and performing energy minimizations in an attempt to find all of the stable conformations of the system, and it is very often a critical stage in a reaction modeling investigation.^1−6^ Due to their very low computational cost, force fields are particularly suited to conformational searching. Since often thousands of energy minimizations are required in a conformational search, a more accurate quantum mechanical method will likely be far too expensive for this application. Therefore, given the diversity of force fields and their parameterizations, a useful endeavor is to assess the performances of different force fields in conformational searching and to determine which is most reliable and best suited to a given task. In a recent review,^7^ we analyzed the literature where different force fields were compared by their performances in conformational analysis and conformational searching. For conformational analysis (i.e., studying the ability of force fields to reproduce the energies and/or geometries of specific molecule conformations), “MM” force fields, specifically MM2, MM3, and MMFF94, were consistently found to obtain good performances in this area. However, there were relatively few studies in which force fields were compared by their abilities to reproduce energies and/or geometries from conformational searching, relative to either experimental crystal structures^8−12^ or quantum mechanical calculations.^13,14^ Thus, the best force field for conformational searching could not be identified. An insufficient number of different systems have been explored with different force fields, and uncertainty exists as to whether conformational searches are agnostic to the choice of force field.^8^ Thus, it is vital that more force field performance comparisons are made to resolve this issue. Therefore, this work provides a comparison of how well different force fields predict the energies and geometries of organic molecule conformers (primarily hydrogen-bond-donating catalysts), relative to a higher level of theory, to inform reaction modeling.

**Figure 1:**
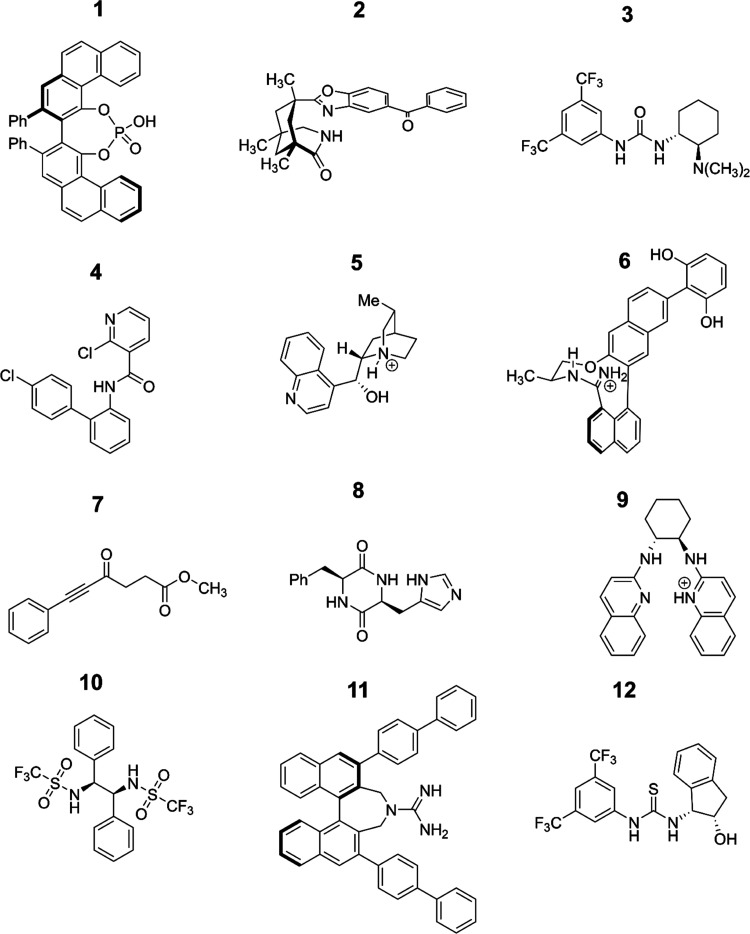
Two-dimensional (2D) structures of 12 of the 20 molecules considered in this study (see also Figure 2).

**Figure 2:**
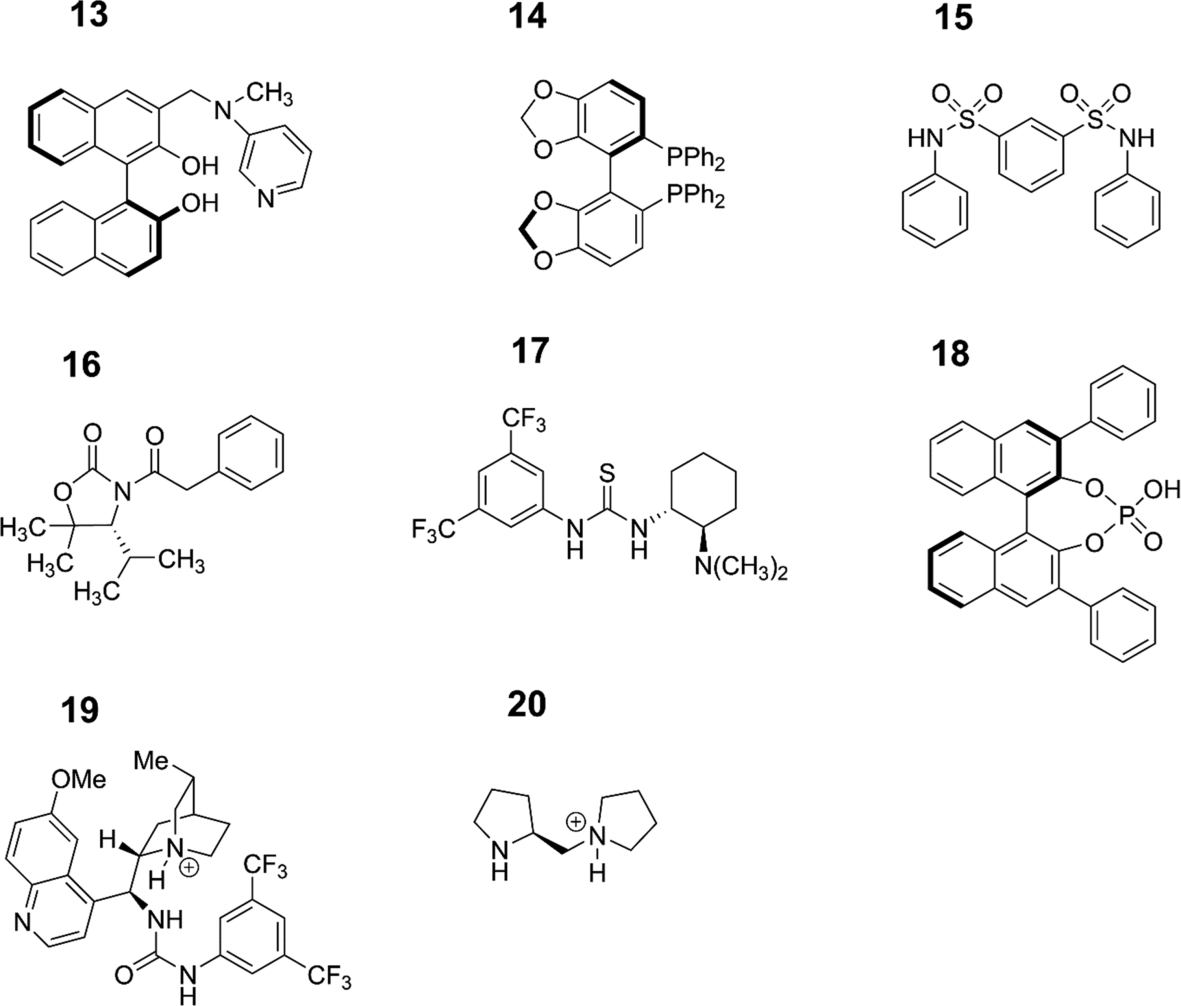
2D structures of the other eight molecules also considered in this study (see also Figure 1).

The general target class of molecules for this investigation was hydrogen-bond-donating catalysts, as well as a few other organic molecules with similar structures. Figures 1 and 2 show the structures of these molecules and note the various structural motifs that may pose a challenge to force fields due to the significant amounts of electronic effects that these structures produce. In this work, we take particular focus on the presence of intramolecular hydrogen bonding and conjugation. The fundamental reasons for these effects are electronic in nature, and therefore, since force fields have no consideration of electrons, they will fail to make accurate predictions of the energies and geometries of systems such as these if their parameterization does not account for these effects properly. In combination, the molecules represent various degrees of conformational flexibility, with the minimum and maximum number of rotatable bonds in the data set being 2 and 11, respectively.

It is perhaps worth briefly mentioning the histories of the classes of force fields used in this study, including the types of system for which they were parameterized. Both the AMBER and OPLS families of force fields were parameterized for proteins,^15−18^ originally using experimental data and small numbers of *ab initio* calculations from small molecules related to proteins such as peptides. However, more recent reparameterizations of the OPLS force field such as OPLS3^19^ and OPLS3e^20^ have extended the scope of these force fields to include a greater array of organic molecules, taking advantage not only of more experimental data but also the greater amount of quantum mechanical computation that may be performed using modern computing hardware. Allinger’s force fields MM2^21^ and MM3^22^ were originally designed for hydrocarbon compounds only and used data from experiment, and experiment and quantum mechanical calculations, respectively. On the other hand, the MMFF94 force field^23^ was intended to be equally useful for both organic molecules and proteins and primarily used data from quantum mechanical calculations. MMFF94s denote a small change in the functional form of MMFF94, in which the energy minimization at unconstrained delocalized trigonal nitrogen atoms causes their geometry to become planar to simulate the experimentally observed “time-averaged” structures.^24^

## 2 Computational Details

In this work, our data set consisted of 20 molecules, most of which were hydrogen-bond-donating catalysts sourced from ref (25), and their two-dimensional structures may be viewed in Figures 1 and 2. For each of these molecules, conformational searches were performed with nine force fields in Schrödinger’s MacroModel v12.6.^26^ The chosen force fields were OPLS3e, OPLS-2005, MMFF, MMFFs, AMBER94, AMBER*, OPLS, MM2*, and MM3*, and they are based on the force fields OPLS3e,^20^ OPLS-AA,^15^ MMFF94,^23^ MMFF94s,^24^ a 1994 parameterization of AMBER,^16^ AMBER,^17^ OPLS,^18^ MM2,^21^ and MM3,^22^ respectively.

Overall, the force fields found a total of 5450 conformers across our chosen molecules, and all structures were optimized to minima with density functional theory (DFT). Please refer to the Supporting Information for full computational methods. The force fields were then compared by their ability to predict single-point DFT energies and geometries of the conformers and by how reliably the force fields find low-energy and nonredundant conformers. To quantify the strength of each force field in predicting the energetic ordering of the conformers, the Spearman coefficient^27^ was calculated between the force field energies and the single-point DFT conformer energies. We calculated the square of the Pearson correlation coefficient (*R*^2^) and the mean absolute deviations (MADs) between the force field and DFT relative energies to evaluate how closely the force field predicts the conformer energies relative to DFT. To determine the quality of the force field geometries, the root-mean-square deviations (RMSDs) between the force field and DFT-optimized structures were calculated. We also test if the force fields can correctly identify the lowest-energy conformers and, if the molecule has any conformers 10 kJ mol^–1^ above the lowest, how many of the conformers within 10 kJ mol^–1^ of the minimum according to the force field were also within 10 kJ mol^–1^ of the minimum DFT energy conformer. Finally, we performed redundant conformer eliminations based only on geometry, with a heavy-atom RMSD cutoff set to 0.1 Å on all of the post-DFT conformers from each force field for each molecule. This was performed to determine what proportion of conformers found by the force fields had converged to the same geometry following DFT optimization.

## 3 Results and Discussion

### 3.1 General Performances of the Force Fields

After conformational searches, it became apparent that some of the force fields lacked the parameterization required for certain functional groups included in our data set, resulting in failed conformational searches. In particular, AMBER94 was only successful for a single molecule (structure 8 in Figure 1), and therefore, DFT optimizations were not performed for this force field. Searches with OPLS3e, OPLS-2005, MMFF, and MMFFs were successful for all 20 molecules. Table 1 summarizes the number of molecules each force field was able to search. Thus, OPLS3e, OPLS-2005, and MMFF/MMFFs are more reliable for a wider range of the molecules in this study.

**Table 1:** Number of Molecules Each Force Field Was Able to Perform Conformational Searches on, out of a Total of 20 Molecules

force field	number of successful molecules (/20)
OPLS3e	20
OPLS-2005	20
MMFF	20
MMFFs	20
AMBER94	1
AMBER*	17
OPLS	13
MM2*	18
MM3*	12

**Figure 3:**
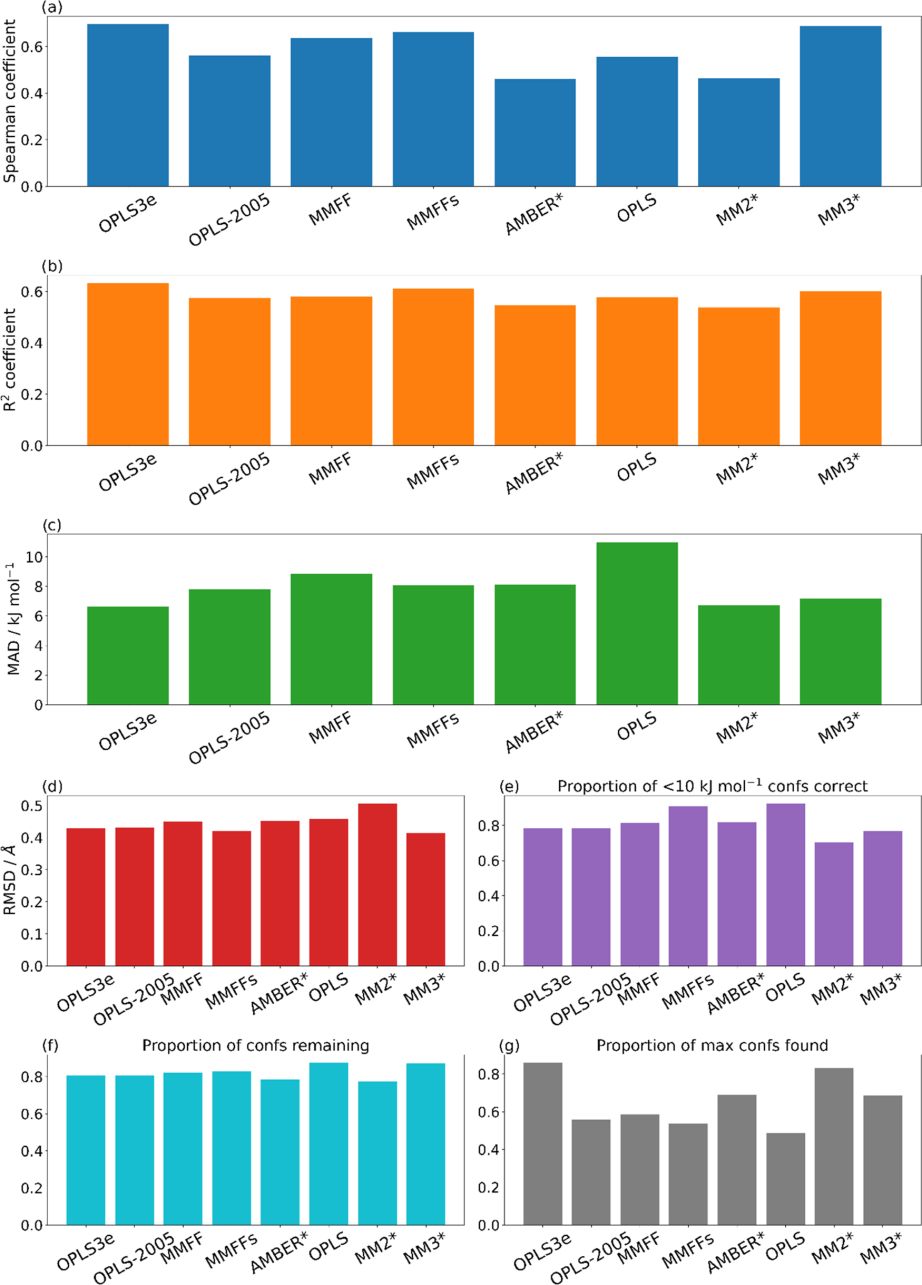
(a) Mean values of the Spearman coefficients between the force field and DFT energies for all of the molecules for which each force field was able to perform conformational searches. (b) The mean values of the *R*^2^ coefficients between the force field and DFT energies for all of the molecules for which each force field was able to perform conformational searches. (c) The mean values of the MADs between the force field and DFT relative energies for all of the molecules for which each force field was able to perform conformational searches. (d) The mean values of the heavy-atom RMSDs between the force field and DFT structures for all of the molecules for which each force field was able to perform conformational searches. (e) The mean values of the proportions of conformers that were correctly predicted by the force field to be within 10 kJ mol^–1^ of the minimum energy conformer according to DFT. (f) The mean values of the ratios between the number of conformers following redundant conformer elimination and the number of conformers found by the force fields. (g) The mean values of the ratios between the final number of conformers for each molecule from each force field and the maximum number of conformers found by any force field for that molecule. The individual plots shown here may also be found in the SI.

Figure 3a–c plots the mean values of the Spearman coefficients, *R*^2^ coefficients, and MADs between the force field and the DFT relative energies from all of the conformers of all of the molecules for which each force field was able to perform conformational searches. The mean values of the Spearman coefficients indicate that OPLS3e, MM3*, and MMFFs (in order of performance) are the best force fields for predicting the energetic ordering of the conformers. A Spearman coefficient closer to unity indicates that the ordering of the force field conformer energies is closer to the ordering observed using DFT; thus, a force field that is associated with a greater Spearman coefficient is more likely to be able to provide a more reliable energetic ordering of the conformers it finds. Similarly, the mean values of the *R*^2^ coefficients for OPLS3e, MMFFs, and MM3* are also slightly higher than for the other force fields, indicating that the force field conformer energies tend to be more strongly correlated with the values from DFT. The MAD mean values between the force field and DFT conformer relative energies show that OPLS3e, MM2*, and MM3* predict the conformer relative energies closer to DFT than the other force fields. Figure 3d shows that the lowest mean heavy-atom RMSDs between the force field and DFT-optimized structures were from MM3*, MMFFs, and OPLS3e, indicating that these force fields have a slight overall advantage in predicting the molecular geometry of the conformers after DFT optimization.

Note the plots in Figure 3, for each force field, give the mean values of the metrics calculated for each molecule in the data set. However, throughout this section, we will direct the reader to the SI, wherein one may find similar plots to Figure 3, but with the values of the metrics for each molecule in the data set plotted individually, to allow more detailed analysis of how individual molecules may influence the average values of the metric presented in Figure 3.

Figures S2, S4, and S6 in the SI show each force field’s Spearman coefficients, *R*^2^ coefficients, and MADs between force field and DFT conformer energies for each molecule in the data set individually. From these plots, it is apparent that many of the force fields perform particularly poorly for molecules 9–11 (note the generally low bars around the centers of the Spearman and *R*^2^ bar groups in Figures S2 and S4 and the generally high bars near the centers of the MAD bar groups in Figure S6). These three molecules together possess a rather unusual set of structures compared with more “normal” organic molecules that the force fields were traditionally parameterized for, such as peptides and hydrocarbons. Molecules 9 and 11 have rather uncommon arrangements of aromatic rings, and molecule 10 contains two triflyl groups that are very likely to have strong influence over the electronics of the system, which may explain the poor energetic predictions of nearly all of the force fields for these three molecules. The four “MM” force fields (i.e., MMFF, MMFFs, MM2*, and MM3*) have low Spearman and *R*^2^ coefficients and high MADs for molecule 4, whereas the OPLS force fields do not tend to have comparatively poor performance for this molecule. The major difference between this structure and the others in this data set appears to be the presence of the chlorine atoms on the phenyl and pyridine aromatic rings. Perhaps, these atoms in this configuration have found a gap in the parameterization of these force fields, which causes the accuracy of their energetic predictions to degrade. The presence of a charged group (in molecules 5, 6, 9, 19, and 20) does seem to degrade the energetic performance in some molecule-force field combinations, but this is not consistent across the range of force fields tested. For instance, the Spearman and *R*^2^ coefficients and MAD for molecule 19 are rather poor for the force fields MMFF, MMFFs, AMBER*, and OPLS, but these force fields perform much better with molecule 20. Thus, despite being an electrostatic effect, a charged group alone does not seem to be a reliable indicator of whether a force field is likely to perform well or not.

**Figure 4:**
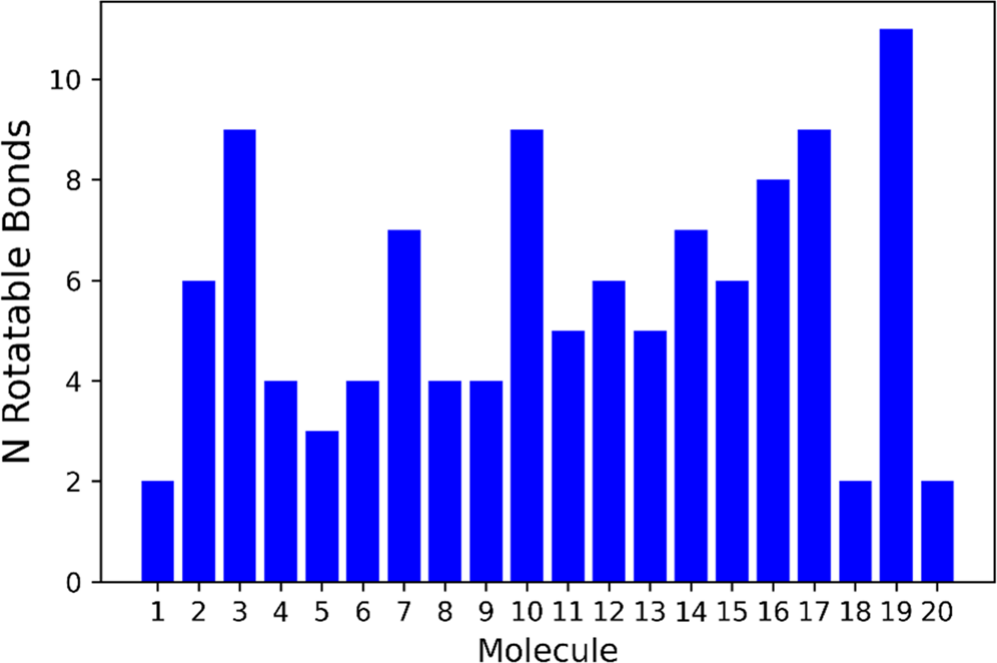
Number of rotatable bonds of each molecule in the data set; the structures that correspond to the numbers on the *x*-axis are shown in Figures 1 and 2.

Figure S8 in the SI shows how the average RMSD between the force field and DFT conformer geometries varies for each molecule in the data set. For all of the force fields, the RMSDs of molecule 19 tend to be higher than those of other molecules, which is potentially due to the high conformational flexibility and the complex structure of this molecule. Molecule 3 is also associated with larger RMSDs for the AMBER*, OPLS, MM2*, and MM3* force fields, and molecule 7 has some of the highest RMSDs in the OPLS3e, MMFFs, OPLS, and MM3* force fields. Additionally, molecule 10 is associated with higher average RMSD for OPLS3e, OPLS-2005, MMFF, MMFFs, AMBER*, and MM2* (*i.e.*, all of the force fields that could perform conformational searches on it) and OPLS-2005, AMBER*, and OPLS particularly struggle to predict the geometries of the conformers of molecule 15. All of the above-mentioned molecules have higher numbers of rotatable bonds compared with the other molecules in this data set (see also Figure 4), and all are quite conformationally flexible (it thus makes intuitive sense why the force fields may have greater RMSDs with these molecules since greater numbers of conformers give greater opportunity for the force field to incorrectly predict the geometries of these conformers). Interestingly, however, the RMSDs for molecules 16 and 17 do not tend to stand out compared with the above-mentioned molecules despite the fact that they are just as flexible and have similar numbers of rotatable bonds.

In conformational searching, it is common to thoroughly search the conformational space and return a selection of the lowest-energy conformers to the user. This is often performed to reduce the computational and time-based costs associated with taking all observed structures forward to DFT level calculations. Table S10 (see SI) shows the proportion of molecules for which the force fields were able to predict the lowest-energy conformer, out of the number of molecules for which each force field was parameterized. Most of the force fields correctly find the lowest conformers for approximately the same proportion of molecules, but OPLS often found greater numbers of lowest-energy conformers than all other force fields and is followed by MMFF and MM2* in terms of performance. However, for molecules with a large number of conformers, a force field has a reduced chance of finding the lowest DFT conformer. Force fields are very often limited by their accuracy, and as a molecule becomes larger and more flexible, the likelihood that the combination of substructures in the molecule will be absent from the force field’s parameterization will increase, and therefore the chance that the force field will be able to correctly assign the conformers to their proper energy range decreases. Thus, for each molecule, we also determined the number of conformers that the force field predicted to be within a 10 kJ mol^–1^ energy window above the lowest-energy conformer, which were also within the 10 kJ mol^–1^ window according to DFT (should the force field find conformers 10 kJ mol^–1^ above the minimum). Figure 3e plots the mean values of the ratios between the number of conformers within 10 kJ mol^–1^ of the minimum (predicted by the force field) and the number of these conformers that were in fact within 10 kJ mol^–1^ of the DFT minimum. This plot indicates that the OPLS, MMFFs, and AMBER* force fields are the best at predicting which conformers are within 10 kJ mol^–1^ of the lowest-energy conformer according to DFT. The best-performing force fields by this measurement are those that are more likely to yield better results if a screening or selection of low-energy conformers is required for a study. The OPLS force field is again found to be useful for predicting low-energy conformers. Of all of the molecules in the data set, Figure S10 in the SI shows that for molecule 8, all of the force fields tend to perform comparatively poorly at predicting which conformers of this molecule are of low energy. That identifying the low-energy conformers of this molecule is problematic for the force fields is interesting, given that (as seen in Table S1 in the SI) this molecule does not tend to produce extremely large numbers of conformers, compared with a molecule such as 3, for which the conformational searches find larger numbers of conformers and the force fields tend to have worse performance at predicting the low-energy conformers. It would seem therefore that the low-energy conformers of this molecule (8) are fairly challenging for force fields to correctly identify.

The next assessment was to perform redundant conformer eliminations on the DFT-optimized structures with a small RMSD cutoff (0.1 Å), thereby eliminating any close to identical structures following DFT optimization. Here, the ideal force field would provide a set of conformers where each structure converges to a unique minimum on the DFT potential energy surface. This ensures that computational resources and time are not wasted during the reaction modeling process. For each force field and each molecule, we calculated the ratios between the number of conformers left following redundant conformer elimination and the numbers of conformers found by the force field. The mean values of these ratios over all molecules are plotted in Figure 3f. OPLS, MM3*, and MMFFs show the best performance here by a small margin, indicating that on average, these force fields find the lowest numbers of nonredundant conformers. This feature would be useful when exploring the conformational space of large chemical systems and may help to reduce the computational time wasted in optimizing redundant conformers. Figure S12 in the SI shows that molecule 6 tended to have greater numbers of redundant conformers following DFT optimization for all of the force fields except MM3* (which could not perform the conformational search on this molecule). It would seem that the unusually large and flexible 10-membered ring system in this molecule has caused the force fields to generate large numbers of unstable conformers, which is again likely due to a gap in the parameterization of these force fields.

Finally, after DFT optimization and a 0.1 Å RMSD redundant conformer elimination, the different force fields were often found to have different numbers of conformers remaining. It would be desirable for a force field to find as many as possible of the actual stable conformers of a molecule from its search. Thus, for each molecule, the final numbers of conformers for each force field were divided by the maximum number of conformers found by any of the force fields for that molecule. Figure 3g shows the mean values of these ratios between the number of final conformers found for each molecule and the maximum number of conformers found by any force field for that molecule. This plot indicates which of the force fields tend to find the greatest proportion of the maximum numbers of stable conformers found, and the greatest values here come from OPLS3e, MM2*, and AMBER*. For the sake of completeness, the maximum numbers of conformers for each of the molecules found after DFT optimization and the redundant conformer elimination are shown in Table 2.

**Table 2:** Maximum Numbers of Conformers from Any of the Conformational Searches for Each Molecule after DFT Optimization and Then Redundant Conformer Elimination That Removed Any Conformers That Had an RMSD between Geometries Lower Than 0.1 Å (*i.e.*, Those That Had Collapsed to Essentially the Same Geometry during DFT Optimization)

molecule	number of distinct DFT conformers	molecule	number of distinct DFT conformers
1	1	11	36
2	32	12	48
3	235	13	83
4	15	14	22
5	10	15	46
6	22	16	26
7	22	17	122
8	43	18	5
9	49	19	117
10	83	20	67

Figure S14 in the SI provides greater detail on what proportions of the maximum numbers of conformers each molecule had at the DFT level each force field was able to locate. For this metric, there does not seem to be a specific set of molecules that presents a significant hurdle for the force fields, but it is apparent from this plot that, again, OPLS3e and MM2* tend to be efficient at finding conformers that are stable at the DFT level but are not redundant.

As a final side note for this section, we have performed some additional small tests that are detailed in the SI Section 11. The first of these was to perform a conformational search on molecule 5, using the conformer–rotamer ensemble sampling tool (CREST) and the second was to perform a conformational search on molecule 1 bound to methyl vinyl ketone using the OPLS3e force field. The conformers from both of these searches were optimized with the same DFT level of theory, and the same performance metrics were computed. In short, the results of these tests did not yield good performance as for the others in this work. Please refer to the SI Section 11 for further details of these results.

### 3.2 Specific Intramolecular Interactions in the Lowest-Energy Conformers

As mentioned above, the molecules of this data set were chosen partly due to their unusual structures and electronic interactions (particularly conjugation and internal hydrogen bonding) that may be challenging to force fields. Therefore, for this section, we have examined the very lowest energy conformers of all of the molecules at the DFT level for these electronic interactions and found that 7 of the 20 molecules in this study have an obvious electronic interaction in their lowest-energy conformer at the DFT level. For the molecules that contain these stabilizing interactions, we have analyzed the lowest-energy conformers predicted by the force fields to determine if they have correctly accounted for these interactions. For the sake of brevity, here we present the analysis of three of the seven molecules (3, 5, and 7), and we direct the reader to further analysis of the other four molecules (8, 10, 19, 20), which may be found in the SI Section 10.

**Figure 5:**
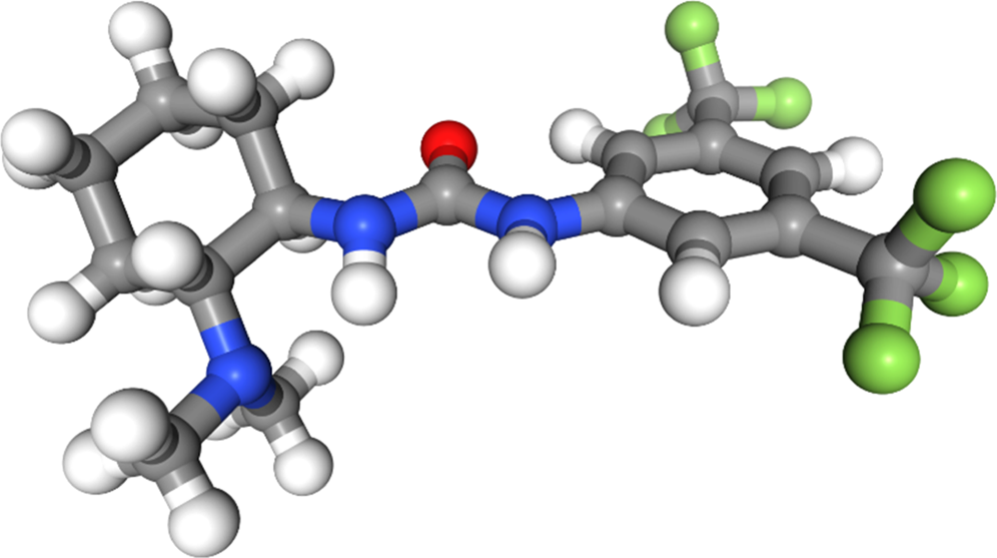
Lowest-energy conformer of molecule 3 from DFT optimization.

**Figure 6:**
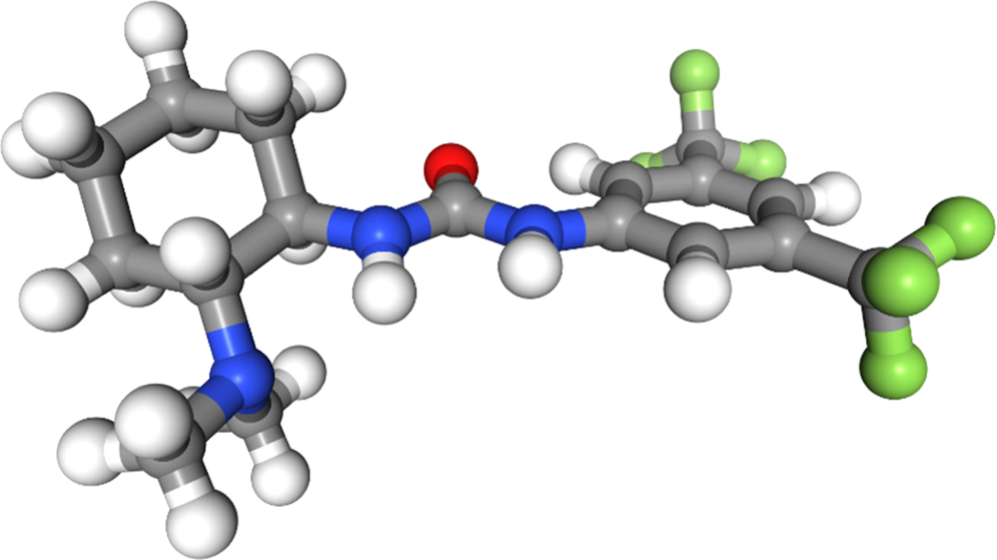
Lowest-energy conformer of molecule 3 from the MMFF force field.

**Figure 7:**
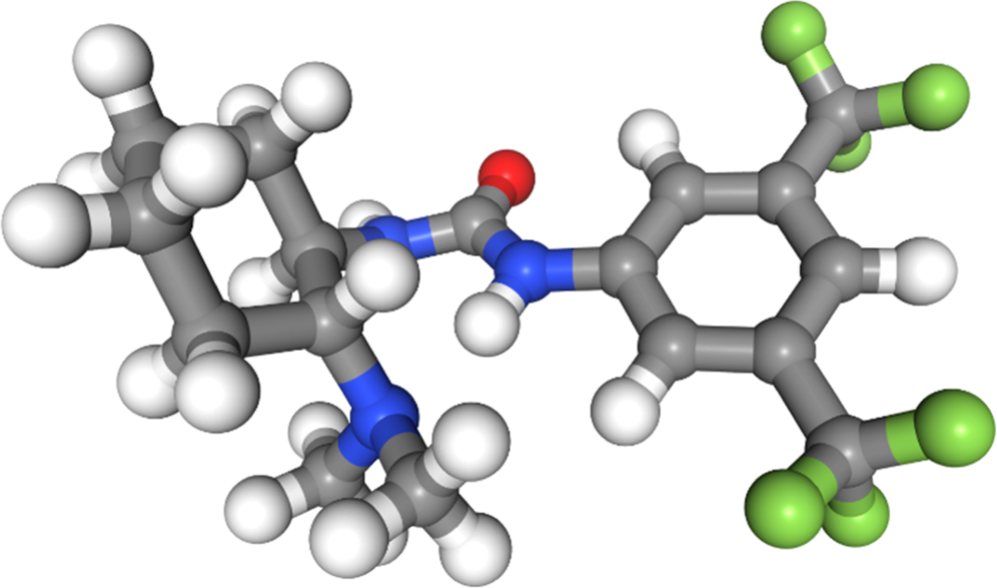
Lowest-energy conformer of molecule 3 from the OPLS3e force field.

The lowest-energy conformer of molecule 3 at the DFT level is shown in Figure 5, and as seen, there is a clear conjugative interaction between the urea group and the trifluoromethylated aryl ring due to the planarity between the two groups. The only force fields to identify this effect in their predicted lowest-energy conformers were MMFFs and MMFF (see also Figures 6 and S16 in the SI). The OPLS3e force field found the geometry between the aryl ring and the urea group to be close to planar, but the stabilization from a hydrogen bond between the urea group and the dimethylamine group attached to the cyclohexane ring appears to be overestimated relative to DFT (Figure 7). None of the other force fields found the same planar geometries in their lowest-energy conformers (see also Figures S15 and S17–S20 in the SI).

**Figure 8:**
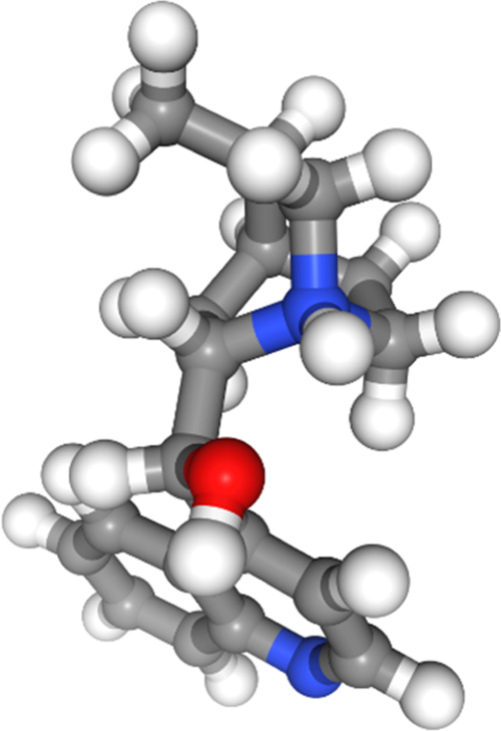
Lowest-energy conformer of molecule 5 from DFT optimization.

**Figure 9:**
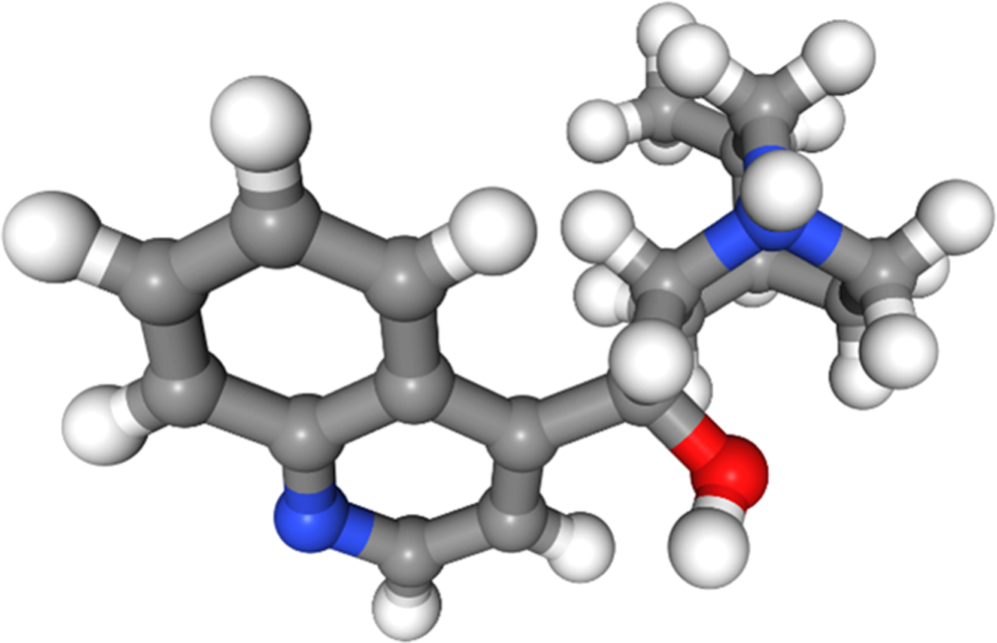
Lowest-energy conformer of molecule 5 from the OPLS3e force field.

The lowest-energy conformer of molecule 5 at the DFT level contains a hydrogen bond between the hydroxyl group and the hydrogen on the protonated amine, as seen in Figure 8. The only force field that did not find this hydrogen bond to be stabilizing is OPLS3e (Figure 9), whereas this interaction is present in all of the lowest-energy conformers for all of the other force fields (see also Figures S21–S26 in the SI).

**Figure 10:**
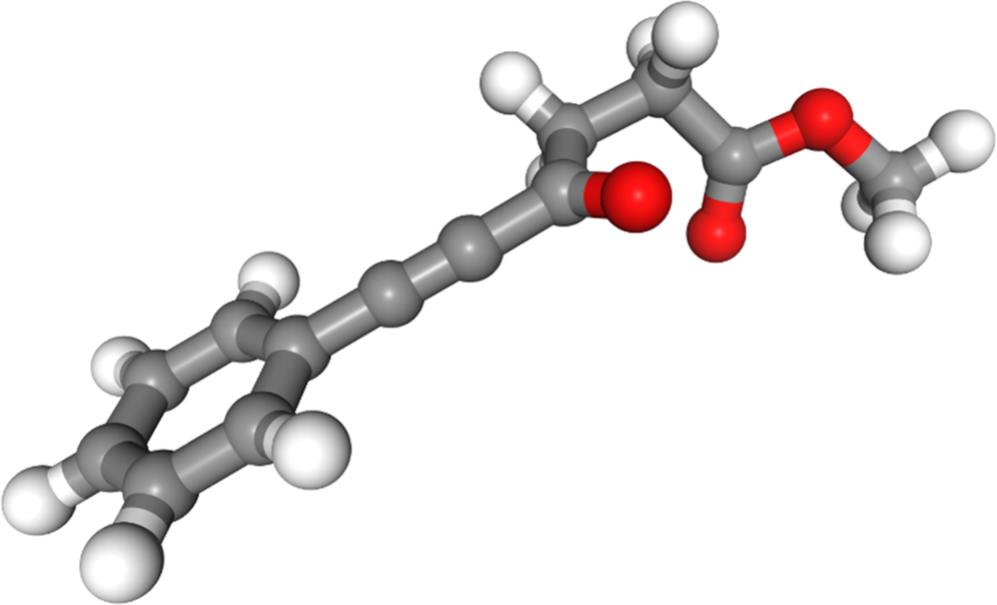
Lowest-energy conformer of molecule 7 from DFT optimization.

**Figure 11:**
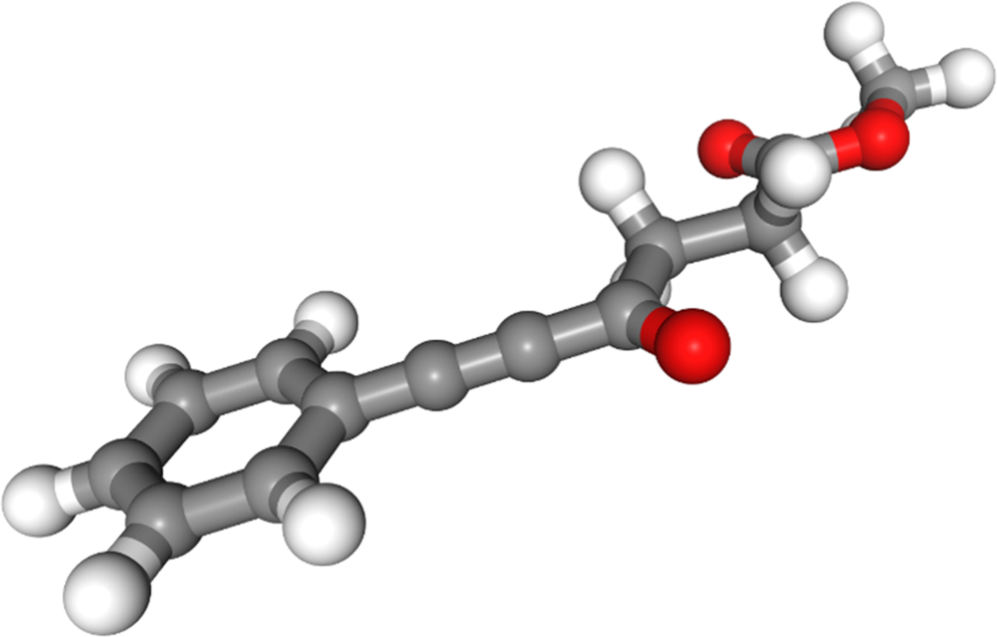
Lowest-energy conformer of molecule 7 from the MM3* force field.

Molecule 7 also has a conjugative interaction between the phenyl ring, the alkyne bond, and the carbonyl bond (Figure 10). The only force field that finds the aryl ring in plane with the carbonyl group is MM3* (Figure 11); all of the other force fields (Figures S27–S32 in the SI) do not find the aryl ring to be in plane with the carbonyl group and thus do not seem to find the conjugative interaction to be particularly stabilizing. On the other hand, all of the force fields correctly find that the s-*cis* ester arrangement is more stabilizing than the s-*trans* conformation; none of the force fields find the s-*trans* in the lowest-energy conformer.

## 4 Conclusions

In summary, we assessed the performance of several force fields in the task of conformationally searching 20 molecules, most of which are hydrogen-bond-donating catalysts, a highly useful class of compounds with applications, for example, in asymmetric catalysis.^1,25^ The criteria used for assessing force field performance were their abilities to predict the relative energies and geometries of the molecule conformers, the force fields’ ability to correctly predict which conformers are of low energy, the proportions of the conformers that were not redundant following DFT optimization, and the proportions of the maximum final numbers of conformers for each molecule found by each force field. Overall, OPLS3e, MMFFs, and MM3* are most frequently found to be the highest-performing force fields, and each performs well in five of the seven metrics considered. For each of the seven metrics considered, Table 3 shows which of these three force fields had one of the top three best performances in that metric.

**Table 3:** For Each of the Generally Best-Performing Force Fields, the Table Shows Whether That Force Field Was One of the Top Three Best Force Fields in Each of the Seven Metrics Used to Assess Force Field Performance^a^

	OPLS3e	MMFFs	MM3*
Spearman	yes	yes	yes
*R*^2^	yes	yes	yes
MAD	yes	no	yes
RMSD	yes	yes	yes
Low E	no	yes	no
nonredundant	no	yes	yes
Max	yes	no	no

a Spearman coefficient between DFT and force field energies, *R*^2^ coefficient between DFT and force field energies, MAD between DFT and force field relative energies, RMSD between DFT and force field geometries, proportion of low energy (less than 10 kJ mol^–1^ above the very lowest energy conformer) correctly identified to be low energy, number of nonredundant conformers found, and the proportion of the maximum possible number of conformers found.

At this point, it is probably worth noting that one should be critical of the accuracies of force fields (particularly when considering energetic predictions) in any chemical investigation that is making use of them. For instance, the greatest average Spearman coefficient for any of the force fields was 0.696 (from OPLS3e) and the lowest average MAD between the force field and DFT conformer relative energies was 6.6376 kJ mol^–1^ (also OPLS3e). This is not to say that force fields should not be used at all for investigations in computational chemistry, but that one should be wary of the deviation between the force field and higher-level energetic predictions.

We have also examined the lowest-energy conformers of the molecules from DFT to identify any stabilizing electronic interactions that may be present in these conformers and compared these with the lowest-energy conformers found by the force fields to determine if they had also correctly found the stabilizing interactions. The results of this analysis indicate that relying on any of the force fields in this work (even those that generally perform better at predicting the energies and geometries of the conformers) to accurately and reliably determine which electronic interactions will be the most stabilizing in complex molecules such as those in this data set is probably not wholly advisable. For example, OPLS3e overestimates the stabilization from a hydrogen bond in molecule 3, but it was the only one of the force fields that did not have a hydrogen bond in the lowest-energy conformer of molecule 5. Likewise, MMFF and MMFFs correctly identify the stabilizing conjugation in molecule 3 but do not predict the conjugation in molecule 7 and overestimate the stabilizing effect of conjugation in molecule 19 (see the SI Section 10). This said, a previous benchmark of force fields for describing noncovalent interactions^28^ (specifically hydrogen bonding and π-stacking) found that the MMFF94 and OPLS force fields are generally better for predicting these interactions, which perhaps explains the overall better performances of the MMFFs and OPLS3e force fields in this work.

MM3* was consistently at the top or within the top three force fields, except when predicting the very lowest energy conformer and finding the overall total number of conformers, and it was fourth best for finding the maximum final numbers of conformers. However, the main drawback of MM3* is in its parameterization. MM3* was only parameterized for 12 of the 20 molecules, the lowest number observed for all force fields in this study (aside from AMBER94, which was parameterized for one of the molecules and DFT optimizations were not performed on these conformers). It appears that MM3* is a comparatively strong force field for finding the energies, geometries, and nonredundant conformers from the conformational searching of hydrogen-bond-donating catalysts but over a more limited range of molecules. For conformer energies and geometries, the OPLS3e force field is one of the most consistently successful at predicting the values from DFT, and it is also the best force field for finding the greatest number of final conformers of the hydrogen-bond-donating catalysts. Finally, MMFFs have produced stronger results with the metrics measuring conformer energy ordering, strength of correlation with DFT, geometry, predicting low-energy conformers, and proportions of nonredundant conformers. Two “MM” force fields (MM3* and MMFFs) performed generally better than other force fields in this study, aligning well with findings from our previous analysis.^7^ Further, it is reassuring that the most recently parameterized force field, OPLS3e, tends to provide a better description of conformer energies and geometries when compared to older force fields, given that it takes advantage of greater amounts of modern experimental and quantum mechanical data for its parameterization.^20^ Considering the recent improvements to this force field in the form of OPLS4,^29^ this force field is likely to remain a strong choice. It should be noted that the findings of this study are applicable to hydrogen-bond-donating catalysts with similar structures to those in this data set; however, based on the results, we conclude that MM3*, MMFFs, and OPLS3e are likely to generally be the better options for conformationally searching these molecules.

## Supporting Information Available

Additional details of conformational searching and DFT optimization, tables containing numerical values for metrics plotted in Figures 3–5, three-dimensional (3D) visualizations of lowest-energy conformers of molecules discussed in Section 3.2 from other force fields; the structures of all conformers of all of the molecules, found with each force field, before and after DFT optimization are available from the University of Bath Research Data Archive (https://doi.org/10.15125/BATH-01065)^30^ (PDF)
