# Supplementary material for: Comparing the Performances of Force Fields in Conformational Searching of Hydrogen-Bond-Donating Catalysts
Source: J Org Chem. 2022 Apr 27;87(9):5703–12. doi: 10.1021/acs.joc.2c00066 (PMC9087191; doi:10.1021/acs.joc.2c00066)
Supplement: Supplementary file 1 — jo2c00066_si_001.pdf [file jo2c00066_si_001.pdf]

Comparing the Performances of Force Fields in Conformational Searching of Hydrogen Bond-Donating Catalysts

Electronic Supplementary Information

Toby Lewis-Atwell,[a] Piers A. Townsend[b] and Matthew N. Grayson*[c]

a. Department of Computer Science, University of Bath, Claverton Down, Bath, BA2 7AY, UK.

b. Centre for Sustainable Chemical Technologies, University of Bath, Claverton Down, Bath, BA2 7AY, UK.

c. Department of Chemistry, University of Bath, Claverton Down, Bath, BA2 7AY, UK.

*Corresponding author: M.N.Grayson@bath.ac.uk

Contents

1. Conformational Searches S2
2. DFT Calculations S4
3. Spearman Coefficients S5
4. R^2 Coefficients S8
5. Mean Absolute Deviations in Conformer Relative Energies S11
6. Deviations in Geometries S14
7. Low Energy Conformers S17
8. Conformers Remaining After Optimization/Redundant Conformer Elimination S22
9. Proportions of Maximum Numbers of Conformers Found S26
10. Conformer Electronic Interactions S29
Molecule 3 ..... S29
Molecule 5 ..... S32
Molecule 7 ..... S35
Molecule 8 ..... S37
Molecule 10 ..... S42
Molecule 19 ..... S45
Molecule 20 ..... S50
11. Additional Minor Tests S55
CREST Conformational Search on Molecule 5 ..... S55
Conformational Search of MVK-Bound Molecule 1 ..... S55
References S57
Coordinates of Lowest Energy Conformers from Force Fields S58
Coordinates of Lowest Energy Conformers (DFT) S292

1. Conformational Searches

Conformational searches were performed with all nine force fields available in Schrödinger's MacroModel v12.6[1] (OPLS3e, OPLS-2005, MMFF, MMFFs, AMBER94, AMBER*, OPLS, MM2* and MM3*) on each of the 20 molecules in Figures 1 and 2 in the main manuscript. The AMBER94 force field was only able to perform conformational searches on a single molecule (structure 8 in Figure 1 in the main manuscript), and therefore, no further analysis was performed for this force field.

Aside from the force field potentials, the settings used in the conformational searches that were change from their default values are detailed as follows. The solvent was set to "None", which means conformational searches were performed in the gas-phase. The energy minimisation method used was "Polak-Ribiere Conjugate Gradient" (PRCG)[2, 3] which is a gradient descent method that updates a point **x** using the gradient of a function **g** at point **x** according to the equation:

$$\boldsymbol{x_{k+1}} = \boldsymbol{x_k} + \boldsymbol{\alpha_k d_k}$$

Where $\boldsymbol{\alpha_k}$ gives the steplength and $\mathbf{d_k}$ is the search direction, which is determined from:

$$\boldsymbol{d_k} = -\boldsymbol{g_k} + \beta_k \boldsymbol{d_{k-1}}$$

Where $\mathbf{g_k}$ is the gradient of the function at point $\mathbf{x_k}$ and β_k is given by:

$$\beta_k = \frac{\boldsymbol{g_k}^{\boldsymbol{T}}(\boldsymbol{g_k} - \boldsymbol{g_{k-1}})}{\|\boldsymbol{g_{k-1}}\|^2}$$

The threshold for considering the energy to be converged was set as a change in the gradient between iterations of less than 0.001. The method used from conformational searching was "Mixed torsional/Low-mode sampling" which uses a combination of the Monte Carlo Multiple Minimum (MCMM) search method and the Low-mode conformational search method (LMCS). Briefly, MCMM[4] randomly adjusts the dihedral angles of the rotatable bonds of a molecule and performs energy minimisations on the resulting structures (in this work, using the gradient descent method described above). LMCS[5] diagonalises the Hessian matrix of the molecule's potential energy and the resulting eigenvectors (which correspond to the "normal modes" of vibration) are used to perform gradient ascent, starting from one conformer, to a saddle point between minima. Once a saddle point is reached, gradient descent is performed to reach to the minimum on the other side of the saddle point. In combination, MCMM generates a wide range of conformers by adjusting the torsions of the molecule, and LMCS searches the local area of the potential energy surface around these conformers by following their low-frequency vibrational modes.

Each molecule was searched for 5000 steps, and the energy window for saving structures was set to 50 kJ mol^{-1}, which means that only conformers with energies within that threshold of the lowest energy conformer were retained. The maximum atom deviation for structures to be considered different conformers was set to 0.25 Å, which means that structures with any atom further apart than that threshold are considered distinct conformers.

Following the conformational searches with molecules 3, 10, 12, 13 and 17, some of the force fields produced too many conformers to optimise with DFT within a practical timeframe. Therefore, redundant conformer eliminations were performed on the conformers of these molecules using the redundant conformer elimination tool in MacroModel. Conformers were eliminated using the root mean square deviation (RMSD) between conformer structures as the criterion for determining conformer similarity. For each molecule, a RMSD cutoff was chosen such that any conformers that had geometries closer than this cutoff were eliminated and the lower energy conformer was retained. The same redundant conformer elimination was applied to the conformer sets from all of the force fields for each of the five molecules. The chosen RMSD cutoffs for each for each of the five molecules are shown in the right-hand column of Table S1. Table S1 also shows the numbers of conformers of each molecule found by each force field, along with the numbers of conformers left after redundant conformer elimination, if performed.

Note that when the structure does not contain a delocalised trigonal nitrogen centre, the results from MMFF and MMFFs will be identical,[6] in these cases the results from MMFF are also taken as those for MMFFs.

Table S1. The numbers of conformers of each molecule found by each force field. Blank cells indicate that the force field was unable to perform conformational searches on the molecule due to limited parameterization (except for MMFFs, in which case the numbers were the same as MMFF). Cells with two numbers separated by '//' indicate the numbers of conformers before and after redundant conformer elimination and the RMSD cutoff used for that molecule is found in the right-most column.

Molecule Number	OPLS3e	OPLS-2005	MMFF	MMFFs	AMBER	OPLS	MM2*	MM3*	RMSD Cutoff / Å
**1**	3	3	2		3		3		
**2**	24	28	29	30	36	24	28	20	
**3**	271//117	424//132	177//63	214//78	892//284	162//47	615//211	280//136	0.6
**4**	11	13	8	8	12	6	18	12	
**5**	8	7	7		6	5	12	7	
**6**	54	48	14		24	25	43		
**7**	32	15	8		18	18	14	17	
**8**	52	33	60	45	25	48	47	56	
**9**	36	52	59	33	29	33	60		
**10**	274//115	27//13	49//28		37//29		240//126		0.8
**11**	44	16	24	16	12	8	48	30	
**12**	206//55	81//18	243//46	190//33					0.6
**13**	124//81	70//40	86//46	47//25	220//104	73//26	94//28	161//46	0.5
**14**	27	20	9				16	21	
**15**	56	25	44		78		22		
**16**	34	16	21	21	31	24	22	19	
**17**	225//91	267//86	379//125	365//122					0.6
**18**	5	4	3		4		12		
**19**	90	46	49	42	108	69	153	112	
**20**	81	26	29		43	9	81	58	

2. DFT Calculations

All DFT calculations were performed using the Gaussian 16, Revision A.03 software.[7] Geometry optimizations were performed on all conformers of all molecules at the M06-2X/6-31G(d) level of theory in the IEFPCM(benzene) solvent model. Once all of the conformer structures had reached minima and were stationary points, single-point energies were calculated at the M06-2X/def2-TZVPP IEFPCM(benzene) level of theory. This level of theory was chosen since it has previously been used with success in reaction modelling studies.[8-10] The quasi-harmonic Gibbs free energies (using Grimme's quasi-harmonic treatment of entropy[11]) from M06-2X/6-31G(d) were corrected using the single-point energies from M06-2X/def2-TZVPP, and these corrected free energies were used as the energies to which the force field energies were compared. The GoodVibes python library[12] was used for the extraction of all DFT thermochemical data.

3. Spearman Coefficients

The Spearman rank correlation coefficient is calculated by first changing the observed values of two random variables (e.g. X and Y) into ranked values (r_X, r_Y which replace the values of X and Y with the indices of their positions in an ordered list of the observed values), and then the same formula for Pearson's coefficient is applied to the ranked values:

$$r_{Spearman} = \frac{cov(r_X, r_Y)}{\sigma_{r_X}\sigma_{r_Y}}$$

Where σ_{rX} and σ_{rY} are the standard deviations of the ranked variables. In this work, the Python module *scipy.stats.spearmanr* was used to calculate the Spearman coefficients between the force field and single-point corrected DFT free energies of all the conformers of each molecule found with each force field. Table S2 gives all of the values of the Spearman coefficients for all force fields and all molecules, and Table S3 gives the mean values for each force field, which are also plotted in Figure 3 a) of the main text.

Table S2. Spearman coefficients for all molecules and all force fields. Empty cells indicate molecules for which a given force field was unable to perform conformational searching. Each coefficient is calculated between the force field energies of all the conformers of a molecule found by that force field, and the corresponding single-point corrected DFT free energies.

Molecule Number	OPLS3e	OPLS-2005	MMFF	MMFFs	AMBER	OPLS	MM2*	MM3*
**1**	0.5	0.5	1	1	-0.5		-0.866	
**2**	0.9426	0.7392	0.9483	0.9581	0.9587	0.9724	0.9513	0.603
**3**	0.713	0.4645	0.8419	0.8795	0.7519	0.6855	0.6299	0.5466
**4**	0.8727	0.6703	0.1667	0.1667	0.3916	0.6	-0.3101	0.2028
**5**	0.7619	0.9286	0.6786	0.6786	0.1429	0.3	0.8722	0.8571
**6**	0.8173	0.8802	0.9824	0.9824	0.9157	0.4498	0.8244	
**7**	0.9258	0.8538	0.8623	0.8623	0.8883	0.7453	0.9692	0.8999
**8**	0.7724	0.6862	0.4958	0.6181	0.92	0.7416	0.669	0.6114
**9**	0.4748	0.3376	0.2775	0.5005	0.5217	0.6208	0.6431	
**10**	0.5257	0.1758	0.2638	0.2638	0.1788		0.4494	
**11**	0.2848	-0.1441	0.1426	0.2676	-0.0769	-0.5714	0.0949	0.4526
**12**	0.5187	0.6202	0.6148	0.608				
**13**	0.7812	0.643	0.583	0.53	0.5122	0.7134	-0.0892	0.5159
**14**	0.8356	0.919	0.4547	0.4547			0.6558	0.8976
**15**	0.7954	0.9369	0.9521	0.9521	0.7724		0.8679	
**16**	0.784	0.95	0.5198	0.6872	0.9195	0.9626	0.8576	0.9596
**17**	0.7654	0.268	0.6643	0.7821				
**18**	0.3	-0.6325	1	1	-0.7746		0.0638	
**19**	0.7473	0.6002	0.4242	0.207	0.4014	0.2984	0.4875	0.7983
**20**	0.8013	0.8272	0.8321	0.8321	0.9145	0.7	0.5871	0.917

Table S3. The mean values of the Spearman coefficients from Table S2 for each force field that are also plotted in Figure S1.

OPLS3e	0.696
OPLS-2005	0.5612
MMFF	0.6352
MMFFs	0.6615
AMBER*	0.4611
OPLS	0.5553
MM2*	0.4643
MM3*	0.6885

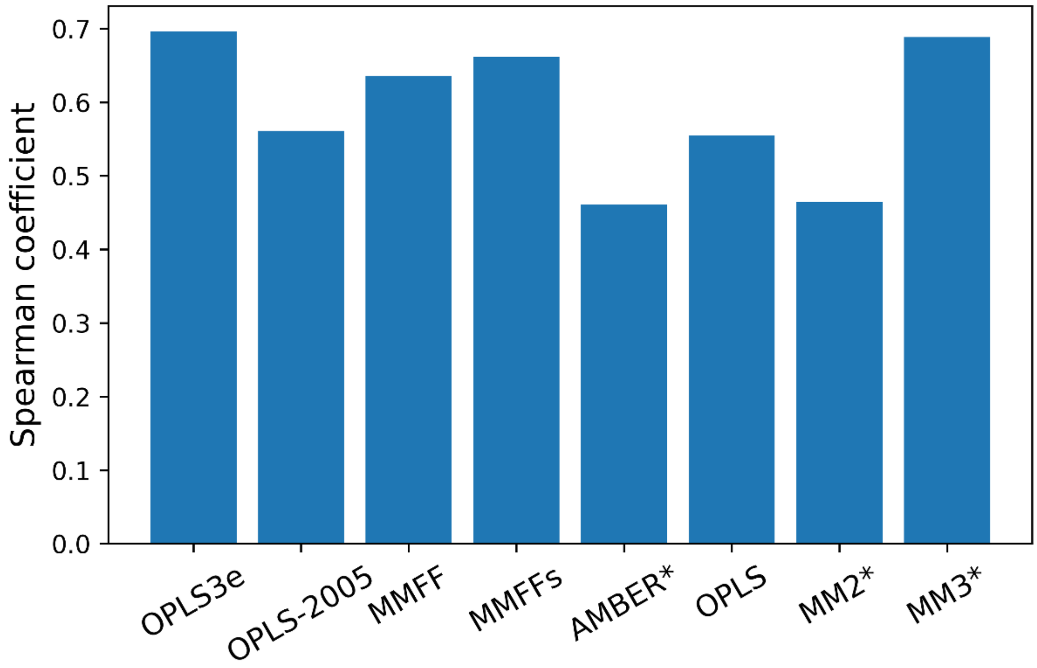

Figure S1. The mean values of the Spearman coefficients between the force field and DFT energies, for all the molecules for which each force field was able to perform conformational searches.

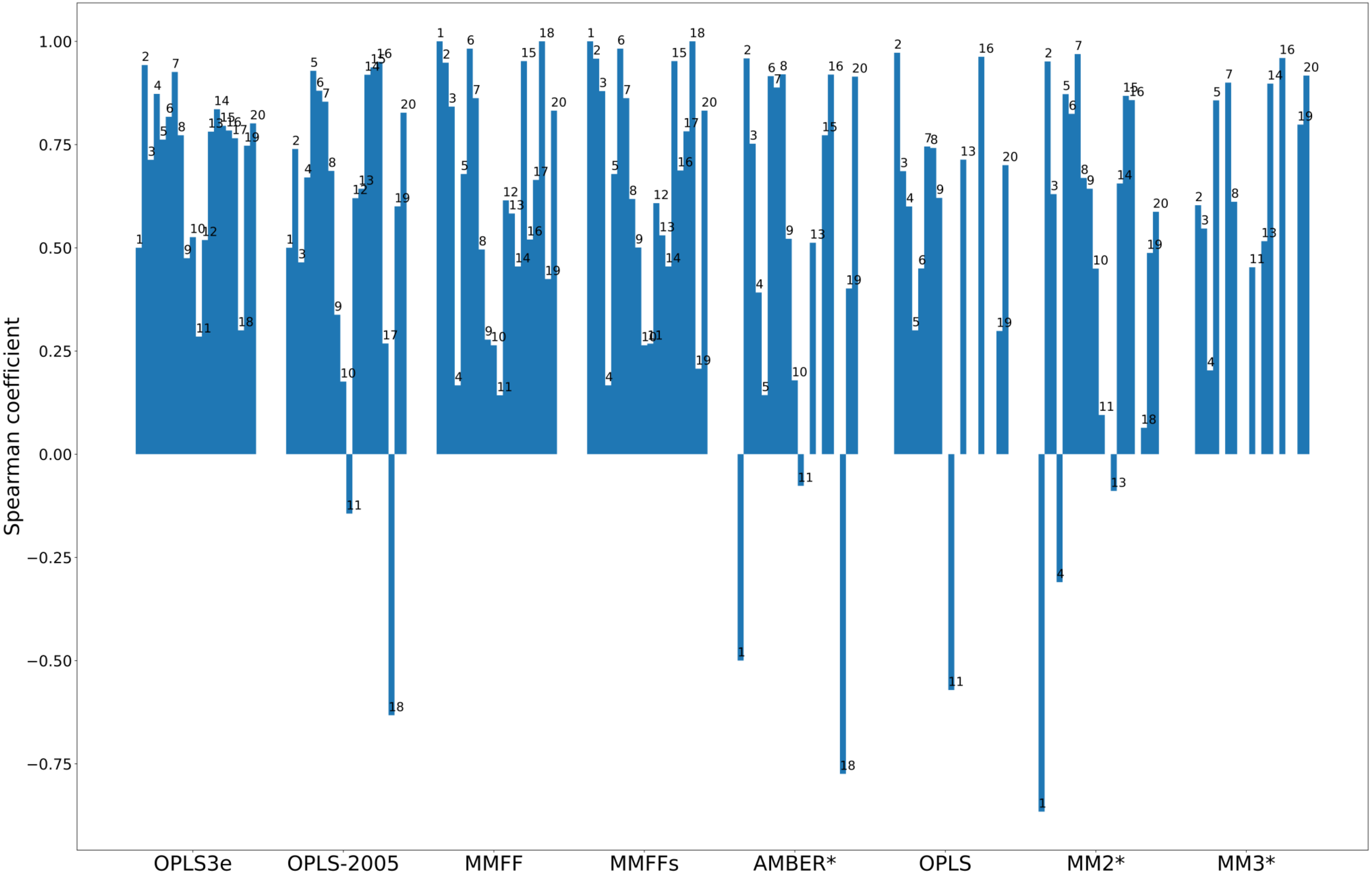

Figure S2. The Spearman coefficients between the force field and DFT conformer energies for each molecule individually.

4. R^2 Coefficients

The Pearson correlation coefficient between two variables (X and Y) is calculated as:

$$r_{Pearson} = \frac{cov(X,Y)}{\sigma_X \sigma_Y}$$

Where σ_X and σ_Y are the standard deviations of X and Y. In this case, the two variables are the values of the force field energies of all the conformers of one molecule found by that force field, and the values of the single-point corrected DFT free energies. The Python module *scipy.stats.linregress* was used to calculate the Pearson coefficients for all the molecules with each force field, and the squares of these values (R^2) are given in Table S4.

Table S4. The R^2 coefficients between the force field and single-point corrected DFT free energies for all conformers of each molecule found with each force field. Empty cells indicate molecules for which a given force field was unable to perform conformational searching.

Molecule Number	OPLS3e	OPLS-2005	MMFF	MMFFs	AMBER	OPLS	MM2*	MM3*
**1**	0.9624	0.9944	1	1	0.9176		0.9403	
**2**	0.947	0.9719	0.974	0.9564	0.8814	0.9124	0.996	0.9303
**3**	0.5777	0.2525	0.7331	0.8158	0.7415	0.4732	0.491	0.3749
**4**	0.7965	0.6829	0.1935	0.1839	0.2641	0.6299	0.0792	0.118
**5**	0.901	0.7482	0.5495	0.5495	0.1315	0.2325	0.8682	0.7645
**6**	0.6983	0.8636	0.9805	0.9805	0.733	0.4164	0.6919	
**7**	0.954	0.9378	0.9629	0.9629	0.8842	0.8082	0.969	0.9551
**8**	0.5928	0.5701	0.2746	0.3837	0.8771	0.4971	0.4282	0.3357
**9**	0.2423	0.1833	0.1313	0.3008	0.4054	0.5331	0.4666	
**10**	0.2481	0.0414	0.0716	0.0716	0.0024		0.2084	
**11**	0.0877	0.0196	0.0232	0.1322	0.0508	0.8177	0.0018	0.1489
**12**	0.3651	0.4031	0.3734	0.3777				
**13**	0.7204	0.5749	0.413	0.4735	0.3703	0.5798	0.0001	0.3488
**14**	0.7604	0.8326	0.8396	0.8396			0.8649	0.8574
**15**	0.7054	0.9113	0.9342	0.9342	0.6145		0.8484	
**16**	0.7247	0.9174	0.6954	0.7333	0.9233	0.9661	0.8536	0.9082
**17**	0.6223	0.1344	0.6067	0.7172				
**18**	0.3455	0.1424	0.9327	0.9327	0.3635		0.0886	
**19**	0.7658	0.5236	0.1481	0.0889	0.1897	0.1173	0.6192	0.7154
**20**	0.6237	0.7701	0.7761	0.7761	0.928	0.5222	0.2503	0.7589

Table S5. The mean values of the R^2 coefficients from Table S4 for each force field, that are also plotted in Figure S3.

OPLS3e	0.6321
OPLS-2005	0.5738
MMFF	0.5807
MMFFs	0.6105
AMBER	0.5458
OPLS	0.5774
MM2*	0.537
MM3*	0.6013

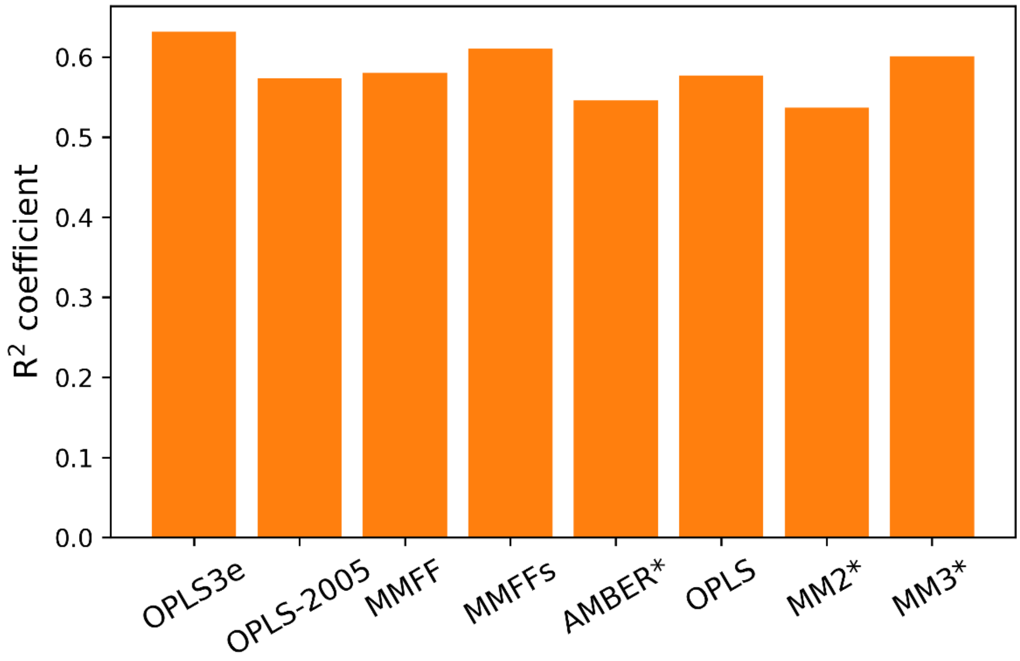

Figure S3. The mean values of the R^2 coefficients between the force field and DFT energies, for all the molecules for which each force field was able to perform conformational searches.

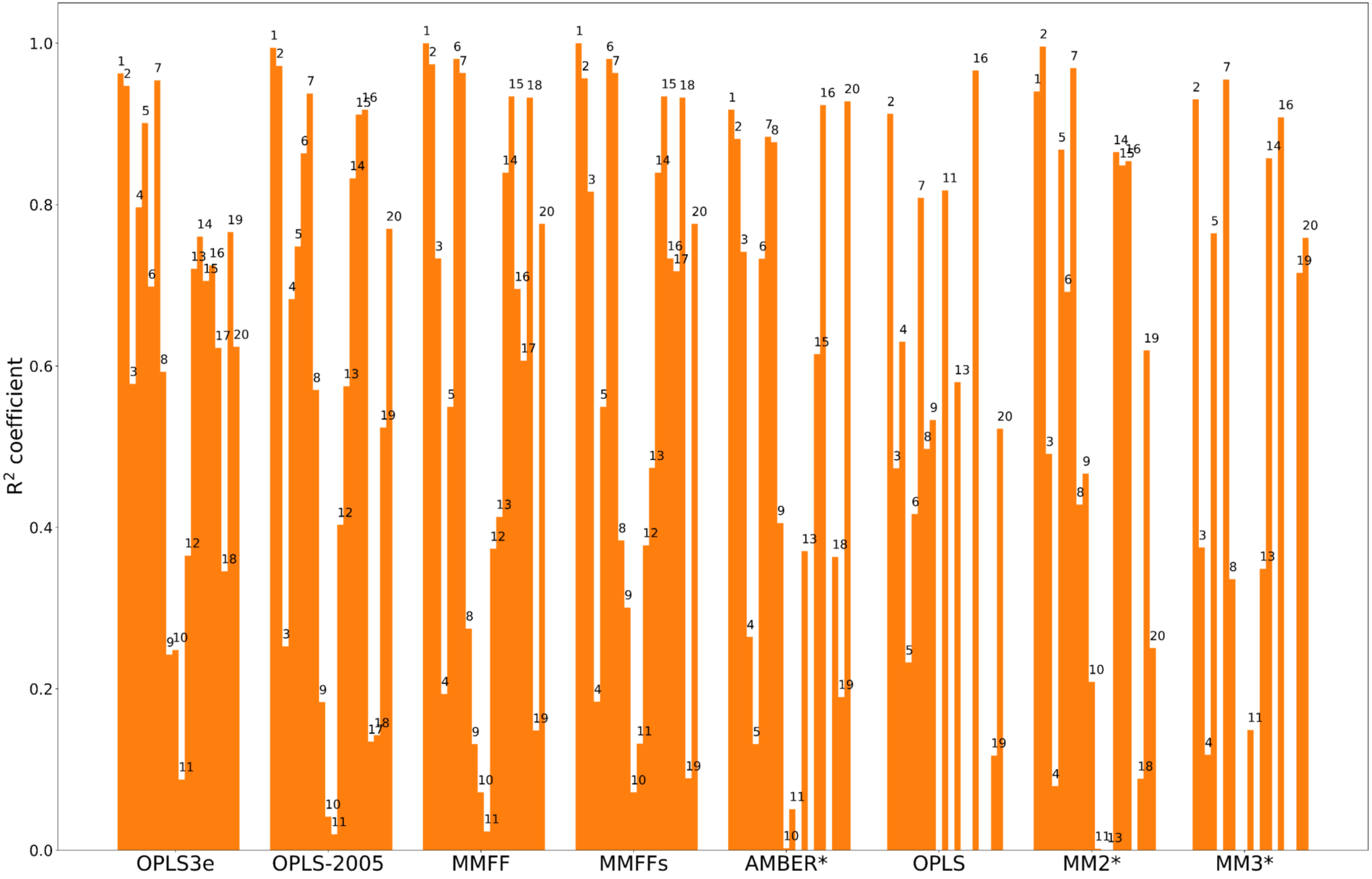

Figure S4. The R2 coefficients between the force field and DFT conformer energies for each molecule individually.

5. Mean Absolute Deviations in Conformer Relative Energies

For each molecule-force field combination, the relative energies of the conformers were calculated, *i.e.* each conformer relative energy was given by the difference between the conformer's energy minus the value of the lowest energy conformer. The absolute deviation between the force field and DFT relative free energies was calculated for each conformer, and the mean absolute deviations (MADs) for each molecule-force field combination are given by the mean of these differences over all the conformers of the molecule each force field was able to find. All of the MAD values are shown in Table S6, and the mean values of the MADs for each force field are shown in Table S7.

Table S6. The mean values of the absolute deviations between the force field and single-point corrected DFT relative conformer free energies for all the molecules and force fields.

Molecule Number	OPLS3e	OPLS-2005	MMFF	MMFFs	AMBER	OPLS	MM2*	MM3*
**1**	0.9248	0.8098	3.6834	3.6834	9.1676		2.4075	
**2**	3.9817	2.0657	2.7747	3.6978	4.9296	4.6899	2.7449	8.648
**3**	7.887	11.3268	8.9368	5.5672	4.5567	12.9924	7.1941	9.3563
**4**	4.9854	3.0206	23.3859	23.4399	4.5264	15.5848	9.2877	11.885
**5**	5.5885	11.7218	4.6573	4.6573	14.5429	26.4423	5.2189	2.4816
**6**	7.2526	3.781	2.4408	2.4408	6.5067	14.1081	5.551	
**7**	4.5707	3.4183	7.3391	7.3391	4.8781	6.6771	2.8614	4.0651
**8**	7.361	7.9887	10.5983	10.0176	5.0945	7.9867	10.3043	9.3752
**9**	13.9917	12.4511	15.2812	11.1354	8.1835	7.6663	10.9871	
**10**	9.9567	20.1165	17.2801	17.2801	19.82		13.2804	
**11**	6.052	7.736	7.8102	2.4359	3.9773	10.933	5.1853	4.3621
**12**	15.1149	14.3032	12.6788	9.3873				
**13**	5.9771	7.6502	6.1541	4.6945	6.7099	3.7885	7.0924	7.6978
**14**	5.4905	8.6113	5.6439	5.6439			4.828	8.5489
**15**	4.6843	6.0386	4.8861	4.8861	7.0534		5.5103	
**16**	5.6346	4.5697	5.5946	6.0885	3.8379	4.0465	4.5438	4.4206
**17**	7.095	12.4578	6.2325	5.608				
**18**	3.0299	0.8279	3.9092	3.9092	6.1803		4.1594	
**19**	6.4575	10.0906	16.2112	17.42	18.9455	24.5945	8.4648	9.279
**20**	6.7169	7.4847	11.8713	11.8713	9.227	3.2709	11.4116	5.7413

Table S7. The mean values of the MADs between the force field and DFT conformer relative free energies from Table S6, that are also plotted in Figure S5.

OPLS3e	6.6376
OPLS-2005	7.8235
MMFF	8.8685
MMFFs	8.0602
AMBER	8.1257
OPLS	10.9832
MM2*	6.7241
MM3*	7.1551

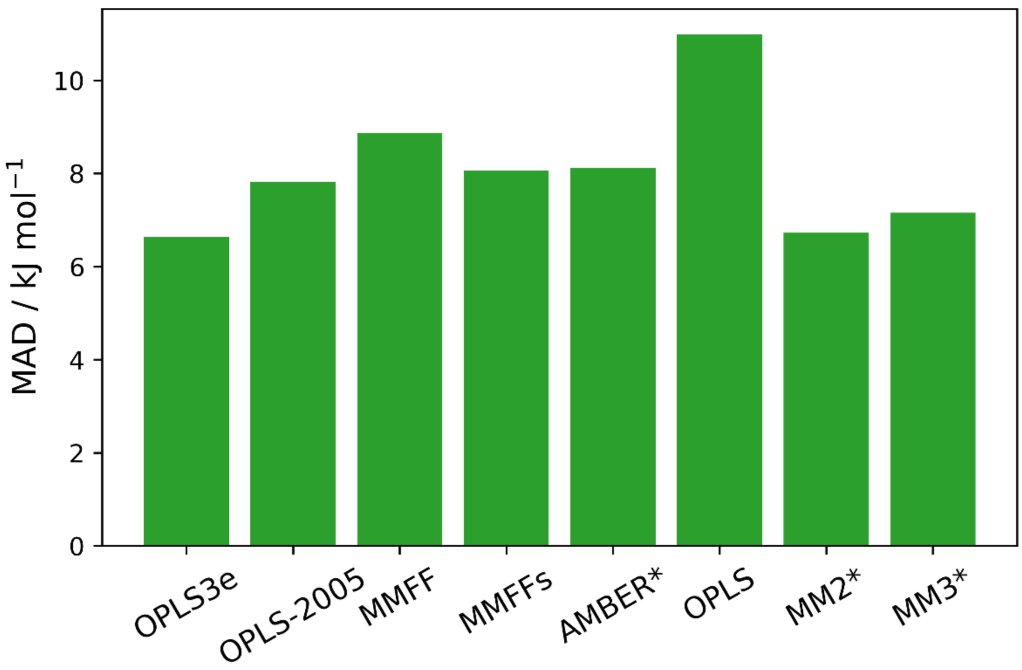

Figure S5. The mean values of the MADs between the force field and DFT relative energies, for all the molecules for which each force field was able to perform conformational searches.

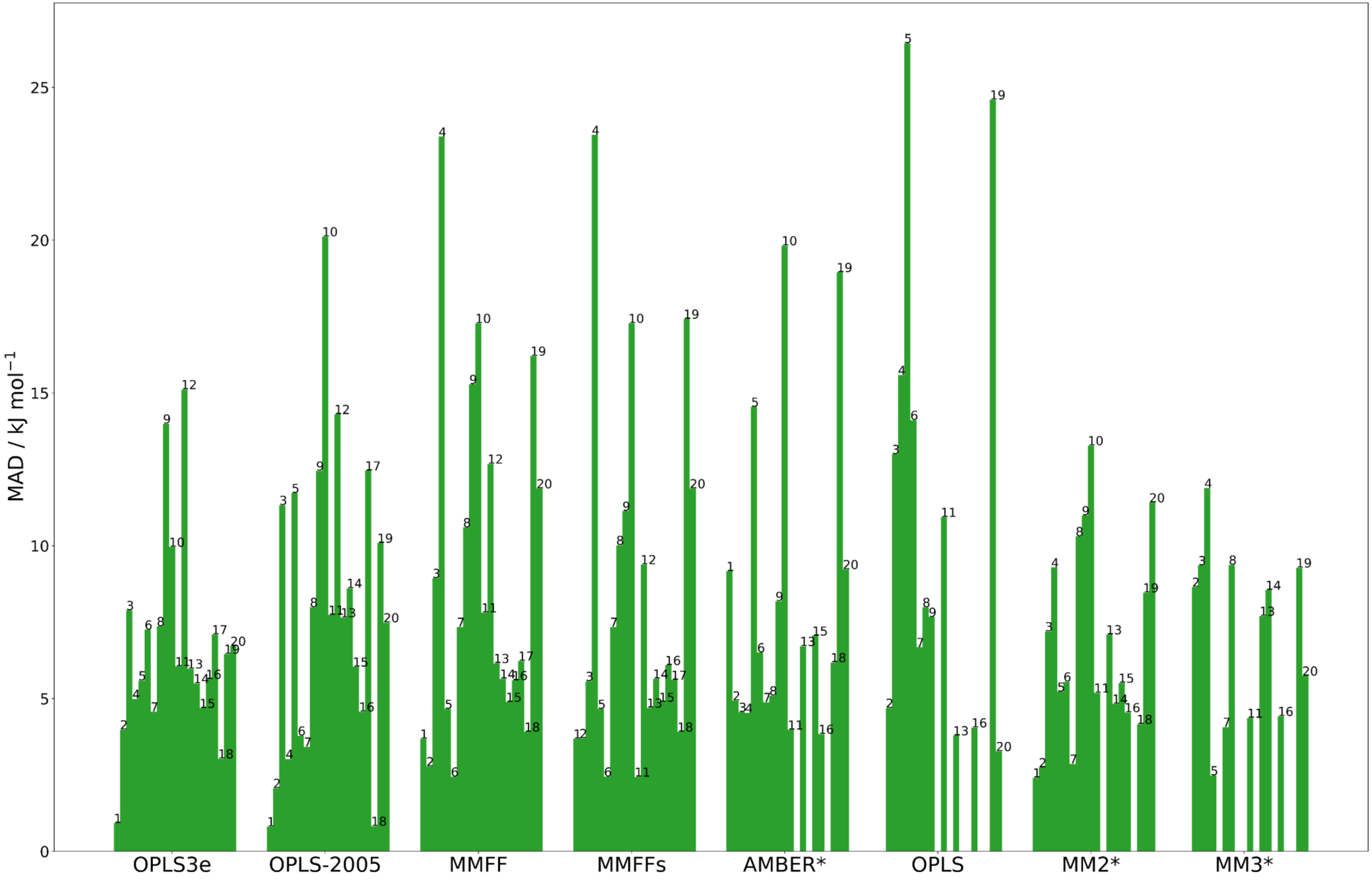

Figure S6. The MADs between the force field and DFT conformer relative energies for each molecule individually.

6. Deviations in Geometries

For each molecule-force field combination, the root mean squared deviations (RMSDs) between the geometries of the conformers optimised with the force field and DFT were calculated, and the mean values of these RMSDs for all of the molecules are shown below in Table S8.

Table S8. The mean values of the RMSDs between the force field and DFT optimised conformers for each molecule in the dataset.

Molecule Number	OPLS3e	OPLS-2005	MMFF	MMFFs	AMBER	OPLS	MM2*	MM3*
**1**	0.3464	0.3149	0.3956	0.3956	0.3477		0.331	
**2**	0.4818	0.331	0.2675	0.2423	0.3638	0.4773	0.224	0.2509
**3**	0.4484	0.4358	0.3187	0.302	0.7373	0.7414	0.6128	0.493
**4**	0.2924	0.2266	0.2834	0.2884	0.3779	0.3233	0.3219	0.1984
**5**	0.2219	0.2093	0.2189	0.2189	0.262	0.3168	0.1907	0.2443
**6**	0.1951	0.2497	0.3432	0.3432	0.2955	0.4143	0.3128	
**7**	0.639	0.7922	0.5955	0.5955	0.4803	0.747	0.4845	0.572
**8**	0.4303	0.5339	0.6495	0.5892	0.284	0.3351	0.483	0.483
**9**	0.4927	0.6954	0.6882	0.5377	0.2876	0.4033	0.6179	
**10**	0.7058	0.702	0.6396	0.6396	0.8947		0.9368	
**11**	0.3377	0.3006	0.385	0.3876	0.1938	0.2962	0.341	0.4014
**12**	0.4626	0.4475	0.4921	0.4146				
**13**	0.3727	0.498	0.4782	0.4709	0.4627	0.5464	0.6289	0.4855
**14**	0.5694	0.5644	0.5504	0.5504			0.6289	0.5024
**15**	0.5064	0.8585	0.4803	0.4803	1.1607		1.2453	
**16**	0.5704	0.2324	0.5256	0.3952	0.3536	0.3315	0.4129	0.3351
**17**	0.395	0.3329	0.4266	0.3946				
**18**	0.2283	0.1656	0.2676	0.2676	0.2219		0.2662	
**19**	0.588	0.5442	0.7883	0.6857	0.797	0.7573	0.788	0.7445
**20**	0.3023	0.1911	0.2136	0.2136	0.1448	0.2627	0.2777	0.2557

Table S9. The mean values of the mean RMSDs between force field and DFT conformer geometries from Table S8, that are also plotted in Figure S7.

OPLS3e	0.4293
OPLS-2005	0.4313
MMFF	0.4504
MMFFs	0.4207
AMBER	0.4509
OPLS	0.4579
MM2*	0.5058
MM3*	0.4139

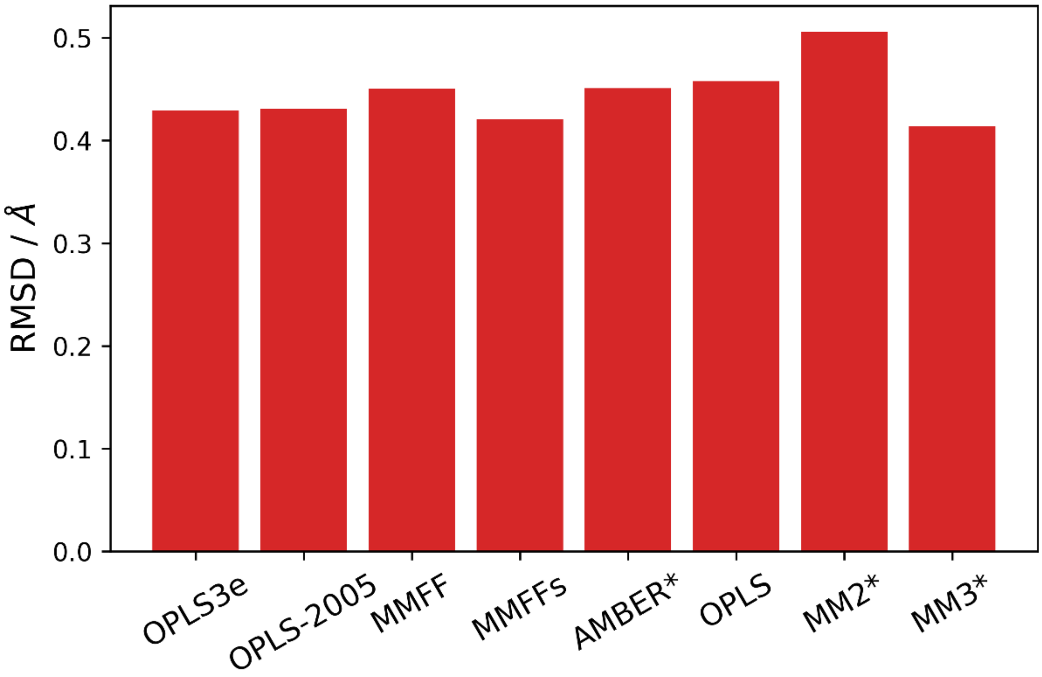

Figure S7. The mean values of the heavy-atom RMSDs between the force field and DFT structures, for all the molecules for which each force field was able to perform conformational searches.

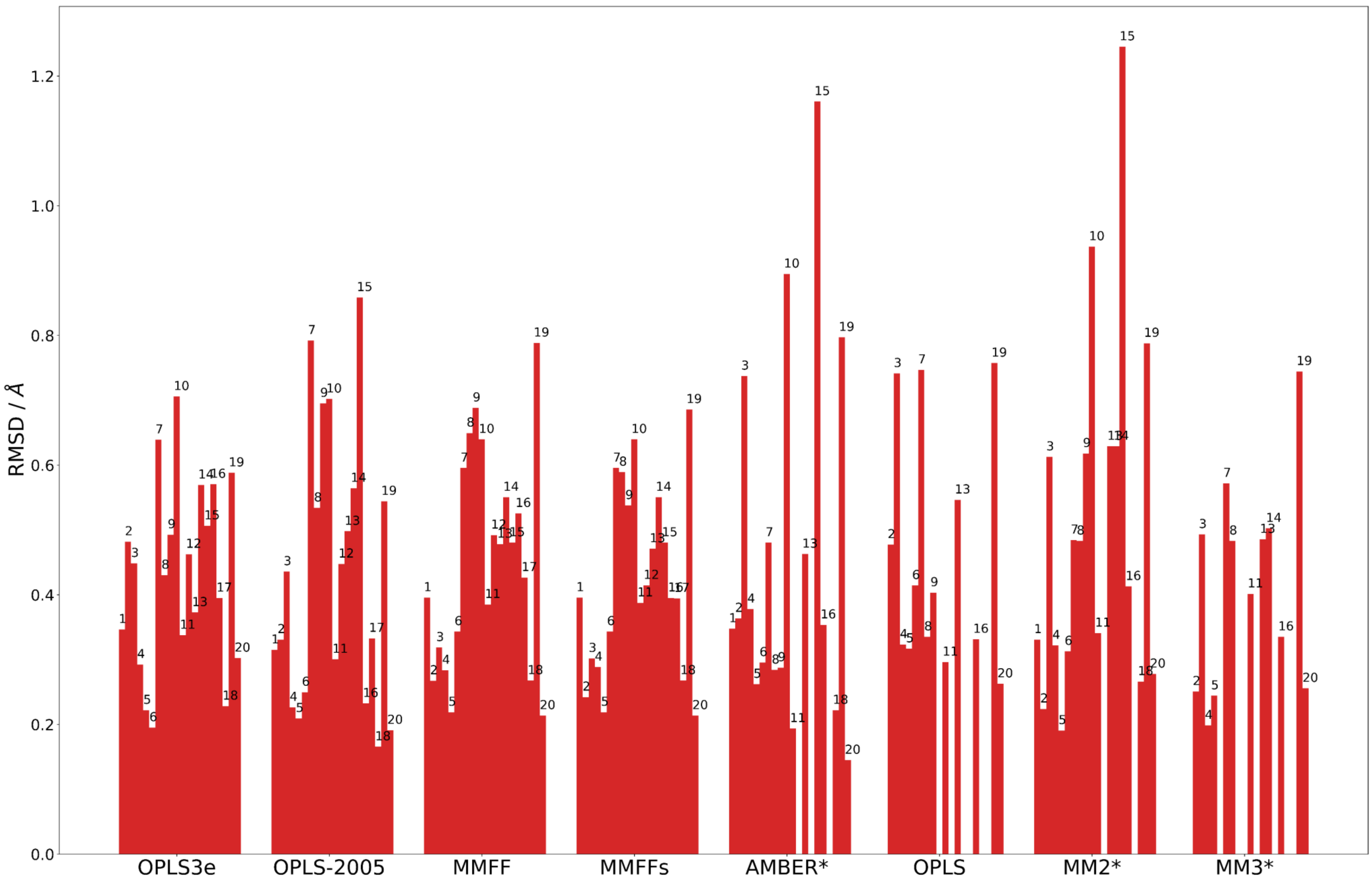

Figure S8. The RMSDs between the force field and DFT conformer geometries for each molecule individually.

7. Low Energy Conformers

Table S10 shows the proportion of molecules for which each force field was able to correctly identify the lowest energy conformer, out of the total number of molecules on which it could perform conformational searches.

Table S10. The numbers of molecules for which each force field was able to correctly predict the lowest energy conformer as a fraction of the number of the molecules it was able to perform conformational searches on.

Force Field	Proportion of correctly predicted lowest conformers
OPLS3e	5/20 = 0.25
OPLS-2005	5/20 = 0.25
MMFF	7/20 = 0.35
MMFFs	4/20 = 0.2
AMBER*	5/17 = 0.29
OPLS	7/13 = 0.54
MM2*	6/18 = 0.33
MM3*	3/12 = 0.25

The numbers of conformers that were correctly predicted to be within 10 kJ mol^{-1} of the minimum energy conformer were also of interest, and the numbers of these conformers are shown in Table S11 and the proportions of correctly predicted low-energy conformers are shown in Table S12.

Table S11. The numbers of conformers of each molecule correctly predicted by the force field to be within 10 kJ mol^{-1} of the minimum energy conformer against the numbers of those conformers that were within 10 kJ mol^{-1} of the minimum energy conformer according to DFT. In the cells, 'x//y' is read as x conformers were predicted by the force field to have a relative energy under 10 kJ mol^{-1} of the minimum energy conformer, and y of those conformers were within 10 kJ mol^{-1} according to DFT. Empty cells indicate the force field was unable to perform a conformational search, or all of the conformers found with that force field were within 10 kJ mol^{-1} of the minimum.

Molecule Number	OPLS3e	OPLS-2005	MMFF	MMFFs	AMBER	OPLS	MM2*	MM3*
**1**			1//1	1//1	2//2			
**2**	7//7	8//8	6//6	6//6	8//8	8//8	8//8	8//8
**3**	6//4	10//4	2//1	2//2	8//6	3//3	6//3	7//2
**4**	4//4	5//5	2//2	2//2	5//5	1//1	5//3	4//3
**5**	4//4	2//2	2//2	2//2	1//1	1//1	3//3	5//5
**6**	14//7	8//8	6//6	6//6	10//8	4//4	6//5	
**7**	14//14	9//9	5//5	5//5	7//7	7//5	6//6	8//8
**8**	7//2	5//2	5//1	6//1	6//5	10//5	15//7	9//4
**9**	1//1	2//2	6//2	1//1	2//1	3//3	2//1	
**10**	14//3	1//0	3//2	3//2	1//0		12//4	
**11**	22//16	8//8	11//11	11//11	10//10	4//4	36//22	18//18
**12**	6//4	5//5	7//6	7//7				
**13**	20//13	8//6	31//18	12//11	36//28	13//13	18//5	43//20
**14**	10//10	6//6	6//6	6//6			10//8	12//6
**15**	5//4	10//5	9//6	9//6	20//10		5//5	
**16**	4//4	6//6	6//3	3//3	3//3	3//3	7//7	5//5
**17**	2//2	4//2	2//2	5//4				
**18**			2//2	2//2	3//3		9//7	
**19**	14//13	10//8	8//8	6//6	7//6	3//3	32//25	16//15
**20**	9//6	8//6	10//10	10//10	9//8	5//4	10//5	6//5

Table S12. Ratios of the numbers of conformers predicted to be within 10 kJ mol^{-1} over the actual number of those conformers within 10 kJ mol^{-1} of the minimum, from the values in Table S11. Empty cells indicate the force field was unable to perform a conformational search, or all of the conformers of the molecule found with that force field were within 10 kJ mol^{-1} of the minimum.

Molecule Number	OPLS3e	OPLS-2005	MMFF	MMFFs	AMBER	OPLS	MM2*	MM3*
**1**			1	1	1			
**2**	1	1	1	1	1	1	1	1
**3**	0.6667	0.4	0.5	1	0.75	1	0.5	0.2857
**4**	1	1	1	1	1	1	0.6	0.75
**5**	1	1	1	1	1	1	1	1
**6**	0.5	1	1	1	0.8	1	0.8333	
**7**	1	1	1	1	1	0.7143	1	1
**8**	0.2857	0.4	0.2	0.1667	0.8333	0.5	0.4667	0.4444
**9**	1	1	0.3333	1	0.5	1	0.5	
**10**	0.2143	0	0.6667	0.6667	0		0.3333	
**11**	0.7273	1	1	1	1	1	0.6111	1
**12**	0.6667	1	0.8571	1				
**13**	0.65	0.75	0.5806	0.9167	0.7778	1	0.2778	0.4651
**14**	1	1	1	1			0.8	0.5
**15**	0.8	0.5	0.6667	0.6667	0.5		1	
**16**	1	1	0.5	1	1	1	1	1
**17**	1	0.5	1	0.8				
**18**			1	1	1		0.7778	
**19**	0.9286	0.8	1	1	0.8571	1	0.7813	0.9375
**20**	0.6667	0.75	1	1	0.8889	0.8	0.5	0.8333

Table S13. The mean values of the proportions of correctly predicted low-energy conformers from Table S12, also plotted in Figure S9.

OPLS3e	0.7837
OPLS-2005	0.7833
MMFF	0.8152
MMFFs	0.9108
AMBER	0.8181
OPLS	0.9242
MM2*	0.7048
MM3*	0.768

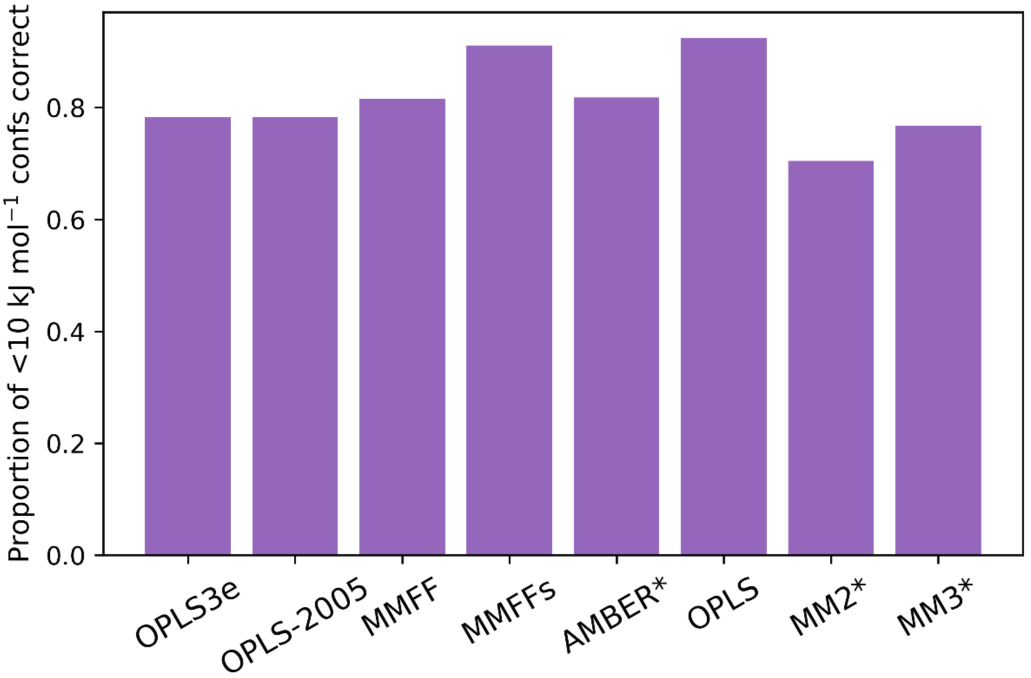

Figure S9. The mean values of the proportions of conformers that were correctly predicted by the force field to be within 10 kJ mol^{-1} of the minimum energy conformer according to DFT.

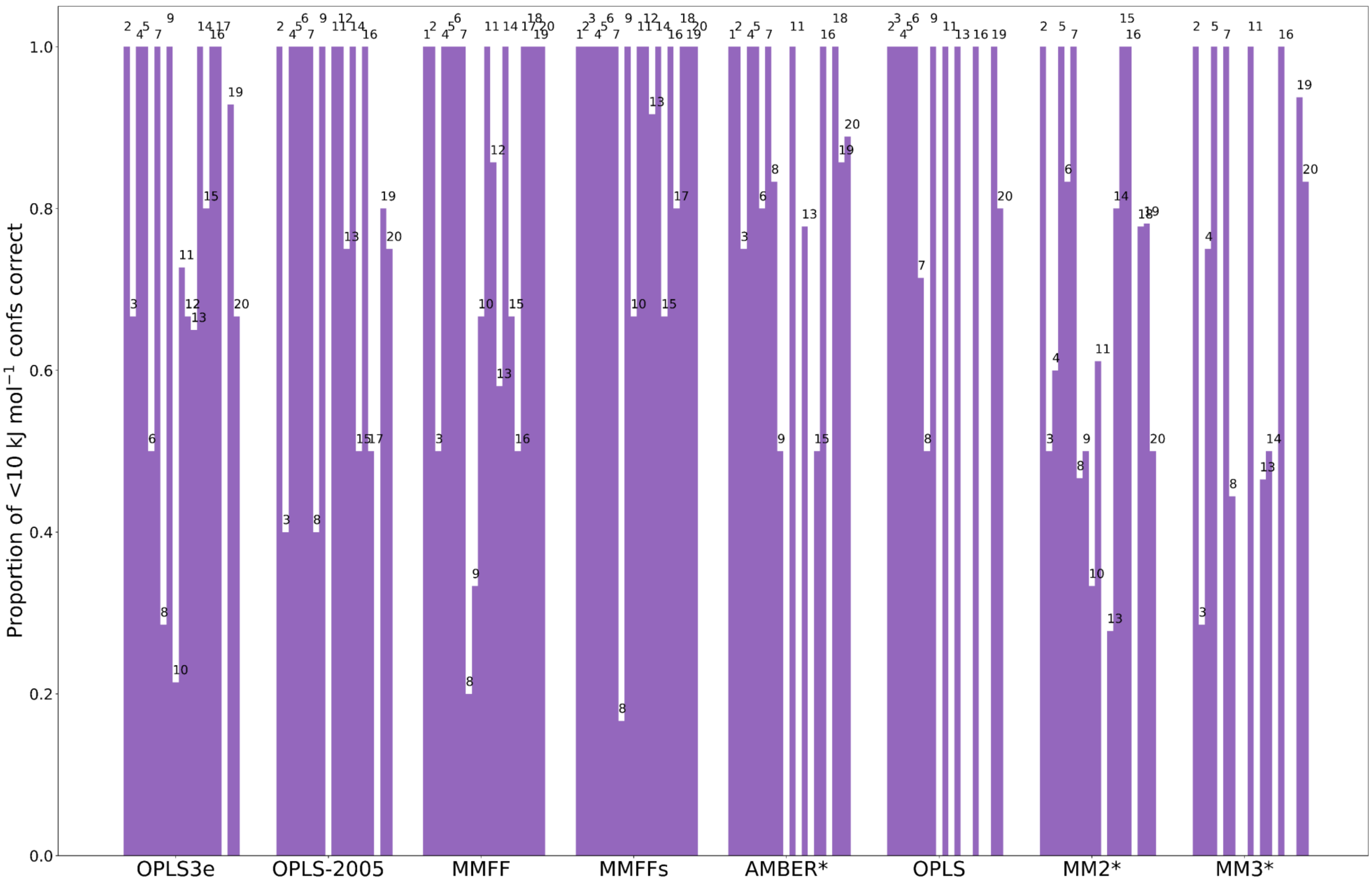

Figure S10. The proportions of conformers that were correctly predicted to be within 10 kJ mol-1 of the DFT minimum energy conformer for each molecule individually.

8. Conformers Remaining After Optimization/Redundant Conformer Elimination

Table S14. The numbers of conformers optimized with DFT against the number of conformers remaining after redundant conformer elimination with RMSD cutoff set to 0.1 Å. Each cell containing 'x//y' indicates that there were x conformers before redundant conformer elimination and y conformers remaining after. For each molecule, the maximum number of conformers remaining found by any of the force fields is highlighted in bold. Empty cells indicate the force field was unable to perform a conformational search for that molecule.

Molecule Number	OPLS3e	OPLS-2005	MMFF	MMFFs	AMBER	OPLS	MM2*	MM3*
**1**	3//**1**	3//**1**	2//**1**	2//**1**	3//**1**		3//**1**	
**2**	24//24	28//20	29//29	30//29	36//**32**	24//20	28//28	20//20
**3**	117//114	132//126	63//63	78//77	284//**235**	47//44	211//201	136//126
**4**	11//11	13//12	8//8	8//8	12//11	6//6	18//**15**	12//12
**5**	8//8	7//7	7//7	7//7	6//6	5//5	12//**10**	7//7
**6**	54//**22**	48//16	14//7	14//7	24//9	25//9	43//17	
**7**	32//**22**	15//11	8//6	8//6	18//16	18//14	14//14	17//15
**8**	52//**43**	33//25	60//36	45//31	25//24	48//39	47//39	56//39
**9**	36//27	52//41	59//37	33//31	29//28	33//30	60//**49**	
**10**	115//**83**	13//11	28//26	28//26	29//24		126//77	
**11**	44//**36**	16//12	24//18	16//8	12//12	8//8	48//35	30//16
**12**	55//**48**	18//18	46//43	33//30				
**13**	81//67	40//39	46//46	25//25	104//**83**	26//22	28//27	46//46
**14**	27//**22**	20//14	9//5	9//5			16//12	21//17
**15**	56//52	25//24	44//43	44//43	78//**46**		22//21	
**16**	34//**26**	16//16	21//16	21//18	31//27	24//24	22//20	19//17
**17**	91//87	86//83	125//**122**	122//**122**				
**18**	5//4	4//2	3//2	3//2	4//1		12//**5**	
**19**	90//84	46//45	49//47	42//39	108//97	69//62	153//**117**	112//98
**20**	81//58	26//24	29//26	29//26	43//40	9//9	81//**67**	58//49

Table S15. The ratios between the numbers of conformers remaining after redundant conformer eliminations and the numbers of conformers before.

Molecule Number	OPLS3e	OPLS-2005	MMFF	MMFFs	AMBER	OPLS	MM2*	MM3*
**1**	0.3333	0.3333	0.5	0.5	0.3333		0.3333	
**2**	1	0.7143	1	0.9667	0.8889	0.8333	1	1
**3**	0.9744	0.9545	1	0.9872	0.8275	0.9362	0.9526	0.9265
**4**	1	0.9231	1	1	0.9167	1	0.8333	1
**5**	1	1	1	1	1	1	0.8333	1
**6**	0.4074	0.3333	0.5	0.5	0.375	0.36	0.3953	
**7**	0.6875	0.7333	0.75	0.75	0.8889	0.7778	1	0.8824
**8**	0.8269	0.7576	0.6	0.6889	0.96	0.8125	0.8298	0.6964
**9**	0.75	0.7885	0.6271	0.9394	0.9655	0.9091	0.8167	
**10**	0.7217	0.8462	0.9286	0.9286	0.8276		0.6111	
**11**	0.8182	0.75	0.75	0.5	1	1	0.7292	0.5333
**12**	0.8727	1	0.9348	0.9091				
**13**	0.8272	0.975	1	1	0.7981	0.8462	0.9643	1
**14**	0.8148	0.7	0.5556	0.5556			0.75	0.8095
**15**	0.9286	0.96	0.9773	0.9773	0.5897		0.9545	
**16**	0.7647	1	0.7619	0.8571	0.871	1	0.9091	0.8947
**17**	0.956	0.9651	0.976	1				
**18**	0.8	0.5	0.6667	0.6667	0.25		0.4167	
**19**	0.9333	0.9783	0.9592	0.9286	0.8981	0.8986	0.7647	0.875
**20**	0.716	0.9231	0.8966	0.8966	0.9302	1	0.8272	0.8448

Table S16. The mean values of the proportions of conformers remaining after redundant conformer elimination, from the values in Table S15, also plotted in Figure S11.

OPLS3e	0.8066
OPLS_2005	0.8068
MMFF	0.8192
MMFFs	0.8276
AMBER	0.7836
OPLS	0.8749
MM2	0.7734
MM3	0.8719

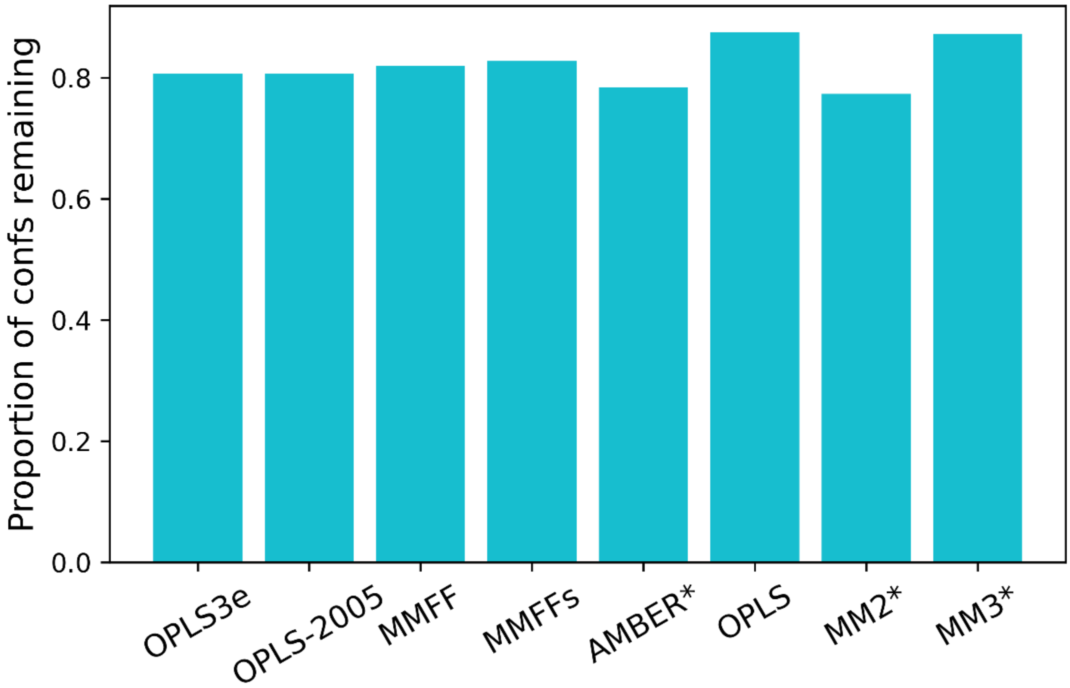

Figure S11. The mean values of the ratios between the number of conformers following redundant conformer elimination and the number of conformers from the force fields from all of the molecules.

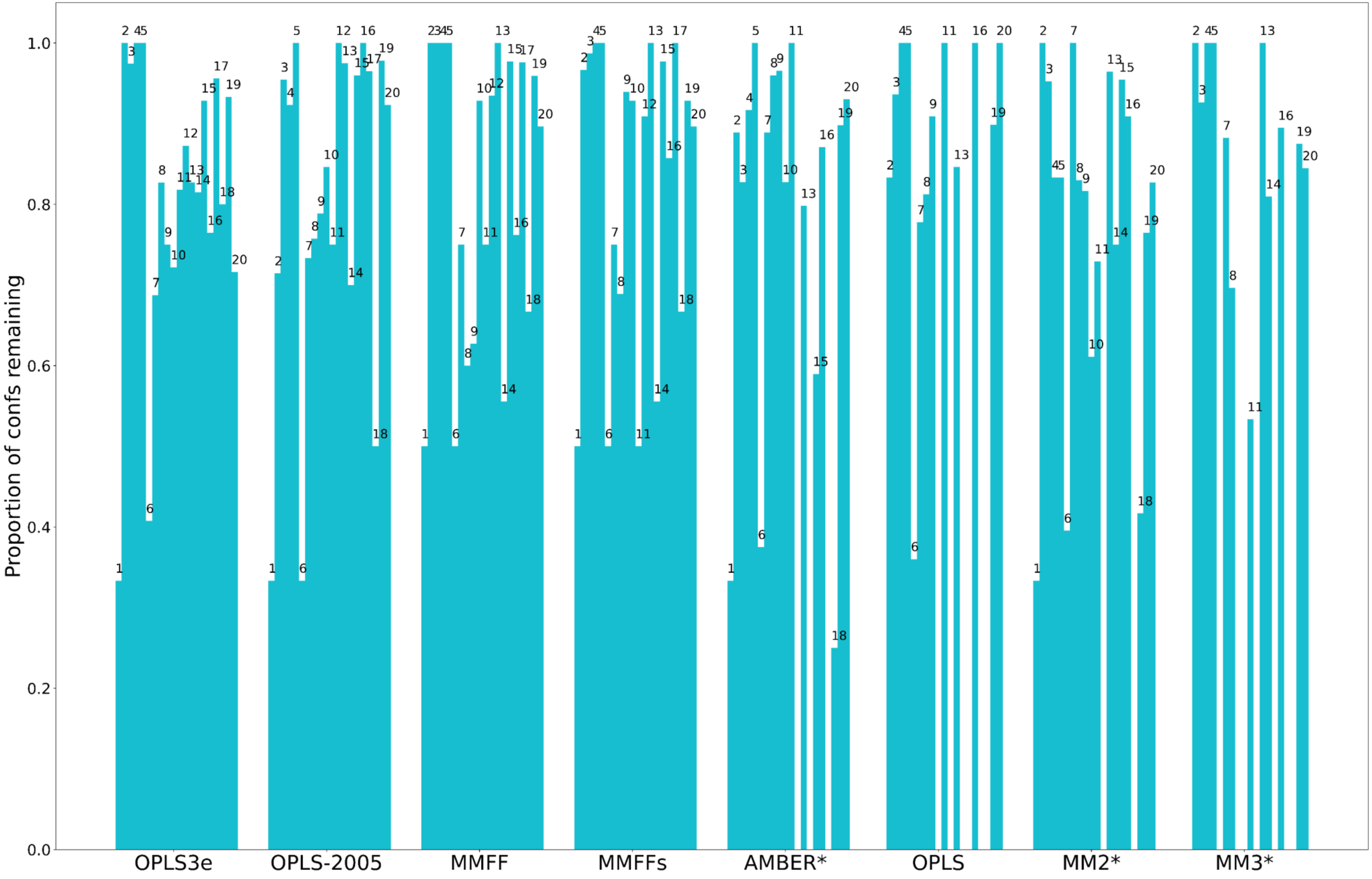

Figure S12. The ratios between the numbers of conformers remaining after the redundant conformer elimination and the numbers of conformers found by the force fields for each molecule individually.

9. Proportions of Maximum Numbers of Conformers Found

Table S17. The final numbers of conformers found by each force field for each molecule, divided by the maximum final number of conformers for any of the force fields for that molecule, from the data in Table S14.

Molecule Number	OPLS3e	OPLS-2005	MMFF	MMFFs	AMBER	OPLS	MM2*	MM3*
**1**	1	1	1	1	1		1	
**2**	0.75	0.625	0.9063	0.9063	1	0.625	0.875	0.625
**3**	0.4851	0.5362	0.2681	0.3277	1	0.1872	0.8553	0.5362
**4**	0.7333	0.8	0.5333	0.5333	0.7333	0.4	1	0.8
**5**	0.8	0.7	0.7	0.7	0.6	0.5	1	0.7
**6**	1	0.7273	0.3182	0.3182	0.4091	0.4091	0.7727	
**7**	1	0.5	0.2727	0.2727	0.7273	0.6364	0.6364	0.6818
**8**	1	0.5814	0.8372	0.7209	0.5581	0.907	0.907	0.907
**9**	0.551	0.8367	0.7551	0.6327	0.5714	0.6122	1	
**10**	1	0.1325	0.3133	0.3133	0.2892		0.9277	
**11**	1	0.3333	0.5	0.2222	0.3333	0.2222	0.9722	0.4444
**12**	1	0.375	0.8958	0.625				
**13**	0.8072	0.4699	0.5542	0.3012	1	0.2651	0.3253	0.5542
**14**	1	0.6364	0.2273	0.2273			0.5455	0.7727
**15**	1	0.4615	0.8269	0.8269	0.8846		0.4038	
**16**	0.963	0.5926	0.5926	0.6667	1	0.8889	0.7407	0.6296
**17**	0.7131	0.6803	1	1				
**18**	0.8	0.4	0.4	0.4	0.2		1	
**19**	0.7179	0.3846	0.4017	0.3333	0.8291	0.5299	1	0.8376
**20**	0.8657	0.3582	0.3881	0.3881	0.597	0.1343	1	0.7313

Table S18. The mean values of the proportions of the maximum number of conformers found by each force field, from the values in Table S17, also plotted in Figure S13.

OPLS3e	0.8593
OPLS_2005	0.5565
MMFF	0.5845
MMFFs	0.5358
AMBER	0.6901
OPLS	0.4859
MM2	0.8312
MM3	0.685

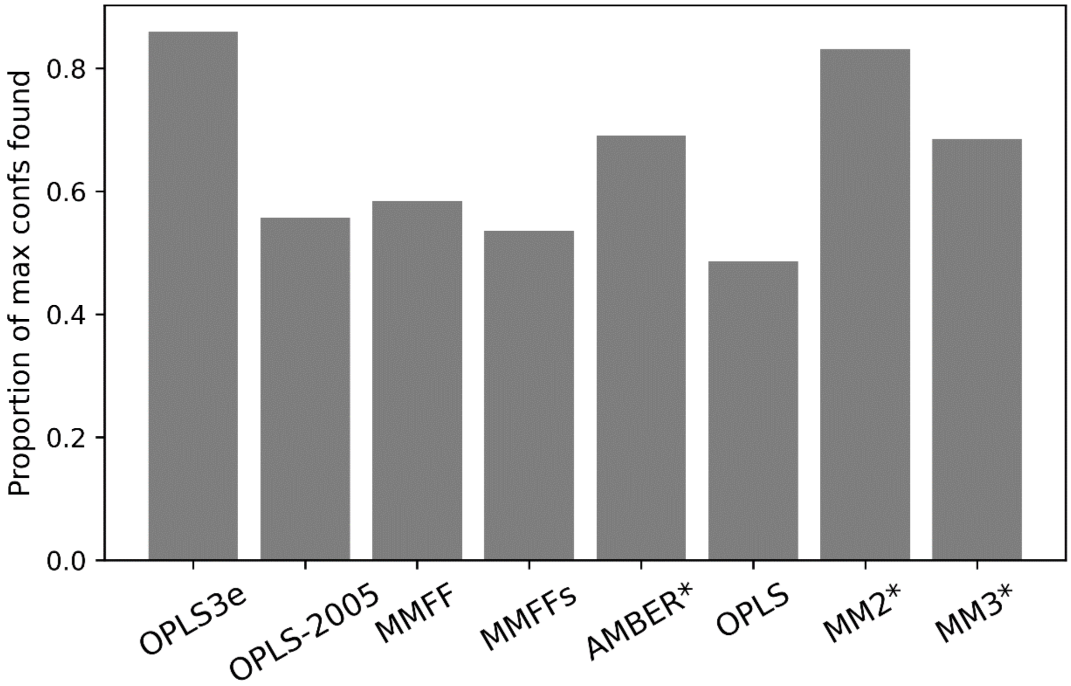

Figure S13. The mean values of the ratios between the final number of conformers for each molecule from each force field and the maximum number of conformers found by any force field for that molecule.

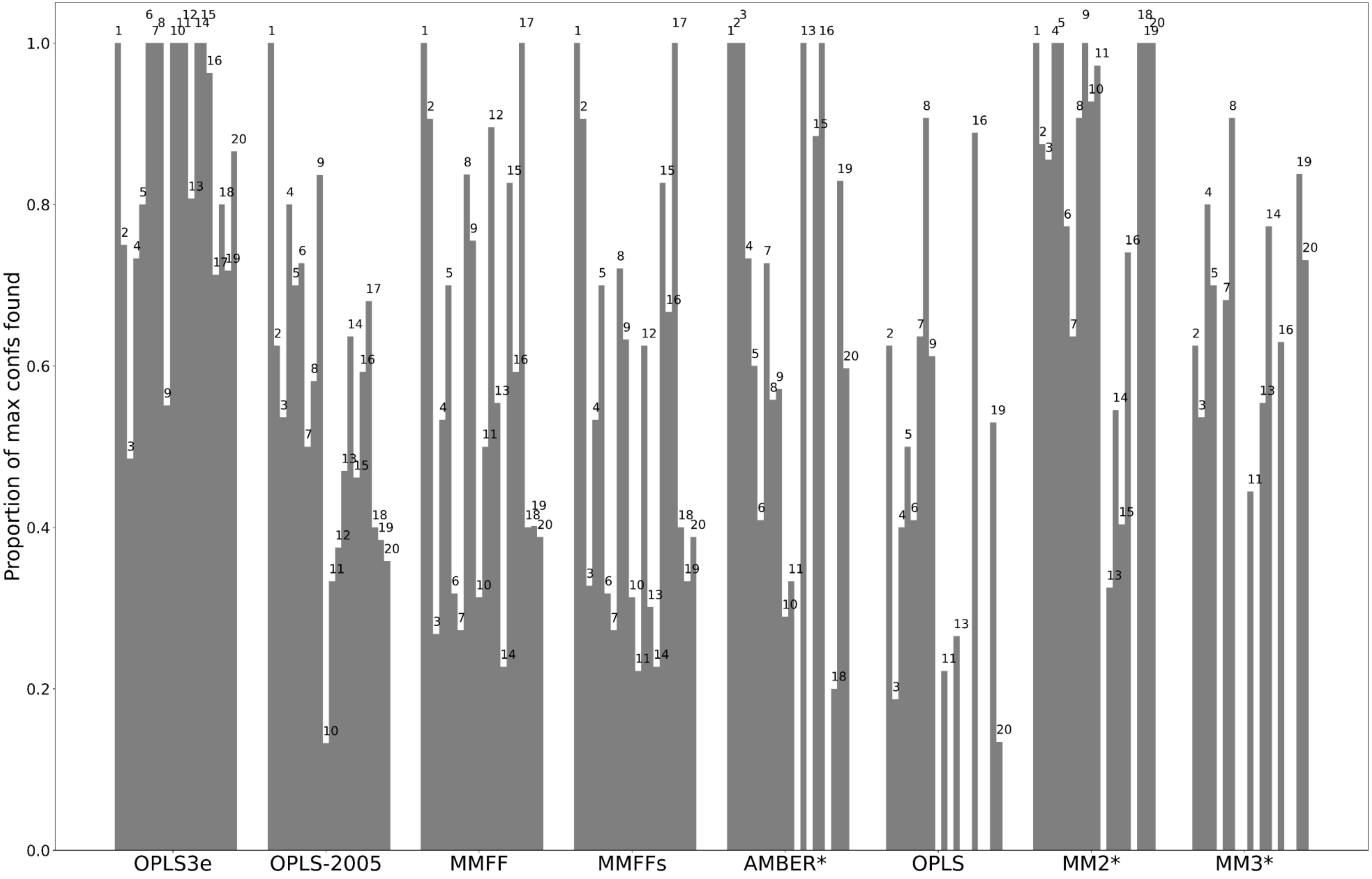

Figure S14. The ratios between the final number of conformers for each molecule from each force field and the maximum number of conformers found by any force field for that molecule, for each molecule individually.

10. Conformer Electronic Interactions

Molecule 3

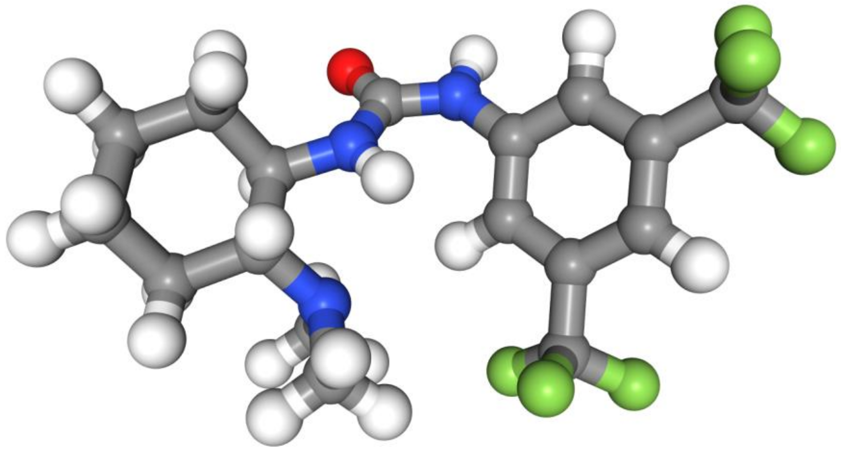

Figure S15. The lowest energy conformer of molecule 3 from the OPLS-2005 force field.

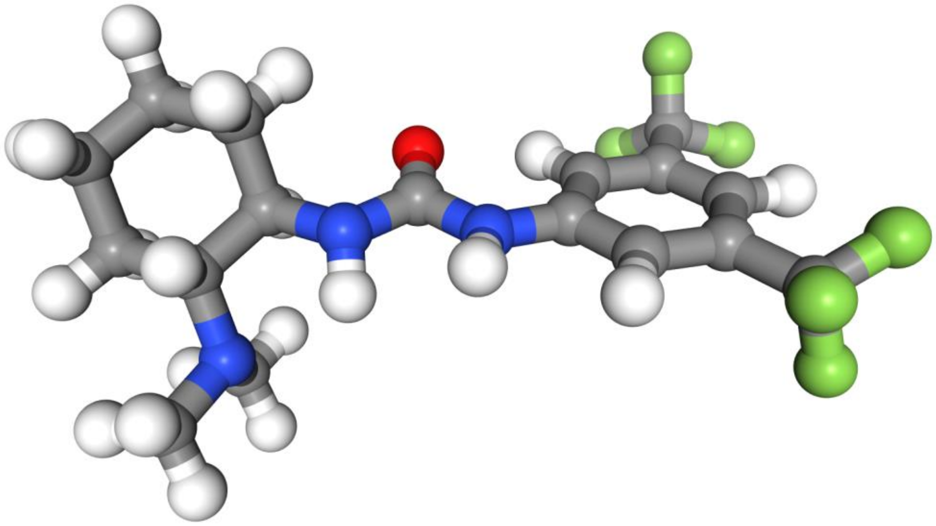

Figure S16. The lowest energy conformer of molecule 3 from the MMFF force field.

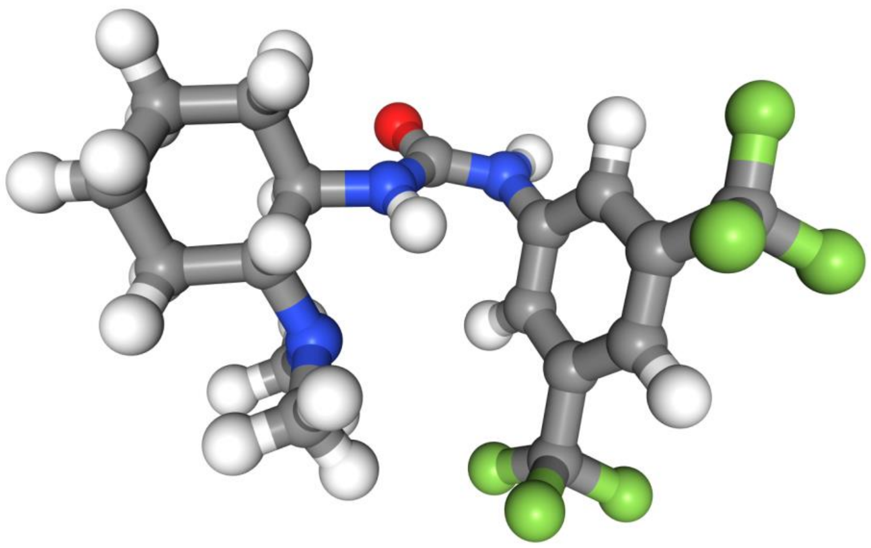

Figure S17. The lowest energy conformer of molecule 3 from the AMBER* force field.

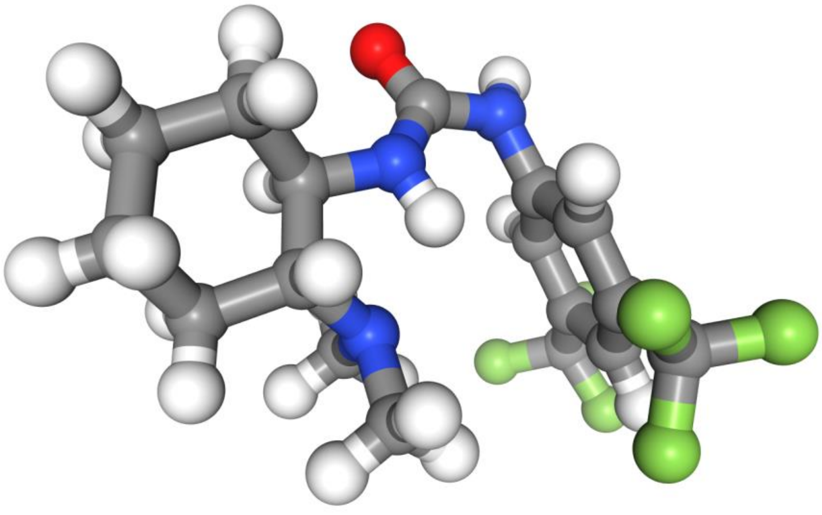

Figure S18. The lowest energy conformer of molecule 3 from the OPLS force field.

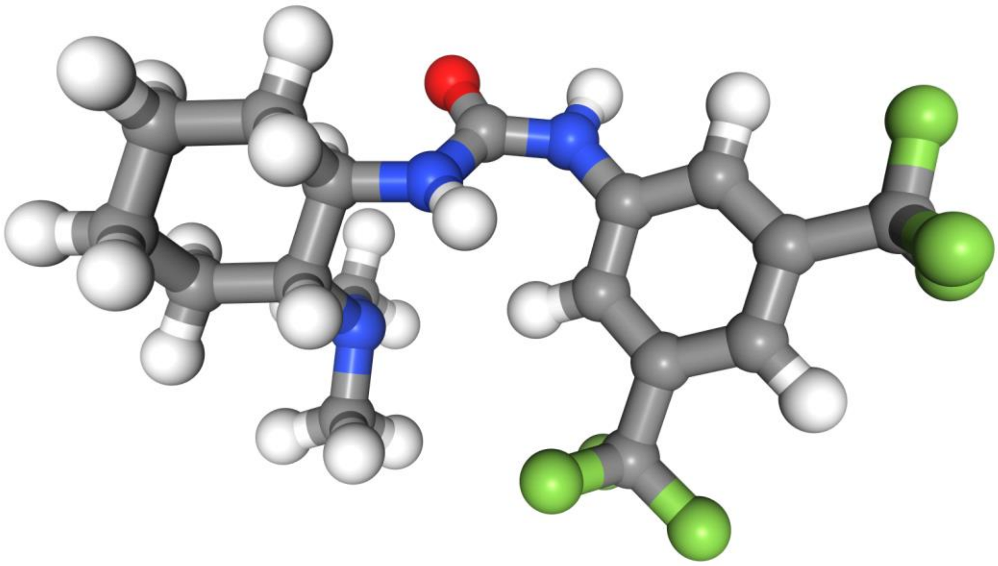

Figure S19. The lowest energy conformer of molecule 3 from the MM2* force field.

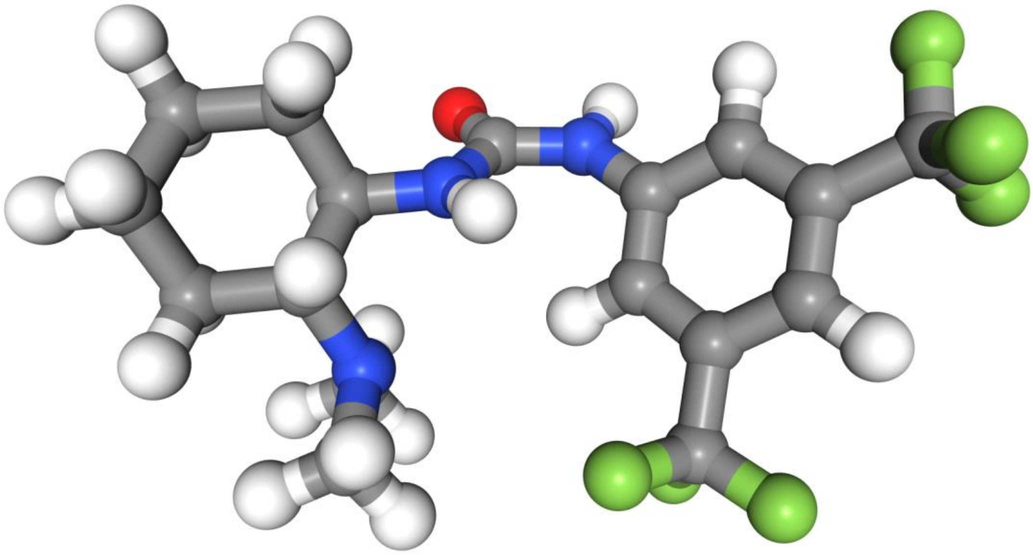

Figure S20. The lowest energy conformer of molecule 3 from the MM3* force field.

Molecule 5

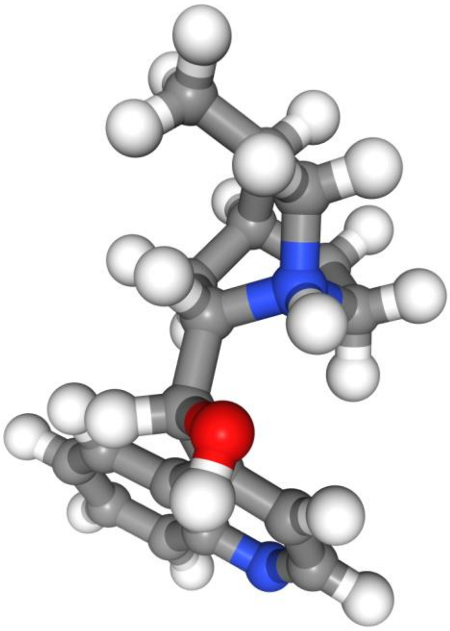

Figure S21. The lowest energy conformer of molecule 5 from the OPLS-2005 force field.

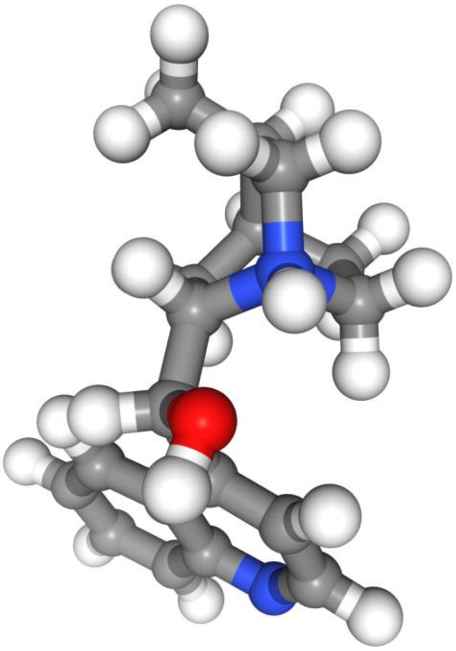

Figure S22. The lowest energy conformer of molecule 5 from the MMFF force field.

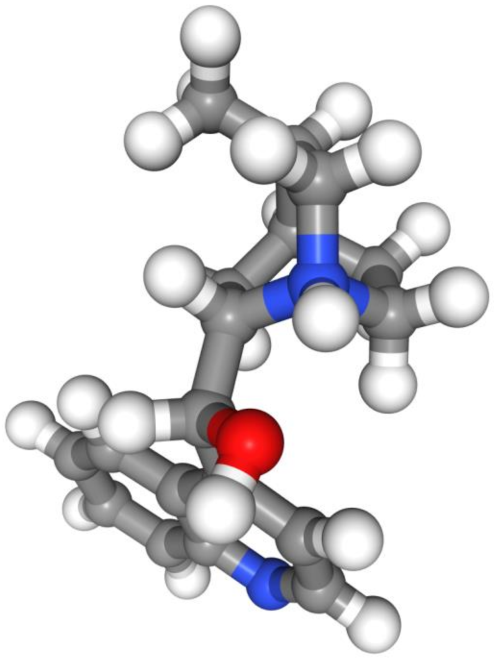

Figure S23. The lowest energy conformer of molecule 5 from the AMBER* force field.

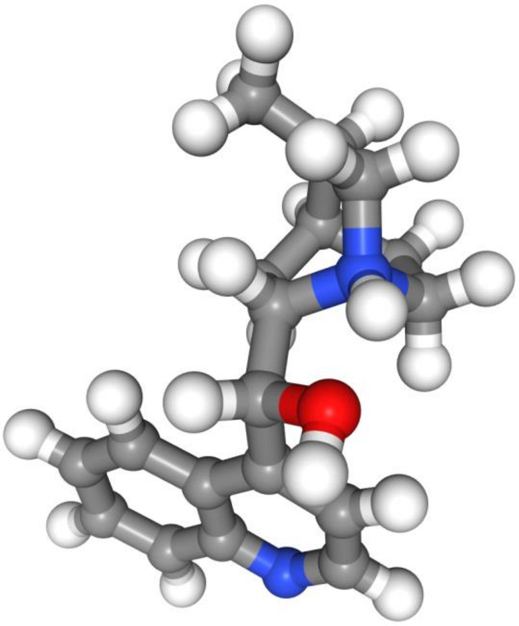

Figure S24. The lowest energy conformer of molecule 5 from the OPLS force field.

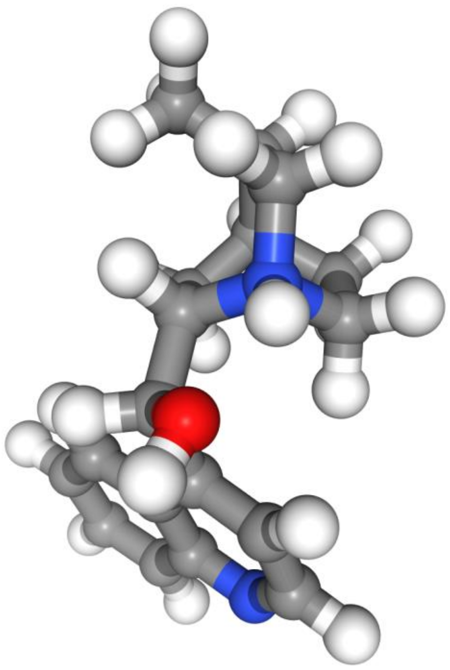

Figure S25. The lowest energy conformer of molecule 5 from the MM2* force field.

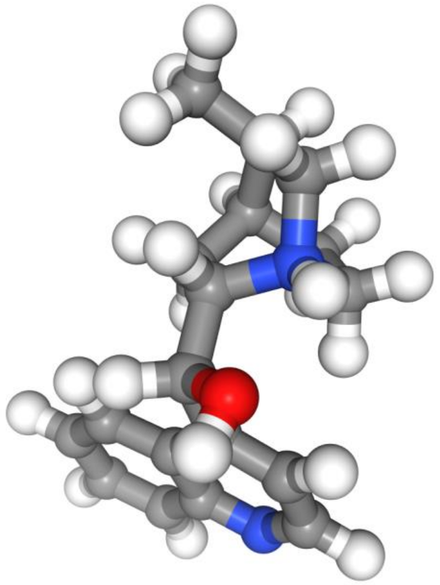

Figure S26. The lowest energy conformer of molecule 5 from the MM3* force field.

Molecule 7

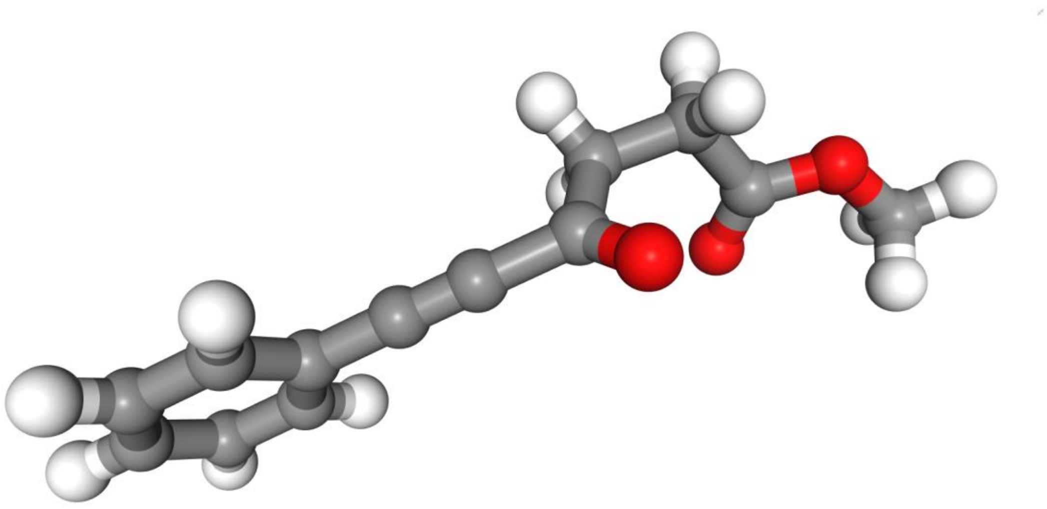

Figure S27. The lowest energy conformer of molecule 7 from the OPLS3e force field.

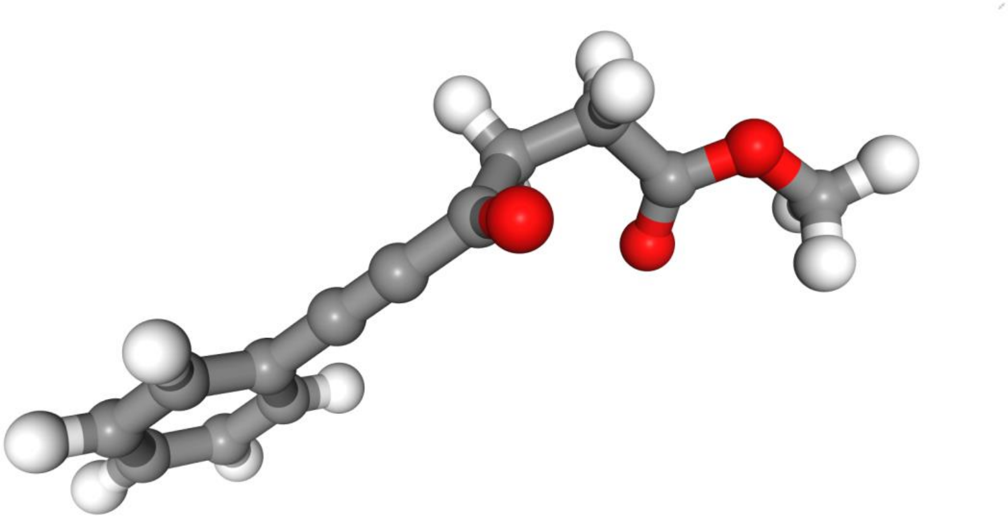

Figure S28. The lowest energy conformer of molecule 7 from the OPLS-2005 force field.

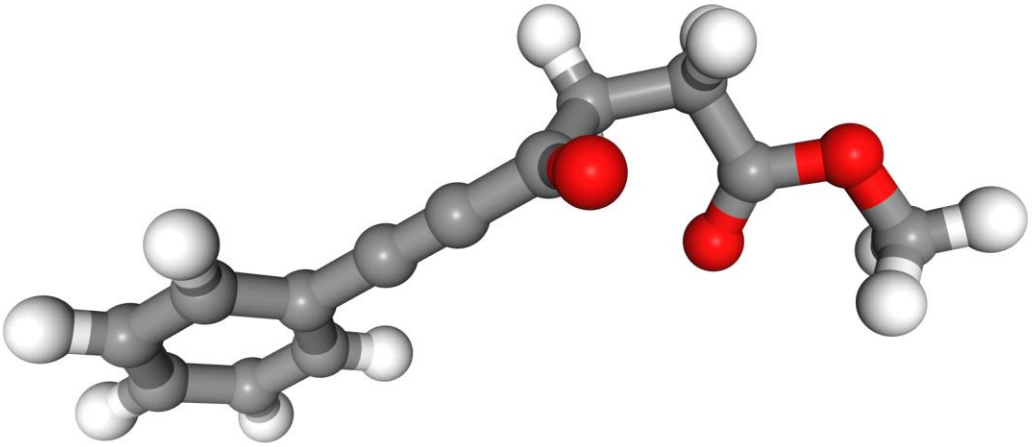

Figure S29. The lowest energy conformer of molecule 7 from the MMFF force field.

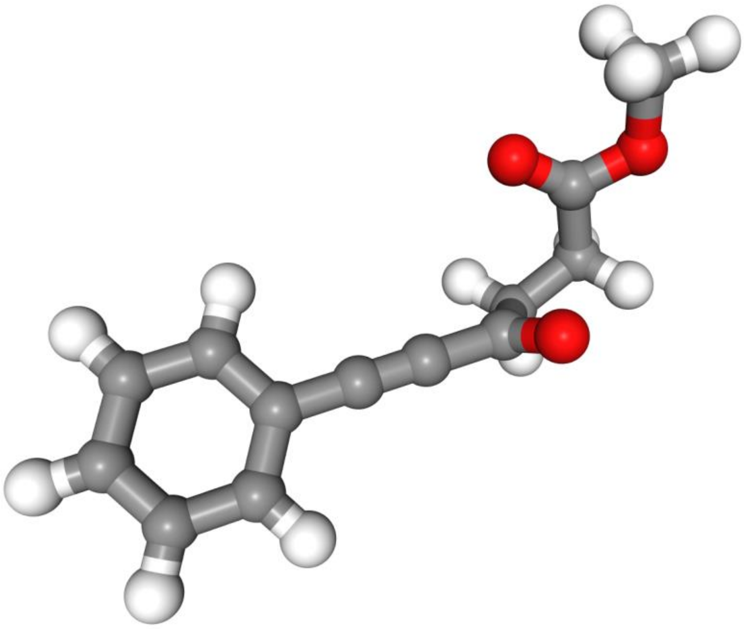

Figure S30. The lowest energy conformer of molecule 7 from the AMBER* force field.

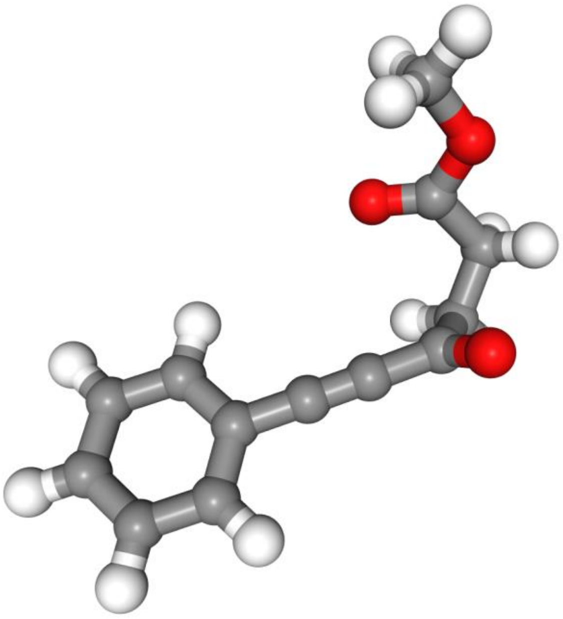

Figure S31. The lowest energy conformer of molecule 7 from the OPLS force field.

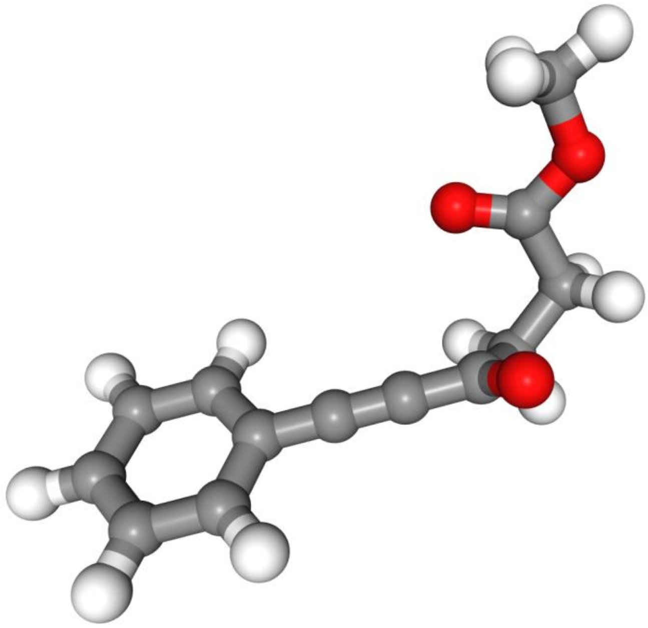

Figure S32. The lowest energy conformer of molecule 7 from the MM2* force field.

Molecule 8

The lowest conformer of molecule 8 at the DFT level contains a hydrogen bond, as seen in Figure S33. OPLS3e, MMFF, MMFFs and AMBER* find this hydrogen bond in their lowest energy conformers, but of these four force fields only AMBER* (Figure S38) closely reproduces the actual lowest energy conformer from DFT (the lowest energy conformers from the other three force fields have substantially different geometries other than the hydrogen bond

and are shown in Figures S34, S36 and S37). On the other hand, the force fields OPLS-2005 (Figure S35), OPLS and MM2* (Figures S39 and S40) all seem to find a π-π stacking interaction between the two aromatic rings to be more stabilising. MM3* (Figure S41) does not seem to find any stabilising electronic interaction at all.

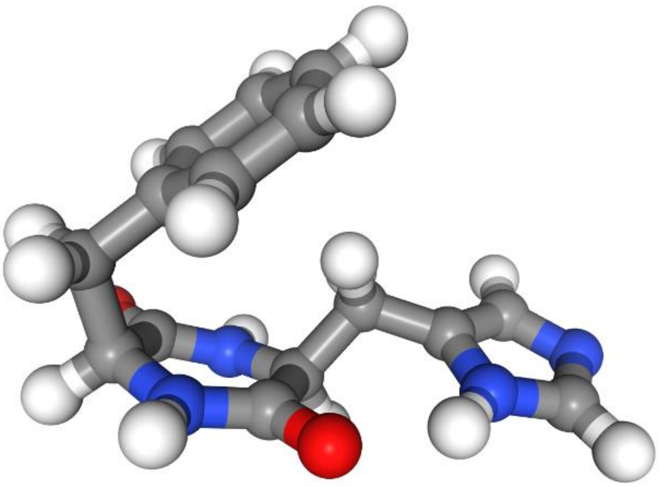

Figure S33. The lowest energy conformer of molecule 8 from DFT optimisation.

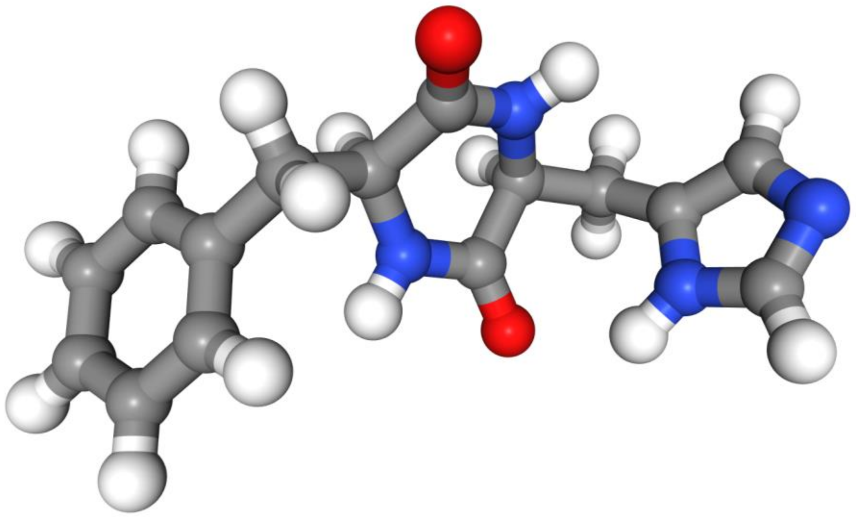

Figure S34. The lowest energy conformer of molecule 8 from the OPLS3e force field.

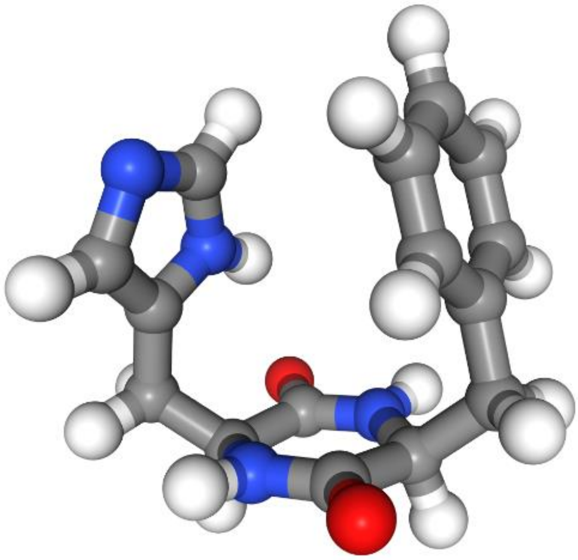

Figure S35. The lowest energy conformer of molecule 8 from the OPLS-2005 force field.

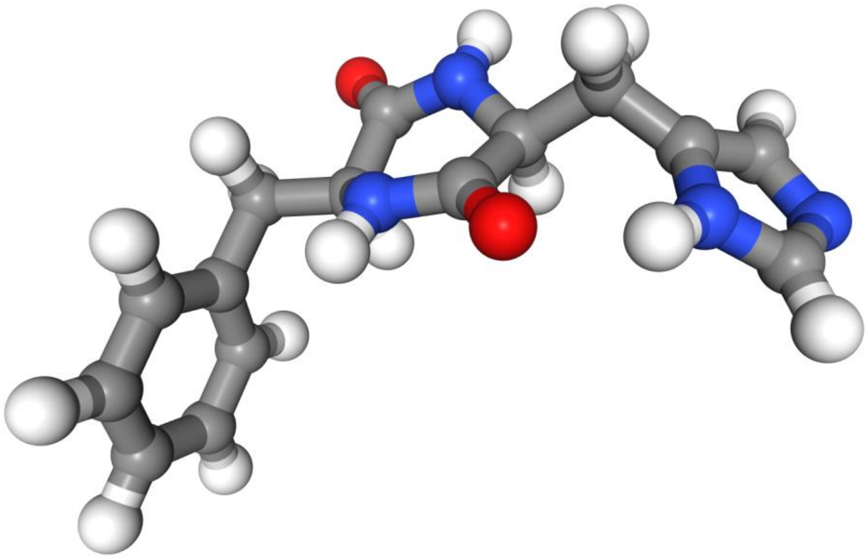

Figure S36. The lowest energy conformer of molecule 8 from the MMFF force field.

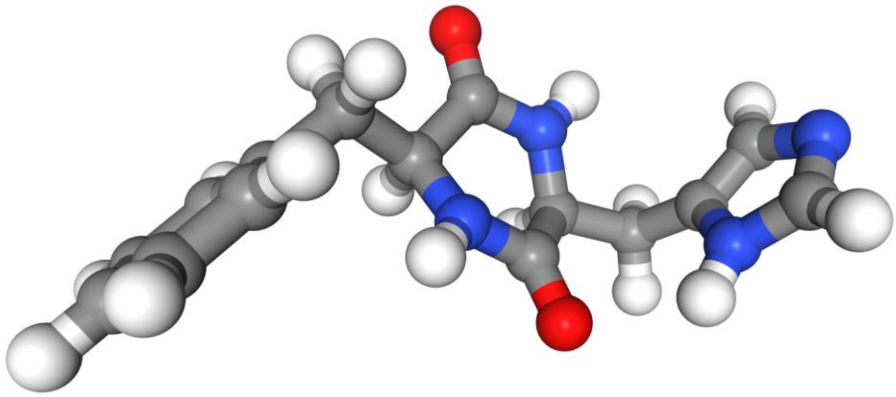

Figure S37. The lowest energy conformer of molecule 8 from the MMFFs force field.

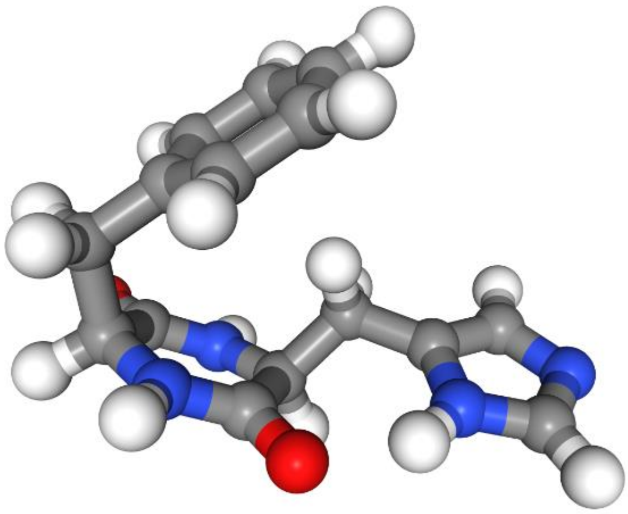

Figure S38. The lowest energy conformer of molecule 8 from the AMBER* force field.

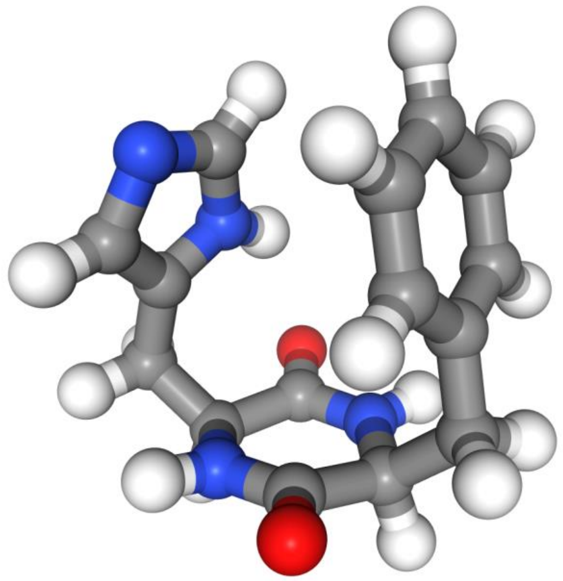

Figure S39. The lowest energy conformer of molecule 8 from the OPLS force field.

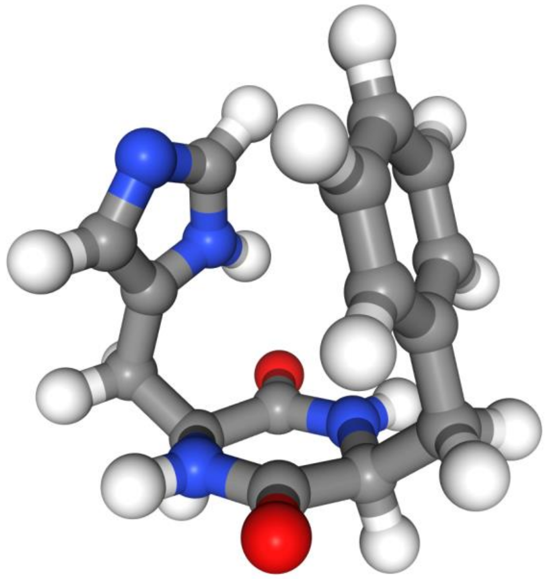

Figure S40. The lowest energy conformer of molecule 8 from the MM2* force field.

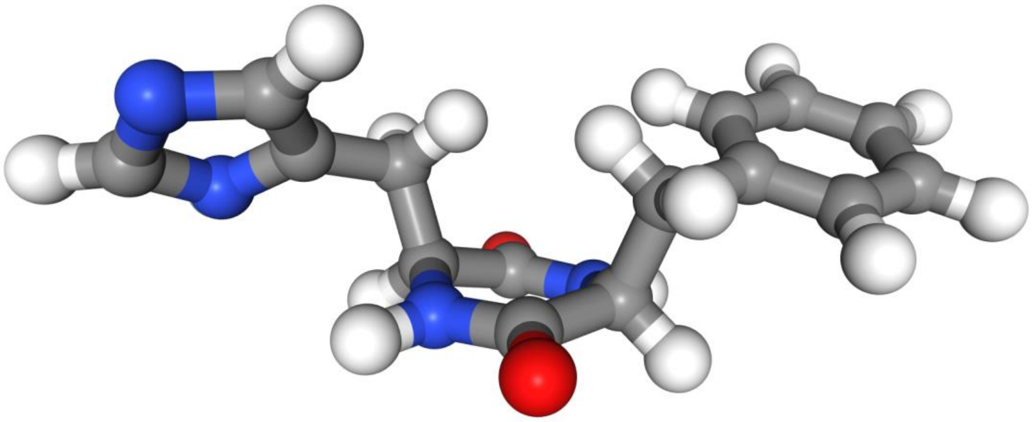

Figure S41. The lowest energy conformer of molecule 8 from the MM3* force field.

Molecule 10

Molecule 10 presents an interesting case, the lowest energy conformer from DFT contains a single hydrogen bond (Figure S42), however none of the force fields were able to predict this configuration was the most stable. OPLS-2005 (Figure S44), MMFF and AMBER* (Figures S45 and S46) have two hydrogen bonds, however the low energy conformers of OPLS3e and MM2* (Figures S43 and S47) have no hydrogen bonds. It would therefore seem that none of the force fields are able to correctly balance the energetic trade-offs between the stabilising interactions from hydrogen bonding and the destabilising interactions of the two triflyl groups.

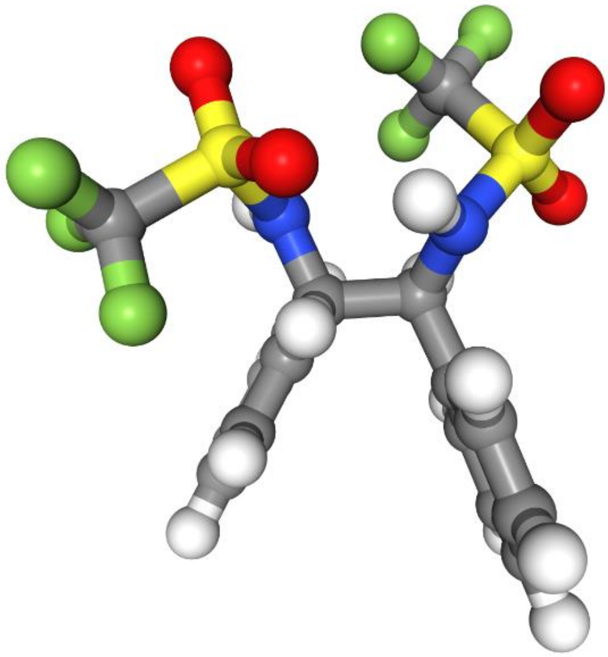

Figure S42. The lowest energy conformer of molecule 10 from DFT optimisation.

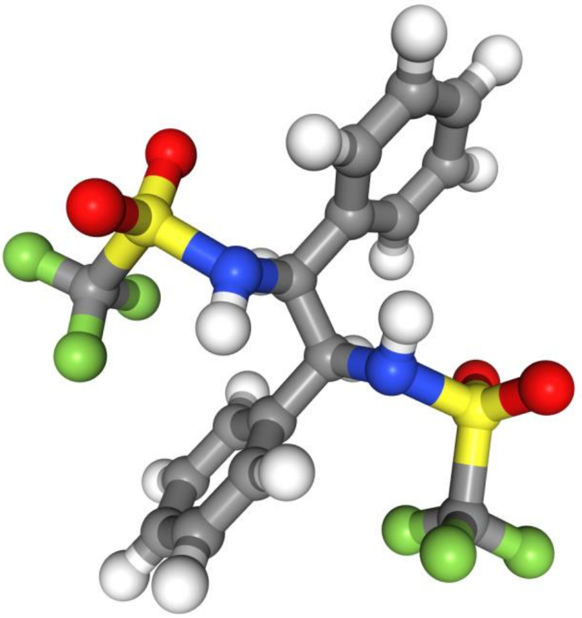

Figure S43. The lowest energy conformer of molecule 10 from the OPLS3e force field.

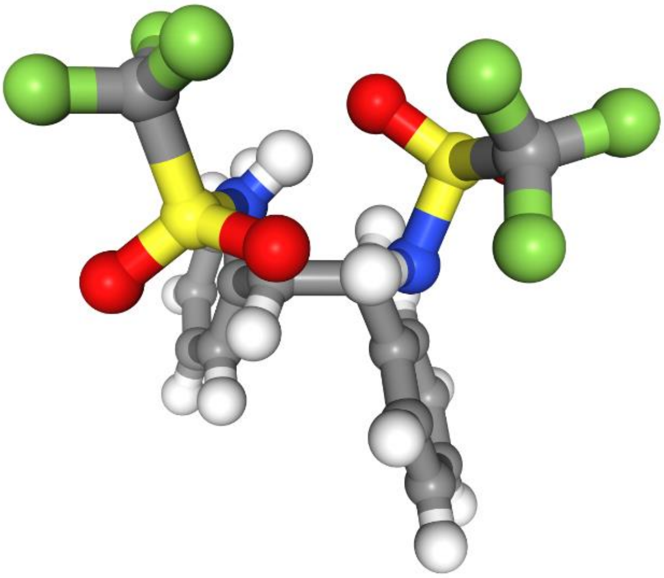

Figure S44. The lowest energy conformer of molecule 10 from the OPLS-2005 force field.

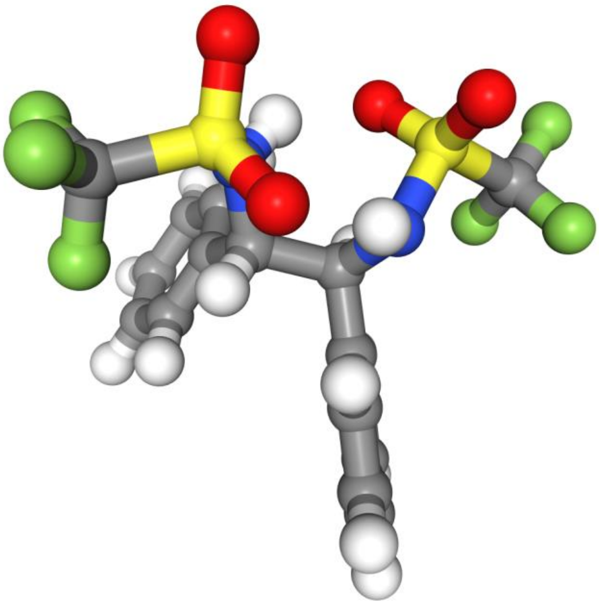

Figure S45. The lowest energy conformer of molecule 10 from the MMFF force field.

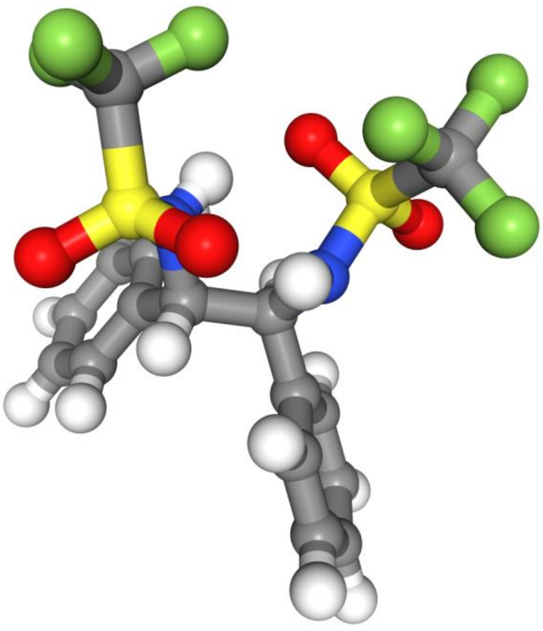

Figure S46. The lowest energy conformer of molecule 10 from the AMBER* force field.

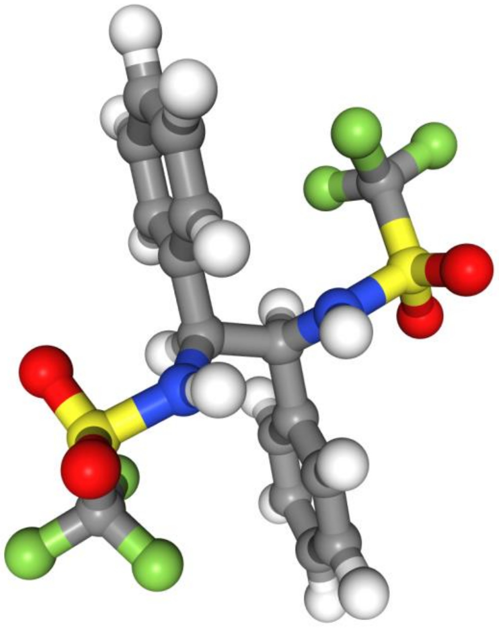

Figure S47. The lowest energy conformer of molecule 10 from the MM2* force field.

Molecule 19

All of the force fields predict the presence of the hydrogen bond in molecule 19 between the protonated amine and the carbonyl oxygen of the urea group that is present in the DFT lowest energy conformer (Figure S48), but the arrangement of the aryl groups varies substantially between force fields, with OPLS3e and MM3* being closest to DFT (Figures S49 and S56). In contrast to molecule 3, where the stabilisation of the conjugative interaction between the

trifluoromethylated phenyl ring and the urea group was correctly predicted to be the most stabilising interaction in the molecule, in the case of molecule 19 MMFF and MMFFs also have the same conjugative interaction (see Figures S51 and S52), but this time the stabilisation of this interaction seems to be overestimated by these force fields.

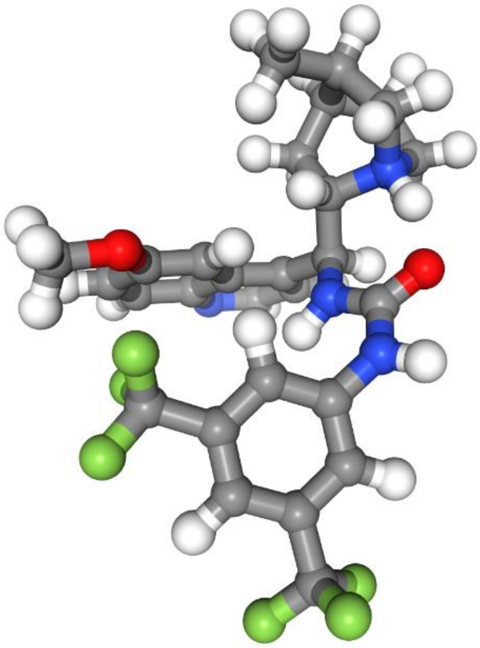

Figure S48. The lowest energy conformer of molecule 19 from DFT optimisation.

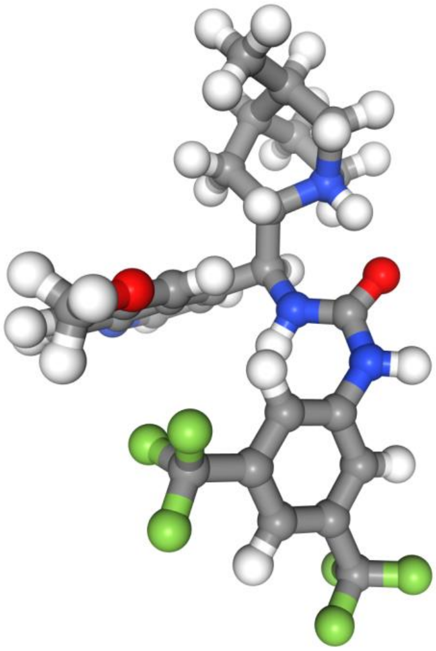

Figure S49. The lowest energy conformer of molecule 19 from the OPLS3e force field.

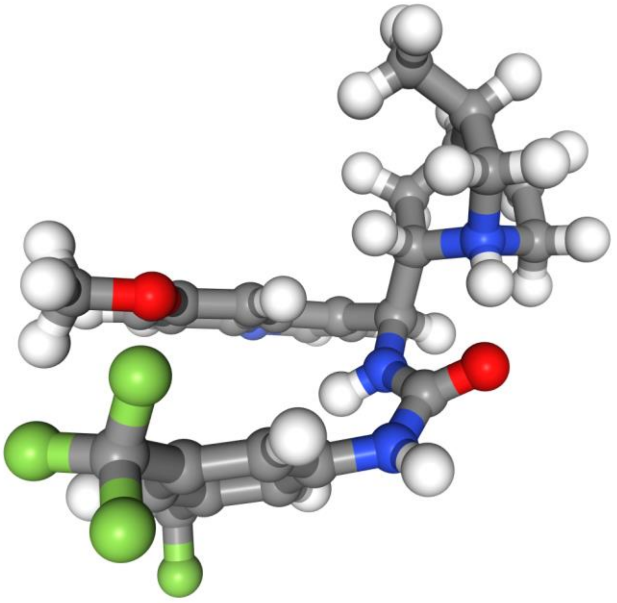

Figure S50. The lowest energy conformer of molecule 19 from the OPLS-2005 force field.

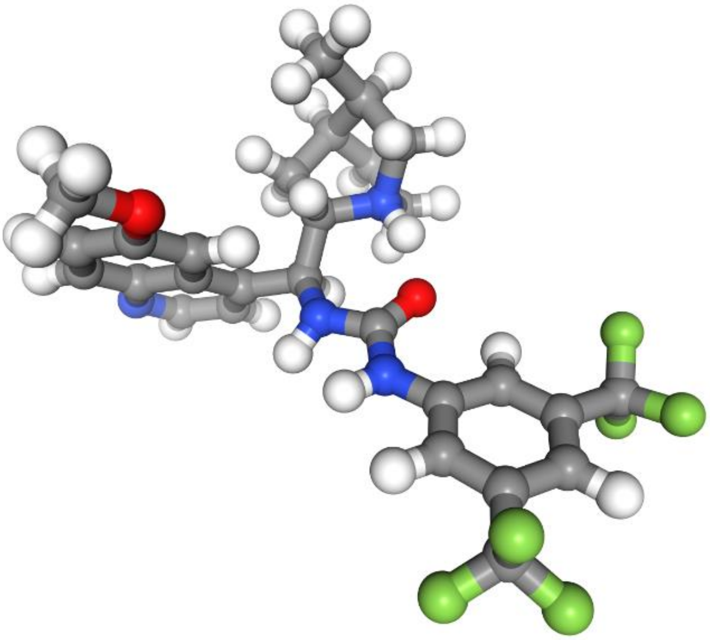

Figure S51. The lowest energy conformer of molecule 19 from the MMFF force field.

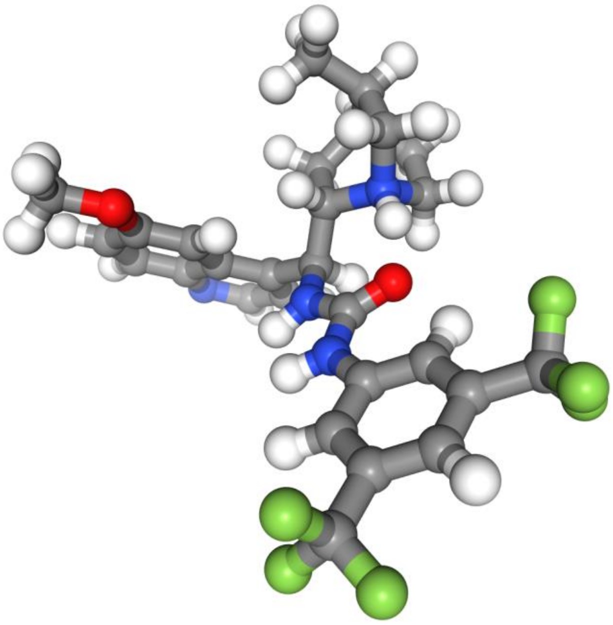

Figure S52. The lowest energy conformer of molecule 19 from the MMFFs force field.

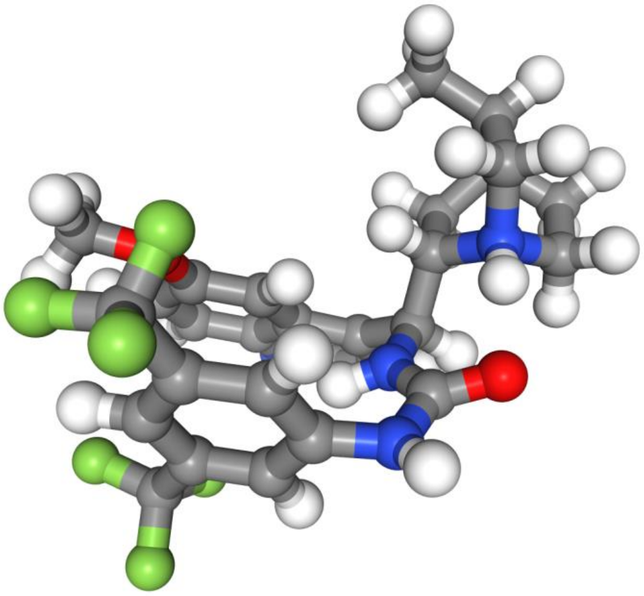

Figure S53. The lowest energy conformer of molecule 19 from the AMBER* force field.

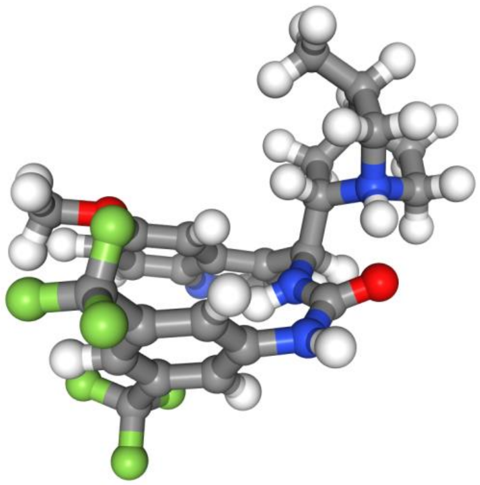

Figure S54. The lowest energy conformer of molecule 19 from the OPLS force field.

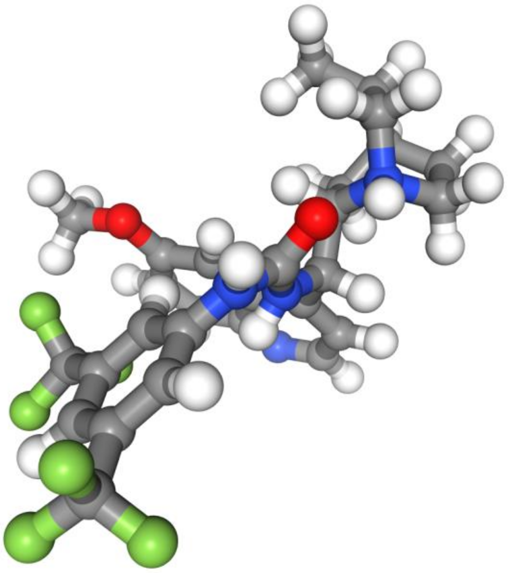

Figure S55. The lowest energy conformer of molecule 19 from the MM2* force field.

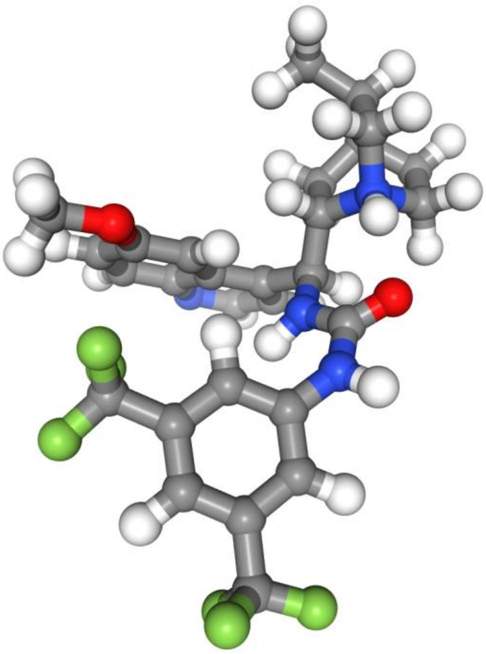

Figure S56. The lowest energy conformer of molecule 19 from the MM3* force field.

Molecule 20

Finally, as seen in Figure S57, the lowest energy conformer of molecule 20 has a hydrogen bond between the protonated amine and secondary amine groups of the two five-membered rings. All of the force fields (Figures S58-62) also have this hydrogen bond within their lowest energy conformers, except for MM2* and MM3* (Figures S63 and S64). These force fields seem to consider the energy penalty for bending the bond angle at the carbon atom that connects the two five-membered rings to be of greater magnitude than the stabilisation gained from the hydrogen bond.

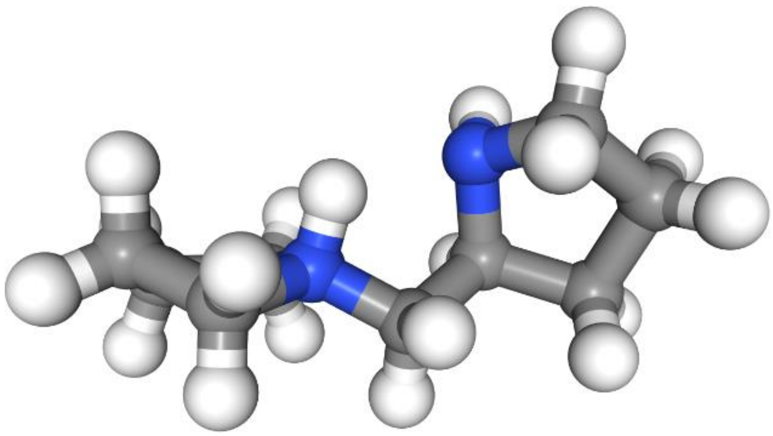

Figure S57. The lowest energy conformer of molecule 20 from DFT optimisation.

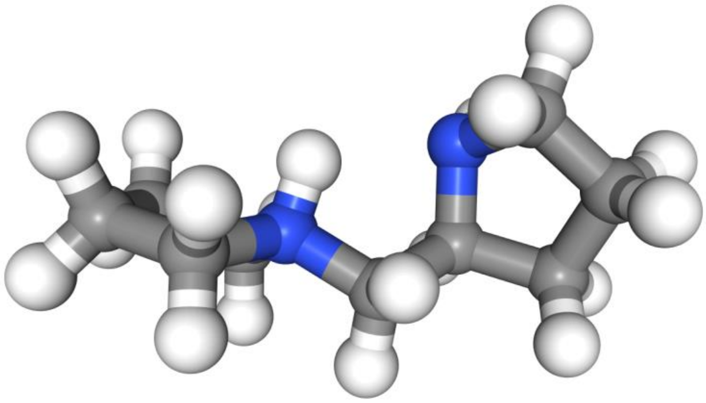

Figure S58. The lowest energy conformer of molecule 20 from the OPLS3e force field.

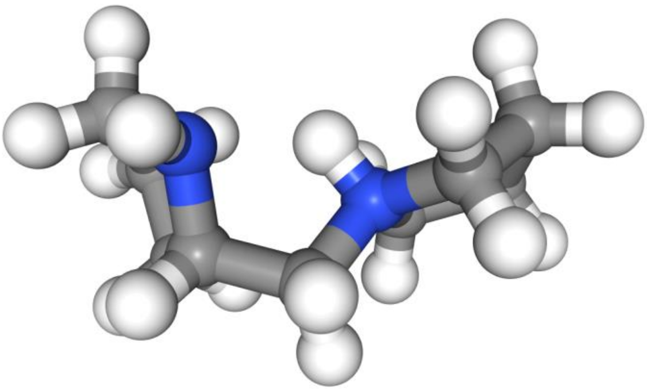

Figure S59. The lowest energy conformer of molecule 20 from the OPLS-2005 force field.

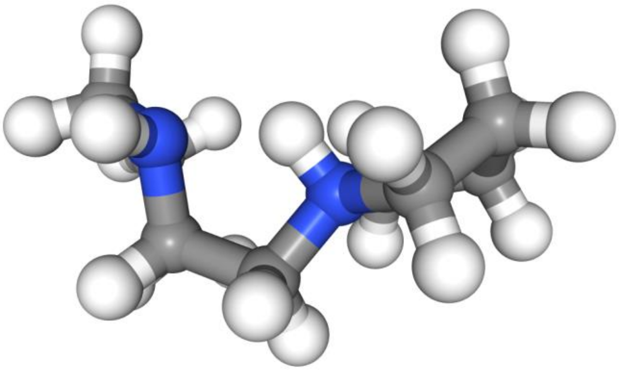

Figure S60. The lowest energy conformer of molecule 20 from the MMFF force field.

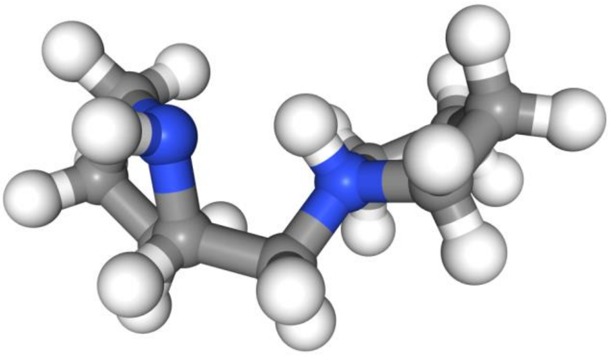

Figure S61. The lowest energy conformer of molecule 20 from the AMBER* force field.

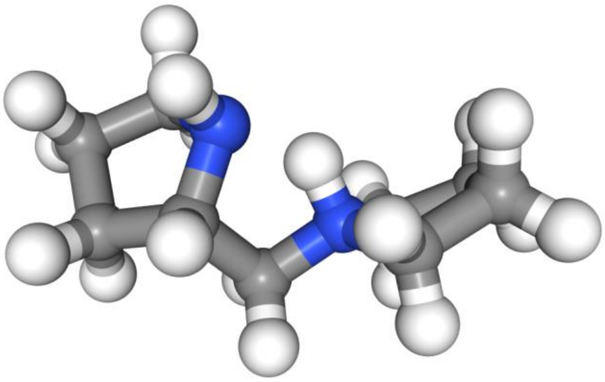

Figure S62. The lowest energy conformer of molecule 20 from the OPLS force field.

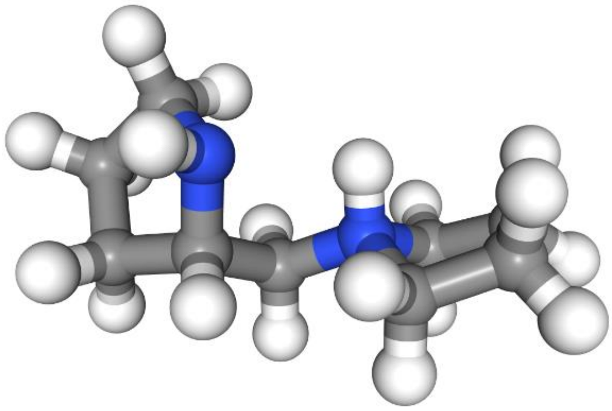

Figure S63. The lowest energy conformer of molecule 20 from the MM2* force field.

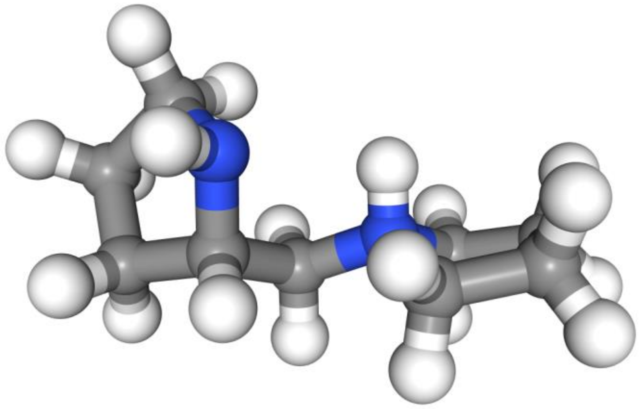

Figure S64. The lowest energy conformer of molecule 20 from the MM3* force field.

11. Additional Minor Tests

CREST Conformational Search on Molecule 5

We have performed a conformational search using the CREST tool on molecule 5, using the GFN2-xTB level of theory and the energy window for saving conformers set to 12 kcal mol^{-1} and all other settings left as their defaults. The resulting conformers were optimised with the same DFT method as used for the rest of this dataset.

The metrics for the CREST method on molecule 5 were computed to be as follows. The Spearman coefficient between the GFN2-xTB and DFT energies was 0.8285. The R^2 correlation coefficient between the GFN2-xTB and DFT energies was 0.3478. The MAD between the GFN2-xTB and DFT conformer relative energies was 22.313 kJ/mol. The average of the RMSDs between the geometries found from CREST and DFT was 0.3856. The proportion of the conformers that were predicted to be within 10 kJ mol^{-1} of the minimum energy conformer by the force field, that were actually within 10 kJ mol^{-1} of the minimum according to DFT was 0.556. The proportion of conformers remaining after redundant conformer elimination with RMSD cutoff set to 0.1 Å was 0.556. The proportion of distinct conformers found by CREST, out of the maximum number of distinct conformers found by any of the force fields was 0.5.

For this molecule, the results from the CREST conformational search are generally worse than most of the force fields. The Spearman coefficient for the CREST conformers is greater than that of the OPLS3e, MMFF, MMFFs, AMBER* and OPLS, indicating that the conformer ordering from CREST is better than these force fields. However, the CREST R^2 is only greater than that of the AMBER* and OPLS force fields, CREST only performs better than OPLS for the MAD between conformer relative energies and the proportion of the maximum possible number of conformers, and CREST performs worse than all of the force fields in the metrics that measure the RMSD between DFT conformer geometries, the proportion of conformers correctly identified to be within 10 kJ mol^{-1} of the minimum energy conformer and the proportion of conformers remaining after redundant conformer elimination. We stress that these results are only for a single molecule, and the performance of CREST relative to the force fields may shift given data from further molecules, however based on these results, we have determined that the force fields shall remain the focus of this study.

Conformational Search of MVK-Bound Molecule 1

We have performed a conformational search using the OPLS3e force field on catalyst 1 interacting with the methyl vinyl ketone, the structure of which may be seen in the image below. The conformers from this search were optimised using the same DFT level of theory as for all other molecules in this work.

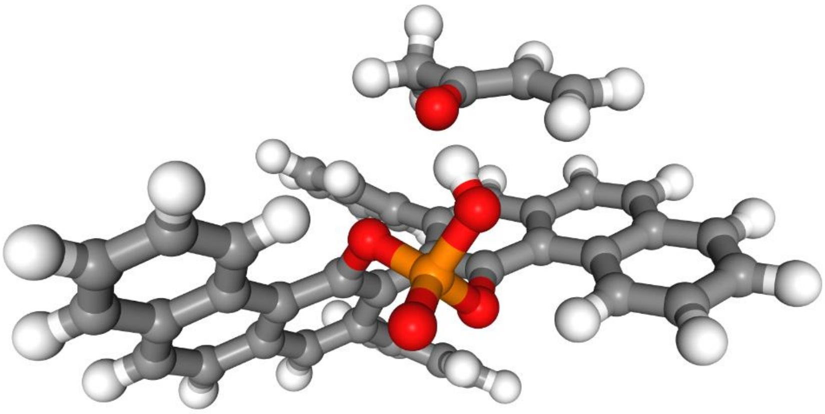

Figure S65. The 3D structure of molecule 1 bound to methyl vinyl ketone.

The metrics for the OPLS3e force field on this molecule are as follows. The Spearman coefficient between the force field and DFT energies was -0.4130. The R^2 correlation coefficient between the force field and DFT energies was 0.1417. The MAD between the force field and DFT relative energies was 8.426 kJ mol^{-1}. The mean RMSD between the force field and DFT conformer geometries was 0.5142. The proportion of the conformers that were predicted to be within 10 kJ mol^{-1} of the minimum energy conformer by the force field, that were actually within 10 kJ mol^{-1} of the minimum according to DFT was 0.3333. The proportion of conformers remaining after redundant conformer elimination with RMSD cutoff set to 0.1 Å was 0.9583.

This system contains 81 atoms, and the molecule in the dataset that is closest to this in number of atoms is catalyst 11 which has 85 atoms. The metrics for OPLS3e for molecule 11 are: Spearman = 0.2848; R^2 = 0.0877; MAD = 6.052 kJ mol^{-1}; RMSD = 0.3377 Å; proportion correctly identified within < 10 kJ mol^{-1} of minimum = 0.7273; proportion remaining after redundant conformer elimination = 0.8182. The performance of OPLS3e (and indeed the other force fields) for molecule 11 is quite poor, particularly in the Spearman and R^2 metrics, and the performance with the methyl vinyl ketone-bound catalyst 1 is generally worse still. It would seem that OPLS3e (and based on the results presented in this work, likely all of the other force fields used in this work) is not particularly suitable for describing the interactions between the species within this system, and perhaps force fields are better for describing smaller, more rigid molecules. We stress again however, that these results are for a single system and further work that goes beyond the scope of this paper is needed to compare force field performances for conformationally searching systems containing catalysts bound to activated substrates.

References

1. *MacroModel | Schrödinger*, https://www.schrodinger.com/products/macromodel [accessed March 2022].
2. M. Li, *Appl. Math. Comput.*, 2014, **248**, 314-322.
3. R. Fletcher and C. M. Reeves, *Computer Journal*, 1964, **7**, 149-154.
4. G. Chang, W. C. Guida and W. C. Still, *J. Am. Chem. Soc.*, 1989, **111**, 4379-4386.
5. I. Kolossváry and W. C. Guida, *J. Am. Chem. Soc.*, 1996, **118**, 5011-5019.
6. T. A. Halgren, *J. Comp. Chem*, 1999, **20**, 720-729.
7. Gaussian 16, Revision A.03, M. J. Frisch, G. W. Trucks, H. B. Schlegel, G. E. Scuseria, M. A. Robb, J. R. Cheeseman, G. Scalmani, V. Barone, G. A. Petersson, H. Nakatsuji, X. Li, M. Caricato, A. V. Marenich, J. Bloino, B. G. Janesko, R. Gomperts, B. Mennucci, H. P. Hratchian, J. V. Ortiz, A. F. Izmaylov, J. L. Sonnenberg, D. Williams-Young, F. Ding, F. Lipparini, F. Egidi, J. Goings, B. Peng, A. Petrone, T. Henderson, D. Ranasinghe, V. G. Zakrzewski, J. Gao, N. Rega, G. Zheng, W. Liang, M. Hada, M. Ehara, K. Toyota, R. Fukuda, J. Hasegawa, M. Ishida, T. Nakajima, Y. Honda, O. Kitao, H. Nakai, T. Vreven, K. Throssell, J. A. M. Jr., J. E. Peralta, F. Ogliaro, M. J. Bearpark, J. J. Heyd, E. N. Brothers, K. N. Kudin, V. N. Staroverov, T. A. Keith, R. Kobayashi, J. Normand, K. Raghavachari, A. P. Rendell, J. C. Burant, S. S. Iyengar, J. Tomasi, M. Cossi, J. M. Millam, M. Klene, C. Adamo, R. Cammi, J. W. Ochterski, R. L. Martin, K. Morokuma, O. Farkas, J. B. Foresman and D. J. Fox, *Gaussian, Inc.*, Wallingford CT, 2016.
8. B. N. Falcone and M. N. Grayson, *Org. Biomol. Chem.*, 2021, **19**, 3656-3664.
9. B. N. Falcone, M. N. Grayson and J. B. Rodriguez, *J. Org. Chem.*, 2018, **83**, 14683-14687.
10. P. B. Momo, A. N. Leveille, E. H. E. Farrar, M. N. Grayson, A. E. Mattson and A. C. B. Burtoloso, *Angew. Chem. Int. Ed.*, 2020, **59**, 15554-15559.
11. S. Grimme, *Chem. Eur. J.*, 2012, **18**, 9955-9964.
12. G. Luchini, J. V. Alegre-Requena, Y. Guan, I. Funes-Ardoiz and R. S. Paton, *GoodVibes: GoodVibes 3.0.1*, 2019, http://doi.org/10.5281/zenodo.595246 [accessed March 2022].

Coordinates of Lowest Energy Conformers from Force Fields

Molecule 1 AMBER

AMBER energy = 189.284 kJ/mol

C	-0.9734	1.3605	0.5697
C	-1.6058	0.1834	0.4780
C	-0.8016	-0.9977	0.1982
C	0.5433	-0.8615	0.0858
H	-1.5691	2.2340	0.7938
O	1.2935	-2.0676	-0.0161
C	-1.2086	-3.3684	0.8841
C	-1.4690	-2.3486	0.0289
C	-2.4181	-2.5655	-1.0537
C	-3.0238	-3.7582	-1.1217
O	-0.1456	-3.1598	1.8080
H	-3.7151	-3.9246	-1.9354
C	1.1366	0.3730	0.1326
C	0.3623	1.4713	0.4034
C	0.9067	2.7045	0.5284
C	2.2197	2.9505	0.4007
H	0.2732	3.5518	0.7517
H	2.5730	3.9640	0.5272
C	-2.7853	-4.7355	-0.2202
C	-1.8999	-4.5491	0.8096
C	-3.2883	-6.9616	0.4364
C	-3.4425	-5.9082	-0.3808
H	-3.8434	-7.8653	0.2293
H	-4.1268	-6.0315	-1.2089
C	3.0856	1.9714	0.1038
C	2.5900	0.6059	-0.0734
C	3.5552	-0.3638	-0.4478

C	4.8574	-0.0456	-0.5836
C	5.3166	1.2645	-0.3746
C	4.4579	2.2441	-0.0483
H	3.3166	-1.3836	-0.6755
H	5.5640	-0.8133	-0.8677
H	6.3679	1.4887	-0.4911
H	4.8343	3.2477	0.0899
C	-1.7402	-5.6684	1.7743
C	-2.4791	-6.9021	1.5034
C	-2.3415	-8.0049	2.3670
C	-1.5527	-7.9288	3.4514
C	-0.8628	-6.7428	3.7494
C	-0.9546	-5.6583	2.9552
H	-2.8755	-8.9234	2.1694
H	-1.4589	-8.7827	4.1078
H	-0.2517	-6.6976	4.6403
H	-0.4206	-4.7974	3.3056
C	-5.8990	0.0038	1.0511
C	-5.3435	0.9271	0.1501
C	-3.9504	0.9822	-0.0303
C	-3.0965	0.1173	0.6821
C	-3.6649	-0.7989	1.5892
C	-5.0572	-0.8601	1.7709
H	-6.9701	-0.0428	1.1902
H	-5.9851	1.5903	-0.4120
H	-3.5329	1.6762	-0.7460
H	-3.0278	-1.4647	2.1538
H	-5.4768	-1.5735	2.4661
C	-3.2965	0.4654	-4.0257
C	-4.3411	-0.1817	-3.3452

C	-4.0506	-1.1655	-2.3840
C	-2.7177	-1.5130	-2.0883
C	-1.6787	-0.8629	-2.7832
C	-1.9637	0.1234	-3.7432
H	-3.5166	1.2238	-4.7640
H	-5.3686	0.0802	-3.5529
H	-4.8588	-1.6394	-1.8451
H	-0.6496	-1.1226	-2.5797
H	-1.1542	0.6161	-4.2630
P	1.3576	-3.0518	1.2516
O	2.2254	-2.2995	2.3599
O	1.8892	-4.3773	0.8692
H	1.8436	-1.4218	2.4140

Molecule 1 MM2

MM2 energy = 257.312 kJ/mol

C	-1.9472	-2.1964	-2.4584
C	-1.1339	-3.3211	-2.3687
C	0.0175	-3.2393	-1.5802
C	0.3147	-2.0729	-0.8558
H	-2.8657	-2.2504	-3.0664
O	1.3815	-2.1060	-0.0154
C	1.2585	-5.0709	-0.3566
C	0.9744	-4.4106	-1.5635
C	1.5210	-4.8542	-2.7711
C	2.3095	-5.9998	-2.7505
O	0.7961	-4.5107	0.7914
H	2.7586	-6.3544	-3.6933
C	-0.4820	-0.9151	-0.9840
C	-1.6242	-1.0143	-1.7938

C	-2.4986	0.0598	-1.9577
C	-2.2496	1.2638	-1.3159
H	-3.3993	-0.0226	-2.5902
H	-2.9566	2.0992	-1.4584
C	2.5414	-6.6985	-1.5666
C	2.0152	-6.2620	-0.3407
C	3.6192	-8.5809	-0.5155
C	3.3348	-7.8421	-1.6544
H	4.2511	-9.4809	-0.6087
H	3.7528	-8.1799	-2.6184
C	-1.1169	1.3974	-0.5152
C	-0.2261	0.3219	-0.3465
C	0.8884	0.5946	0.4618
C	1.1052	1.8261	1.0786
C	0.1973	2.8626	0.9037
C	-0.9131	2.6376	0.0985
H	1.6801	-0.1361	0.6589
H	1.9994	1.9857	1.7056
H	0.3584	3.8416	1.3862
H	-1.6274	3.4665	-0.0447
C	2.2948	-7.0314	0.8134
C	3.0996	-8.1807	0.7141
C	3.4111	-8.9707	1.8254
C	2.9238	-8.6522	3.0877
C	2.1123	-7.5325	3.2176
C	1.8114	-6.7532	2.1012
H	4.0480	-9.8662	1.7252
H	3.1684	-9.2768	3.9636
H	1.7056	-7.2670	4.2087
H	1.1494	-5.9096	2.3245

C	-2.3455	-6.8757	-4.4881
C	-1.9346	-6.9607	-3.1594
C	-1.5470	-5.8110	-2.4727
C	-1.5556	-4.5640	-3.1031
C	-1.9835	-4.4890	-4.4317
C	-2.3725	-5.6357	-5.1229
H	-2.6511	-7.7845	-5.0335
H	-1.9187	-7.9389	-2.6496
H	-1.2431	-5.9028	-1.4174
H	-1.9996	-3.5209	-4.9597
H	-2.6966	-5.5620	-6.1747
C	0.9564	-2.8434	-6.5439
C	0.8175	-4.2271	-6.4575
C	1.0051	-4.8727	-5.2361
C	1.3255	-4.1480	-4.0845
C	1.4765	-2.7626	-4.1868
C	1.2896	-2.1121	-5.4056
H	0.8067	-2.3303	-7.5088
H	0.5535	-4.8115	-7.3551
H	0.8756	-5.9668	-5.1896
H	1.7567	-2.1632	-3.3054
H	1.4084	-1.0173	-5.4700
P	1.3470	-3.0947	1.2162
O	0.4040	-2.4883	2.3201
O	2.6966	-3.2178	1.7599
H	-0.4370	-2.4218	1.9048

Molecule 1 MMFF

MMFF energy = 433.248 kJ/mol

C	-0.9818	1.3515	0.6355

C	-1.6699	0.1321	0.4988
C	-0.9263	-1.0112	0.1460
C	0.4835	-0.8795	0.0285
H	-1.5544	2.2339	0.9262
O	1.1968	-2.0529	-0.0891
C	-1.2888	-3.4094	0.8190
C	-1.5606	-2.3440	-0.0835
C	-2.4164	-2.5985	-1.1723
C	-3.0217	-3.8650	-1.2518
O	-0.2885	-3.1725	1.7367
H	-3.6773	-4.0681	-2.1003
C	1.1979	0.3565	0.0991
C	0.4037	1.4764	0.4367
C	0.9859	2.7341	0.6076
C	2.3536	2.9188	0.4506
H	0.3773	3.5950	0.8794
H	2.7645	3.9147	0.6039
C	-2.8081	-4.8781	-0.3016
C	-1.9319	-4.6856	0.7925
C	-3.2926	-7.1437	0.4145
C	-3.4701	-6.0947	-0.4778
H	-3.8249	-8.0755	0.2342
H	-4.1366	-6.2470	-1.3251
C	3.1792	1.8560	0.0893
C	2.6276	0.5630	-0.1149
C	3.5486	-0.4313	-0.5442
C	4.9154	-0.1821	-0.7126
C	5.4207	1.0829	-0.4695
C	4.5543	2.0969	-0.0774
H	3.2168	-1.4333	-0.7946

H	5.5750	-0.9793	-1.0460
H	6.4798	1.2869	-0.6000
H	4.9640	3.0912	0.0912
C	-1.7706	-5.7804	1.7460
C	-2.4589	-7.0033	1.5216
C	-2.3251	-8.0899	2.4037
C	-1.5242	-8.0030	3.5363
C	-0.8547	-6.8216	3.8009
C	-0.9809	-5.7358	2.9264
H	-2.8529	-9.0226	2.2140
H	-1.4304	-8.8509	4.2086
H	-0.2318	-6.7322	4.6871
H	-0.4448	-4.8374	3.2107
C	-5.8789	0.1086	1.3685
C	-5.0114	-0.6981	2.1003
C	-3.6429	-0.6913	1.8180
C	-3.1188	0.1117	0.7914
C	-4.0107	0.9273	0.0741
C	-5.3791	0.9239	0.3568
H	-6.9430	0.1032	1.5890
H	-5.3957	-1.3325	2.8948
H	-2.9829	-1.3185	2.4143
H	-3.6413	1.5588	-0.7321
H	-6.0519	1.5549	-0.2175
C	-3.1739	0.1851	-4.3585
C	-4.2299	-0.3138	-3.6008
C	-3.9804	-1.2163	-2.5636
C	-2.6723	-1.6307	-2.2601
C	-1.6238	-1.1261	-3.0473
C	-1.8710	-0.2217	-4.0833

H	-3.3657	0.8880	-5.1648
H	-5.2485	0.0007	-3.8111
H	-4.8176	-1.5820	-1.9715
H	-0.5989	-1.4413	-2.8621
H	-1.0439	0.1605	-4.6758
P	1.2219	-3.0671	1.1704
O	1.9797	-2.2289	2.3159
O	1.9247	-4.3634	0.9171
H	2.9418	-2.3721	2.2631

Molecule 1 OPLS-2005

OPLS-2005 energy = 433.248 kJ/mol

C	-0.5714	1.4944	0.6080
C	-1.3537	0.3276	0.5806
C	-0.7277	-0.9052	0.2594
C	0.6769	-0.9394	0.0486
H	-1.0455	2.4317	0.8618
O	1.2410	-2.1778	-0.0466
C	-1.2527	-3.2145	1.0856
C	-1.5107	-2.1673	0.1612
C	-2.4995	-2.3483	-0.8404
C	-3.2536	-3.5337	-0.8351
O	-0.1549	-3.0444	1.8766
H	-4.0037	-3.6757	-1.5999
C	1.4703	0.2482	0.0201
C	0.8059	1.4670	0.3305
C	1.5046	2.6855	0.3966
C	2.8823	2.7201	0.1566
H	0.9893	3.6021	0.6451
H	3.4085	3.6614	0.2206

C	-3.0479	-4.5361	0.1283
C	-2.0477	-4.4008	1.1313
C	-3.6850	-6.7286	0.9639
C	-3.8535	-5.6857	0.0472
H	-4.3055	-7.6094	0.8857
H	-4.6016	-5.7797	-0.7267
C	3.5663	1.5408	-0.1825
C	2.8770	0.2950	-0.2763
C	3.6455	-0.8284	-0.6918
C	5.0257	-0.7295	-0.9505
C	5.6844	0.5012	-0.8150
C	4.9505	1.6362	-0.4380
H	3.2247	-1.8012	-0.8619
H	5.5779	-1.6039	-1.2640
H	6.7434	0.5784	-1.0152
H	5.4574	2.5866	-0.3540
C	-1.9030	-5.4583	2.0954
C	-2.7247	-6.6195	1.9833
C	-2.6106	-7.6920	2.8928
C	-1.6887	-7.6342	3.9488
C	-0.8874	-6.4932	4.0998
C	-0.9974	-5.4227	3.1921
H	-3.2354	-8.5669	2.7871
H	-1.6037	-8.4566	4.6443
H	-0.1843	-6.4338	4.9179
H	-0.3631	-4.5844	3.4059
C	-5.5341	0.7010	1.5853
C	-4.7214	-0.2108	2.2840
C	-3.3567	-0.3312	1.9576
C	-2.7857	0.4463	0.9248

C	-3.6119	1.3660	0.2410
C	-4.9771	1.4919	0.5636
H	-6.5814	0.7944	1.8341
H	-5.1422	-0.8183	3.0720
H	-2.7421	-1.0307	2.5058
H	-3.1979	1.9665	-0.5564
H	-5.5962	2.1926	0.0226
C	-3.2782	0.5110	-3.9776
C	-1.9714	0.0323	-3.7703
C	-1.7177	-0.9023	-2.7478
C	-2.7591	-1.3647	-1.9119
C	-4.0679	-0.8840	-2.1405
C	-4.3281	0.0500	-3.1621
H	-3.4747	1.2281	-4.7616
H	-1.1618	0.3792	-4.3959
H	-0.7109	-1.2671	-2.6033
H	-4.8785	-1.2193	-1.5096
H	-5.3333	0.4146	-3.3155
P	1.3348	-3.1602	1.2427
O	2.2261	-2.3098	2.2825
O	1.8454	-4.5322	0.9860
H	3.0283	-2.7668	2.4706

Molecule 1 OPLS3e

OPLS3e energy = 242.106 kJ/mol

C	-2.2847	-2.4195	-2.2560
C	-1.4184	-3.5056	-2.2177
C	-0.1841	-3.3741	-1.5494
C	0.1252	-2.1764	-0.8617
H	-3.2387	-2.5248	-2.7519

O	1.2940	-2.1513	-0.1483
C	1.2044	-5.1220	-0.3419
C	0.8080	-4.5177	-1.5597
C	1.3343	-4.9783	-2.7832
C	2.1695	-6.0883	-2.7825
O	0.6885	-4.5819	0.8061
H	2.5771	-6.4405	-3.7189
C	-0.7441	-1.0247	-0.9402
C	-1.9776	-1.1911	-1.6425
C	-2.9085	-0.1366	-1.7134
C	-2.6521	1.0805	-1.1105
H	-3.8413	-0.2849	-2.2384
H	-3.3838	1.8734	-1.1696
C	2.5293	-6.7575	-1.5987
C	2.0574	-6.2905	-0.3322
C	3.7560	-8.5676	-0.5432
C	3.3707	-7.8837	-1.6802
H	4.4028	-9.4289	-0.6280
H	3.7216	-8.2174	-2.6461
C	-1.4410	1.3063	-0.4335
C	-0.4611	0.2615	-0.3533
C	0.7677	0.5937	0.3086
C	0.9801	1.8473	0.8750
C	0.0038	2.8277	0.8083
C	-1.1953	2.5712	0.1585
H	1.5904	-0.0908	0.3802
H	1.9192	2.0616	1.3655
H	0.1834	3.7968	1.2518
H	-1.9476	3.3443	0.0956
C	2.4614	-7.0097	0.8511

C	3.3175	-8.1549	0.7264
C	3.7286	-8.8772	1.8754
C	3.3109	-8.4912	3.1410
C	2.4830	-7.3911	3.2917
C	2.0611	-6.6621	2.1836
H	4.3754	-9.7352	1.7627
H	3.6320	-9.0459	4.0110
H	2.1644	-7.0939	4.2806
H	1.4245	-5.8274	2.3982
C	-2.8333	-7.1615	-3.9649
C	-2.8457	-5.9872	-4.7131
C	-2.3832	-4.8033	-4.1437
C	-1.8989	-4.7835	-2.8295
C	-1.9063	-5.9718	-2.0884
C	-2.3657	-7.1586	-2.6532
H	-3.1902	-8.0821	-4.4039
H	-3.2065	-5.9935	-5.7312
H	-2.3743	-3.8977	-4.7333
H	-1.5498	-5.9725	-1.0677
H	-2.3590	-8.0701	-2.0731
C	0.7064	-3.0343	-6.5578
C	1.1420	-2.2925	-5.4628
C	1.3441	-2.9250	-4.2390
C	1.1032	-4.2970	-4.0950
C	0.6756	-5.0289	-5.2101
C	0.4734	-4.4018	-6.4372
H	0.5497	-2.5445	-7.5081
H	1.3250	-1.2320	-5.5589
H	1.6886	-2.3469	-3.3929
H	0.4741	-6.0863	-5.1139

H	0.1330	-4.9739	-7.2879
P	1.2910	-3.1172	1.1559
O	0.1271	-2.4781	2.0611
O	2.5756	-3.1412	1.8974
H	0.5181	-2.0825	2.8573

Molecule 2 AMBER

AMBER energy = 175.281 kJ/mol

C	-6.5213	-6.2839	-0.0993
C	-6.2240	-7.6295	-0.0136
C	-6.1621	-8.2912	1.2208
C	-6.3996	-7.5625	2.4064
C	-6.7160	-6.1842	2.3097
C	-6.7751	-5.5366	1.0589
O	-6.5241	-5.8457	-1.4014
N	-6.0173	-8.1276	-1.2771
H	-5.9320	-9.3464	1.2684
H	-6.9235	-5.6141	3.2047
H	-7.0104	-4.4842	0.9878
C	-6.1997	-7.0373	-2.0539
C	-6.0291	-7.1114	-3.5697
C	-5.4534	-8.4518	-4.0815
C	-3.9140	-8.6024	-4.0547
C	-3.2633	-7.3992	-4.7368
C	-3.6604	-6.1483	-3.9485
C	-5.1768	-5.9432	-4.1095
H	-5.7458	-8.5317	-5.1291
H	-5.9178	-9.2917	-3.5633
C	-3.5629	-9.8653	-4.8566
C	-3.2899	-8.7602	-2.6537

H	-2.1781	-7.5123	-4.7249
H	-3.6008	-7.3197	-5.7707
C	-2.9445	-4.9453	-4.5810
H	-5.3831	-5.8284	-5.1741
H	-5.4675	-5.0147	-3.6160
C	-7.4506	-6.9456	-4.1333
H	-3.9507	-9.7920	-5.8731
H	-2.4814	-9.9944	-4.9110
H	-3.9986	-10.7419	-4.3757
H	-3.1859	-4.8741	-5.6415
H	-3.2539	-4.0242	-4.0854
H	-1.8658	-5.0577	-4.4675
C	-3.2729	-6.2802	-2.4689
O	-3.1027	-5.2855	-1.7731
H	-2.2825	-9.1666	-2.7525
N	-3.1603	-7.4983	-1.9412
H	-3.8753	-9.4656	-2.0638
H	-2.9473	-7.5290	-0.9572
H	-8.0885	-7.7542	-3.7734
H	-7.8757	-5.9934	-3.8122
H	-7.4412	-6.9684	-5.2236
C	-6.3295	-8.2531	3.7186
O	-6.6853	-9.4178	3.8150
C	-4.8939	-6.1615	7.2121
C	-5.8939	-7.1407	7.3299
C	-6.3522	-7.8180	6.1859
C	-5.8263	-7.5247	4.9116
C	-4.8123	-6.5476	4.8095
C	-4.3509	-5.8673	5.9506
H	-4.5384	-5.6412	8.0911

H	-6.3095	-7.3772	8.2997
H	-7.1181	-8.5762	6.2797
H	-4.3714	-6.3210	3.8485
H	-3.5733	-5.1215	5.8578

Molecule 2 MM2

MM2 energy = 81.797 kJ/mol

C	-3.4715	-8.4861	3.2692
C	-3.9564	-7.2201	3.5487
C	-4.1191	-6.7541	4.8459
C	-3.7586	-7.6043	5.8975
C	-3.2853	-8.8940	5.6209
C	-3.1319	-9.3456	4.3075
O	-3.4198	-8.6956	1.8892
N	-4.2074	-6.6470	2.3696
H	-4.5136	-5.7391	5.0169
H	-3.0440	-9.5954	6.4360
H	-2.7610	-10.3607	4.0908
C	-3.9145	-7.4966	1.4438
C	-4.1156	-7.1832	-0.0346
C	-5.0113	-5.9497	-0.2771
C	-6.5427	-6.1609	-0.1938
C	-6.9535	-7.3589	-1.0626
C	-6.2084	-8.6128	-0.5820
C	-4.6936	-8.4057	-0.7763
H	-4.7990	-5.6080	-1.3165
H	-4.7028	-5.1027	0.3785
C	-7.2353	-4.8814	-0.7181
C	-7.0529	-6.4323	1.2330
H	-8.0558	-7.5188	-1.0020

H	-6.7142	-7.1581	-2.1330
C	-6.6754	-9.8388	-1.3915
H	-4.5110	-8.2708	-1.8680
H	-4.1390	-9.3300	-0.4913
C	-2.6870	-6.9002	-0.5534
H	-6.9310	-4.6451	-1.7633
H	-8.3446	-4.9841	-0.7222
H	-6.9814	-4.0004	-0.0851
H	-6.5205	-9.6829	-2.4836
H	-6.1183	-10.7616	-1.1096
H	-7.7606	-10.0365	-1.2340
C	-6.4903	-8.8464	0.8989
O	-6.3987	-9.9466	1.4114
H	-8.1566	-6.2845	1.2763
N	-6.8302	-7.7810	1.7145
H	-6.6116	-5.7049	1.9514
H	-6.9997	-8.0569	2.6818
H	-2.0130	-7.7745	-0.4003
H	-2.6797	-6.6716	-1.6437
H	-2.2331	-6.0269	-0.0305
C	-3.9513	-7.1302	7.2977
O	-4.8186	-6.3213	7.5419
C	-1.4760	-8.5550	10.4999
C	-2.7833	-8.1396	10.7459
C	-3.5798	-7.6809	9.6972
C	-3.0817	-7.6472	8.3922
C	-1.7633	-8.0445	8.1551
C	-0.9660	-8.5017	9.2041
H	-0.8444	-8.9158	11.3292
H	-3.1871	-8.1725	11.7720

H	-4.6115	-7.3576	9.9156
H	-1.3196	-7.9859	7.1478
H	0.0737	-8.8155	9.0099

Molecule 2 MM3

MM3 energy = 206.151 kJ/mol

C	-6.9219	-3.4179	-6.1743
C	-7.2504	-4.7414	-5.9292
C	-7.7408	-5.5560	-6.9412
C	-7.8925	-5.0296	-8.2356
C	-7.5654	-3.6800	-8.4648
C	-7.0762	-2.8666	-7.4367
O	-6.4703	-2.8542	-5.0239
N	-6.9906	-4.9911	-4.5560
H	-8.0081	-6.5998	-6.7115
H	-7.6936	-3.2547	-9.4748
H	-6.8178	-1.8108	-7.6182
C	-6.5002	-3.8215	-4.0544
C	-6.1381	-3.6256	-2.6035
C	-5.0499	-2.5399	-2.4145
C	-3.5954	-2.9596	-2.7429
C	-3.2460	-4.2209	-1.9417
C	-4.2110	-5.3470	-2.3199
C	-5.6383	-4.9227	-1.9212
H	-5.0717	-2.2198	-1.3469
H	-5.3188	-1.6316	-2.9996
C	-2.6551	-1.8072	-2.3236
C	-3.3672	-3.2675	-4.2238
H	-2.1962	-4.5287	-2.1511
H	-3.3062	-4.0111	-0.8498

C	-3.8312	-6.6074	-1.5240
H	-5.6607	-4.7739	-0.8170
H	-6.3506	-5.7540	-2.1255
C	-7.4293	-3.1579	-1.8921
H	-1.5844	-2.0649	-2.4824
H	-2.8598	-0.8800	-2.9039
H	-2.7680	-1.5517	-1.2467
H	-3.8353	-6.4205	-0.4271
H	-4.5368	-7.4473	-1.7114
H	-2.8124	-6.9685	-1.7891
C	-4.1326	-5.6263	-3.8254
O	-4.3920	-6.7177	-4.2747
H	-2.2772	-3.1684	-4.4343
N	-3.7958	-4.5845	-4.6702
H	-3.8515	-2.4740	-4.8361
H	-3.8082	-4.8036	-5.6755
H	-7.2679	-2.9799	-0.8058
H	-7.8153	-2.2074	-2.3240
H	-8.2431	-3.9122	-1.9800
C	-8.4105	-5.8636	-9.3493
O	-9.1064	-5.3833	-10.2170
C	-7.3978	-10.0380	-9.4310
C	-8.6436	-9.6053	-9.8829
C	-8.9634	-8.2471	-9.8535
C	-8.0489	-7.3034	-9.3674
C	-6.7945	-7.7521	-8.9284
C	-6.4723	-9.1100	-8.9561
H	-7.1431	-11.1112	-9.4540
H	-9.3764	-10.3363	-10.2649
H	-9.9513	-7.9139	-10.2141

H	-6.0431	-7.0322	-8.5636
H	-5.4827	-9.4492	-8.6046

Molecule 2 MMFF

MMFF energy = 336.176 kJ/mol

C	-4.7614	-2.9548	1.5724
C	-3.8157	-3.0196	0.5636
C	-2.8750	-1.9898	0.4377
C	-2.9335	-0.9325	1.3635
C	-3.9266	-0.8945	2.3598
C	-4.8697	-1.9217	2.4931
O	-5.5425	-4.0668	1.4830
N	-3.9982	-4.1625	-0.1720
H	-2.1475	-2.0230	-0.3646
H	-3.9690	-0.0476	3.0451
H	-5.6409	-1.9099	3.2531
C	-5.0480	-4.7366	0.3922
C	-5.7093	-6.0470	0.0285
C	-5.4861	-6.3886	-1.4702
C	-6.4711	-5.7432	-2.4818
C	-7.9127	-6.0581	-2.0597
C	-8.1809	-5.4540	-0.6763
C	-7.2255	-6.0812	0.3695
H	-5.5827	-7.4792	-1.5809
H	-4.4534	-6.1613	-1.7682
C	-6.1991	-6.3483	-3.8723
C	-6.3288	-4.2128	-2.5801
H	-8.6174	-5.6483	-2.7965
H	-8.0757	-7.1440	-2.0403
C	-9.6342	-5.7523	-0.2678

H	-7.5202	-7.1357	0.4781
H	-7.4094	-5.6296	1.3535
C	-4.9972	-7.1271	0.8821
H	-6.8935	-5.9529	-4.6224
H	-5.1807	-6.1214	-4.2069
H	-6.3140	-7.4381	-3.8617
H	-9.8530	-5.3731	0.7371
H	-10.3432	-5.2788	-0.9569
H	-9.8351	-6.8295	-0.2680
C	-7.9880	-3.9364	-0.7324
O	-8.6455	-3.1437	-0.0637
H	-6.8146	-3.8465	-3.4931
N	-6.9533	-3.4805	-1.4997
H	-5.2792	-3.9058	-2.6352
H	-6.8611	-2.4710	-1.4909
H	-5.1219	-6.9327	1.9543
H	-5.3964	-8.1272	0.6784
H	-3.9202	-7.1523	0.6755
C	-1.9810	0.2040	1.2835
O	-2.3808	1.3341	1.5507
C	2.1130	-0.2702	0.1332
C	1.4109	0.8857	-0.2073
C	0.0761	1.0285	0.1742
C	-0.5631	0.0077	0.8877
C	0.1507	-1.1422	1.2472
C	1.4862	-1.2806	0.8630
H	3.1542	-0.3787	-0.1613
H	1.9036	1.6788	-0.7643
H	-0.4649	1.9368	-0.0843
H	-0.3091	-1.9290	1.8392

H	2.0423	-2.1730	1.1405

Molecule 2 MMFF

MMFF energy = 333.885 kJ/mol

C	-5.8565	-2.0158	-2.9621
C	-6.4060	-1.9838	-1.6920
C	-5.7178	-1.3294	-0.6628
C	-4.4905	-0.7198	-0.9800
C	-3.9565	-0.7968	-2.2797
C	-4.6400	-1.4480	-3.3146
O	-6.7154	-2.6968	-3.7701
N	-7.6046	-2.6503	-1.6872
H	-6.1313	-1.3214	0.3387
H	-2.9880	-0.3430	-2.4913
H	-4.2451	-1.5180	-4.3205
C	-7.7331	-3.0776	-2.9324
C	-8.8733	-3.8597	-3.5442
C	-9.6257	-4.6874	-2.4665
C	-9.0130	-6.0661	-2.0972
C	-8.8028	-6.8888	-3.3746
C	-7.8276	-6.1522	-4.3004
C	-8.4213	-4.7821	-4.7103
H	-10.6412	-4.8793	-2.8444
H	-9.7678	-4.0930	-1.5535
C	-10.0032	-6.7985	-1.1711
C	-7.6586	-5.9560	-1.3729
H	-8.4088	-7.8826	-3.1202
H	-9.7616	-7.0534	-3.8842
C	-7.5962	-6.9983	-5.5644
H	-9.3012	-4.9878	-5.3380

H	-7.7163	-4.2550	-5.3671
C	-9.8524	-2.8026	-4.1142
H	-9.6386	-7.7978	-0.9074
H	-10.1553	-6.2413	-0.2399
H	-10.9803	-6.9228	-1.6514
H	-8.5399	-7.2137	-6.0779
H	-6.9403	-6.4839	-6.2763
H	-7.1221	-7.9552	-5.3171
C	-6.4853	-5.9493	-3.5952
O	-5.4145	-5.8745	-4.1924
H	-7.4412	-6.8849	-0.8311
N	-6.5201	-5.7597	-2.2443
H	-7.6602	-5.1545	-0.6271
H	-5.6157	-5.5668	-1.8291
H	-9.3698	-2.1869	-4.8828
H	-10.7302	-3.2739	-4.5707
H	-10.2106	-2.1266	-3.3282
C	-3.6947	-0.0220	0.0617
O	-2.4696	-0.1023	0.0267
C	-5.4798	2.2496	3.2178
C	-6.0851	2.2321	1.9610
C	-5.5157	1.4964	0.9193
C	-4.3423	0.7653	1.1417
C	-3.7266	0.8033	2.3984
C	-4.2992	1.5400	3.4360
H	-5.9227	2.8268	4.0261
H	-6.9966	2.8002	1.7907
H	-5.9885	1.5196	-0.0589
H	-2.7994	0.2598	2.5699
H	-3.8204	1.5650	4.4119

Molecule 2 OPLS-2005

OPLS-2005 energy = 333.885 kJ/mol

C	-9.0030	1.2566	-3.2147
C	-8.4610	0.3025	-4.1000
C	-7.2731	0.6285	-4.7823
C	-6.6435	1.8833	-4.5791
C	-7.2309	2.8260	-3.6917
C	-8.4167	2.5113	-2.9935
O	-10.1414	0.7202	-2.6528
N	-9.2552	-0.8238	-4.1046
H	-6.8478	-0.0880	-5.4700
H	-6.7774	3.7991	-3.5505
H	-8.8806	3.2116	-2.3151
C	-10.2692	-0.4886	-3.2874
C	-11.3759	-1.4096	-2.8257
C	-11.6822	-2.5224	-3.8759
C	-12.6202	-2.1538	-5.0634
C	-13.9185	-1.5599	-4.4974
C	-13.5985	-0.2734	-3.7110
C	-12.6997	-0.6452	-2.4995
H	-12.1706	-3.3383	-3.3419
H	-10.7545	-2.9598	-4.2477
C	-12.9370	-3.4478	-5.8385
C	-12.0010	-1.1283	-6.0530
H	-14.6145	-1.3385	-5.3089
H	-14.4219	-2.2829	-3.8540
C	-14.9175	0.3472	-3.2013
H	-13.3010	-1.2798	-1.8483
H	-12.4914	0.2426	-1.9004

C	-10.8688	-2.0832	-1.5342
H	-13.6280	-3.2640	-6.6622
H	-12.0326	-3.8868	-6.2620
H	-13.3966	-4.2002	-5.1966
H	-14.7312	1.2246	-2.5799
H	-15.5520	0.6669	-4.0290
H	-15.4898	-0.3609	-2.6022
C	-12.8714	0.7395	-4.6261
O	-12.9295	1.9444	-4.4045
H	-12.4898	-1.2081	-7.0249
N	-12.1451	0.2709	-5.6473
H	-10.9479	-1.3503	-6.2309
H	-11.6601	0.9674	-6.1914
H	-10.6484	-1.3416	-0.7653
H	-11.5990	-2.7782	-1.1196
H	-9.9503	-2.6417	-1.7195
C	-5.3972	2.1880	-5.3478
O	-5.2123	1.6944	-6.4608
C	-2.4372	4.8269	-3.6280
C	-3.1469	3.9417	-2.7946
C	-4.1147	3.0782	-3.3449
C	-4.3892	3.1005	-4.7333
C	-3.6583	3.9808	-5.5630
C	-2.6903	4.8441	-5.0126
H	-1.6927	5.4871	-3.2065
H	-2.9446	3.9208	-1.7332
H	-4.6415	2.3934	-2.6948
H	-3.8415	3.9946	-6.6289
H	-2.1391	5.5164	-5.6545

Molecule 2 OPLS3e

OPLS3e energy = 301.668 kJ/mol

C	-2.8657	-2.8934	-3.3087
C	-1.9447	-3.8888	-3.6633
C	-0.6081	-3.5397	-3.8855
C	-0.2247	-2.1905	-3.7322
C	-1.1911	-1.2159	-3.4067
C	-2.5311	-1.5458	-3.1731
O	-4.0741	-3.4862	-3.1458
N	-2.5769	-5.0969	-3.7312
H	0.1072	-4.2983	-4.1715
H	-0.9040	-0.1724	-3.3482
H	-3.2837	-0.8116	-2.9262
C	-3.8346	-4.7972	-3.4597
C	-5.0105	-5.7852	-3.3799
C	-4.9771	-6.8605	-4.5132
C	-5.4002	-6.3927	-5.9413
C	-6.8705	-5.9438	-5.8525
C	-6.9872	-4.7388	-4.8825
C	-6.4107	-5.0791	-3.4628
H	-5.6063	-7.7080	-4.2341
H	-3.9646	-7.2656	-4.5686
C	-5.3078	-7.5947	-6.9106
C	-4.5305	-5.2142	-6.4976
H	-7.2532	-5.6804	-6.8419
H	-7.5020	-6.7631	-5.5042
C	-8.4682	-4.2923	-4.7545
H	-7.1285	-5.7781	-3.0306
H	-6.4531	-4.2014	-2.8137
C	-4.9152	-6.5215	-2.0195

H	-5.6618	-7.3382	-7.9104
H	-4.2800	-7.9465	-7.0111
H	-5.9063	-8.4398	-6.5672
H	-8.8831	-3.9813	-5.7151
H	-9.0983	-5.0980	-4.3772
H	-8.5786	-3.4483	-4.0707
C	-6.1696	-3.5519	-5.4969
O	-6.4414	-2.3634	-5.3719
H	-4.5860	-5.2194	-7.5880
N	-5.0596	-3.8693	-6.1852
H	-3.4758	-5.3072	-6.2363
H	-4.5770	-3.0532	-6.5255
H	-3.9640	-7.0473	-1.9200
H	-4.9733	-5.8191	-1.1864
H	-5.7090	-7.2570	-1.8835
C	1.2044	-1.8026	-4.0141
O	1.8619	-2.4654	-4.8219
C	3.1219	1.4965	-2.0369
C	2.0828	0.8275	-1.3957
C	1.4508	-0.2386	-2.0307
C	1.8411	-0.6289	-3.3215
C	2.9116	0.0346	-3.9391
C	3.5427	1.1007	-3.3035
H	3.6151	2.3210	-1.5424
H	1.7756	1.1273	-0.4040
H	0.6677	-0.7639	-1.5008
H	3.2556	-0.2717	-4.9189
H	4.3602	1.6130	-3.7904

Molecule 2 OPLS

OPLS energy = 119.998 kJ/mol

C	-9.8659	-3.7085	-6.3387
C	-8.6739	-3.2328	-5.8406
C	-7.6730	-2.7189	-6.6719
C	-7.9020	-2.6850	-8.0623
C	-9.1237	-3.1847	-8.5782
C	-10.1156	-3.6958	-7.7160
O	-10.6922	-4.1629	-5.3381
N	-8.6808	-3.3676	-4.4452
H	-6.7448	-2.3556	-6.2555
H	-9.3047	-3.1907	-9.6435
H	-11.0470	-4.0793	-8.1061
C	-9.8915	-3.9235	-4.2158
C	-10.3411	-4.2868	-2.7985
C	-9.2839	-4.0705	-1.6846
C	-8.3363	-5.2551	-1.3673
C	-9.1561	-6.5271	-1.1361
C	-9.9010	-6.8530	-2.4330
C	-10.9143	-5.7219	-2.7115
H	-9.8433	-3.8955	-0.7661
H	-8.7037	-3.1658	-1.8644
C	-7.5968	-4.9102	-0.0646
C	-7.2643	-5.5477	-2.4385
H	-8.4924	-7.3525	-0.8755
H	-9.8640	-6.3733	-0.3208
C	-10.7031	-8.1451	-2.2167
H	-11.6289	-5.7375	-1.8883
H	-11.4691	-5.9558	-3.6198
C	-11.5013	-3.3109	-2.5355
H	-6.9853	-4.0202	-0.2147

H	-8.3064	-4.7210	0.7412
H	-6.9521	-5.7356	0.2384
H	-11.3221	-8.3600	-3.0883
H	-10.0187	-8.9781	-2.0532
H	-11.3500	-8.0479	-1.3446
C	-8.9044	-7.0356	-3.5840
O	-9.1699	-7.7517	-4.5429
H	-6.4549	-6.1223	-1.9884
N	-7.7621	-6.3480	-3.5506
H	-6.8446	-4.6137	-2.8102
H	-7.1681	-6.4517	-4.3578
H	-12.2907	-3.4576	-3.2732
H	-11.9309	-3.4589	-1.5443
H	-11.1370	-2.2856	-2.6080
C	-6.8337	-2.1471	-8.9551
O	-5.6623	-2.2843	-8.6409
C	-7.7873	-0.1345	-12.6479
C	-6.6230	-0.9072	-12.5364
C	-6.3223	-1.5565	-11.3293
C	-7.1850	-1.4459	-10.2266
C	-8.3454	-0.6571	-10.3398
C	-8.6462	-0.0079	-11.5471
H	-8.0178	0.3673	-13.5770
H	-5.9540	-1.0015	-13.3798
H	-5.4213	-2.1480	-11.2459
H	-9.0104	-0.5347	-9.4975
H	-9.5390	0.5957	-11.6276

Molecule 3 AMBER

AMBER energy = -20.156 kJ/mol

C	0.2228	0.1805	3.2908
C	-0.8808	-0.6398	3.6028
C	-1.8038	-0.9335	2.5805
C	-1.6465	-0.4394	1.2709
C	-0.5247	0.3689	0.9905
C	0.4152	0.6801	1.9887
H	0.9478	0.4201	4.0570
C	-1.0985	-1.2072	5.0027
H	-2.6575	-1.5573	2.8070
C	-2.6776	-0.7945	0.2031
H	-0.3734	0.7544	-0.0085
N	1.5285	1.4734	1.6858
F	-2.4483	-0.1843	-1.0161
F	-2.6673	-2.1615	0.0093
F	-3.9341	-0.4187	0.6360
F	-0.0609	-0.9477	5.8789
F	-2.2534	-0.6647	5.5291
F	-1.2526	-2.5781	4.9255
H	1.4705	2.4795	1.6202
C	2.7265	0.8613	1.5805
O	3.7595	1.5495	1.4116
N	2.7993	-0.4748	1.6657
H	1.9621	-1.0006	1.8618
C	6.0121	-2.2447	0.7027
C	4.7171	-1.4561	0.4813
C	4.0352	-1.2136	1.8314
C	3.7027	-2.5675	2.4771
C	5.0064	-3.3312	2.7487
C	5.7307	-3.5702	1.4191
H	6.6988	-1.6477	1.3046

H	6.4795	-2.4475	-0.2618
H	4.0534	-2.0154	-0.1794
H	4.9590	-0.5013	0.0126
H	4.7184	-0.6489	2.4674
N	2.8534	-2.3905	3.6901
H	3.1211	-3.1397	1.7521
H	5.6589	-2.7557	3.4060
H	4.7984	-4.2977	3.2070
H	5.1146	-4.2031	0.7787
H	6.6746	-4.0826	1.6104
C	3.5344	-1.7851	4.8577
C	2.1524	-3.6330	4.0862
H	4.2847	-2.4632	5.2650
H	4.0086	-0.8428	4.5872
H	2.8001	-1.5774	5.6366
H	2.8598	-4.3908	4.4227
H	1.4601	-3.4202	4.9011
H	1.5788	-4.0260	3.2463

Molecule 3 MM2

MM2 energy = -140.266 kJ/mol

C	1.4478	-2.8719	1.1519
C	0.7436	-3.1968	2.3131
C	-0.3789	-2.4393	2.6567
C	-0.8075	-1.3796	1.8556
C	-0.0872	-1.0787	0.6985
C	1.0513	-1.8051	0.3429
H	2.3379	-3.4540	0.8625
C	1.1617	-4.3368	3.2074
H	-0.9313	-2.6950	3.5749

C	-2.0189	-0.5475	2.1924
H	-0.4454	-0.2568	0.0575
N	1.7503	-1.5350	-0.7761
F	-2.6319	-0.9372	3.3784
F	-1.6593	0.7905	2.3280
F	-2.9696	-0.6323	1.1802
F	2.2547	-5.0425	2.7150
F	1.5051	-3.8598	4.4686
F	0.1172	-5.2428	3.3637
H	2.0945	-2.3223	-1.3280
C	2.0272	-0.3666	-1.3684
O	2.4925	-0.3925	-2.5318
N	1.8271	0.7881	-0.7223
H	1.6327	0.7227	0.2730
C	3.4404	4.1387	-1.5657
C	3.2935	2.7508	-0.9251
C	1.9846	2.0842	-1.3791
C	0.7714	2.9659	-1.0062
C	0.9129	4.3516	-1.6637
C	2.2292	5.0243	-1.2398
H	3.5331	4.0304	-2.6727
H	4.3777	4.6270	-1.2053
H	3.3057	2.8481	0.1864
H	4.1629	2.1097	-1.2073
H	2.0333	1.9782	-2.4877
N	-0.4968	2.2863	-1.2590
H	0.8363	3.1134	0.0987
H	0.9031	4.2546	-2.7742
H	0.0642	5.0173	-1.3811
H	2.2057	5.2283	-0.1426

H	2.3340	6.0116	-1.7512
C	-0.7811	1.9833	-2.6489
C	-1.6343	2.9174	-0.6137
H	-1.7026	1.3619	-2.7200
H	-0.9355	2.9085	-3.2490
H	0.0295	1.3758	-3.1059
H	-1.8987	3.8866	-1.0942
H	-2.5200	2.2440	-0.6648
H	-1.4299	3.0872	0.4677

Molecule 3 MM3

MM3 energy = 197.32 kJ/mol

C	1.1138	-1.3505	2.9959
C	-0.1121	-0.6961	2.7985
C	-0.7933	-0.9066	1.5888
C	-0.2791	-1.7505	0.5925
C	0.9481	-2.3863	0.8305
C	1.6566	-2.1876	2.0181
H	1.6731	-1.1969	3.9351
C	-0.7060	0.2220	3.8509
H	-1.7588	-0.3949	1.4249
C	-1.0082	-1.9912	-0.7163
H	1.3597	-3.0722	0.0683
N	2.8694	-2.8319	2.2369
F	-1.2583	-3.3075	-0.8622
F	-2.1742	-1.3191	-0.7793
F	-0.2267	-1.5941	-1.7408
F	-0.8658	1.4551	3.3302
F	-1.9187	-0.2404	4.2152
F	0.0704	0.3118	4.9483

H	2.9199	-3.1973	3.1948
C	3.9584	-3.0856	1.4266
O	4.8243	-3.8463	1.7842
N	4.0143	-2.4471	0.2037
H	3.3308	-1.7049	0.0154
C	7.4710	-2.2715	-1.4309
C	6.3508	-1.9745	-0.4226
C	5.0660	-2.7261	-0.7713
C	4.6164	-2.3320	-2.2014
C	5.7198	-2.6713	-3.2175
C	7.0300	-1.9507	-2.8652
H	7.7584	-3.3460	-1.3616
H	8.3798	-1.6801	-1.1732
H	6.1511	-0.8785	-0.3995
H	6.6946	-2.2551	0.5995
H	5.3039	-3.8160	-0.7666
N	3.3152	-2.9453	-2.5379
H	4.4745	-1.2271	-2.2065
H	5.9049	-3.7690	-3.2362
H	5.4104	-2.3770	-4.2458
H	6.8910	-0.8504	-2.9743
H	7.8305	-2.2449	-3.5827
C	2.6614	-2.3279	-3.6912
C	3.2852	-4.4052	-2.6312
H	3.2009	-2.5517	-4.6336
H	1.6218	-2.7012	-3.7931
H	2.6055	-1.2269	-3.5684
H	3.8716	-4.7695	-3.4987
H	3.6832	-4.8746	-1.7106
H	2.2414	-4.7616	-2.7499

Molecule 3 MMFF

MMFF energy = 40.87 kJ/mol

C	0.7821	-0.9744	2.3504
C	0.0845	-0.7708	3.5527
C	-1.1035	-0.0255	3.5291
C	-1.5952	0.5042	2.3261
C	-0.8853	0.2699	1.1355
C	0.3001	-0.4676	1.1376
H	1.7102	-1.5445	2.3717
C	0.6282	-1.3137	4.8520
H	-1.6512	0.1455	4.4576
C	-2.8840	1.2915	2.2918
H	-1.2824	0.6871	0.2122
N	1.0632	-0.7252	-0.0160
F	-3.9367	0.5464	1.8584
F	-3.2464	1.7701	3.5160
F	-2.8246	2.3786	1.4750
F	1.3496	-2.4578	4.6904
F	1.4592	-0.4315	5.4707
F	-0.3455	-1.6118	5.7556
H	1.8928	-1.2902	0.0924
C	0.7333	-0.4515	-1.3260
O	-0.3247	0.0070	-1.7331
N	1.7797	-0.7893	-2.1607
H	2.6903	-0.5508	-1.7834
C	0.7261	-1.2026	-5.8218
C	0.7610	-1.4789	-4.3206
C	1.7106	-0.5127	-3.5967
C	3.1433	-0.6038	-4.2127

C	3.0887	-0.2960	-5.7211
C	2.1198	-1.2356	-6.4405
H	0.2724	-0.2198	-5.9996
H	0.0870	-1.9424	-6.3169
H	1.0786	-2.5149	-4.1445
H	-0.2555	-1.3974	-3.9196
H	1.3198	0.5064	-3.7141
N	4.1205	0.1915	-3.4165
H	3.4477	-1.6558	-4.1028
H	2.7652	0.7379	-5.8912
H	4.0794	-0.4036	-6.1757
H	2.5109	-2.2598	-6.4020
H	2.0582	-0.9586	-7.4990
C	3.9648	1.6408	-3.5575
C	5.5028	-0.1917	-3.7148
H	4.1928	1.9929	-4.5692
H	2.9539	1.9656	-3.2945
H	4.6378	2.1594	-2.8648
H	6.1928	0.3101	-3.0269
H	5.6488	-1.2674	-3.5673
H	5.8002	0.0625	-4.7375

Molecule 3 MMFF

MMFF energy = 47.905 kJ/mol

C	0.1047	-1.3526	0.8033
C	-0.8387	-0.5724	1.4949
C	-0.3901	0.3561	2.4467
C	0.9793	0.5147	2.7042
C	1.9000	-0.2665	1.9860
C	1.4727	-1.2070	1.0405

H	-0.2638	-2.0699	0.0728
C	-2.3131	-0.7631	1.2268
H	-1.1152	0.9621	2.9929
C	1.4619	1.5340	3.7076
H	2.9639	-0.1346	2.1803
N	2.4689	-1.9484	0.3771
F	1.6755	2.7527	3.1413
F	0.5816	1.7400	4.7254
F	2.6386	1.1836	4.2978
F	-3.0755	0.2685	1.6891
F	-2.6051	-0.8711	-0.0981
F	-2.8080	-1.8839	1.8186
H	3.4266	-1.7583	0.6289
C	2.2999	-2.9596	-0.5434
O	1.2368	-3.4425	-0.9085
N	3.5142	-3.3978	-1.0175
H	4.3056	-2.7696	-0.9702
C	3.3911	-6.9164	-2.5304
C	3.2376	-5.8288	-1.4697
C	3.6416	-4.4526	-2.0206
C	5.1119	-4.5027	-2.5504
C	5.2382	-5.5837	-3.6410
C	4.7997	-6.9511	-3.1141
H	2.6681	-6.7414	-3.3367
H	3.1505	-7.8923	-2.0937
H	3.8535	-6.0759	-0.5953
H	2.1991	-5.8188	-1.1206
H	2.9562	-4.1974	-2.8392
N	5.6116	-3.1472	-2.9113
H	5.7240	-4.8276	-1.6952

H	4.6208	-5.3265	-4.5099
H	6.2725	-5.6644	-3.9921
H	5.5044	-7.2854	-2.3425
H	4.8434	-7.6877	-3.9243
C	7.0688	-3.1209	-3.0560
C	4.9813	-2.5804	-4.1053
H	7.4191	-2.0897	-3.1787
H	7.5568	-3.5069	-2.1543
H	7.4191	-3.7007	-3.9163
H	5.3181	-1.5477	-4.2535
H	5.2201	-3.1423	-5.0145
H	3.8934	-2.5295	-4.0022

Molecule 3 OPLS-2005

OPLS-2005 energy = 47.905 kJ/mol

C	13.9597	-1.7906	-0.8219
C	15.2787	-2.0950	-1.2198
C	15.6078	-2.0928	-2.5906
C	14.6384	-1.7670	-3.5605
C	13.3220	-1.4642	-3.1517
C	12.9707	-1.4932	-1.7813
H	13.7126	-1.8033	0.2305
C	16.3295	-2.4426	-0.1773
H	16.6129	-2.3368	-2.9033
C	15.0172	-1.7588	-5.0334
H	12.5906	-1.2035	-3.9042
N	11.6641	-1.1686	-1.3204
F	14.9843	-3.0065	-5.4981
F	16.2505	-1.2770	-5.1865
F	14.1812	-1.0064	-5.7488

F	17.5514	-2.1399	-0.6157
F	16.1080	-1.7715	0.9530
F	16.2729	-3.7484	0.0771
H	11.5981	-0.5930	-0.4941
C	10.4645	-1.4720	-1.8384
O	9.4468	-0.9287	-1.4293
N	10.4704	-2.3954	-2.8024
H	11.3580	-2.7452	-3.1261
C	7.1589	-4.0407	-3.8302
C	8.3156	-3.6437	-2.8958
C	9.3420	-2.7680	-3.6417
C	9.8819	-3.5307	-4.8743
C	8.7227	-3.8776	-5.8345
C	7.6683	-4.7318	-5.1083
H	6.5917	-3.1486	-4.1001
H	6.4642	-4.6958	-3.3031
H	8.7996	-4.5400	-2.5058
H	7.9160	-3.1077	-2.0336
H	8.8412	-1.8525	-3.9623
N	11.0192	-2.8251	-5.4899
H	10.2830	-4.4756	-4.5015
H	8.2489	-2.9694	-6.2079
H	9.0823	-4.4251	-6.7048
H	8.0996	-5.7001	-4.8504
H	6.8319	-4.9377	-5.7772
C	10.6563	-1.5899	-6.1828
C	11.7772	-3.6865	-6.3926
H	11.5512	-1.0761	-6.5369
H	10.0225	-1.7798	-7.0499
H	10.1330	-0.8984	-5.5223

H	11.1946	-3.9781	-7.2670
H	12.6711	-3.1738	-6.7505
H	12.1057	-4.5936	-5.8837

Molecule 3 OPLS3e

OPLS3e energy = 63.374 kJ/mol

C	2.5298	-0.1694	0.9797
C	2.5697	0.5795	2.1614
C	1.8810	0.1115	3.2870
C	1.1620	-1.0897	3.2416
C	1.1346	-1.8247	2.0494
C	1.8159	-1.3664	0.9117
H	3.0487	0.1872	0.1012
C	3.3581	1.8877	2.2210
H	1.9024	0.6873	4.2035
C	0.4197	-1.5991	4.4777
H	0.5836	-2.7543	2.0193
N	1.8567	-2.0487	-0.3143
F	-0.8465	-1.1893	4.4215
F	0.4374	-2.9315	4.5350
F	0.9662	-1.1285	5.6005
F	3.8268	2.1142	3.4491
F	4.3919	1.8664	1.3772
F	2.5535	2.8929	1.8802
H	2.5060	-1.7070	-1.0210
C	1.0694	-3.0325	-0.7689
O	0.0822	-3.4752	-0.1955
N	1.3850	-3.4639	-1.9935
H	0.6153	-3.9642	-2.4075
C	4.3904	-5.7200	-2.9658

C	3.1297	-5.2664	-2.2046
C	2.7165	-3.8105	-2.5286
C	3.9114	-2.7992	-2.3812
C	5.1416	-3.2949	-3.1872
C	5.5544	-4.7302	-2.8075
H	4.1524	-5.8312	-4.0254
H	4.6944	-6.7107	-2.6230
H	3.3094	-5.3530	-1.1312
H	2.3098	-5.9515	-2.4284
H	2.5057	-3.8278	-3.5990
N	3.5604	-1.3770	-2.5693
H	4.1989	-2.8917	-1.3308
H	4.9335	-3.2394	-4.2558
H	5.9978	-2.6455	-3.0101
H	5.9077	-4.7473	-1.7746
H	6.3995	-5.0499	-3.4197
C	4.6331	-0.4533	-2.1887
C	3.0617	-1.0501	-3.9082
H	4.2655	0.5743	-2.1646
H	5.0166	-0.6804	-1.1923
H	5.4717	-0.4817	-2.8866
H	2.7508	-0.0050	-3.9613
H	3.8181	-1.2106	-4.6782
H	2.1855	-1.6466	-4.1645

Molecule 3 OPLS

OPLS energy = -173.441 kJ/mol

C	1.4555	-0.2586	1.8411
C	0.1050	0.1006	1.6788
C	-0.5186	0.9032	2.6533

C	0.1955	1.3294	3.7908
C	1.5386	0.9468	3.9476
C	2.1643	0.1400	2.9873
H	1.9613	-0.8585	1.0971
C	-0.6781	-0.3695	0.4607
H	-1.5521	1.1865	2.5175
C	-0.4554	2.1823	4.8699
H	2.0817	1.2559	4.8296
N	3.5184	-0.2652	3.1898
F	0.3302	3.2917	5.1084
F	-1.7274	2.6041	4.5380
F	-0.5220	1.4365	6.0289
F	-0.6534	0.6323	-0.4858
F	-0.1450	-1.5215	-0.0832
F	-1.9920	-0.6321	0.7963
H	4.2235	0.3700	3.5336
C	3.7125	-1.5453	3.5429
O	4.8001	-1.8626	4.0677
N	2.7387	-2.4397	3.3616
H	1.8273	-2.1127	3.0693
C	3.2870	-6.1108	4.1551
C	3.5484	-4.7936	3.4126
C	2.6311	-3.7225	4.0107
C	1.1752	-4.1290	3.7881
C	0.8832	-5.4025	4.5960
C	1.8078	-6.5176	4.0867
H	3.5736	-5.9931	5.2007
H	3.8957	-6.8988	3.7117
H	3.3487	-4.9174	2.3478
H	4.5922	-4.5123	3.5483

H	2.8468	-3.6083	5.0733
N	0.2623	-2.9631	3.9957
H	1.0857	-4.3841	2.7316
H	1.0716	-5.2370	5.6570
H	-0.1488	-5.7222	4.4590
H	1.5521	-6.7503	3.0525
H	1.6556	-7.4110	4.6925
C	0.0971	-2.5242	5.4037
C	-1.0731	-3.1742	3.3912
H	-0.4995	-1.6121	5.4265
H	-0.4123	-3.2871	5.9922
H	1.0599	-2.3007	5.8606
H	-1.6511	-2.2522	3.4583
H	-0.9731	-3.4315	2.3367
H	-1.6167	-3.9659	3.9056

Molecule 4 AMBER

AMBER energy = 116.39 kJ/mol

C	1.4953	-0.2425	0.4463
C	0.6974	0.8068	-0.0430
C	-0.6999	0.7715	0.1113
C	-1.3199	-0.3116	0.7583
C	-0.5220	-1.3579	1.2526
C	0.8753	-1.3219	1.1016
H	1.1633	1.6422	-0.5467
H	-1.3053	1.5810	-0.2701
H	-0.9882	-2.1941	1.7538
H	1.4795	-2.1291	1.4928
C	5.7821	-0.0972	0.0182
C	5.1278	0.7886	0.8829

C	3.7335	0.7250	1.0046
C	2.9856	-0.2111	0.2678
C	3.6309	-1.1006	-0.6280
C	5.0447	-1.0324	-0.7262
H	6.8586	-0.0652	-0.0757
H	5.6936	1.5089	1.4571
H	3.2256	1.3986	1.6800
N	2.7871	-1.9864	-1.3377
H	5.6279	-1.6887	-1.3502
H	1.8052	-2.0004	-1.1075
C	3.1985	-2.7774	-2.3569
O	4.3262	-2.7575	-2.8474
C	0.3704	-5.3234	-4.2426
C	1.2987	-4.6378	-5.0412
C	2.2338	-3.8039	-4.4132
C	2.2108	-3.6770	-3.0115
C	1.2402	-4.4357	-2.3073
N	0.3387	-5.2301	-2.9085
H	-0.3541	-5.9656	-4.7254
H	1.2911	-4.7515	-6.1170
H	2.9660	-3.2593	-4.9932

Molecule 4 MM2

MM2 energy = -84.009 kJ/mol

C	0.5328	0.0347	0.5816
C	0.4627	-0.3628	1.9189
C	-0.6835	-0.9774	2.4217
C	-1.7747	-1.2115	1.5851
C	-1.7111	-0.8239	0.2469
C	-0.5644	-0.2043	-0.2490

H	1.3139	-0.1832	2.5966
H	-0.7139	-1.2749	3.4836
H	-2.5638	-1.0044	-0.4293
H	-0.5298	0.0820	-1.3132
C	4.1390	1.7481	-1.0103
C	4.1915	0.5980	-0.2271
C	3.0148	0.0641	0.2940
C	1.7818	0.6727	0.0459
C	1.7283	1.8415	-0.7235
C	2.9120	2.3607	-1.2563
H	5.0658	2.1740	-1.4305
H	5.1592	0.1070	-0.0289
H	3.0701	-0.8605	0.8926
N	0.5972	2.5225	-0.9834
H	2.8879	3.2785	-1.8684
H	0.4184	2.7883	-1.9543
C	-0.3461	3.0534	-0.1177
O	-1.3142	3.6302	-0.5729
C	0.0982	2.7187	4.0488
C	1.2244	3.0907	3.3176
C	1.0936	3.2086	1.9354
C	-0.1502	2.9453	1.3582
C	-1.2180	2.5995	2.1871
N	-1.0912	2.4759	3.5076
H	0.1557	2.6073	5.1556
H	2.1881	3.2858	3.8171
H	1.9627	3.5135	1.3303

Molecule 4 MM3

MM3 energy = 85.843 kJ/mol

C	1.4220	0.9478	0.7866
C	0.2674	0.2210	1.1037
C	-0.8409	0.8554	1.6689
C	-0.8027	2.2240	1.9311
C	0.3437	2.9567	1.6271
C	1.4500	2.3211	1.0589
H	0.2245	-0.8612	0.8955
H	-1.7483	0.2756	1.9050
H	0.3753	4.0391	1.8344
H	2.3546	2.9093	0.8322
C	4.8552	-1.0501	-0.8047
C	4.2518	-1.5008	0.3654
C	3.1286	-0.8418	0.8609
C	2.5900	0.2687	0.1981
C	3.1930	0.7172	-0.9936
C	4.3291	0.0524	-1.4750
H	5.7466	-1.5658	-1.2003
H	4.6656	-2.3710	0.9026
H	2.6692	-1.1943	1.7996
N	2.7056	1.7976	-1.7202
H	4.8129	0.3904	-2.4074
H	3.4623	2.4736	-1.8833
C	1.4651	2.1299	-2.2359
O	1.2997	3.2051	-2.7626
C	-1.7546	-0.6332	-2.0168
C	-0.4772	-1.1110	-2.2969
C	0.5850	-0.2126	-2.3739
C	0.3465	1.1550	-2.1705
C	-0.9737	1.5524	-1.8920
N	-2.0005	0.6747	-1.8151

H	-2.6150	-1.3207	-1.9497
H	-0.3121	-2.1891	-2.4575
H	1.5969	-0.5834	-2.6065

Molecule 4 MMFF

MMFF energy = 241.934 kJ/mol

C	1.3273	-0.0426	0.0603
C	0.5194	0.9359	-0.5397
C	-0.8714	0.8873	-0.4131
C	-1.4643	-0.1439	0.3115
C	-0.6793	-1.1267	0.9100
C	0.7114	-1.0761	0.7840
H	0.9742	1.7489	-1.1036
H	-1.4819	1.6565	-0.8789
H	-1.1411	-1.9336	1.4728
H	1.3176	-1.8493	1.2535
C	5.5786	0.0644	-0.3823
C	4.7825	-0.5454	-1.3438
C	3.3994	-0.5664	-1.1806
C	2.7923	0.0216	-0.0540
C	3.6082	0.6448	0.9250
C	4.9963	0.6550	0.7431
H	6.6591	0.0864	-0.5025
H	5.2366	-1.0035	-2.2186
H	2.7858	-1.0485	-1.9398
N	2.9814	1.2408	2.0513
H	5.6633	1.1221	1.4604
H	1.9681	1.1949	2.0481
C	3.5780	1.8784	3.1206
O	4.7812	2.0115	3.3161

C	1.0436	3.3028	6.1931
C	2.2498	2.7165	6.5333
C	3.0705	2.2565	5.5063
C	2.6639	2.3882	4.1713
C	1.4454	3.0116	3.9389
N	0.6231	3.4602	4.9204
H	0.3661	3.6773	6.9562
H	2.5478	2.6211	7.5723
H	4.0273	1.7951	5.7452

Molecule 4 MMFF

MMFF energy = 241.937 kJ/mol

C	1.1742	-0.0451	0.5142
C	0.5081	-0.4194	-0.6638
C	-0.8818	-0.3134	-0.7604
C	-1.6156	0.1692	0.3208
C	-0.9720	0.5485	1.4963
C	0.4182	0.4435	1.5911
H	1.0744	-0.7960	-1.5141
H	-1.3827	-0.6065	-1.6793
H	-1.5429	0.9231	2.3419
H	0.9120	0.7374	2.5161
C	5.4339	-0.3032	0.7762
C	4.8199	0.8796	0.3835
C	3.4304	0.9420	0.3074
C	2.6352	-0.1767	0.6225
C	3.2655	-1.3824	1.0243
C	4.6631	-1.4252	1.0944
H	6.5179	-0.3602	0.8396
H	5.4204	1.7513	0.1373

H	2.9604	1.8737	-0.0029
N	2.4504	-2.4996	1.3468
H	5.1928	-2.3215	1.4004
H	1.4523	-2.3295	1.2866
C	2.8502	-3.7625	1.7358
O	4.0032	-4.1673	1.8374
C	-0.1781	-6.6400	2.4006
C	0.9909	-7.0267	1.7694
C	1.9762	-6.0642	1.5626
C	1.7667	-4.7441	1.9847
C	0.5677	-4.4675	2.6276
N	-0.4119	-5.3833	2.8335
H	-0.9787	-7.3517	2.5852
H	1.1354	-8.0528	1.4476
H	2.9079	-6.3420	1.0725

Molecule 4 OPLS-2005

OPLS-2005 energy = 241.937 kJ/mol

C	1.8995	-0.2911	0.6132
C	1.1528	0.4495	-0.3291
C	-0.2063	0.7404	-0.1003
C	-0.8309	0.2988	1.0807
C	-0.0941	-0.4328	2.0306
C	1.2644	-0.7242	1.7968
H	1.6262	0.7993	-1.2361
H	-0.7701	1.3049	-0.8289
H	-0.5729	-0.7703	2.9381
H	1.8228	-1.2896	2.5294
C	6.0629	-1.1421	-0.0565
C	5.6703	-0.2230	0.9312

C	4.3040	0.0391	1.1371
C	3.3163	-0.6099	0.3660
C	3.7116	-1.5523	-0.6199
C	5.0908	-1.8024	-0.8300
H	7.1112	-1.3433	-0.2240
H	6.4159	0.2844	1.5265
H	4.0089	0.7536	1.8921
N	2.6765	-2.1913	-1.3628
H	5.4360	-2.4952	-1.5820
H	1.7615	-1.7981	-1.2060
C	2.7296	-3.2290	-2.2172
O	3.7301	-3.9121	-2.4241
C	-0.9483	-4.4400	-4.0155
C	-0.1173	-5.4183	-3.4517
C	1.0917	-5.0099	-2.8629
C	1.4246	-3.6406	-2.8393
C	0.5285	-2.7369	-3.4670
N	-0.6348	-3.1327	-4.0236
H	-1.8858	-4.7139	-4.4766
H	-0.4000	-6.4609	-3.4751
H	1.7536	-5.7439	-2.4249

Molecule 4 OPLS3e

OPLS3e energy = 60.708 kJ/mol

C	1.1181	0.1384	0.4005
C	0.9109	0.1994	1.7827
C	-0.3568	0.4680	2.2941
C	-1.4239	0.6706	1.4232
C	-1.2344	0.6012	0.0459
C	0.0334	0.3307	-0.4626

H	1.7389	0.0504	2.4609
H	-0.5149	0.5215	3.3612
H	-2.0693	0.7518	-0.6230
H	0.1741	0.2595	-1.5323
C	5.0155	-0.7987	-1.1535
C	4.3624	-1.6710	-0.2890
C	3.1049	-1.3399	0.2094
C	2.4784	-0.1377	-0.1448
C	3.1489	0.7549	-1.0124
C	4.4154	0.4060	-1.5129
H	5.9899	-1.0509	-1.5465
H	4.8269	-2.6061	-0.0103
H	2.6020	-2.0342	0.8677
N	2.5126	1.9796	-1.3081
H	4.9614	1.0541	-2.1799
H	1.6152	2.0874	-0.8646
C	2.9090	3.0210	-2.0638
O	3.9746	3.1095	-2.6680
C	0.2204	6.3260	-2.1383
C	1.5094	6.5162	-1.6435
C	2.3807	5.4268	-1.6215
C	1.9476	4.1740	-2.0787
C	0.6381	4.0725	-2.5903
N	-0.2139	5.1308	-2.6071
H	-0.4890	7.1414	-2.1769
H	1.8309	7.4857	-1.2905
H	3.3911	5.5556	-1.2543

Molecule 4 OPLS

OPLS energy = 42.962 kJ/mol

C	1.4747	0.0007	-0.2590
C	0.4724	0.5563	-1.0695
C	-0.4295	1.4917	-0.5356
C	-0.3322	1.8798	0.8090
C	0.6728	1.3293	1.6178
C	1.5750	0.3936	1.0856
H	0.3923	0.2609	-2.1057
H	-1.2029	1.9165	-1.1582
H	0.7483	1.6277	2.6530
H	2.3471	-0.0294	1.7127
C	4.2478	-2.8379	-1.9149
C	4.3530	-1.4780	-2.2365
C	3.4399	-0.5634	-1.6931
C	2.4280	-1.0003	-0.8247
C	2.3190	-2.3642	-0.4898
C	3.2333	-3.2789	-1.0518
H	4.9445	-3.5497	-2.3331
H	5.1334	-1.1364	-2.9012
H	3.5181	0.4855	-1.9391
N	1.2379	-2.7633	0.3638
H	3.1626	-4.3347	-0.8352
H	0.3836	-2.2200	0.3770
C	1.4590	-3.6495	1.3596
O	2.5550	-4.1717	1.5538
C	-1.6429	-4.8909	3.9791
C	-0.4782	-5.6338	3.8976
C	0.5188	-5.2049	3.0359
C	0.3312	-4.0509	2.2774
C	-0.8899	-3.3686	2.4466
N	-1.8560	-3.7812	3.2751

H	-2.4208	-5.2265	4.6507
H	-0.3498	-6.5275	4.4915
H	1.4329	-5.7750	2.9578

Molecule 5 AMBER

AMBER energy = 42.962 kJ/mol

O	2.1188	-1.7431	-3.8212
C	0.9068	-1.3795	-4.4656
C	0.2188	-0.3664	-3.5232
H	0.3073	-2.2907	-4.4881
C	1.1667	-0.9602	-5.9118
H	-0.3557	-1.0053	-2.8501
C	-0.7810	0.6451	-4.1146
N	1.2115	0.3261	-2.6699
C	0.1567	-0.9890	-6.9035
C	2.4494	-0.5387	-6.3108
C	-0.6914	1.9385	-3.2880
H	-1.7926	0.2378	-4.0856
H	-0.5442	0.9023	-5.1457
C	1.7629	1.5159	-3.3599
C	0.5321	0.7720	-1.4318
C	-1.1623	-1.4246	-6.6335
C	0.4932	-0.5578	-8.2135
C	2.6676	-0.1416	-7.6381
H	3.2822	-0.5199	-5.6235
C	0.6700	2.5655	-3.6127
H	-1.4964	2.6263	-3.5542
C	-0.6931	1.6435	-1.7756
H	2.2011	1.2235	-4.3113
H	2.5540	1.9607	-2.7541

H	0.2264	-0.0921	-0.8393
H	1.2306	1.3535	-0.8274
C	-2.1395	-1.4263	-7.6416
H	-1.4646	-1.7724	-5.6597
C	-0.5011	-0.5655	-9.2084
N	1.7123	-0.1383	-8.5674
H	3.6586	0.1781	-7.9337
H	0.6998	2.8885	-4.6551
H	0.8335	3.4390	-2.9787
H	-0.6022	2.5918	-1.2413
C	-2.0104	1.0036	-1.3180
C	-1.8083	-0.9936	-8.9322
H	-0.2498	-0.2386	-10.2083
H	-2.1581	0.0198	-1.7610
H	-2.0068	0.9009	-0.2319
H	-2.8449	1.6470	-1.6005
H	-2.5528	-0.9939	-9.7175
H	2.4629	-2.5396	-4.2474
H	1.9454	-0.3534	-2.4923
H	-3.1463	-1.7644	-7.4314

Molecule 5 MM2

MM2 energy = 294.876 kJ/mol

O	-1.4599	-1.5934	-1.9328
C	-0.6026	-0.8035	-1.1389
C	-1.2290	0.6031	-1.1942
H	0.3624	-0.8007	-1.6990
C	-0.3476	-1.4510	0.2070
H	-0.9302	0.9988	-2.1970
C	-0.8770	1.6485	-0.1296

N	-2.7253	0.4446	-1.1851
C	0.7707	-1.1489	1.0050
C	-1.2192	-2.4381	0.6810
C	-2.1186	2.5009	0.1774
H	-0.0315	2.2881	-0.4763
H	-0.5424	1.1720	0.8184
C	-3.2944	0.2964	0.2001
C	-3.3509	1.6477	-1.8400
C	1.7446	-0.2177	0.6264
C	0.9224	-1.8097	2.2352
C	-0.9745	-3.0357	1.9145
H	-2.1045	-2.7538	0.1053
C	-3.0821	1.6078	0.9729
H	-1.8418	3.4002	0.7811
C	-2.8528	2.9139	-1.1112
H	-2.8139	-0.5428	0.7498
H	-4.3836	0.0483	0.1611
H	-3.0945	1.6949	-2.9263
H	-4.4663	1.5792	-1.8001
C	2.8366	0.0662	1.4444
H	1.6813	0.3266	-0.3287
C	2.0180	-1.5201	3.0520
N	0.0611	-2.7224	2.6803
H	-1.6610	-3.8151	2.3177
H	-2.6717	1.3986	1.9900
H	-4.0515	2.1370	1.1345
H	-3.7463	3.5174	-0.8132
C	-2.0089	3.8351	-2.0074
C	2.9754	-0.5859	2.6657
H	2.1362	-2.0369	4.0198

H	-1.1060	3.3253	-2.4105
H	-2.6024	4.1955	-2.8792
H	-1.6642	4.7328	-1.4445
H	3.8367	-0.3666	3.3193
H	-0.9943	-2.3873	-2.1819
H	-2.9322	-0.4265	-1.7456
H	3.5903	0.8067	1.1268

Molecule 5 MM3

MM3 energy = 211.955 kJ/mol

O	-0.2850	0.2045	-1.5614
C	0.1357	0.2226	-0.2014
C	-1.1276	-0.1976	0.5865
H	0.3939	1.2701	0.0708
C	1.3755	-0.6416	-0.0868
H	-1.7983	0.6983	0.5378
C	-0.9260	-0.5126	2.0891
N	-1.8959	-1.3282	-0.0471
C	2.3486	-0.4444	0.9204
C	1.6345	-1.6513	-1.0297
C	-1.8863	-1.6406	2.5116
H	-1.0915	0.4006	2.7038
H	0.1139	-0.8448	2.2911
C	-1.3309	-2.6776	0.2937
C	-3.3052	-1.2739	0.4647
C	2.2487	0.5770	1.8820
C	3.4660	-1.3066	0.9715
C	2.7677	-2.4515	-0.9185
H	0.9540	-1.8369	-1.8766
C	-1.3970	-2.9245	1.8214

H	-1.8700	-1.7718	3.6191
C	-3.3244	-1.3668	2.0200
H	-0.2805	-2.7769	-0.0472
H	-1.8969	-3.4804	-0.2335
H	-3.8096	-0.3376	0.1334
H	-3.9053	-2.1085	0.0335
C	3.2190	0.7294	2.8732
H	1.4106	1.2899	1.8835
C	4.4269	-1.1439	1.9716
N	3.6567	-2.2966	0.0751
H	2.9760	-3.2514	-1.6499
H	-0.3934	-3.2147	2.2085
H	-2.0843	-3.7708	2.0499
H	-3.9621	-2.2373	2.3119
C	-3.9383	-0.1129	2.6650
C	4.3094	-0.1335	2.9229
H	5.2927	-1.8264	1.9958
H	-5.0008	0.0189	2.3607
H	-3.9234	-0.1793	3.7757
H	-3.3998	0.8173	2.3821
H	5.0803	-0.0144	3.7042
H	0.3380	0.7202	-2.0713
H	-1.8917	-1.2011	-1.0684
H	3.1284	1.5372	3.6205

Molecule 5 MMFF

MMFF energy = 420.781 kJ/mol

O	-0.4989	-1.9626	-1.9790
C	-0.0570	-0.6826	-1.4691
C	-1.3241	-0.0201	-0.8429

H	0.2231	-0.1090	-2.3601
C	1.1741	-0.8572	-0.6020
H	-1.9124	0.2901	-1.7183
C	-1.2168	1.1852	0.0951
N	-2.2392	-1.0567	-0.1603
C	2.1185	0.1971	-0.4154
C	1.4242	-2.0904	0.0204
C	-2.3363	1.1120	1.1599
H	-1.2928	2.1128	-0.4835
H	-0.2533	1.2210	0.6101
C	-1.8542	-1.3526	1.2713
C	-3.6709	-0.5591	-0.2094
C	2.0421	1.4720	-1.0355
C	3.2137	-0.0535	0.4476
C	2.5327	-2.2242	0.8309
H	0.7740	-2.9497	-0.1139
C	-1.9858	-0.0669	2.0875
H	-2.3745	2.0429	1.7363
C	-3.7183	0.7998	0.5216
H	-0.8376	-1.7402	1.2830
H	-2.5234	-2.1390	1.6366
H	-3.9682	-0.5041	-1.2621
H	-4.2924	-1.3121	0.2874
C	2.9965	2.4607	-0.7927
H	1.2451	1.7247	-1.7284
C	4.1467	0.9477	0.6753
N	3.4006	-1.2275	1.0646
H	2.7604	-3.1622	1.3311
H	-1.0488	0.1284	2.6228
H	-2.7581	-0.1904	2.8564

H	-4.4582	0.7178	1.3296
C	-4.2048	1.9189	-0.3987
C	4.0496	2.1991	0.0686
H	4.9836	0.7446	1.3415
H	-5.2208	1.7089	-0.7501
H	-4.2266	2.8751	0.1343
H	-3.5680	2.0363	-1.2805
H	4.8040	2.9577	0.2650
H	0.2421	-2.3160	-2.5130
H	-2.1375	-1.9173	-0.7287
H	2.9230	3.4307	-1.2800

Molecule 5 OPLS-2005

OPLS-2005 energy = 420.781 kJ/mol

O	3.1795	3.1076	-5.7879
C	3.0995	2.2805	-4.6390
C	2.2511	1.0560	-5.0940
H	2.5341	2.8524	-3.9019
C	4.4838	1.9952	-4.0528
H	1.2532	1.4848	-5.2110
C	2.0765	-0.1634	-4.1438
N	2.6332	0.5644	-6.4535
C	4.6908	1.6752	-2.6828
C	5.6203	2.1130	-4.8844
C	1.9543	-1.4544	-4.9931
H	1.2042	-0.0262	-3.5029
H	2.9252	-0.2797	-3.4717
C	3.7578	-0.4196	-6.4228
C	1.4424	-0.0805	-7.0761
C	3.6262	1.6069	-1.7469

C	6.0238	1.4268	-2.2408
C	6.8914	1.8605	-4.3474
H	5.5551	2.4013	-5.9242
C	3.3434	-1.6834	-5.6245
H	1.6726	-2.3077	-4.3727
C	0.9508	-1.2502	-6.1649
H	4.6519	0.0144	-5.9851
H	4.0360	-0.6940	-7.4419
H	0.6537	0.6567	-7.2390
H	1.7031	-0.4555	-8.0678
C	3.8737	1.2723	-0.4031
H	2.6019	1.8040	-2.0165
C	6.2438	1.0880	-0.8942
N	7.0791	1.5017	-3.0710
H	7.7784	1.9378	-4.9603
H	4.0859	-1.9035	-4.8546
H	3.3251	-2.5587	-6.2768
H	0.9562	-2.1623	-6.7661
C	-0.4999	-1.0695	-5.6874
C	5.1844	1.0083	0.0258
H	7.2553	0.8980	-0.5662
H	-1.1886	-1.0220	-6.5323
H	-0.8146	-1.9103	-5.0669
H	-0.6383	-0.1607	-5.1019
H	5.3808	0.7542	1.0588
H	3.6033	3.9183	-5.5238
H	2.9046	1.4004	-6.9631
H	3.0598	1.2218	0.3096

Molecule 5 OPLS3e

OPLS3e energy = 450.901 kJ/mol

O	1.5147	3.3805	-3.2502
C	1.1310	2.2669	-2.4658
C	-0.3954	2.2935	-2.1261
H	1.7050	2.3738	-1.5444
C	1.5239	0.9397	-3.1261
H	-0.5975	1.3819	-1.5569
C	-1.3898	2.3481	-3.3293
N	-0.7575	3.4436	-1.1254
C	1.8011	-0.2223	-2.3272
C	1.5957	0.8227	-4.5213
C	-2.5215	3.3900	-3.1132
H	-1.7942	1.3549	-3.5306
H	-0.8455	2.5950	-4.2428
C	-0.8181	4.8367	-1.8238
C	-2.1823	3.1551	-0.5595
C	1.7429	-0.2544	-0.9023
C	2.1543	-1.4301	-3.0137
C	1.9477	-0.3953	-5.0916
H	1.3810	1.6643	-5.1661
C	-1.8426	4.7798	-2.9902
H	-3.2407	3.3742	-3.9379
C	-3.2092	3.0923	-1.7428
H	0.1830	5.1084	-2.1613
H	-1.1070	5.5699	-1.0664
H	-2.1280	2.2254	0.0139
H	-2.4170	3.9570	0.1462
C	2.0328	-1.4263	-0.2056
H	1.4804	0.6236	-0.3360
C	2.4414	-2.6023	-2.2750

N	2.2254	-1.5039	-4.3703
H	2.0152	-0.5063	-6.1655
H	-1.3330	5.0276	-3.9245
H	-2.5950	5.5613	-2.8634
H	-3.9526	3.8754	-1.5688
C	-4.0094	1.7714	-1.7519
C	2.3795	-2.5885	-0.8845
H	2.7111	-3.5065	-2.8036
H	-4.5815	1.6469	-0.8308
H	-4.7276	1.7458	-2.5735
H	-3.3686	0.8947	-1.8519
H	2.6045	-3.4879	-0.3266
H	2.4420	3.2620	-3.4860
H	-0.0768	3.4706	-0.3747
H	1.9941	-1.4388	0.8755

Molecule 5 OPLS

OPLS energy = 8.505 kJ/mol

O	0.8439	0.9508	0.3727
C	1.0448	0.0267	-0.7081
C	1.9842	-1.0420	-0.1249
H	1.5931	0.5732	-1.4743
C	-0.2845	-0.4417	-1.2967
H	2.9599	-0.6562	-0.4213
C	1.9977	-2.5199	-0.5544
N	1.9087	-0.9201	1.3447
C	-0.4009	-0.9575	-2.5957
C	-1.4417	-0.3410	-0.5283
C	2.3822	-3.3459	0.6909
H	2.7249	-2.6609	-1.3538

H	1.0249	-2.8597	-0.9062
C	0.8744	-1.8078	1.9345
C	3.2259	-1.3036	1.9012
C	0.7076	-1.0844	-3.4633
C	-1.6662	-1.3649	-3.0317
C	-2.6447	-0.7723	-1.0639
H	-1.4397	0.0650	0.4717
C	1.1720	-3.2836	1.6332
H	2.5814	-4.3814	0.4148
C	3.5751	-2.7471	1.4752
H	-0.1071	-1.5650	1.5359
H	0.8384	-1.6635	3.0145
H	3.9986	-0.6090	1.5696
H	3.1873	-1.2545	2.9898
C	0.5481	-1.6152	-4.7513
H	1.7044	-0.7866	-3.1835
C	-1.8166	-1.8956	-4.3217
N	-2.7667	-1.2827	-2.2848
H	-3.5388	-0.6911	-0.4604
H	0.3016	-3.7451	1.1659
H	1.3914	-3.8131	2.5608
H	3.7142	-3.3378	2.3812
C	4.8988	-2.8237	0.7039
C	-0.7188	-2.0230	-5.1824
H	-2.7968	-2.2075	-4.6528
H	5.0932	-3.8580	0.4199
H	4.8685	-2.2060	-0.1929
H	5.7084	-2.4755	1.3455
H	-0.8541	-2.4318	-6.1740
H	0.4120	1.7384	0.0069

H	1.6450	0.0614	1.4648
H	1.3970	-1.7092	-5.4147

Molecule 6 AMBER

AMBER energy = 154.271 kJ/mol

C	-0.3444	1.5048	1.0177
C	-1.0551	0.3991	0.7215
C	-0.4532	-0.7452	0.1527
C	0.8707	-0.7504	-0.1083
H	-0.8485	2.3525	1.4611
H	-2.1189	0.3838	0.9231
H	1.3144	-1.6407	-0.5325
C	1.6638	0.3754	0.1703
C	1.0342	1.5476	0.7505
C	1.8291	2.6732	1.0201
C	3.1462	2.6825	0.7113
C	3.7561	1.5344	0.1510
C	3.0411	0.4165	-0.0889
H	1.3668	3.5239	1.4980
O	3.9238	3.7975	0.8976
H	3.5363	-0.4529	-0.5000
C	7.9335	1.5231	-0.7925
C	7.4266	0.9760	0.3313
C	6.1063	0.9936	0.6086
C	5.2088	1.5629	-0.2206
H	9.0028	1.4721	-0.9522
H	8.1023	0.5074	1.0351
H	5.7620	0.5403	1.5292
C	5.7016	2.1775	-1.4589
C	7.1392	2.1220	-1.7026

C	7.6322	2.6696	-2.8316
C	6.8272	3.2618	-3.7371
C	5.4910	3.3444	-3.5668
C	4.8764	2.8398	-2.4756
H	8.6966	2.6360	-3.0278
H	7.2750	3.6819	-4.6293
H	4.9084	3.8330	-4.3368
C	3.4101	2.9802	-2.3521
N	2.6305	1.9772	-2.8142
H	3.0807	1.1563	-3.1975
N	2.8314	4.1515	-1.7710
H	1.6256	1.9766	-2.7124
H	1.8294	4.2159	-1.6741
C	3.6153	5.1862	-1.0968
C	3.1440	6.5661	-1.5669
C	3.4574	5.0727	0.4295
H	4.6764	5.1053	-1.3334
H	3.2808	6.6562	-2.6453
H	2.0903	6.7120	-1.3259
H	3.7313	7.3426	-1.0746
H	2.4274	5.2751	0.7202
H	4.0850	5.8343	0.8943
C	-2.8820	-4.2146	-0.7067
C	-2.7603	-3.7473	0.6094
C	-1.9635	-2.6275	0.8968
C	-1.2883	-1.9589	-0.1499
C	-1.4060	-2.4319	-1.4777
C	-2.2040	-3.5600	-1.7454
H	-3.4960	-5.0799	-0.9162
H	-3.2782	-4.2562	1.4111

O	-1.8478	-2.2109	2.1921
O	-0.7604	-1.8017	-2.5049
H	-2.3098	-3.9374	-2.7526
H	-1.1983	-1.5141	2.3127
H	-0.9481	-2.1982	-3.3587

Molecule 6 MM2

MM2 energy = 224.837 kJ/mol

C	2.1308	1.7775	4.4941
C	1.2158	1.4319	5.4870
C	-0.0341	2.0499	5.5447
C	-0.3514	3.0177	4.5900
H	3.1109	1.2713	4.4728
H	1.4875	0.6558	6.2221
H	-1.3330	3.5184	4.6330
C	0.5625	3.3693	3.5944
C	1.8128	2.7497	3.5435
C	2.7239	3.1085	2.5476
C	2.4379	4.0916	1.5942
C	1.1790	4.6961	1.6635
C	0.2509	4.3381	2.6409
H	3.6977	2.5946	2.5133
O	3.3123	4.4644	0.6125
H	-0.7300	4.8422	2.6593
C	-0.0214	7.5373	-1.2968
C	-0.4053	6.2027	-1.3797
C	0.0096	5.3149	-0.3929
C	0.8042	5.7516	0.6690
H	-0.3570	8.2323	-2.0852
H	-1.0314	5.8523	-2.2178

H	-0.2951	4.2567	-0.4599
C	1.1951	7.0956	0.7657
C	0.7701	7.9844	-0.2354
C	1.1281	9.3349	-0.1918
C	1.9123	9.8302	0.8455
C	2.3396	8.9634	1.8455
C	1.9842	7.6150	1.8023
H	0.7951	10.0334	-0.9784
H	2.1923	10.8969	0.8750
H	2.9635	9.3511	2.6688
C	2.4992	6.7837	2.9095
N	1.6763	6.6583	4.0397
H	1.9535	6.1208	4.8488
N	3.7250	6.2391	2.8757
H	0.7652	7.0994	4.0410
H	4.0267	5.6799	3.6771
C	4.5762	6.2464	1.6724
C	5.9915	6.6829	2.0833
C	4.6076	4.8593	1.0079
H	4.2072	6.9871	0.9236
H	5.9768	7.6964	2.5471
H	6.4334	5.9760	2.8227
H	6.6732	6.7247	1.2026
H	5.0737	4.0851	1.6582
H	5.2029	4.9058	0.0668
C	-2.8405	0.9792	8.5886
C	-3.2124	0.9374	7.2481
C	-2.3116	1.2851	6.2388
C	-1.0206	1.6778	6.6144
C	-0.6195	1.7298	7.9555

C	-1.5512	1.3736	8.9335
H	-3.5641	0.7014	9.3737
H	-4.2354	0.6208	6.9844
O	-2.6710	1.2272	4.9265
O	0.6366	2.1317	8.2960
H	-1.2671	1.4069	9.9987
H	-3.5831	0.9017	4.8366
H	0.7625	2.0631	9.2581

Molecule 6 MMFF

MMFF energy = 405.584 kJ/mol

C	-0.5049	1.5476	0.9996
C	-1.2254	0.3908	0.6926
C	-0.5812	-0.7389	0.1710
C	0.8065	-0.6856	-0.0303
H	-1.0414	2.4012	1.4103
H	-2.3016	0.3724	0.8634
H	1.3101	-1.5686	-0.4235
C	1.5430	0.4733	0.2617
C	0.8781	1.6064	0.7787
C	1.6121	2.7684	1.0568
C	2.9802	2.8280	0.7761
C	3.6627	1.7006	0.2871
C	2.9289	0.5254	0.0492
H	1.1124	3.6252	1.5029
O	3.6968	3.9801	0.9803
H	3.4504	-0.3612	-0.3127
C	7.8877	1.5700	-0.3145
C	7.3202	1.1837	0.8914
C	5.9451	1.2529	1.0513

C	5.1110	1.7138	0.0123
H	8.9705	1.5017	-0.4179
H	7.9493	0.8261	1.7034
H	5.5137	0.9418	2.0029
C	5.6822	2.1354	-1.2421
C	7.0973	2.0389	-1.3727
C	7.7403	2.4107	-2.5630
C	7.0259	2.8884	-3.6536
C	5.6493	3.0090	-3.5633
C	4.9711	2.6493	-2.3808
H	8.8241	2.3290	-2.6520
H	7.5492	3.1694	-4.5653
H	5.1001	3.3973	-4.4201
C	3.5146	2.8485	-2.3864
N	2.7747	1.8941	-2.9136
H	1.7650	1.9245	-2.9210
N	2.9499	3.9571	-1.9328
H	3.2200	1.0496	-3.2565
H	1.9408	4.0154	-1.9824
C	3.6602	5.0676	-1.2346
C	3.3022	6.3571	-1.9609
C	3.2147	5.1142	0.2408
H	4.7418	4.9155	-1.2833
H	3.8023	7.2164	-1.5032
H	3.6077	6.3033	-3.0112
H	2.2218	6.5370	-1.9409
H	2.1282	5.2311	0.3267
H	3.6591	5.9951	0.7183
C	-2.8304	-4.2844	-0.7557
C	-2.5920	-3.9368	0.5711

C	-1.8527	-2.7924	0.8709
C	-1.3398	-1.9601	-0.1418
C	-1.5933	-2.3387	-1.4747
C	-2.3295	-3.4874	-1.7827
H	-3.4054	-5.1785	-0.9855
H	-2.9773	-4.5586	1.3757
O	-1.6573	-2.5486	2.2052
O	-1.1138	-1.5608	-2.5010
H	-2.5237	-3.7776	-2.8111
H	-1.0435	-1.8025	2.3070
H	-1.4675	-1.9171	-3.3339

Molecule 6 OPLS-2005

OPLS-2005 energy = 405.584 kJ/mol

C	-0.4897	1.5125	0.9835
C	-1.1930	0.3271	0.6955
C	-0.5262	-0.8025	0.1711
C	0.8646	-0.7242	-0.0561
H	-1.0242	2.3613	1.3861
H	-2.2588	0.2827	0.8751
H	1.3773	-1.5884	-0.4543
C	1.5761	0.4601	0.2206
C	0.8959	1.5856	0.7401
C	1.6070	2.7721	1.0038
C	2.9895	2.8499	0.7240
C	3.6744	1.7238	0.2123
C	2.9614	0.5294	-0.0216
H	1.0882	3.6236	1.4191
O	3.6924	4.0174	0.8963
H	3.4842	-0.3371	-0.4012

C	7.9450	1.6712	-0.3362
C	7.3444	1.3060	0.8676
C	5.9530	1.3607	0.9719
C	5.1167	1.7829	-0.0850
H	9.0218	1.6139	-0.4179
H	7.9447	0.9748	1.7040
H	5.5019	1.0601	1.9080
C	5.7433	2.1904	-1.3172
C	7.1723	2.1048	-1.4292
C	7.8152	2.4481	-2.6337
C	7.0867	2.8759	-3.7443
C	5.6963	2.9834	-3.6420
C	4.9957	2.6715	-2.4531
H	8.8916	2.3751	-2.7092
H	7.5963	3.1316	-4.6638
H	5.1520	3.3416	-4.5064
C	3.5507	2.9150	-2.5231
N	2.7751	2.0262	-3.1464
H	1.7679	2.0766	-3.1548
N	2.9801	4.0302	-2.0347
H	3.2099	1.1839	-3.4987
H	1.9781	4.1321	-2.0649
C	3.6699	5.1076	-1.2883
C	3.4288	6.4512	-1.9890
C	3.2063	5.1518	0.1854
H	4.7478	4.9383	-1.2902
H	4.0687	7.2275	-1.5664
H	3.6539	6.3952	-3.0546
H	2.3965	6.7864	-1.8807
H	2.1244	5.2657	0.2580

H	3.6280	6.0335	0.6696
C	-2.7164	-4.4235	-0.6695
C	-2.6272	-3.9392	0.6469
C	-1.9072	-2.7617	0.9195
C	-1.2679	-2.0447	-0.1241
C	-1.3615	-2.5475	-1.4490
C	-2.0829	-3.7293	-1.7155
H	-3.2697	-5.3289	-0.8735
H	-3.1094	-4.4744	1.4529
O	-1.8285	-2.3331	2.2139
O	-0.7645	-1.8950	-2.4939
H	-2.1609	-4.1171	-2.7207
H	-1.2709	-1.5749	2.3092
H	-0.9678	-2.2947	-3.3260

Molecule 6 OPLS3e

OPLS3e energy = 10.908 kJ/mol

C	7.1356	5.9363	3.9582
C	7.6156	6.1606	5.2465
C	6.9864	5.6129	6.3670
C	5.8532	4.8160	6.1804
H	7.6502	6.3610	3.1080
H	8.5037	6.7616	5.3833
H	5.3677	4.3771	7.0400
C	5.3301	4.5715	4.8869
C	5.9813	5.1446	3.7566
C	5.4577	4.9017	2.4660
C	4.3007	4.1393	2.2984
C	3.6589	3.5720	3.4063
C	4.1817	3.7704	4.6859

H	5.9650	5.3088	1.6045
O	3.7585	3.9615	1.0462
H	3.7008	3.3171	5.5415
C	0.2543	0.9722	3.0474
C	1.5573	0.5106	2.9677
C	2.6153	1.3959	3.0727
C	2.4188	2.7666	3.2501
H	-0.5646	0.2689	2.9765
H	1.7550	-0.5443	2.8315
H	3.6236	1.0058	3.0178
C	1.0672	3.3017	3.2948
C	-0.0127	2.3522	3.2137
C	-1.3552	2.7853	3.2993
C	-1.6562	4.1270	3.4598
C	-0.6430	5.0682	3.5159
C	0.7100	4.7077	3.4215
H	-2.1528	2.0567	3.2408
H	-2.6901	4.4408	3.5241
H	-0.9393	6.1064	3.6115
C	1.6669	5.8037	3.4179
N	1.9411	6.3467	4.5821
H	2.7402	6.9439	4.7094
N	2.1982	6.2594	2.2885
H	1.4968	5.9234	5.3811
H	2.8884	6.9768	2.4181
C	2.0262	5.7251	0.9172
C	1.4460	6.8174	0.0043
C	3.3418	5.1326	0.3420
H	1.3048	4.9020	0.9431
H	2.1202	7.6686	-0.1035

H	1.2527	6.4311	-0.9985
H	0.4940	7.1941	0.3823
H	4.1335	5.8817	0.2862
H	3.1720	4.8171	-0.6892
C	8.5221	6.3377	10.3351
C	7.1877	6.6018	10.0432
C	6.6898	6.3701	8.7632
C	7.5181	5.8670	7.7422
C	8.8650	5.6086	8.0600
C	9.3587	5.8411	9.3410
H	8.9081	6.5175	11.3289
H	6.5493	6.9890	10.8233
O	5.3705	6.6667	8.5445
O	9.7374	5.1090	7.1321
H	10.3931	5.6385	9.5768
H	4.9784	7.0415	9.3350
H	10.5956	4.9140	7.5120

Molecule 6 OPLS

OPLS energy = 201.102 kJ/mol

C	0.1560	0.9792	-1.0656
C	-0.1687	-0.2425	-1.5325
C	0.7667	-1.3004	-1.5449
C	2.0151	-1.1165	-1.0705
H	-0.5854	1.7657	-1.0700
H	-1.1674	-0.4156	-1.9108
H	2.7154	-1.9397	-1.0806
C	2.4066	0.1396	-0.5881
C	1.4522	1.2255	-0.5910
C	1.8585	2.4860	-0.1287

C	3.1345	2.6879	0.2724
C	4.0586	1.6165	0.2965
C	3.7002	0.3807	-0.1063
H	1.1348	3.2861	-0.1048
O	3.5948	3.9348	0.6143
H	4.4196	-0.4250	-0.0701
C	8.0175	2.1170	1.8203
C	7.0079	1.5769	2.5312
C	5.7657	1.4361	2.0250
C	5.4586	1.8323	0.7752
H	8.9928	2.2038	2.2783
H	7.2011	1.2456	3.5421
H	4.9980	0.9952	2.6458
C	6.5142	2.4301	-0.0432
C	7.8265	2.5468	0.5612
C	8.8370	3.0831	-0.1447
C	8.6522	3.5146	-1.4080
C	7.4557	3.4370	-2.0260
C	6.3726	2.9232	-1.4127
H	9.8169	3.1718	0.3040
H	9.4940	3.9389	-1.9385
H	7.3690	3.8042	-3.0385
C	5.1011	2.8922	-2.1681
N	4.8397	1.7716	-2.8830
H	5.5087	1.0121	-2.8233
N	4.2377	4.0364	-2.2372
H	3.9996	1.6149	-3.4228
H	3.4253	4.0338	-2.8342
C	4.3300	5.1980	-1.3551
C	4.0533	6.4593	-2.1734

C	3.2978	5.0796	-0.2224
H	5.3191	5.3083	-0.9149
H	4.7960	6.5428	-2.9671
H	3.0589	6.4042	-2.6171
H	4.1161	7.3385	-1.5320
H	2.2950	5.0548	-0.6449
H	3.3760	5.9680	0.4043
C	-0.4037	-5.1424	-3.1089
C	-0.9701	-4.6649	-1.9174
C	-0.5851	-3.4179	-1.4061
C	0.3687	-2.6377	-2.0897
C	0.9404	-3.1209	-3.2864
C	0.5509	-4.3725	-3.7917
H	-0.7040	-6.1049	-3.4977
H	-1.7033	-5.2559	-1.3871
O	-1.1437	-2.9787	-0.2410
O	1.8722	-2.3839	-3.9676
H	0.9784	-4.7552	-4.7066
H	-0.7859	-2.1363	0.0513
H	2.1412	-2.8116	-4.7849

Molecule 7 AMBER

AMBER energy = -73.318 kJ/mol

C	0.1980	-0.0684	3.5369
C	0.5259	0.0995	2.1808
C	-0.4931	0.2765	1.2279
C	-1.8441	0.2876	1.6202
C	-2.1648	0.1179	2.9795
C	-1.1494	-0.0595	3.9362
H	0.9802	-0.2056	4.2709

H	1.5610	0.0915	1.8691
H	-0.2377	0.4033	0.1853
C	-2.8561	0.4658	0.6698
H	-3.2001	0.1222	3.2895
H	-1.4059	-0.1901	4.9781
C	-3.7257	0.6217	-0.1505
C	-4.7605	0.8128	-1.1329
O	-5.5630	-0.0731	-1.3801
C	-4.8238	2.1580	-1.8499
H	-3.8086	2.5293	-1.9953
H	-5.3694	2.8654	-1.2256
C	-5.5141	2.0395	-3.2127
H	-5.5144	3.0132	-3.7024
H	-6.5468	1.7194	-3.0657
C	-4.7909	1.0379	-4.1074
O	-3.6520	0.6807	-3.9651
O	-5.6086	0.6367	-5.0841
C	-5.0426	-0.3424	-5.9631
H	-4.7613	-1.2383	-5.4068
H	-5.7883	-0.6152	-6.7095
H	-4.1671	0.0616	-6.4742

Molecule 7 MM2

MM2 energy = -21.521 kJ/mol

C	0.3433	-0.4093	-0.6555
C	-0.4403	-1.5615	-0.6107
C	-1.6945	-1.5302	-0.0014
C	-2.1683	-0.3457	0.5650
C	-1.3822	0.8070	0.5191
C	-0.1283	0.7747	-0.0904

H	1.3353	-0.4345	-1.1376
H	-0.0677	-2.4987	-1.0577
H	-2.3056	-2.4487	0.0279
C	-3.3508	-0.3145	1.1399
H	-1.7449	1.7501	0.9630
H	0.4914	1.6868	-0.1256
C	-4.4472	-0.2813	1.6744
C	-5.6271	-0.2382	2.2520
O	-6.5550	-0.8228	1.7417
C	-5.7813	0.5559	3.5385
H	-5.7818	-0.1724	4.3839
H	-4.9058	1.2319	3.6881
C	-7.0861	1.3675	3.5599
H	-7.9630	0.6812	3.4878
H	-7.1690	1.9220	4.5245
C	-7.1397	2.3816	2.4339
O	-6.1821	2.7422	1.7896
O	-8.4015	2.8139	2.2575
C	-8.6086	3.7535	1.2315
H	-8.3256	3.3053	0.2508
H	-8.0293	4.6804	1.4504
H	-9.6958	3.9916	1.2269

Molecule 7 MM3

MM3 energy = -16.366 kJ/mol

C	0.0055	0.1243	-2.1566
C	0.2079	1.1502	-1.2314
C	-0.8230	2.0480	-0.9439
C	-2.0571	1.9161	-1.5851
C	-2.2597	0.8903	-2.5102

C	-1.2283	-0.0053	-2.7958
H	0.8205	-0.5839	-2.3826
H	1.1847	1.2487	-0.7288
H	-2.8748	2.6222	-1.3619
H	-3.2350	0.7879	-3.0155
H	-1.3884	-0.8158	-3.5268
C	-0.4611	3.9051	0.7289
C	-0.6339	3.0148	-0.0727
C	-0.2687	4.8709	1.6006
O	-1.1916	5.6226	1.8173
C	1.0840	4.9910	2.2812
H	1.3030	4.0398	2.8195
H	1.8707	5.1157	1.5014
C	1.1418	6.1629	3.2627
H	0.9239	7.1145	2.7250
H	0.3562	6.0384	4.0434
C	2.4957	6.2823	3.9431
O	3.4106	5.5231	3.7174
O	2.6161	7.2984	4.8243
C	3.8661	7.4443	5.4816
H	3.8166	8.3137	6.1699
H	4.1033	6.5403	6.0819
H	4.6781	7.6300	4.7468

Molecule 7 MMFF

MMFF energy = -20.789 kJ/mol

C	0.7908	-0.6738	-0.5870
C	0.5959	0.6538	-0.2060
C	-0.6415	1.0621	0.2959
C	-1.6850	0.1414	0.4166

C	-1.4878	-1.1880	0.0358
C	-0.2498	-1.5947	-0.4661
H	1.7549	-0.9913	-0.9762
H	1.4077	1.3707	-0.2978
H	-0.7832	2.0984	0.5933
C	-2.9534	0.5595	0.9281
H	-2.2924	-1.9134	0.1294
H	-0.0969	-2.6298	-0.7604
C	-4.0186	0.9132	1.3562
C	-5.2996	1.3446	1.8661
O	-5.7756	0.7955	2.8518
C	-5.9975	2.4296	1.0781
H	-5.2556	3.1483	0.7111
H	-6.4710	1.9565	0.2104
C	-7.0567	3.1345	1.9182
H	-7.6339	3.8144	1.2823
H	-7.7402	2.4056	2.3673
C	-6.4239	3.9558	3.0150
O	-5.2345	4.2350	3.0799
O	-7.3795	4.3676	3.8926
C	-6.8741	5.1456	4.9783
H	-7.7131	5.4015	5.6313
H	-6.4273	6.0736	4.6081
H	-6.1470	4.5689	5.5587

Molecule 7 OPLS-2005

OPLS-2005 energy = -20.789 kJ/mol

C	3.6246	-0.0343	-1.4138
C	2.6983	0.7687	-2.1078
C	1.3925	0.9343	-1.6046

C	1.0112	0.2968	-0.4060
C	1.9393	-0.5072	0.2872
C	3.2450	-0.6723	-0.2166
H	4.6258	-0.1619	-1.8004
H	2.9881	1.2572	-3.0268
H	0.6855	1.5503	-2.1421
C	-0.3342	0.4684	0.1135
H	1.6536	-1.0006	1.2053
H	3.9544	-1.2896	0.3155
C	-1.4688	0.6029	0.5450
C	-2.8161	0.7596	1.1120
O	-3.8249	0.6232	0.4214
C	-2.8796	1.1252	2.5958
H	-2.1452	0.5373	3.1477
H	-2.5725	2.1663	2.6884
C	-4.2899	0.9584	3.1997
H	-4.3921	1.6159	4.0621
H	-5.0614	1.2594	2.4902
C	-4.5737	-0.4660	3.6732
O	-3.7038	-1.3377	3.6978
O	-5.8438	-0.6276	4.0586
C	-6.2675	-1.9017	4.5102
H	-6.1396	-2.6524	3.7291
H	-7.3224	-1.8685	4.7817
H	-5.6984	-2.2124	5.3874

Molecule 7 OPLS3e

OPLS3e energy = -16.31 kJ/mol

C	1.3840	-0.8504	-0.0986
C	1.1678	0.3198	-0.8251

C	-0.0943	0.9106	-0.8221
C	-1.1325	0.3271	-0.0927
C	-0.9120	-0.8444	0.6348
C	0.3502	-1.4350	0.6315
H	2.3634	-1.3078	-0.1001
H	1.9742	0.7686	-1.3870
H	-0.2631	1.8194	-1.3828
C	-2.4508	0.9437	-0.0900
H	-1.7144	-1.2948	1.2024
H	0.5255	-2.3407	1.1940
C	-3.5526	1.4716	-0.0808
C	-4.9557	2.1748	-0.1147
O	-5.4591	2.6927	0.8806
C	-5.6933	2.1605	-1.4598
H	-4.9847	2.5638	-2.1843
H	-5.8657	1.1361	-1.7856
C	-7.0064	2.9996	-1.5365
H	-7.6443	2.6566	-2.3491
H	-7.5919	2.8151	-0.6348
C	-6.8186	4.5166	-1.6129
O	-5.7420	5.0240	-1.9271
O	-7.9438	5.1823	-1.3083
C	-7.9461	6.6029	-1.3020
H	-8.9353	6.9730	-1.0326
H	-7.6898	7.0014	-2.2851
H	-7.2292	6.9925	-0.5769

Molecule 7 OPLS

OPLS energy = -72.817 kJ/mol

C	-0.0303	-3.8675	0.0786

C	-1.1202	-3.7544	-0.7966
C	-1.9128	-2.5964	-0.7873
C	-1.6214	-1.5458	0.0959
C	-0.5297	-1.6618	0.9699
C	0.2640	-2.8190	0.9621
H	0.5808	-4.7587	0.0717
H	-1.3500	-4.5593	-1.4796
H	-2.7510	-2.5127	-1.4638
C	-2.3952	-0.3804	0.1275
H	-0.2988	-0.8556	1.6517
H	1.1031	-2.9012	1.6375
C	-3.0575	0.6311	0.1638
C	-3.8331	1.8434	0.2230
O	-4.0642	2.4900	-0.7858
C	-4.3515	2.2985	1.5844
H	-5.4409	2.2627	1.5743
H	-3.9883	1.6233	2.3602
C	-3.9106	3.7286	1.9095
H	-4.3458	4.4111	1.1787
H	-4.2795	3.9963	2.8996
C	-2.3919	3.8749	1.8932
O	-1.6109	3.0257	2.2300
O	-2.0557	5.0974	1.4508
C	-0.6445	5.3615	1.3509
H	-0.1748	4.6597	0.6604
H	-0.1733	5.2875	2.3318
H	-0.5049	6.3733	0.9706

Molecule 8 AMBER

AMBER energy = -33.309 kJ/mol

N	1.4903	10.3165	0.5433
C	2.5901	8.7687	2.1441
N	3.6935	8.7300	1.1924
C	2.7109	10.5034	-0.2282
H	2.2756	7.7361	2.3014
C	3.1028	9.3181	3.4862
H	2.4599	10.3437	-1.2778
C	3.2112	11.9462	-0.0820
O	0.2899	9.4694	2.2049
C	1.3704	9.5504	1.6278
O	4.8228	9.4462	-0.5845
C	3.8209	9.5067	0.1177
H	0.6584	10.7890	0.2304
H	4.4456	8.0851	1.3719
H	2.4450	12.6289	-0.4491
H	4.0855	12.0639	-0.7254
C	4.3101	13.0499	3.9715
C	3.0008	13.2693	3.5124
C	2.6475	12.9152	2.1987
C	3.5959	12.3386	1.3311
C	4.9067	12.1242	1.8006
C	5.2635	12.4769	3.1136
H	4.5833	13.3223	4.9821
H	2.2644	13.7094	4.1707
H	1.6349	13.0850	1.8610
H	5.6508	11.6816	1.1514
H	6.2729	12.3081	3.4624
H	3.1383	10.4040	3.4414
H	4.1189	8.9556	3.6461
C	2.3044	8.9180	4.7042

C	2.6958	8.4926	5.9498
N	1.5794	8.2489	6.7329
C	0.5277	8.5274	5.9679
N	0.9156	8.9308	4.7464
H	3.7193	8.3673	6.2719
H	-0.5011	8.4367	6.2856
H	0.3034	9.1888	3.9751

Molecule 8 MM2

MM2 energy = 124.208 kJ/mol

N	-4.2142	3.4454	-3.9836
C	-2.9451	2.9103	-1.8663
N	-1.7681	2.6645	-2.6997
C	-3.1559	2.8556	-4.8057
H	-3.2722	1.9085	-1.4989
C	-2.5489	3.7756	-0.6612
H	-3.5395	1.8613	-5.1372
C	-2.9101	3.7363	-6.0447
O	-5.1044	3.9803	-2.0109
C	-4.1596	3.4863	-2.6012
O	-0.8385	2.1984	-4.6680
C	-1.8423	2.5539	-4.0773
H	-5.0884	3.8071	-4.3685
H	-0.8578	2.3919	-2.3246
H	-3.8465	3.7984	-6.6477
H	-2.1582	3.2645	-6.7199
C	-1.6415	7.7299	-4.9987
C	-2.9974	7.4372	-5.1324
C	-3.4032	6.1461	-5.4669
C	-2.4634	5.1331	-5.6745

C	-1.1067	5.4379	-5.5376
C	-0.6958	6.7265	-5.1996
H	-1.3186	8.7502	-4.7313
H	-3.7497	8.2276	-4.9708
H	-4.4799	5.9321	-5.5683
H	-0.3441	4.6587	-5.6987
H	0.3784	6.9531	-5.0925
H	-1.6772	3.3222	-0.1335
H	-3.3796	3.8375	0.0802
C	-2.1970	5.1786	-1.0937
C	-0.9895	5.7629	-1.1346
N	-1.1045	7.0254	-1.5713
C	-2.3603	7.1921	-1.7858
N	-3.0636	6.0937	-1.5241
H	-0.0134	5.3071	-0.8521
H	-2.8016	8.1472	-2.1510
H	-4.0803	5.9847	-1.6072

Molecule 8 MM3

MM3 energy = 263.842 kJ/mol

N	0.0711	5.2700	-2.0346
C	-1.6022	4.0019	-3.4153
N	-1.8219	5.2644	-4.1137
C	0.2460	6.2614	-3.0918
H	-2.5901	3.6172	-3.0616
C	-0.9921	2.9444	-4.3434
H	0.3344	7.2700	-2.6186
C	1.5308	6.0111	-3.8971
O	-0.7163	3.2929	-1.3093
C	-0.7256	4.1498	-2.1624

O	-1.1230	7.3036	-4.7534
C	-0.9525	6.3352	-4.0499
H	0.6395	5.3333	-1.1787
H	-2.6128	5.3511	-4.7680
H	1.6561	6.8060	-4.6676
H	1.4441	5.0546	-4.4592
C	5.0410	5.9038	-1.3850
C	4.5577	7.1214	-1.8623
C	3.4246	7.1537	-2.6757
C	2.7607	5.9699	-3.0201
C	3.2573	4.7520	-2.5390
C	4.3897	4.7188	-1.7248
H	5.9372	5.8778	-0.7424
H	5.0731	8.0601	-1.5972
H	3.0536	8.1237	-3.0477
H	2.7512	3.8068	-2.7990
H	4.7710	3.7538	-1.3500
H	-0.8862	1.9734	-3.8080
H	0.0320	3.2519	-4.6516
C	-1.8420	2.7427	-5.5634
C	-1.5684	3.0138	-6.8399
N	-2.6171	2.6875	-7.5952
C	-3.6020	2.2489	-6.7827
N	-3.0968	2.2903	-5.5424
H	-0.6357	3.4222	-7.2515
H	-4.5131	1.7374	-7.1254
H	-3.3638	1.5246	-4.9077

Molecule 8 MMFF

MMFF energy = 353.286 kJ/mol

N	1.0478	1.3407	-1.2028
C	-0.6287	0.0858	-2.5143
N	-1.0710	-0.3336	-1.1812
C	1.2341	0.1136	-0.4234
H	-0.0304	-0.7381	-2.9246
C	-1.8226	0.4084	-3.4257
H	1.8237	-0.5758	-1.0412
C	1.9285	0.3702	0.9214
O	0.4932	2.1590	-3.2382
C	0.3095	1.3125	-2.3666
O	-0.3909	-1.3164	0.7384
C	-0.1389	-0.5523	-0.1871
H	1.7978	2.0235	-1.2164
H	-1.8826	-0.9344	-1.0936
H	1.9909	-0.5582	1.5042
H	1.3250	1.0586	1.5281
C	5.9114	1.9566	0.4312
C	5.6642	0.6066	0.1904
C	4.3784	0.0903	0.3567
C	3.3271	0.9227	0.7647
C	3.5876	2.2782	1.0093
C	4.8743	2.7917	0.8418
H	6.9139	2.3570	0.3039
H	6.4754	-0.0456	-0.1235
H	4.2062	-0.9677	0.1711
H	2.7913	2.9446	1.3347
H	5.0692	3.8435	1.0355
H	-2.2091	1.4139	-3.2151
H	-2.6463	-0.2850	-3.2096
C	-1.4962	0.2917	-4.8765

C	-1.8636	-0.6212	-5.8410
N	-1.2957	-0.3110	-7.0487
C	-0.5985	0.7814	-6.8369
N	-0.6941	1.1913	-5.5392
H	-2.5037	-1.4876	-5.7320
H	-0.0096	1.3129	-7.5722
H	-0.2194	1.9866	-5.1207

Molecule 8 MMFF

MMFF energy = 363.883 kJ/mol

N	0.9022	-0.5690	0.3486
C	-1.5371	-0.8137	0.1767
N	-1.4650	-1.0187	1.6216
C	0.8802	-1.6944	1.2903
H	-1.5752	-1.8133	-0.2761
C	-2.7830	-0.0329	-0.2646
H	0.7437	-2.6055	0.6943
C	2.1606	-1.7776	2.1341
O	-0.1505	0.5771	-1.2988
C	-0.2286	-0.1632	-0.3220
O	-0.4142	-2.0240	3.3521
C	-0.3513	-1.5683	2.2153
H	1.7842	-0.2398	-0.0270
H	-2.2951	-0.9586	2.1991
H	2.0870	-2.5965	2.8617
H	2.2728	-0.8623	2.7306
C	5.6926	-2.3971	-0.2672
C	4.7915	-3.4416	-0.0704
C	3.6527	-3.2450	0.7119
C	3.4045	-1.9986	1.3033

C	4.3208	-0.9566	1.1043
C	5.4585	-1.1559	0.3212
H	6.5808	-2.5522	-0.8743
H	4.9788	-4.4118	-0.5235
H	2.9652	-4.0747	0.8612
H	4.1584	0.0178	1.5607
H	6.1656	-0.3435	0.1733
H	-3.6775	-0.6009	0.0228
H	-2.8041	0.0445	-1.3592
C	-2.8848	1.3366	0.3192
C	-3.6664	1.8668	1.3223
N	-3.3979	3.1989	1.4979
C	-2.4712	3.4882	0.6139
N	-2.1325	2.3966	-0.1313
H	-4.4087	1.3638	1.9287
H	-2.0122	4.4568	0.4681
H	-1.4286	2.3622	-0.8617

Molecule 8 OPLS-2005

OPLS-2005 energy = 363.883 kJ/mol

N	0.2134	-1.7106	-6.9921
C	-0.2794	-1.0398	-4.6637
N	-1.1883	-2.1911	-4.6079
C	-0.9095	-2.6550	-7.0201
H	-0.9262	-0.1620	-4.6841
C	0.5486	-0.9428	-3.3636
H	-1.6967	-2.1612	-7.5910
C	-0.5111	-3.9162	-7.8207
O	1.5229	-0.1872	-6.0250
C	0.5500	-0.9313	-5.9609

O	-2.4051	-3.8193	-5.5276
C	-1.5649	-2.9360	-5.6515
H	0.8130	-1.6745	-7.7991
H	-1.5787	-2.4456	-3.7157
H	-0.3792	-3.6541	-8.8708
H	-1.3296	-4.6383	-7.8084
C	3.0978	-5.8618	-6.3722
C	1.8822	-6.0286	-5.6822
C	0.7097	-5.4128	-6.1597
C	0.7479	-4.6163	-7.3245
C	1.9687	-4.4557	-8.0156
C	3.1400	-5.0773	-7.5405
H	3.9953	-6.3380	-6.0047
H	1.8475	-6.6241	-4.7808
H	-0.2158	-5.5476	-5.6172
H	2.0161	-3.8541	-8.9116
H	4.0721	-4.9527	-8.0725
H	1.1500	-0.0324	-3.3703
H	-0.1208	-0.8473	-2.5083
C	1.4722	-2.1136	-3.1133
C	1.4538	-3.1070	-2.1595
N	2.5114	-3.9913	-2.3805
C	3.1316	-3.5099	-3.4499
N	2.5674	-2.3933	-3.9145
H	0.7604	-3.2650	-1.3455
H	3.9987	-3.9780	-3.8954
H	2.8687	-1.8670	-4.7261

Molecule 8 OPLS3e

OPLS3e energy = 119.228 kJ/mol

N	-5.4456	5.1771	3.2330
C	-5.2888	2.9971	4.2862
N	-6.2921	2.4746	3.3214
C	-6.8318	4.7958	2.8415
H	-5.8518	3.3195	5.1646
C	-4.2591	1.9281	4.7486
H	-7.4487	4.9428	3.7299
C	-7.4172	5.6577	1.6912
O	-3.3825	4.5644	3.8417
C	-4.5835	4.3141	3.7963
O	-7.6372	2.8010	1.5671
C	-6.9938	3.2720	2.4973
H	-5.0436	6.0305	2.8764
H	-6.3038	1.4946	3.0831
H	-8.4760	5.4332	1.5442
H	-6.9467	5.3891	0.7426
C	-6.9738	9.8904	2.3264
C	-6.0781	9.2212	1.4947
C	-6.2235	7.8524	1.2844
C	-7.2635	7.1581	1.9087
C	-8.1600	7.8347	2.7394
C	-8.0144	9.2037	2.9490
H	-6.8616	10.9530	2.4882
H	-5.2760	9.7622	1.0132
H	-5.5290	7.3366	0.6363
H	-8.9683	7.3046	3.2223
H	-8.7059	9.7315	3.5901
H	-4.7874	1.1738	5.3327
H	-3.5505	2.3705	5.4522
C	-3.5079	1.2425	3.6213

C	-3.5005	-0.0560	3.1231
N	-2.6745	-0.2132	2.1018
C	-2.1412	0.9693	1.9259
N	-2.6072	1.8935	2.8221
H	-4.0867	-0.8841	3.4947
H	-1.4156	1.2466	1.1754
H	-2.3618	2.8764	2.8888

Molecule 8 OPLS

OPLS energy = -44.575 kJ/mol

N	-1.6484	-0.6396	0.7500
C	-0.3137	0.7389	2.3066
N	0.8144	-0.0990	1.9321
C	-0.5941	-1.6311	0.5916
H	-0.5788	0.4691	3.3290
C	0.1179	2.2146	2.3317
H	-0.8934	-2.5158	1.1537
C	-0.5055	-2.0400	-0.8891
O	-2.4513	1.3323	1.3652
C	-1.5622	0.4901	1.4473
O	1.7849	-1.8037	0.9063
C	0.7604	-1.1829	1.1619
H	-2.4760	-0.7551	0.1932
H	1.7291	0.1647	2.2544
H	-1.4470	-2.5257	-1.1459
H	0.2944	-2.7699	-1.0138
C	0.1077	1.2266	-3.6708
C	-1.1834	0.7318	-3.4395
C	-1.3806	-0.3280	-2.5415
C	-0.2873	-0.8978	-1.8671

C	1.0053	-0.4052	-2.1092
C	1.2011	0.6572	-3.0033
H	0.2627	2.0453	-4.3590
H	-2.0269	1.1668	-3.9558
H	-2.3793	-0.7028	-2.3750
H	1.8563	-0.8378	-1.6021
H	2.1971	1.0410	-3.1727
H	0.9952	2.2943	2.9740
H	-0.6761	2.8259	2.7611
C	0.4783	2.7655	0.9646
C	1.7231	3.1018	0.5006
N	1.5904	3.5803	-0.7950
C	0.2879	3.5325	-1.0777
N	-0.4266	3.0591	-0.0430
H	2.6506	3.0094	1.0470
H	-0.1076	3.8446	-2.0329
H	-1.4276	2.9096	-0.0056

Molecule 9 AMBER

AMBER energy = 2.626 kJ/mol

C	-1.8541	-7.0881	2.2386
C	-1.1310	-5.8293	1.7301
C	-0.7244	-5.9781	0.2580
C	0.1863	-7.1975	0.0960
C	-0.5194	-8.4583	0.6030
C	-0.9201	-8.2930	2.0722
H	-2.7490	-7.3054	1.6590
N	-2.1827	-6.9267	3.6477
H	-0.2101	-5.7180	2.3053
N	-1.9047	-4.6101	1.9531

H	-0.1860	-5.0861	-0.0660
H	-1.5974	-6.1109	-0.3806
H	0.4357	-7.3227	-0.9590
H	1.1103	-7.0438	0.6557
H	-1.4073	-8.6472	-0.0026
H	0.1559	-9.3101	0.5061
H	-0.0196	-8.1574	2.6735
H	-1.4300	-9.1995	2.4025
H	-1.4454	-7.1446	4.3018
H	-1.4076	-3.8616	2.4179
N	-4.4733	-6.1103	3.3638
C	-3.4382	-6.4803	4.1630
C	-3.5972	-6.4336	5.5611
C	-4.8121	-6.0163	6.1183
H	-4.9330	-5.9801	7.1932
N	-3.7887	-5.2848	0.6924
C	-3.1165	-4.2904	1.2843
C	-3.5569	-2.9571	1.2928
C	-4.7452	-2.6327	0.6259
H	-5.1039	-1.6133	0.6119
C	-5.4396	-3.6569	-0.0347
C	-4.9065	-4.9672	0.0206
C	-5.5905	-6.0030	-0.6437
C	-6.7845	-5.7514	-1.3402
C	-7.3098	-4.4508	-1.3830
C	-6.6355	-3.4053	-0.7325
H	-5.1940	-7.0081	-0.6316
H	-7.2955	-6.5563	-1.8520
H	-8.2258	-4.2519	-1.9248
H	-7.0425	-2.4044	-0.7788

C	-5.8661	-5.6539	5.2683
C	-5.6694	-5.7151	3.8682
C	-6.7340	-5.3606	3.0141
C	-7.9678	-4.9422	3.5381
C	-8.1537	-4.8769	4.9268
C	-7.1050	-5.2341	5.7885
H	-6.6364	-5.4095	1.9422
H	-8.7778	-4.6725	2.8725
H	-9.1046	-4.5555	5.3329
H	-7.2644	-5.1824	6.8573
H	-2.9900	-2.1824	1.7905
H	-2.7909	-6.7186	6.2239
N	-4.3460	-6.0747	2.3571

Molecule 9 MM2

MM2 energy = 31.153 kJ/mol

C	1.3927	-0.6282	3.3231
C	1.3562	0.9160	3.3253
C	2.7669	1.5238	3.4092
C	3.5360	0.9778	4.6213
C	3.5837	-0.5564	4.5952
C	2.1655	-1.1438	4.5514
H	1.9364	-1.0209	2.4350
N	0.0214	-1.1632	3.3598
H	0.8285	1.2045	4.2662
N	0.5538	1.5418	2.2560
H	2.6995	2.6363	3.4770
H	3.3422	1.2864	2.4843
H	3.0406	1.3181	5.5618
H	4.5734	1.3909	4.6314

H	4.1587	-0.8977	3.7016
H	4.1236	-0.9343	5.4967
H	1.6247	-0.8644	5.4866
H	2.2181	-2.2590	4.5261
H	-0.4819	-1.1092	4.2511
H	-0.0009	2.3654	2.5095
N	-0.2296	-1.4640	0.9653
C	-0.7391	-1.3597	2.2698
C	-2.1234	-1.4801	2.4248
C	-2.9750	-1.6740	1.3367
H	-4.0593	-1.7546	1.5240
N	1.7137	0.5336	0.6150
C	0.9002	1.5222	0.9658
C	0.4144	2.4779	0.0718
C	0.8302	2.4143	-1.2568
H	0.4641	3.1568	-1.9857
C	1.7246	1.4106	-1.6313
C	2.1419	0.5069	-0.6486
C	3.0402	-0.5061	-0.9914
C	3.5167	-0.6196	-2.2964
C	3.0954	0.2821	-3.2718
C	2.1997	1.2969	-2.9389
H	3.3749	-1.2303	-0.2290
H	4.2242	-1.4245	-2.5579
H	3.4703	0.1924	-4.3054
H	1.8732	2.0059	-3.7184
C	-2.4879	-1.7620	0.0315
C	-1.1128	-1.6616	-0.1840
C	-0.5915	-1.7479	-1.4742
C	-1.4450	-1.9312	-2.5604

C	-2.8196	-2.0309	-2.3519
C	-3.3390	-1.9479	-1.0603
H	0.4953	-1.6726	-1.6396
H	-1.0333	-1.9978	-3.5817
H	-3.4969	-2.1767	-3.2103
H	-4.4293	-2.0298	-0.9139
H	-0.2769	3.2746	0.3942
H	-2.5713	-1.4132	3.4309
N	0.7627	-1.3947	0.7968

Molecule 9 MMFF

MMFF energy = 122.011 kJ/mol

C	0.2649	-0.9579	0.1527
C	0.6475	0.2405	1.0829
C	1.4031	-0.2754	2.3136
C	0.5743	-1.3093	3.0747
C	0.1240	-2.4597	2.1765
C	-0.5968	-1.9634	0.9239
H	1.1888	-1.4689	-0.1563
N	-0.4963	-0.4760	-1.0314
H	-0.2812	0.7180	1.4310
N	1.4823	1.2117	0.3277
H	1.6475	0.5590	2.9820
H	2.3582	-0.7231	2.0099
H	1.1594	-1.7049	3.9126
H	-0.3080	-0.8201	3.5057
H	0.9991	-3.0513	1.8804
H	-0.5362	-3.1289	2.7397
H	-1.5516	-1.5024	1.2082
H	-0.8423	-2.8176	0.2812

H	-0.8135	0.4744	-0.8159
H	2.3815	1.3608	0.7908
N	0.7815	0.7125	-2.6218
C	0.3004	-0.4553	-2.1841
C	0.6269	-1.6199	-2.8774
C	1.4246	-1.5429	-4.0166
H	1.6673	-2.4540	-4.5623
N	0.0902	2.5995	-0.9326
C	0.8471	2.4531	0.1842
C	0.9460	3.4266	1.1656
C	0.2524	4.6181	0.9925
H	0.3245	5.3943	1.7520
C	-0.5371	4.8037	-0.1455
C	-0.5964	3.7564	-1.0878
C	-1.3936	3.9360	-2.2122
C	-2.1144	5.1160	-2.4216
C	-2.0448	6.1455	-1.4910
C	-1.2586	5.9912	-0.3525
H	-1.4744	3.1442	-2.9520
H	-2.7334	5.2288	-3.3094
H	-2.6051	7.0650	-1.6479
H	-1.2156	6.8021	0.3725
C	1.9029	-0.3000	-4.4477
C	1.5599	0.8513	-3.7034
C	2.0319	2.1111	-4.0881
C	2.8384	2.2350	-5.2197
C	3.1758	1.1049	-5.9645
C	2.7136	-0.1572	-5.5833
H	1.7806	3.0026	-3.5184
H	3.2075	3.2129	-5.5257

H	3.8055	1.2065	-6.8479
H	2.9953	-1.0236	-6.1808
H	1.5463	3.2755	2.0569
H	0.2438	-2.5808	-2.5424
N	0.5797	1.5326	-2.0412

Molecule 9 MMFF

MMFF energy = 145.944 kJ/mol

C	0.6910	0.1290	0.5355
C	-0.6388	-0.5903	0.1536
C	-1.8818	0.2190	0.5660
C	-1.8449	0.6194	2.0361
C	-0.5682	1.3818	2.3644
C	0.6667	0.5600	2.0163
H	0.8347	1.0417	-0.0514
N	1.8503	-0.7558	0.3685
H	-0.6653	-1.5299	0.7237
N	-0.7178	-0.9908	-1.2667
H	-2.7889	-0.3663	0.3709
H	-1.9648	1.1320	-0.0367
H	-2.7162	1.2414	2.2700
H	-1.9133	-0.2770	2.6644
H	-0.5488	2.3291	1.8120
H	-0.5540	1.6323	3.4311
H	0.7003	-0.3253	2.6657
H	1.5682	1.1420	2.2454
H	1.9757	-1.4584	1.0932
H	-1.1048	-1.9146	-1.4390
N	2.2224	-0.4242	-1.9580
C	2.5933	-0.9320	-0.7749

C	3.8138	-1.6140	-0.7018
C	4.5998	-1.7797	-1.8364
H	5.5465	-2.3130	-1.7605
N	-0.1152	1.0104	-2.2906
C	-0.9585	-0.0453	-2.2439
C	-2.0336	-0.1779	-3.1063
C	-2.2857	0.8336	-4.0267
H	-3.1350	0.7436	-4.7009
C	-1.4678	1.9656	-4.0538
C	-0.3910	2.0195	-3.1474
C	0.4207	3.1471	-3.1621
C	0.1932	4.2038	-4.0487
C	-0.8676	4.1405	-4.9444
C	-1.6997	3.0239	-4.9482
H	1.2503	3.2207	-2.4633
H	0.8407	5.0780	-4.0345
H	-1.0522	4.9620	-5.6336
H	-2.5323	2.9879	-5.6485
C	4.1623	-1.2620	-3.0539
C	2.9294	-0.5713	-3.0950
C	2.4673	-0.0618	-4.3136
C	3.2210	-0.2193	-5.4787
C	4.4404	-0.8903	-5.4360
C	4.9111	-1.4120	-4.2309
H	1.5177	0.4559	-4.3857
H	2.8566	0.1807	-6.4239
H	5.0265	-1.0113	-6.3463
H	5.8656	-1.9372	-4.2234
H	-2.6940	-1.0378	-3.0557
H	4.1628	-2.0138	0.2480

N	1.3311	0.0783	-1.9941

Molecule 9 OPLS-2005

OPLS-2005 energy = 145.944 kJ/mol

C	-8.0960	1.1889	-3.9428
C	-8.6504	2.6217	-3.7688
C	-8.8481	3.2854	-5.1441
C	-7.5390	3.2996	-5.9493
C	-6.9597	1.8845	-6.1040
C	-6.7751	1.2082	-4.7362
H	-8.8296	0.6155	-4.5135
N	-7.9179	0.5758	-2.6372
H	-7.9128	3.2203	-3.2283
N	-9.9048	2.6247	-3.0163
H	-9.2025	4.3103	-5.0215
H	-9.6149	2.7576	-5.7138
H	-7.7121	3.7396	-6.9328
H	-6.8101	3.9430	-5.4534
H	-7.6229	1.2833	-6.7282
H	-6.0047	1.9308	-6.6299
H	-6.0090	1.7419	-4.1708
H	-6.4002	0.1944	-4.8796
H	-8.1833	1.2659	-1.9282
H	-10.6947	2.9776	-3.5399
N	-8.3063	-1.6631	-3.3179
C	-8.5463	-0.6559	-2.4474
C	-9.4111	-0.8835	-1.3593
C	-10.0169	-2.1475	-1.2044
H	-10.6844	-2.3295	-0.3755
N	-8.8873	2.5867	-0.9077

C	-9.8834	3.0540	-1.6874
C	-10.9017	3.8909	-1.2167
C	-10.8703	4.2990	0.1283
H	-11.6362	4.9524	0.5208
C	-9.8211	3.8422	0.9474
C	-8.8379	2.9877	0.3862
C	-7.7940	2.5297	1.2140
C	-7.7191	2.9149	2.5673
C	-8.7008	3.7637	3.1106
C	-9.7538	4.2291	2.3014
H	-7.0291	1.8882	0.8068
H	-6.9070	2.5648	3.1899
H	-8.6425	4.0629	4.1499
H	-10.5021	4.8838	2.7257
C	-9.7393	-3.1655	-2.1407
C	-8.8563	-2.8893	-3.2134
C	-8.5537	-3.8836	-4.1605
C	-9.1349	-5.1632	-4.0415
C	-10.0159	-5.4426	-2.9769
C	-10.3194	-4.4462	-2.0256
H	-7.8810	-3.6927	-4.9836
H	-8.9054	-5.9331	-4.7668
H	-10.4578	-6.4278	-2.8921
H	-10.9949	-4.6767	-1.2142
H	-11.6983	4.2386	-1.8599
H	-9.6195	-0.0947	-0.6480
N	-7.6605	-1.4952	-4.0871

Molecule 9 OPLS3e

OPLS3e energy = 43.021 kJ/mol

C	0.5699	1.4332	0.3081
C	-0.2284	0.0941	0.2693
C	-1.5963	0.1888	0.9829
C	-1.4431	0.7072	2.4252
C	-0.7006	2.0568	2.4573
C	0.6695	1.9897	1.7490
H	0.0726	2.1999	-0.2851
N	1.9434	1.2542	-0.2018
H	0.3353	-0.6613	0.8225
N	-0.3998	-0.4644	-1.0802
H	-2.0833	-0.7878	0.9949
H	-2.2727	0.8503	0.4405
H	-2.4259	0.8116	2.8898
H	-0.9041	-0.0261	3.0288
H	-1.3200	2.8202	1.9815
H	-0.5707	2.3836	3.4912
H	1.3379	1.3696	2.3503
H	1.1078	2.9897	1.7347
H	2.6213	0.8766	0.4424
H	-0.3877	-1.4724	-1.1287
N	1.4797	1.8420	-2.4840
C	2.3462	1.4327	-1.5467
C	3.6699	1.1982	-1.9079
C	4.0899	1.3888	-3.2279
H	5.1174	1.2101	-3.5196
N	-1.5412	1.4145	-2.0078
C	-1.1707	0.1135	-2.0974
C	-1.4969	-0.6856	-3.1881
C	-2.2667	-0.1572	-4.2242
H	-2.5438	-0.7615	-5.0774

C	-2.6993	1.1882	-4.1439
C	-2.3134	1.9442	-2.9951
C	-2.7232	3.2923	-2.8840
C	-3.4985	3.8727	-3.8871
C	-3.8743	3.1361	-5.0076
C	-3.4853	1.8047	-5.1480
H	-2.4403	3.8606	-2.0097
H	-3.8209	4.9015	-3.7937
H	-4.4836	3.6019	-5.7710
H	-3.7879	1.2359	-6.0165
C	3.1544	1.8268	-4.2010
C	1.8030	2.0582	-3.8009
C	0.8353	2.4945	-4.7280
C	1.2099	2.6983	-6.0564
C	2.5247	2.4756	-6.4656
C	3.4986	2.0451	-5.5606
H	-0.1807	2.6744	-4.4117
H	0.4782	3.0349	-6.7818
H	2.7883	2.6429	-7.5034
H	4.5146	1.8784	-5.8954
H	-1.1706	-1.7140	-3.2321
H	4.3822	0.8698	-1.1638
N	0.5096	1.9161	-2.2048

Molecule 9 OPLS

OPLS energy = -69.576 kJ/mol

C	3.3893	-3.4542	-2.5012
C	2.0559	-2.6903	-2.4970
C	2.2468	-1.2125	-2.1226
C	2.9278	-1.1025	-0.7565

C	4.2679	-1.8429	-0.7732
C	4.0578	-3.3174	-1.1293
H	4.0817	-3.0538	-3.2386
N	3.1372	-4.8598	-2.7598
H	1.4343	-3.1362	-1.7199
N	1.3056	-2.8427	-3.7446
H	1.2742	-0.7222	-2.0787
H	2.8684	-0.7023	-2.8573
H	3.1001	-0.0514	-0.5251
H	2.2828	-1.5331	0.0101
H	4.9306	-1.3817	-1.5062
H	4.7274	-1.7744	0.2128
H	3.4337	-3.7873	-0.3683
H	5.0248	-3.8203	-1.1515
H	2.9481	-5.4287	-1.9468
H	0.3305	-3.0768	-3.6007
N	3.3995	-4.7982	-5.1832
C	3.2245	-5.4905	-4.0364
C	3.1337	-6.8901	-4.0693
C	3.2383	-7.5669	-5.2854
H	3.1737	-8.6454	-5.3248
N	2.9052	-1.9317	-5.2364
C	1.6233	-2.1877	-4.9680
C	0.5803	-1.8546	-5.8208
C	0.8711	-1.1798	-6.9964
H	0.0822	-0.8958	-7.6773
C	2.1928	-0.8615	-7.2671
C	3.1722	-1.2485	-6.3554
C	4.5145	-0.9289	-6.6162
C	4.8706	-0.2339	-7.7823

C	3.8793	0.1476	-8.6973
C	2.5376	-0.1665	-8.4370
H	5.2828	-1.2128	-5.9126
H	5.9058	0.0122	-7.9735
H	4.1473	0.6866	-9.5959
H	1.7734	0.1327	-9.1400
C	3.4350	-6.8216	-6.4486
C	3.5145	-5.4221	-6.3730
C	3.7127	-4.6758	-7.5469
C	3.8273	-5.3210	-8.7852
C	3.7450	-6.7176	-8.8529
C	3.5498	-7.4685	-7.6864
H	3.7876	-3.6019	-7.5236
H	3.9814	-4.7446	-9.6872
H	3.8342	-7.2177	-9.8079
H	3.4889	-8.5459	-7.7497
H	-0.4448	-2.0908	-5.5753
H	2.9908	-7.4614	-3.1628
N	3.3817	-3.7742	-5.1683

Molecule 10 AMBER

AMBER energy = 69.095 kJ/mol

C	-1.4826	-2.8999	-3.9390
C	-1.5925	-4.3006	-3.8841
C	-1.1774	-4.9929	-2.7344
C	-0.6559	-4.2809	-1.6415
C	-0.5479	-2.8801	-1.7012
C	-0.9557	-2.1757	-2.8507
H	-1.8094	-2.3759	-4.8257
H	-1.9997	-4.8444	-4.7254

H	-1.2634	-6.0702	-2.6888
H	-0.3403	-4.8091	-0.7524
H	-0.1496	-2.3418	-0.8529
C	-0.8312	-0.6646	-2.9176
N	-2.1357	-0.0895	-3.1995
H	-0.5062	-0.3079	-1.9388
C	0.1815	-0.1607	-3.9573
H	0.0685	-0.7731	-4.8535
N	-0.1318	1.2149	-4.3056
C	4.3225	-0.4085	-2.6618
C	3.8690	-1.2030	-3.7279
C	2.5296	-1.1189	-4.1480
C	1.6289	-0.2449	-3.5087
C	2.0963	0.5514	-2.4438
C	3.4346	0.4708	-2.0201
H	5.3532	-0.4704	-2.3393
H	4.5502	-1.8772	-4.2283
H	2.1925	-1.7312	-4.9722
H	1.4223	1.2346	-1.9473
H	3.7810	1.0881	-1.2027
H	-2.3887	-0.0010	-4.1787
H	-0.4477	1.8124	-3.5481
S	-2.7655	1.0045	-2.1934
O	-1.8384	2.1223	-2.3047
C	-4.4281	1.4368	-2.8528
O	-2.8555	0.3409	-0.9070
S	-0.6970	1.5619	-5.7774
O	-1.9804	0.8737	-5.8048
C	-0.9077	3.3885	-5.8479
O	0.3227	1.1043	-6.7015

F	-4.3024	1.9631	-4.1253
F	-5.2173	0.3045	-2.9423
F	-5.0568	2.3674	-2.0469
F	0.2971	4.0198	-5.5968
F	-1.3820	3.7877	-7.0833
F	-1.8043	3.7921	-4.8757

Molecule 10 MM2

MM2 energy = 614.696 kJ/mol

C	-0.2525	-1.8019	-2.3650
C	-1.1839	-0.9017	-2.8806
C	-1.6640	0.1383	-2.0874
C	-1.2088	0.2719	-0.7773
C	-0.2769	-0.6306	-0.2671
C	0.2153	-1.6754	-1.0544
H	0.1029	-2.6210	-3.0105
H	-1.5451	-1.0150	-3.9168
H	-2.4027	0.8499	-2.4932
H	-1.5886	1.0907	-0.1432
H	0.0662	-0.5136	0.7743
C	1.2117	-2.6576	-0.4701
N	2.2156	-3.0855	-1.4636
H	1.7543	-2.1544	0.3656
C	0.4555	-3.8547	0.1635
H	-0.1909	-3.4254	0.9658
N	-0.4241	-4.5420	-0.8019
C	3.3167	-6.5157	2.0169
C	2.7322	-5.4855	2.7505
C	1.8060	-4.6369	2.1460
C	1.4485	-4.8041	0.8051

C	2.0453	-5.8378	0.0784
C	2.9715	-6.6898	0.6783
H	4.0512	-7.1876	2.4921
H	3.0049	-5.3401	3.8095
H	1.3594	-3.8225	2.7401
H	1.7980	-5.9914	-0.9841
H	3.4355	-7.5006	0.0916
H	1.9404	-3.6942	-2.2381
H	-0.0434	-4.8796	-1.6891
S	3.6707	-2.2996	-1.6592
O	3.2297	-0.9648	-1.5701
C	4.7712	-2.8359	-0.3134
O	4.0134	-2.8233	-2.9237
S	-1.8948	-5.1904	-0.3663
O	-1.5381	-5.7731	0.8655
C	-3.0875	-3.8214	-0.2512
O	-2.0862	-6.0253	-1.4875
F	6.0278	-2.2393	-0.4021
F	4.2634	-2.5189	0.9466
F	4.9777	-4.2151	-0.3237
F	-4.3607	-4.2707	0.0954
F	-2.7203	-2.8757	0.7062
F	-3.2163	-3.1359	-1.4587

Molecule 10 MMFF

MMFF energy = 889.652 kJ/mol

C	0.3746	0.3582	0.3975
C	-0.6936	1.1871	0.7440
C	-2.0038	0.7483	0.5688
C	-2.2487	-0.5198	0.0475

C	-1.1830	-1.3521	-0.2989
C	0.1441	-0.9228	-0.1299
H	1.3893	0.7223	0.5503
H	-0.5029	2.1771	1.1512
H	-2.8348	1.3961	0.8362
H	-3.2716	-0.8605	-0.0933
H	-1.4017	-2.3372	-0.7070
C	1.3206	-1.8266	-0.4776
N	1.1305	-2.4077	-1.8339
H	2.2118	-1.1869	-0.5184
C	1.5304	-2.9334	0.6308
H	0.5498	-3.2640	0.9972
N	2.2360	-4.1349	0.1095
C	3.6316	-1.2782	4.0400
C	2.2569	-1.4942	4.0996
C	1.5851	-2.0358	3.0026
C	2.2823	-2.3687	1.8298
C	3.6683	-2.1431	1.7841
C	4.3370	-1.6026	2.8837
H	4.1541	-0.8593	4.8963
H	1.7066	-1.2406	5.0025
H	0.5093	-2.1887	3.0723
H	4.2434	-2.3906	0.8937
H	5.4109	-1.4380	2.8389
H	0.5274	-3.2402	-1.8933
H	3.0292	-3.9597	-0.5233
S	2.4696	-2.4905	-2.8546
O	2.0609	-3.3848	-3.9204
C	2.6867	-0.7466	-3.5130
O	3.5767	-2.8696	-1.9886

S	1.3044	-5.4612	-0.3540
O	2.2139	-6.2985	-1.1121
C	0.8269	-6.3044	1.2531
O	0.1412	-4.8880	-1.0155
F	3.7470	-0.6496	-4.3615
F	1.5873	-0.3098	-4.1869
F	2.9080	0.1409	-2.4997
F	1.9144	-6.7003	1.9702
F	0.1069	-5.4675	2.0560
F	0.0575	-7.4076	1.0417

Molecule 10 OPLS-2005

OPLS-2005 energy = 889.652 kJ/mol

C	0.9337	-0.1891	-2.7512
C	0.1065	0.8898	-3.1188
C	-0.4482	1.7219	-2.1280
C	-0.1742	1.4755	-0.7693
C	0.6543	0.3980	-0.4010
C	1.2130	-0.4424	-1.3891
H	1.3542	-0.8184	-3.5222
H	-0.1018	1.0808	-4.1617
H	-1.0827	2.5499	-2.4097
H	-0.6009	2.1128	-0.0082
H	0.8488	0.2162	0.6466
C	2.0925	-1.6212	-0.9872
N	3.2891	-1.7025	-1.8264
H	2.4197	-1.4133	0.0347
C	1.3096	-2.9645	-0.9572
H	0.8872	-3.1164	-1.9536
N	2.1842	-4.1026	-0.6698

C	-2.0821	-2.8148	1.7643
C	-0.7906	-3.0695	2.2632
C	0.3072	-3.1241	1.3827
C	0.1203	-2.9253	-0.0040
C	-1.1787	-2.6720	-0.4972
C	-2.2756	-2.6159	0.3842
H	-2.9245	-2.7731	2.4396
H	-0.6427	-3.2249	3.3223
H	1.2925	-3.3223	1.7792
H	-1.3416	-2.5089	-1.5532
H	-3.2664	-2.4187	0.0007
H	3.2619	-2.5206	-2.4475
H	3.0861	-3.7994	-0.2817
S	4.7633	-1.8389	-1.0040
O	4.6590	-3.1259	-0.2956
C	6.0238	-1.9420	-2.3496
O	4.9292	-0.6015	-0.2333
S	2.5941	-5.0754	-1.9941
O	3.3539	-4.1744	-2.8773
C	3.7034	-6.3674	-1.2800
O	1.3448	-5.6749	-2.4762
F	5.4376	-1.7425	-3.5299
F	6.9518	-1.0075	-2.1482
F	6.5874	-3.1492	-2.3255
F	3.2327	-7.5712	-1.6032
F	4.9286	-6.2134	-1.7808
F	3.7406	-6.2436	0.0466

Molecule 10 OPLS3e

OPLS3e energy = 238.123 kJ/mol

C	1.0585	-0.5200	1.6277
C	1.7401	0.1951	2.6085
C	2.9851	0.7471	2.3215
C	3.5511	0.5947	1.0593
C	2.8673	-0.1164	0.0774
C	1.6175	-0.6890	0.3507
H	0.0913	-0.9323	1.8769
H	1.3016	0.3232	3.5880
H	3.5144	1.3013	3.0836
H	4.5179	1.0266	0.8423
H	3.3272	-0.2209	-0.8949
C	0.9077	-1.4863	-0.7634
N	-0.4101	-2.0319	-0.3350
H	0.7126	-0.7553	-1.5510
C	1.7942	-2.6284	-1.3508
H	2.6506	-2.1680	-1.8464
N	2.3140	-3.4972	-0.2597
C	-0.2390	-4.8156	-4.4776
C	-0.1273	-5.3899	-3.2142
C	0.5309	-4.7019	-2.1990
C	1.0842	-3.4364	-2.4467
C	0.9632	-2.8714	-3.7210
C	0.3033	-3.5594	-4.7354
H	-0.7506	-5.3501	-5.2656
H	-0.5485	-6.3671	-3.0235
H	0.6086	-5.1650	-1.2253
H	1.3773	-1.8958	-3.9345
H	0.2110	-3.1204	-5.7188
H	-0.5694	-2.9908	-0.6497
H	2.1177	-3.1101	0.6664

S	-1.8550	-1.0688	-0.5934
O	-1.5119	0.3158	-0.2521
C	-2.2076	-1.1969	-2.4764
O	-2.9193	-1.7659	0.1355
S	3.9987	-3.9832	-0.3008
O	4.3251	-4.3370	1.0840
C	4.0424	-5.5612	-1.3935
O	4.7361	-2.9009	-0.9646
F	-3.3405	-0.5454	-2.7451
F	-1.1920	-0.6466	-3.1458
F	-2.3297	-2.4865	-2.7982
F	3.1763	-6.4493	-0.8990
F	3.7062	-5.2308	-2.6421
F	5.2795	-6.0614	-1.3701

Molecule 11 AMBER

AMBER energy = 42.122 kJ/mol

C	0.8608	0.1334	-0.6654
C	0.8049	0.7434	0.5474
C	-0.3941	1.0899	1.1008
C	-1.5652	0.8275	0.4766
C	2.1249	1.0551	1.2581
H	-2.4835	1.1226	0.9644
C	-1.6246	0.2619	-0.7404
C	-0.3824	-0.1022	-1.3962
C	-0.4487	-0.6407	-2.6978
C	-1.6403	-0.8418	-3.2935
C	-2.8448	-0.5121	-2.6422
C	-2.8410	0.0264	-1.4075
H	0.4506	-0.8938	-3.2399

H	-1.6716	-1.2602	-4.2902
H	-3.7861	-0.6839	-3.1458
H	-3.7803	0.2848	-0.9392
C	4.6934	-0.8349	-2.3091
C	2.2039	-0.2372	-1.2488
C	3.1657	0.7142	-1.3631
C	4.3941	0.4097	-1.8727
H	5.6816	-1.0145	-2.7082
C	2.8440	2.1278	-0.8730
C	3.8282	-1.8586	-2.2162
C	2.5169	-1.6083	-1.6459
C	1.6371	-2.6998	-1.4936
C	1.9991	-3.9310	-1.9040
C	3.2619	-4.1636	-2.4826
C	4.1519	-3.1635	-2.6314
H	0.6649	-2.5696	-1.0412
H	1.3091	-4.7548	-1.7833
H	3.5248	-5.1628	-2.8015
H	5.1201	-3.3757	-3.0628
H	3.5162	2.8513	-1.3213
H	1.8618	2.4284	-1.2399
N	2.8290	2.1698	0.6002
H	2.7684	0.1747	1.2349
H	1.9611	1.2274	2.3160
N	4.2269	4.0840	0.8284
C	3.4552	3.2134	1.3543
N	3.2353	3.3391	2.7396
H	4.4044	3.8737	-0.1391
H	3.6998	4.0779	3.2445
H	2.5763	2.7464	3.2190

C	-0.5936	3.1452	4.9213
C	-0.0163	3.7675	3.7984
C	0.0439	3.1025	2.5622
C	-0.4642	1.7988	2.4239
C	-1.0476	1.1766	3.5425
C	-1.1102	1.8431	4.7790
H	0.4024	4.7601	3.8814
H	0.5036	3.5916	1.7129
H	-1.4472	0.1766	3.4533
H	-1.5696	1.3511	5.6240
C	7.5056	3.4293	-2.0590
C	7.0568	2.9321	-0.8210
C	6.0557	1.9481	-0.7647
C	5.4762	1.4478	-1.9438
C	5.9290	1.9345	-3.1830
C	6.9337	2.9167	-3.2393
H	7.4861	3.2999	0.1001
H	5.7188	1.5798	0.1960
H	5.4934	1.5612	-4.0988
H	7.2553	3.2923	-4.1998
C	-0.7720	5.1993	8.7318
C	-1.0922	5.8819	7.5467
C	-1.0330	5.2129	6.3114
C	-0.6553	3.8571	6.2443
C	-0.3365	3.1825	7.4396
C	-0.3935	3.8478	8.6771
H	-0.8161	5.7126	9.6825
H	-1.3862	6.9214	7.5837
H	-1.2922	5.7455	5.4077
H	-0.0316	2.1466	7.4090

H	-0.1424	3.3194	9.5860
C	10.5742	6.4830	-2.2236
C	9.5263	6.5849	-1.2939
C	8.5322	5.5919	-1.2423
C	8.5717	4.4878	-2.1170
C	9.6276	4.3961	-3.0455
C	10.6243	5.3864	-3.1000
H	11.3396	7.2457	-2.2640
H	9.4816	7.4279	-0.6188
H	7.7248	5.6873	-0.5302
H	9.6834	3.5510	-3.7161
H	11.4308	5.3020	-3.8146

Molecule 11 MM2

MM2 energy = -186.17 kJ/mol

C	1.3374	3.8542	3.9465
C	1.4148	4.4962	2.7021
C	0.4546	5.4557	2.3616
C	-0.5580	5.7725	3.2656
C	2.5158	4.1024	1.7363
H	-1.3069	6.5328	2.9859
C	-0.6633	5.1072	4.4865
C	0.2765	4.1265	4.8237
C	0.1029	3.4362	6.0293
C	-0.9495	3.7340	6.8933
C	-1.8612	4.7309	6.5593
C	-1.7173	5.4107	5.3525
H	0.7865	2.6275	6.3327
H	-1.0629	3.1796	7.8404
H	-2.6965	4.9710	7.2388

H	-2.4564	6.1868	5.0908
C	4.2987	0.8858	4.8420
C	2.3965	2.8360	4.2814
C	2.5724	1.7617	3.3978
C	3.5356	0.7869	3.6796
H	5.0531	0.1095	5.0549
C	1.7438	1.7132	2.1297
C	4.1564	1.9734	5.7030
C	3.2146	2.9676	5.4136
C	3.1426	4.0749	6.2671
C	3.9543	4.1774	7.3954
C	4.8676	3.1688	7.6877
C	4.9689	2.0719	6.8358
H	2.4483	4.9074	6.0713
H	3.8763	5.0575	8.0562
H	5.5131	3.2444	8.5791
H	5.7087	1.2872	7.0688
H	1.7985	0.6942	1.6871
H	0.6617	1.8753	2.3423
N	2.2332	2.7600	1.2295
H	3.5133	4.1110	2.2333
H	2.5818	4.8676	0.9334
N	1.7017	1.4920	-0.5684
C	2.3259	2.4848	-0.0673
N	3.1234	3.1851	-0.8507
H	1.9071	1.4086	-1.5595
H	3.1864	2.9802	-1.8453
H	3.7743	3.8404	-0.4251
C	0.4152	7.4827	-1.4516
C	0.6001	8.1939	-0.2627

C	0.6124	7.5415	0.9695
C	0.4428	6.1590	1.0377
C	0.2448	5.4440	-0.1436
C	0.2358	6.0990	-1.3740
H	0.7551	9.2852	-0.2799
H	0.7677	8.1239	1.8933
H	0.0884	4.3530	-0.1079
H	0.0688	5.5013	-2.2851
C	4.3429	-2.5423	1.0494
C	3.8664	-2.7515	2.3465
C	3.6055	-1.6780	3.1972
C	3.8130	-0.3683	2.7653
C	4.3005	-0.1521	1.4762
C	4.5579	-1.2275	0.6275
H	3.6738	-3.7721	2.7159
H	3.2230	-1.8689	4.2141
H	4.4909	0.8754	1.1238
H	4.9500	-1.0173	-0.3811
C	0.3904	9.5038	-5.2649
C	-0.1895	10.1042	-4.1494
C	-0.1796	9.4494	-2.9182
C	0.4061	8.1875	-2.7803
C	0.9839	7.5959	-3.9075
C	0.9782	8.2467	-5.1408
H	0.3842	10.0202	-6.2396
H	-0.6589	11.0982	-4.2421
H	-0.6571	9.9445	-2.0567
H	1.4675	6.6076	-3.8378
H	1.4416	7.7676	-6.0199
C	5.1391	-5.8670	-1.5848

C	5.4278	-5.9648	-0.2253
C	5.1689	-4.8894	0.6239
C	4.6207	-3.7013	0.1318
C	4.3363	-3.6159	-1.2343
C	4.5917	-4.6887	-2.0878
H	5.3425	-6.7167	-2.2582
H	5.8639	-6.8943	0.1784
H	5.4194	-4.9925	1.6925
H	3.8915	-2.7013	-1.6596
H	4.3584	-4.6063	-3.1630

Molecule 11 MM3

MM3 energy = -412.627 kJ/mol

C	3.5985	0.8786	3.8607
C	3.7473	1.9461	4.7623
C	3.0711	1.9233	5.9905
C	2.2519	0.8421	6.3218
C	4.6123	3.1132	4.3436
H	1.7323	0.8526	7.2944
C	2.0286	-0.2142	5.4362
C	2.6670	-0.2181	4.1872
C	2.3772	-1.2452	3.2783
C	1.4975	-2.2726	3.6174
C	0.8856	-2.2785	4.8683
C	1.1465	-1.2484	5.7705
H	2.8402	-1.2526	2.2782
H	1.2841	-3.0774	2.8937
H	0.1868	-3.0882	5.1385
H	0.6423	-1.2498	6.7512
C	5.7014	1.0473	0.1374

C	4.3318	0.9242	2.5806
C	4.1903	2.0524	1.7508
C	4.8809	2.1088	0.5285
H	6.2326	1.1187	-0.8263
C	3.2813	3.1740	2.2078
C	5.9231	-0.0606	0.9568
C	5.2742	-0.1446	2.1965
C	5.5630	-1.2246	3.0427
C	6.4521	-2.2240	2.6488
C	7.0747	-2.1492	1.4056
C	6.8147	-1.0667	0.5667
H	5.0914	-1.2979	4.0359
H	6.6636	-3.0712	3.3230
H	7.7804	-2.9368	1.0917
H	7.3268	-1.0045	-0.4078
H	3.0718	3.8673	1.3645
H	2.2910	2.7427	2.4825
N	3.8841	3.8706	3.3365
H	5.5772	2.7354	3.9368
H	4.9068	3.7271	5.2199
N	2.1498	5.4283	3.2548
C	3.3465	5.0201	3.6703
N	4.0554	5.9195	4.2975
H	1.8707	6.3800	3.5066
H	4.9543	5.9906	3.8119
H	3.9453	5.7796	5.3063
C	3.4201	5.2025	8.7319
C	2.3435	5.1229	7.8409
C	2.2297	4.0512	6.9544
C	3.1914	3.0386	6.9380

C	4.2644	3.1097	7.8291
C	4.3775	4.1813	8.7164
H	1.5683	5.9071	7.8410
H	1.3770	4.0036	6.2555
H	5.0290	2.3148	7.8270
H	5.2393	4.2247	9.4029
C	4.5656	5.5056	-2.0800
C	5.1564	5.6253	-0.8170
C	5.2597	4.5210	0.0300
C	4.7688	3.2730	-0.3639
C	4.1836	3.1487	-1.6267
C	4.0824	4.2528	-2.4747
H	5.5562	6.5980	-0.4860
H	5.7339	4.6332	1.0195
H	3.7896	2.1714	-1.9524
H	3.6030	4.1341	-3.4605
C	3.7753	8.5029	11.4399
C	3.4563	8.7271	10.1014
C	3.3400	7.6504	9.2208
C	3.5418	6.3372	9.6645
C	3.8625	6.1261	11.0114
C	3.9781	7.2007	11.8946
H	3.8667	9.3530	12.1369
H	3.2975	9.7565	9.7379
H	3.0971	7.8437	8.1630
H	4.0151	5.1009	11.3870
H	4.2277	7.0196	12.9538
C	4.2455	8.9007	-4.6731
C	3.9033	9.0138	-3.3263
C	4.0090	7.9066	-2.4830

C	4.4561	6.6730	-2.9730
C	4.7956	6.5728	-4.3281
C	4.6923	7.6785	-5.1736
H	4.1627	9.7750	-5.3404
H	3.5464	9.9784	-2.9274
H	3.7248	8.0068	-1.4225
H	5.1598	5.6149	-4.7345
H	4.9662	7.5860	-6.2382

Molecule 11 MMFF

MMFF energy = 143.847 kJ/mol

C	1.4163	2.7770	4.2381
C	1.8628	1.7273	3.3798
C	2.7779	0.7629	3.8510
C	3.2227	0.8457	5.1783
C	1.4081	1.7396	1.9232
H	3.9231	0.0923	5.5403
C	2.8383	1.8901	6.0275
C	1.9525	2.8898	5.5617
C	1.6762	3.9633	6.4465
C	2.2056	4.0188	7.7381
C	3.0418	3.0072	8.1840
C	3.3599	1.9530	7.3314
H	1.0363	4.7865	6.1348
H	1.9621	4.8549	8.3884
H	3.4575	3.0426	9.1874
H	4.0349	1.1792	7.6923
C	-1.2138	5.7414	2.5681
C	0.4688	3.7905	3.6753
C	0.8706	4.5104	2.5070

C	0.0267	5.4985	1.9595
H	-1.8609	6.5081	2.1407
C	2.1803	4.1190	1.8261
C	-1.6618	5.0007	3.6682
C	-0.8375	3.9957	4.2248
C	-1.3833	3.2350	5.2901
C	-2.6566	3.4856	5.8070
C	-3.4324	4.4993	5.2662
C	-2.9390	5.2482	4.2006
H	-0.8154	2.4203	5.7352
H	-3.0364	2.8844	6.6290
H	-4.4241	4.7006	5.6620
H	-3.5704	6.0268	3.7766
H	2.5223	4.9078	1.1528
H	2.9857	4.0838	2.5723
N	2.0826	2.8081	1.1522
H	0.3194	1.8744	1.8684
H	1.5567	0.7507	1.4823
N	2.5660	3.5408	-1.0793
C	2.3790	2.6457	-0.1734
N	2.5197	1.3731	-0.6026
H	2.4115	4.4758	-0.7060
H	3.1388	0.8261	-0.0174
H	2.8701	1.3921	-1.5553
C	4.3691	-2.3333	1.3169
C	3.1947	-2.5394	2.0579
C	2.6790	-1.5392	2.8896
C	3.3218	-0.3002	2.9973
C	4.4998	-0.0910	2.2674
C	5.0149	-1.0933	1.4389

H	2.6739	-3.4935	2.0002
H	1.7672	-1.7333	3.4509
H	5.0198	0.8633	2.3339
H	5.9247	-0.8932	0.8757
C	1.0915	7.7566	-1.5484
C	1.4070	8.2253	-0.2634
C	1.0557	7.4965	0.8783
C	0.3791	6.2753	0.7660
C	0.0466	5.8103	-0.5119
C	0.4025	6.5386	-1.6518
H	1.9488	9.1615	-0.1425
H	1.3222	7.8860	1.8587
H	-0.4748	4.8617	-0.6319
H	0.1352	6.1411	-2.6295
C	5.9350	-5.3963	-1.2485
C	6.7452	-4.8221	-0.2725
C	6.2347	-3.8246	0.5628
C	4.9062	-3.3848	0.4381
C	4.1060	-3.9780	-0.5528
C	4.6154	-4.9747	-1.3896
H	6.3316	-6.1714	-1.8990
H	7.7748	-5.1507	-0.1579
H	6.8823	-3.3975	1.3259
H	3.0754	-3.6560	-0.6886
H	3.9811	-5.4183	-2.1527
C	2.2245	9.9601	-5.0528
C	2.5744	8.6182	-4.9280
C	2.2039	7.9017	-3.7867
C	1.4785	8.5134	-2.7503
C	1.1363	9.8682	-2.8966

C	1.5052	10.5859	-4.0379
H	2.5117	10.5172	-5.9407
H	3.1375	8.1260	-5.7166
H	2.4968	6.8561	-3.7053
H	0.5641	10.3736	-2.1214
H	1.2272	11.6321	-4.1337

Molecule 11 MMFF

MMFF energy = 159.403 kJ/mol

C	1.2627	2.7573	4.1262
C	1.7713	1.7369	3.2650
C	2.6445	0.7514	3.7709
C	2.9990	0.7959	5.1278
C	1.4275	1.8084	1.7800
H	3.6676	0.0268	5.5162
C	2.5618	1.8169	5.9796
C	1.7084	2.8302	5.4857
C	1.3700	3.8762	6.3819
C	1.8117	3.8931	7.7071
C	2.6187	2.8689	8.1774
C	2.9949	1.8406	7.3168
H	0.7502	4.7079	6.0527
H	1.5233	4.7092	8.3645
H	2.9663	2.8742	9.2069
H	3.6451	1.0557	7.6989
C	-1.3042	5.7398	2.3896
C	0.3396	3.7803	3.5400
C	0.8038	4.5479	2.4275
C	-0.0225	5.5376	1.8561
H	-1.9366	6.5091	1.9451

C	2.1638	4.2047	1.8255
C	-1.8081	4.9565	3.4344
C	-1.0033	3.9465	4.0101
C	-1.6032	3.1422	5.0126
C	-2.9114	3.3555	5.4538
C	-3.6686	4.3745	4.8971
C	-3.1211	5.1661	3.8906
H	-1.0509	2.3224	5.4678
H	-3.3322	2.7215	6.2299
H	-4.6870	4.5471	5.2344
H	-3.7376	5.9487	3.4521
H	2.5286	5.0184	1.1939
H	2.9195	4.1633	2.6217
N	2.1372	2.9151	1.1081
H	0.3440	1.9385	1.6548
H	1.6092	0.8456	1.2966
N	2.8273	3.6940	-1.0341
C	2.6784	2.7792	-0.1391
N	3.1174	1.5583	-0.4826
H	2.4288	4.5734	-0.7076
H	3.5282	1.4857	-1.4031
H	3.3909	0.8749	0.2046
C	4.3693	-2.3258	1.2995
C	3.0761	-2.4640	1.8277
C	2.5152	-1.4701	2.6373
C	3.2306	-0.3036	2.9349
C	4.5268	-0.1650	2.4207
C	5.0863	-1.1610	1.6133
H	2.4956	-3.3614	1.6206
H	1.5122	-1.6133	3.0345

H	5.1068	0.7310	2.6369
H	6.0896	-1.0132	1.2172
C	1.2169	7.9058	-1.5181
C	1.4658	8.3340	-0.2048
C	1.0572	7.5699	0.8938
C	0.3885	6.3534	0.7082
C	0.1212	5.9293	-0.5993
C	0.5346	6.6926	-1.6958
H	2.0006	9.2652	-0.0275
H	1.2738	7.9276	1.8984
H	-0.3953	4.9869	-0.7751
H	0.3179	6.3284	-2.6987
C	6.0783	-5.3679	-1.1990
C	6.7636	-4.9252	-0.0707
C	6.2066	-3.9345	0.7426
C	4.9552	-3.3700	0.4434
C	4.2806	-3.8319	-0.6993
C	4.8369	-4.8217	-1.5142
H	6.5114	-6.1376	-1.8324
H	7.7317	-5.3513	0.1797
H	6.7549	-3.6114	1.6252
H	3.3146	-3.4113	-0.9716
H	4.3007	-5.1624	-2.3962
C	2.5292	10.2154	-4.8879
C	2.8896	8.8749	-4.7768
C	2.4607	8.1238	-3.6791
C	1.6654	8.6991	-2.6737
C	1.3140	10.0529	-2.8050
C	1.7412	10.8051	-3.9027
H	2.8621	10.7994	-5.7418

H	3.5070	8.4110	-5.5415
H	2.7633	7.0803	-3.6060
H	0.6897	10.5302	-2.0524
H	1.4556	11.8502	-3.9881

Molecule 11 OPLS-2005

OPLS-2005 energy = 159.403 kJ/mol

C	-1.7746	4.5651	-0.7385
C	-0.6128	4.8330	0.0362
C	-0.1651	6.1600	0.2508
C	-0.8938	7.2261	-0.3044
C	0.2030	3.6544	0.5671
H	-0.5549	8.2386	-0.1383
C	-2.0133	6.9856	-1.1189
C	-2.4458	5.6566	-1.3617
C	-3.5351	5.4565	-2.2459
C	-4.1994	6.5479	-2.8371
C	-3.7787	7.8608	-2.5628
C	-2.6834	8.0783	-1.7081
H	-3.8801	4.4619	-2.4854
H	-5.0328	6.3747	-3.5026
H	-4.2877	8.6993	-3.0160
H	-2.3536	9.0890	-1.5159
C	-2.9278	0.4421	-1.3560
C	-2.1996	3.1483	-0.9238
C	-1.2677	2.2195	-1.4661
C	-1.6311	0.8684	-1.6911
H	-3.2035	-0.5888	-1.5269
C	0.1699	2.6815	-1.7199
C	-3.8436	1.3234	-0.7576

C	-3.4804	2.6733	-0.5181
C	-4.4110	3.5106	0.1461
C	-5.6790	3.0295	0.5239
C	-6.0359	1.6971	0.2532
C	-5.1165	0.8442	-0.3831
H	-4.1678	4.5367	0.3776
H	-6.3772	3.6858	1.0232
H	-7.0086	1.3264	0.5427
H	-5.3904	-0.1835	-0.5731
H	0.7043	1.9812	-2.3602
H	0.1448	3.6033	-2.3018
N	0.9273	2.9345	-0.4873
H	-0.4674	2.9536	1.0659
H	0.8803	3.9787	1.3555
N	2.4568	1.1374	-0.6006
C	2.1480	2.3234	-0.2354
N	3.0709	3.0718	0.4454
H	1.6927	0.6832	-1.0869
H	2.9478	4.0337	0.7276
H	3.9870	2.7080	0.6624
C	3.4551	7.0024	2.4530
C	3.5032	6.6122	1.0965
C	2.3213	6.3455	0.3793
C	1.0622	6.4604	1.0014
C	1.0065	6.8565	2.3519
C	2.1895	7.1220	3.0690
H	4.4563	6.5072	0.5984
H	2.3794	6.0414	-0.6568
H	0.0490	6.9536	2.8432
H	2.1204	7.4298	4.1022

C	1.2005	-1.9868	-3.2635
C	0.4608	-1.1516	-4.1293
C	-0.4712	-0.2265	-3.6200
C	-0.6857	-0.1170	-2.2318
C	0.0365	-0.9594	-1.3643
C	0.9724	-1.8792	-1.8742
H	0.6199	-1.2077	-5.1963
H	-1.0188	0.4105	-4.2996
H	-0.1165	-0.8904	-0.2962
H	1.5195	-2.5093	-1.1877
C	7.0786	7.8044	4.6542
C	6.0295	7.0870	5.2577
C	4.8473	6.8275	4.5370
C	4.6936	7.2776	3.2059
C	5.7569	7.9967	2.6139
C	6.9410	8.2599	3.3302
H	7.9863	8.0047	5.2053
H	6.1315	6.7329	6.2733
H	4.0552	6.2673	5.0119
H	5.6637	8.3613	1.6013
H	7.7428	8.8133	2.8632
C	4.0959	-4.7819	-4.8150
C	4.3996	-4.0053	-3.6817
C	3.4514	-3.0939	-3.1773
C	2.1891	-2.9431	-3.7948
C	1.8994	-3.7289	-4.9333
C	2.8436	-4.6428	-5.4412
H	4.8221	-5.4816	-5.2031
H	5.3609	-4.1051	-3.1988
H	3.7025	-2.4964	-2.3123

H	0.9390	-3.6388	-5.4193
H	2.6052	-5.2381	-6.3106

Molecule 11 OPLS3e

OPLS3e energy = 151.596 kJ/mol

C	0.6592	6.9414	-2.1306
C	-0.0184	5.7733	-1.7049
C	-1.3514	5.8445	-1.2614
C	-2.0155	7.0678	-1.2547
C	0.7821	4.4618	-1.6714
H	-3.0403	7.1154	-0.9156
C	-1.3631	8.2632	-1.6090
C	0.0002	8.2163	-2.0286
C	0.6456	9.4576	-2.3130
C	-0.0404	10.6656	-2.2250
C	-1.3753	10.6903	-1.8450
C	-2.0381	9.5079	-1.5341
H	1.6856	9.4681	-2.6039
H	0.4698	11.5906	-2.4529
H	-1.8968	11.6346	-1.7798
H	-3.0718	9.5344	-1.2208
C	4.7505	6.7357	-3.5450
C	2.0832	6.8614	-2.6398
C	3.1001	6.3120	-1.8267
C	4.4261	6.2468	-2.2835
H	5.7738	6.6879	-3.8887
C	2.6585	5.6447	-0.5214
C	3.7648	7.2462	-4.4112
C	2.4059	7.2913	-3.9745
C	1.4278	7.7481	-4.9082

C	1.7898	8.1759	-6.1823
C	3.1198	8.1563	-6.5797
C	4.1043	7.6943	-5.7129
H	0.3849	7.7655	-4.6281
H	1.0301	8.5242	-6.8676
H	3.3876	8.4908	-7.5718
H	5.1355	7.6639	-6.0342
H	3.4905	5.5347	0.1702
H	2.0102	6.3461	0.0067
N	1.8997	4.3960	-0.7094
H	1.1874	4.3057	-2.6734
H	0.1016	3.6231	-1.5333
N	1.9519	2.0169	-0.6449
C	2.4682	3.1208	-0.3904
N	3.6998	3.1089	0.2559
H	1.0607	2.1422	-1.0963
H	4.4087	3.6519	-0.2086
H	4.0282	2.1665	0.3738
C	-3.4888	2.3936	0.1743
C	-3.9457	3.1060	-0.9410
C	-3.2553	4.2231	-1.4049
C	-2.0906	4.6515	-0.7641
C	-1.6386	3.9529	0.3585
C	-2.3264	2.8335	0.8181
H	-4.8318	2.7822	-1.4664
H	-3.6180	4.7507	-2.2751
H	-0.7437	4.2768	0.8718
H	-1.9547	2.3137	1.6889
C	7.5014	4.3768	0.0666
C	7.0601	3.7982	-1.1294

C	6.0724	4.4122	-1.8945
C	5.5023	5.6161	-1.4766
C	5.9485	6.2076	-0.2934
C	6.9350	5.5913	0.4719
H	7.4723	2.8570	-1.4634
H	5.7347	3.9436	-2.8087
H	5.5249	7.1467	0.0337
H	7.2696	6.0710	1.3801
C	-5.5974	-1.0679	1.5844
C	-4.2055	-1.0528	1.5982
C	-3.5239	0.0724	1.1408
C	-4.2230	1.1901	0.6651
C	-5.6240	1.1545	0.6587
C	-6.3105	0.0324	1.1165
H	-6.1278	-1.9399	1.9395
H	-3.6552	-1.9099	1.9584
H	-2.4434	0.0673	1.1435
H	-6.1876	2.0074	0.3104
H	-7.3907	0.0176	1.1110
C	10.5230	2.4657	2.4399
C	10.6188	2.4444	1.0514
C	9.6369	3.0652	0.2831
C	8.5532	3.7131	0.8918
C	8.4758	3.7240	2.2912
C	9.4543	3.1033	3.0638
H	11.2834	1.9838	3.0378
H	11.4520	1.9513	0.5717
H	9.7296	3.0531	-0.7929
H	7.6446	4.2034	2.7871
H	9.3821	3.1139	4.1418

Molecule 11 OPLS

OPLS energy = -12.004 kJ/mol

C	1.3427	0.0641	0.0437
C	0.6604	0.8348	0.9367
C	-0.7087	0.8549	0.9306
C	-1.4319	0.1149	0.0605
C	1.4745	1.7173	1.8963
H	-2.5099	0.1662	0.1056
C	-0.8530	-0.6373	-0.8881
C	0.5883	-0.6869	-0.9627
C	1.1743	-1.4377	-1.9990
C	0.4004	-2.1124	-2.8693
C	-1.0031	-2.0797	-2.7742
C	-1.6149	-1.3615	-1.8158
H	2.2456	-1.4879	-2.1191
H	0.8665	-2.6884	-3.6557
H	-1.5981	-2.6318	-3.4873
H	-2.6937	-1.3377	-1.7696
C	5.6318	0.0943	0.0736
C	2.8578	0.0551	0.0619
C	3.5430	1.2278	-0.0555
C	4.9129	1.2332	-0.0468
H	6.7100	0.1580	0.0695
C	2.7316	2.5313	-0.1364
C	5.0499	-1.1020	0.2488
C	3.6088	-1.1846	0.2736
C	3.0194	-2.4406	0.5113
C	3.7902	-3.5314	0.6771
C	5.1939	-3.4466	0.6264

C	5.8087	-2.2681	0.4221
H	1.9478	-2.5563	0.5670
H	3.3217	-4.4889	0.8532
H	5.7865	-4.3397	0.7626
H	6.8876	-2.2224	0.4011
H	3.3297	3.3635	-0.4884
H	1.9557	2.4551	-0.8992
N	2.1000	2.8384	1.1638
H	2.2532	1.1356	2.3912
H	0.8801	2.0745	2.7301
N	1.4082	4.5011	2.7080
C	2.0881	4.1698	1.6848
N	2.8429	5.2022	1.1044
H	0.8619	3.7157	3.0304
H	2.8003	6.1252	1.5094
H	3.4597	5.0189	0.3280
C	-2.9497	3.3558	3.6428
C	-2.2831	3.9203	2.5431
C	-1.5638	3.1049	1.6557
C	-1.4957	1.7187	1.8651
C	-2.1688	1.1508	2.9573
C	-2.8924	1.9663	3.8428
H	-2.3222	4.9862	2.3717
H	-1.0455	3.5485	0.8165
H	-2.1224	0.0843	3.1225
H	-3.3956	1.5207	4.6881
C	7.2073	4.8801	-0.3421
C	7.1612	3.9800	-1.4197
C	6.4168	2.7930	-1.3220
C	5.7103	2.4965	-0.1466

C	5.7633	3.3905	0.9346
C	6.5046	4.5784	0.8360
H	7.7051	4.1948	-2.3276
H	6.3853	2.1068	-2.1556
H	5.2129	3.1725	1.8397
H	6.5201	5.2641	1.6704
C	-5.1363	5.8662	6.3778
C	-3.8667	6.2550	5.9267
C	-3.1552	5.4374	5.0356
C	-3.7095	4.2263	4.5914
C	-4.9808	3.8392	5.0445
C	-5.6924	4.6572	5.9352
H	-5.6841	6.4962	7.0637
H	-3.4341	7.1848	6.2665
H	-2.1745	5.7413	4.6990
H	-5.4200	2.9138	4.7022
H	-6.6713	4.3559	6.2789
C	9.4788	8.5291	-0.6474
C	9.5411	7.7568	0.5215
C	8.8013	6.5687	0.6207
C	7.9956	6.1463	-0.4487
C	7.9350	6.9213	-1.6180
C	8.6745	8.1097	-1.7168
H	10.0482	9.4443	-0.7234
H	10.1608	8.0759	1.3468
H	8.8610	5.9762	1.5219
H	7.3119	6.6104	-2.4435
H	8.6223	8.7035	-2.6178

Molecule 12 MMFF

MMFF energy = 255.769 kJ/mol

C	1.2060	-1.7615	-2.0081
C	2.6784	-2.1776	-1.7012
C	2.6615	-2.8937	-0.3390
N	1.1099	-0.4859	-2.7167
H	0.6865	-2.5554	-2.5568
H	3.0959	-2.8211	-2.4824
O	3.5423	-1.0291	-1.6110
H	2.5092	-3.9707	-0.4653
H	3.5660	-2.6995	0.2448
C	0.6199	-1.6728	-0.6241
C	1.4474	-2.3016	0.3126
C	1.0910	-2.3727	1.6499
C	-0.1159	-1.7947	2.0477
C	-0.9537	-1.1738	1.1098
C	-0.5926	-1.1183	-0.2378
H	1.7314	-2.8654	2.3749
H	-0.4145	-1.8335	3.0926
H	-1.8960	-0.7387	1.4347
H	-1.2562	-0.6566	-0.9620
H	3.4945	-0.5911	-2.4792
H	1.3299	0.3088	-2.1290
C	1.2646	-0.2791	-4.0533
S	1.5941	-1.4667	-5.1814
N	1.2040	1.0115	-4.4977
H	1.5615	1.1115	-5.4437
C	-0.3978	4.5866	-2.9393
C	-0.7897	3.3692	-2.3618
C	-0.2564	2.1713	-2.8679
C	0.6523	2.1767	-3.9279

C	0.9990	3.3988	-4.5153
C	0.4962	4.6137	-4.0197
H	-0.8045	5.5221	-2.5501
C	-1.7620	3.3529	-1.2050
H	-0.6139	1.2352	-2.4477
H	1.6882	3.4209	-5.3596
C	0.8843	5.9209	-4.6691
F	-2.3051	2.1263	-0.9699
F	-1.1786	3.7366	-0.0375
F	-2.8138	4.1964	-1.3950
F	2.1492	5.9077	-5.1729
F	0.0682	6.2396	-5.7097
F	0.8343	6.9775	-3.8123

Molecule 12 MMFF

MMFF energy = 257.209 kJ/mol

C	-2.0749	-1.8408	-0.3871
C	-1.3568	-0.4934	-0.7112
C	-0.0121	-0.8545	-1.3649
N	-2.8698	-1.7723	0.8390
H	-2.6878	-2.1660	-1.2356
H	-1.9598	0.1464	-1.3635
O	-1.1008	0.2679	0.4840
H	-0.1140	-0.9346	-2.4521
H	0.7813	-0.1481	-1.1039
C	-0.9062	-2.7805	-0.2535
C	0.2542	-2.2170	-0.7966
C	1.4499	-2.9175	-0.8178
C	1.4757	-4.2043	-0.2776
C	0.3122	-4.7779	0.2554

C	-0.8913	-4.0697	0.2601
H	2.3477	-2.4804	-1.2438
H	2.4034	-4.7715	-0.2781
H	0.3466	-5.7862	0.6617
H	-1.7918	-4.5312	0.6514
H	-1.9554	0.3249	0.9476
H	-2.3414	-1.9213	1.6884
C	-4.1504	-1.3289	0.9672
S	-5.0576	-0.6488	-0.2604
N	-4.7057	-1.3620	2.2144
H	-5.5198	-0.7588	2.2919
C	-3.9005	-3.5503	5.7230
C	-4.6657	-2.3757	5.7739
C	-4.9181	-1.6780	4.5809
C	-4.3897	-2.1151	3.3614
C	-3.6519	-3.2999	3.3286
C	-3.3895	-4.0234	4.5043
H	-3.7097	-4.1114	6.6400
C	-5.2478	-1.8881	7.0797
H	-5.5198	-0.7699	4.6193
H	-3.3172	-3.7155	2.3816
C	-2.6113	-5.3178	4.4588
F	-5.3478	-0.5317	7.1419
F	-6.5025	-2.3683	7.2932
F	-4.5119	-2.2589	8.1631
F	-1.9621	-5.5182	3.2787
F	-1.6582	-5.3884	5.4277
F	-3.4105	-6.4046	4.6367

Molecule 12 OPLS-2005

OPLS-2005 energy = 257.209 kJ/mol

C	0.0017	-3.7661	0.3075
C	0.2911	-2.2452	0.0851
C	1.7143	-2.1254	-0.5225
N	-0.4449	-4.0008	1.6766
H	-0.7294	-4.1099	-0.4253
H	-0.4530	-1.8145	-0.5868
O	0.2503	-1.5212	1.3160
H	1.6711	-1.8153	-1.5671
H	2.3576	-1.4291	0.0167
C	1.3257	-4.4291	0.0112
C	2.2699	-3.5194	-0.4501
C	3.5749	-3.9239	-0.7847
C	3.9106	-5.2876	-0.6392
C	2.9528	-6.2154	-0.1667
C	1.6477	-5.7905	0.1647
H	4.3051	-3.2121	-1.1419
H	4.9060	-5.6253	-0.8888
H	3.2199	-7.2567	-0.0584
H	0.9125	-6.4958	0.5250
H	0.4261	-0.6076	1.1385
H	-0.0961	-3.3055	2.3217
C	-1.3676	-4.8988	2.0987
S	-2.1206	-6.0545	1.1760
N	-1.6459	-4.7957	3.4226
H	-2.2082	-5.5534	3.7864
C	-0.4054	-1.9033	6.2693
C	-0.9069	-1.4711	5.0242
C	-1.3238	-2.4280	4.0765
C	-1.2233	-3.8094	4.3570

C	-0.7504	-4.2272	5.6167
C	-0.3375	-3.2782	6.5766
H	-0.0734	-1.1766	6.9966
C	-0.9832	0.0111	4.6913
H	-1.7167	-2.0868	3.1286
H	-0.6827	-5.2805	5.8505
C	0.1988	-3.7343	7.9243
F	-2.0565	0.2679	3.9437
F	-1.0577	0.7457	5.8011
F	0.1072	0.3658	4.0133
F	-0.3438	-4.8988	8.2799
F	1.5196	-3.8802	7.8398
F	-0.0774	-2.8344	8.8680

Molecule 12 OPLS3e

OPLS3e energy = 37.988 kJ/mol

C	0.6905	0.2249	2.0195
C	-0.6032	0.7373	1.3479
C	-0.2723	0.9860	-0.1199
N	1.4956	1.2838	2.6547
H	0.4425	-0.5893	2.7022
H	-1.3117	-0.0919	1.4068
O	-1.2402	1.8460	1.9503
H	0.0194	2.0250	-0.2798
H	-1.1277	0.7724	-0.7603
C	1.4570	-0.3559	0.8485
C	0.9081	0.0771	-0.3643
C	1.5209	-0.2626	-1.5701
C	2.6507	-1.0834	-1.5668
C	3.1816	-1.5411	-0.3589

C	2.5917	-1.1717	0.8516
H	1.1260	0.0997	-2.5084
H	3.1211	-1.3587	-2.5012
H	4.0614	-2.1706	-0.3619
H	3.0270	-1.5069	1.7828
H	-1.5351	1.5841	2.8288
H	2.1322	1.7924	2.0592
C	1.3960	1.6638	3.9630
S	0.5083	0.8873	5.1337
N	2.1380	2.7352	4.3529
H	2.1867	2.8597	5.3535
C	3.8862	5.8961	2.2026
C	2.7767	5.2437	1.6503
C	2.1813	4.1941	2.3589
C	2.6967	3.7831	3.5975
C	3.7899	4.4595	4.1426
C	4.3964	5.5154	3.4504
H	4.3517	6.7108	1.6622
C	2.2142	5.6662	0.2926
H	1.2970	3.7217	1.9503
H	4.1835	4.1554	5.1036
C	5.6000	6.2439	4.0499
F	1.7795	4.6051	-0.3894
F	1.1927	6.4981	0.4879
F	3.1339	6.2891	-0.4462
F	6.7134	5.6286	3.6554
F	5.6483	7.5138	3.6449
F	5.5562	6.2312	5.3831

Molecule 13 AMBER

AMBER energy = 118.308 kJ/mol

C	2.2173	1.3379	-0.0086
C	-0.5453	1.6737	-0.0102
C	0.3067	2.6787	-0.3042
C	1.6506	2.5319	-0.3127
H	-1.6073	1.8702	-0.0260
H	-0.1044	3.6478	-0.5480
O	2.4202	3.6155	-0.6339
C	1.3421	0.2109	0.3044
C	-0.0944	0.4424	0.2931
C	-0.9544	-0.6283	0.5993
C	-0.4490	-1.8423	0.8943
C	0.9417	-2.0697	0.9015
C	1.8110	-1.0805	0.6155
H	-2.0252	-0.4841	0.5986
H	-1.1214	-2.6562	1.1267
H	1.3190	-3.0546	1.1383
H	2.8706	-1.2904	0.6252
C	6.4805	0.9625	0.0284
C	3.7166	1.1843	0.0197
C	4.4340	1.2434	-1.1252
C	5.7917	1.1477	-1.1221
H	7.5561	0.8853	-0.0143
O	3.7813	1.4319	-2.3105
C	6.5658	1.2632	-2.4347
C	5.8723	0.8796	1.2256
C	4.4259	0.9964	1.2786
C	3.7907	0.9310	2.5337
C	4.5170	0.7509	3.6541
C	5.9198	0.6292	3.6009

C	6.5800	0.6927	2.4278
H	2.7167	1.0267	2.6112
H	4.0130	0.7022	4.6095
H	6.4747	0.4879	4.5180
H	7.6571	0.6032	2.4223
H	3.3491	3.3986	-0.7584
H	4.4074	1.7051	-2.9958
N	6.1345	2.4039	-3.2500
H	7.6379	1.3426	-2.2487
H	6.3932	0.3426	-2.9946
C	6.8157	2.4998	-4.5367
C	5.4900	6.0498	-1.3127
C	4.6418	5.6967	-2.3755
C	4.8657	4.4853	-3.0424
C	5.9218	3.6682	-2.6141
C	6.7162	4.1293	-1.5409
N	6.5118	5.2888	-0.9010
H	5.3107	6.9841	-0.7982
H	4.2203	4.1806	-3.8529
H	7.5401	3.5146	-1.2134
H	6.8317	1.5263	-5.0280
H	6.2975	3.2052	-5.1879
H	7.8415	2.8412	-4.3902
H	3.8270	6.3436	-2.6670

Molecule 13 MM2

MM2 energy = 201.214 kJ/mol

C	3.1905	-0.0381	0.5798
C	1.6935	-0.0540	-1.7510
C	1.0723	-0.2237	-0.5162

C	1.8066	-0.2201	0.6712
H	1.0761	-0.0642	-2.6653
H	-0.0209	-0.3646	-0.4797
O	1.2072	-0.3885	1.8820
C	3.8328	0.1354	-0.6522
C	3.0751	0.1263	-1.8284
C	3.6988	0.2971	-3.0668
C	5.0779	0.4798	-3.1443
C	5.8373	0.4934	-1.9774
C	5.2163	0.3224	-0.7413
H	3.1090	0.2903	-3.9992
H	5.5662	0.6148	-4.1243
H	6.9293	0.6407	-2.0327
H	5.8518	0.3419	0.1593
C	5.4891	-0.1218	4.1601
C	3.9797	-0.0429	1.8556
C	4.6164	-1.2402	2.2010
C	5.3936	-1.2674	3.3665
H	6.0981	-0.1533	5.0786
O	4.4648	-2.3237	1.3898
C	6.1499	-2.5121	3.7830
C	4.8423	1.0640	3.8122
C	4.0805	1.1090	2.6416
C	3.4482	2.3053	2.2892
C	3.5657	3.4384	3.0925
C	4.3222	3.3868	4.2604
C	4.9597	2.2003	4.6167
H	2.8433	2.3840	1.3709
H	3.0610	4.3761	2.8041
H	4.4180	4.2818	4.8983

H	5.5593	2.1752	5.5424
H	0.2478	-0.5004	1.7741
H	4.9390	-3.0991	1.7296
N	7.0206	-3.0086	2.7114
H	6.7783	-2.3407	4.6870
H	5.4035	-3.2913	4.0607
C	7.2256	-4.4349	2.4665
C	9.0969	-0.2620	0.4565
C	8.5268	-1.3567	-0.1901
C	7.8290	-2.2924	0.5730
C	7.7125	-2.1181	1.9574
C	8.3426	-0.9711	2.4655
N	9.0187	-0.0587	1.7681
H	9.6583	0.5074	-0.1212
H	7.3639	-3.1484	0.0581
H	8.2664	-0.8468	3.5686
H	6.7034	-4.7617	1.5392
H	8.3131	-4.6652	2.3899
H	6.8166	-5.0374	3.3085
H	8.6203	-1.4757	-1.2826

Molecule 13 MM3

MM3 energy = 186.981 kJ/mol

C	0.9269	3.1199	-1.0086
C	-0.3797	5.4810	-0.2079
C	-0.4479	4.3287	0.5748
C	0.1959	3.1536	0.1872
H	-0.9022	6.3909	0.1321
H	-1.0214	4.3445	1.5172
O	0.0744	2.0743	1.0096

C	1.0051	4.3397	-1.8339
C	0.3396	5.4997	-1.4065
C	0.4055	6.6619	-2.1835
C	1.1269	6.6779	-3.3768
C	1.7899	5.5287	-3.8014
C	1.7290	4.3665	-3.0332
H	-0.1141	7.5772	-1.8546
H	1.1744	7.5988	-3.9823
H	2.3641	5.5382	-4.7433
H	2.2621	3.4666	-3.3818
C	2.9127	-0.4428	-2.2931
C	1.6015	1.8882	-1.4459
C	0.9546	0.9832	-2.3010
C	1.6075	-0.1806	-2.7324
H	3.4225	-1.3571	-2.6416
O	-0.3166	1.2031	-2.7422
C	0.9273	-1.1564	-3.6704
C	3.6011	0.4293	-1.4469
C	2.9772	1.6039	-1.0056
C	3.6757	2.4779	-0.1627
C	4.9792	2.1877	0.2394
C	5.5977	1.0194	-0.2001
C	4.9100	0.1450	-1.0408
H	3.2016	3.4079	0.1916
H	5.5200	2.8832	0.9033
H	6.6292	0.7875	0.1148
H	5.4080	-0.7762	-1.3870
H	0.5562	1.2813	0.7197
H	-0.7360	2.0200	-2.4229
N	1.2130	-0.8379	-5.0719

H	1.2253	-2.1938	-3.4035
H	-0.1711	-1.1330	-3.4927
C	0.6785	0.3513	-5.7252
C	5.1340	-1.8983	-6.0364
C	4.7477	-0.5711	-6.2078
C	3.4427	-0.1884	-5.9007
C	2.5403	-1.1463	-5.4186
C	3.0280	-2.4597	-5.2923
N	4.2881	-2.8434	-5.5845
H	6.1615	-2.2210	-6.2720
H	3.1542	0.8666	-6.0235
H	2.3597	-3.2659	-4.9496
H	1.1057	1.2924	-5.3135
H	0.8526	0.3383	-6.8244
H	-0.4233	0.4139	-5.5847
H	5.4732	0.1701	-6.5791

Molecule 13 MMFF

MMFF energy = 471.799 kJ/mol

C	1.6028	0.7763	0.5043
C	-0.3906	2.5926	1.3236
C	0.5189	2.0964	2.2515
C	1.5050	1.2077	1.8432
H	-1.1521	3.2913	1.6628
H	0.4649	2.4068	3.2910
O	2.3621	0.7816	2.8249
C	0.6733	1.2940	-0.4520
C	-0.3257	2.2069	-0.0222
C	-1.2528	2.7237	-0.9428
C	-1.2137	2.3558	-2.2864

C	-0.2450	1.4657	-2.7278
C	0.6840	0.9440	-1.8244
H	-2.0190	3.4239	-0.6165
H	-1.9383	2.7659	-2.9845
H	-0.2063	1.1745	-3.7739
H	1.4298	0.2519	-2.2095
C	4.6386	-2.0301	-0.6743
C	2.6376	-0.2165	0.1096
C	3.9343	0.2235	-0.2326
C	4.9419	-0.6625	-0.6357
H	5.4144	-2.7374	-0.9661
O	4.1984	1.5691	-0.1593
C	6.3586	-0.2155	-0.9395
C	3.3655	-2.5139	-0.3354
C	2.3479	-1.6123	0.0645
C	1.0870	-2.1604	0.4021
C	0.8406	-3.5336	0.3444
C	1.8490	-4.4003	-0.0514
C	3.1028	-3.8933	-0.3880
H	0.2708	-1.5135	0.7204
H	-0.1401	-3.9190	0.6112
H	1.6623	-5.4700	-0.0967
H	3.8790	-4.5921	-0.6933
H	3.1237	0.3621	2.3898
H	4.9930	1.7408	-0.7130
N	6.3757	1.0770	-1.6741
H	6.8791	-0.1574	0.0246
H	6.8813	-0.9728	-1.5386
C	5.9767	0.9618	-3.0879
C	9.8277	3.3107	-0.8872

C	8.9535	3.0416	0.1488
C	7.8096	2.2934	-0.1288
C	7.5517	1.8205	-1.4239
C	8.5043	2.1721	-2.3867
N	9.6316	2.8918	-2.1523
H	10.7313	3.8907	-0.7234
H	7.1043	2.1031	0.6772
H	8.4070	1.8675	-3.4237
H	5.9240	1.9489	-3.5610
H	6.6652	0.3220	-3.6514
H	4.9753	0.5253	-3.1670
H	9.1482	3.4117	1.1489

Molecule 13 MMFF

MMFF energy = 474.472 kJ/mol

C	0.5979	0.4758	0.1950
C	-2.1898	0.1229	0.0272
C	-1.3814	-0.9300	0.4433
C	-0.0064	-0.7542	0.5236
H	-3.2648	-0.0366	-0.0303
H	-1.8232	-1.8883	0.7033
O	0.7059	-1.8457	0.9453
C	-0.2309	1.5582	-0.2340
C	-1.6347	1.3639	-0.3126
C	-2.4695	2.4142	-0.7281
C	-1.9429	3.6586	-1.0688
C	-0.5730	3.8687	-0.9993
C	0.2703	2.8340	-0.5886
H	-3.5465	2.2703	-0.7905
H	-2.6027	4.4608	-1.3888

H	-0.1537	4.8359	-1.2653
H	1.3389	3.0409	-0.5533
C	4.8424	0.9358	0.6367
C	2.0671	0.6414	0.3241
C	2.6251	1.1036	1.5391
C	4.0061	1.2660	1.7113
H	5.9196	1.0506	0.7477
O	1.8338	1.4114	2.6206
C	4.5365	1.7612	3.0453
C	4.3333	0.4740	-0.5864
C	2.9369	0.3213	-0.7604
C	2.4835	-0.1483	-2.0176
C	3.3691	-0.4531	-3.0534
C	4.7344	-0.2963	-2.8632
C	5.2120	0.1647	-1.6381
H	1.4200	-0.2797	-2.2105
H	2.9871	-0.8086	-4.0069
H	5.4287	-0.5279	-3.6665
H	6.2861	0.2861	-1.5103
H	1.6578	-1.6559	0.8795
H	0.8948	1.3339	2.3753
N	5.9792	2.0564	3.0529
H	3.9755	2.6458	3.3725
H	4.3223	0.9854	3.7926
C	6.8576	1.0327	3.6064
C	7.4181	5.7945	1.8553
C	8.2874	4.9245	2.4815
C	7.8058	3.6750	2.8823
C	6.4735	3.2960	2.6646
C	5.7026	4.2720	2.0154

N	6.1306	5.4988	1.6095
H	7.7429	6.7768	1.5265
H	8.5031	3.0093	3.3810
H	4.6593	4.0959	1.7711
H	7.2237	1.3300	4.5949
H	6.3384	0.0746	3.7225
H	7.7099	0.8436	2.9443
H	9.3187	5.2062	2.6604

Molecule 13 OPLS-2005

OPLS-2005 energy = 474.472 kJ/mol

C	-0.1037	-0.3695	-1.6264
C	-1.4930	1.0376	-3.6615
C	-1.2803	-0.3459	-3.7799
C	-0.5931	-1.0435	-2.7716
H	-2.0236	1.5625	-4.4424
H	-1.6465	-0.8759	-4.6472
O	-0.4085	-2.3877	-2.9186
C	-0.3201	1.0310	-1.5092
C	-1.0177	1.7288	-2.5307
C	-1.2421	3.1168	-2.4195
C	-0.7787	3.8216	-1.2933
C	-0.0865	3.1403	-0.2761
C	0.1441	1.7562	-0.3841
H	-1.7730	3.6481	-3.1962
H	-0.9545	4.8844	-1.2091
H	0.2695	3.6764	0.5925
H	0.6807	1.2671	0.4167
C	1.9032	-2.5570	1.5001
C	0.6023	-1.1339	-0.5733

C	-0.0930	-1.4811	0.6116
C	0.5582	-2.1727	1.6577
H	2.3968	-3.0854	2.3023
O	-1.4047	-1.1206	0.7494
C	-0.1586	-2.4929	2.9661
C	2.6059	-2.2371	0.3248
C	1.9627	-1.5182	-0.7152
C	2.7038	-1.1951	-1.8781
C	4.0501	-1.5873	-2.0047
C	4.6761	-2.3045	-0.9693
C	3.9548	-2.6270	0.1947
H	2.2534	-0.6421	-2.6897
H	4.6027	-1.3349	-2.8983
H	5.7097	-2.6038	-1.0661
H	4.4433	-3.1729	0.9887
H	0.0954	-2.7643	-2.2107
H	-1.7128	-0.5942	0.0243
N	0.5924	-2.1358	4.1753
H	-1.1337	-2.0064	3.0052
H	-0.3726	-3.5621	2.9640
C	1.0328	-3.1869	5.0871
C	1.9217	1.8202	4.5904
C	2.5822	0.8241	5.3246
C	2.1431	-0.5044	5.1993
C	1.0572	-0.8081	4.3455
C	0.4660	0.2884	3.6647
N	0.8837	1.5655	3.7763
H	2.2350	2.8514	4.6606
H	2.6566	-1.2746	5.7537
H	-0.3796	0.1529	3.0084

H	0.4368	-4.0918	4.9623
H	2.0759	-3.4475	4.9038
H	0.9275	-2.8729	6.1265
H	3.4123	1.0750	5.9670

Molecule 13 OPLS3e

OPLS3e energy = 177.415 kJ/mol

C	-1.1132	-1.0714	0.3036
C	-3.2321	-2.9334	0.5175
C	-2.3385	-2.7512	1.5655
C	-1.3002	-1.8392	1.4618
H	-4.0370	-3.6490	0.6088
H	-2.4456	-3.3248	2.4743
O	-0.4507	-1.7115	2.5259
C	-2.0307	-1.2341	-0.7872
C	-3.0965	-2.1826	-0.6727
C	-3.9992	-2.3541	-1.7524
C	-3.8569	-1.6061	-2.9172
C	-2.8311	-0.6771	-3.0368
C	-1.9295	-0.4834	-1.9946
H	-4.8029	-3.0714	-1.6659
H	-4.5503	-1.7444	-3.7346
H	-2.7321	-0.0981	-3.9444
H	-1.1448	0.2516	-2.1089
C	2.2297	1.6444	-0.0500
C	0.0416	-0.1186	0.2085
C	1.1383	-0.4326	-0.6095
C	2.2155	0.4438	-0.7582
H	3.0625	2.3237	-0.1690
O	1.1371	-1.6258	-1.2869

C	3.3898	0.1062	-1.6839
C	1.1605	2.0024	0.7988
C	0.0446	1.1192	0.9296
C	-1.0301	1.5209	1.7743
C	-0.9877	2.7301	2.4628
C	0.1065	3.5760	2.3340
C	1.1732	3.2275	1.5117
H	-1.8949	0.8824	1.8831
H	-1.8134	3.0151	3.0994
H	0.1247	4.5132	2.8718
H	2.0173	3.8944	1.4083
H	0.1947	-1.0213	2.3495
H	0.3096	-2.0880	-1.1189
N	3.7405	1.2186	-2.5857
H	4.2405	-0.1627	-1.0552
H	3.1762	-0.7830	-2.2798
C	5.1305	1.2574	-3.0478
C	0.8581	2.7003	-5.2471
C	1.9886	3.4742	-4.9859
C	2.9465	2.9731	-4.1010
C	2.7711	1.7208	-3.4945
C	1.5961	1.0230	-3.8221
N	0.6499	1.4875	-4.6790
H	0.0900	3.0502	-5.9220
H	3.8098	3.5744	-3.8682
H	1.3934	0.0509	-3.4052
H	5.8255	1.0083	-2.2444
H	5.4219	2.2350	-3.4312
H	5.2799	0.5426	-3.8592
H	2.1183	4.4416	-5.4479

Molecule 13 OPLS

OPLS energy = 81.392 kJ/mol

C	0.0099	-0.5261	2.4092
C	-2.2053	-0.2571	4.0637
C	-1.0117	-0.6964	4.5203
C	0.0709	-0.8292	3.7246
H	-3.0330	-0.1726	4.7522
H	-0.9135	-0.9526	5.5650
O	1.2314	-1.2794	4.2806
C	-1.2540	-0.0440	1.8647
C	-2.3746	0.0758	2.7719
C	-3.6042	0.5343	2.2798
C	-3.7292	0.8563	0.9791
C	-2.6406	0.7419	0.0931
C	-1.4389	0.3068	0.5158
H	-4.4533	0.6306	2.9406
H	-4.6809	1.2086	0.6079
H	-2.7717	1.0079	-0.9461
H	-0.6322	0.2355	-0.1990
C	3.4390	-1.0270	-0.0884
C	1.2212	-0.6853	1.5372
C	1.4339	-1.8454	0.8729
C	2.5216	-2.0156	0.0739
H	4.2944	-1.1749	-0.7281
O	0.5309	-2.8620	1.0135
C	2.6983	-3.3475	-0.6530
C	3.3172	0.1632	0.5266
C	2.1812	0.3999	1.3868
C	2.0626	1.6476	2.0234

C	2.9995	2.5935	1.8252
C	4.1060	2.3602	0.9859
C	4.2635	1.1827	0.3538
H	1.2292	1.8693	2.6740
H	2.8998	3.5497	2.3187
H	4.8404	3.1404	0.8460
H	5.1194	1.0275	-0.2871
H	1.9635	-1.3159	3.6592
H	-0.2110	-2.6362	1.5823
N	3.9120	-3.4721	-1.4686
H	2.7012	-4.1413	0.0950
H	1.8223	-3.5281	-1.2766
C	5.0615	-4.0809	-0.8079
C	3.8838	-2.5173	-5.5260
C	4.8756	-3.3657	-5.0612
C	4.9003	-3.6949	-3.7156
C	3.9263	-3.1645	-2.8746
C	2.9743	-2.3207	-3.4529
N	2.9414	-1.9926	-4.7461
H	3.8731	-2.2672	-6.5775
H	5.6665	-4.3637	-3.3587
H	2.2187	-1.8852	-2.8186
H	5.1039	-5.1427	-1.0512
H	5.9804	-3.5932	-1.1342
H	4.9893	-3.9655	0.2738
H	5.6152	-3.7658	-5.7381

Molecule 14 MM2

MM2 energy = 208.83 kJ/mol

C	-0.9851	-5.1369	5.7956

C	-2.1289	-4.3613	6.0244
C	-2.5493	-3.3956	5.1081
C	-1.8046	-3.2186	3.9487
C	-0.6766	-3.9772	3.7124
C	-0.2489	-4.9420	4.6199
P	-0.4304	-6.4335	6.9573
H	-2.7357	-4.5033	6.9324
H	-3.4518	-2.7894	5.2900
O	-2.0109	-2.3603	2.9214
O	-0.1246	-3.6227	2.5268
C	3.3587	-7.1433	3.7821
C	3.3378	-6.0891	4.6867
C	2.1703	-5.3976	4.9369
C	0.9796	-5.7308	4.2979
C	0.9782	-6.7905	3.3818
C	2.1693	-7.4857	3.1364
H	4.2934	-7.6948	3.5888
O	4.3482	-5.5797	5.4310
O	2.4011	-4.4207	5.8471
P	-0.6019	-7.2380	2.5808
H	2.1947	-8.3327	2.4329
C	-0.9787	-2.6269	1.9809
C	3.7689	-4.5427	6.2122
H	-0.3900	-1.6960	1.8126
H	-1.4373	-3.0224	1.0451
H	4.2912	-3.5839	5.9896
H	3.8282	-4.8264	7.2885
C	-1.2858	-4.2028	10.9227
C	-2.2616	-5.0508	10.4029
C	-2.0308	-5.7384	9.2118

C	-0.8222	-5.5896	8.5268
C	0.1483	-4.7329	9.0539
C	-0.0790	-4.0441	10.2446
H	-1.4686	-3.6583	11.8645
H	-3.2200	-5.1759	10.9347
H	-2.8291	-6.3933	8.8273
H	1.1111	-4.5887	8.5344
H	0.6961	-3.3720	10.6501
C	-3.7131	-9.6880	6.7037
C	-2.7068	-9.7464	7.6652
C	-1.7371	-8.7463	7.7226
C	-1.7597	-7.6715	6.8287
C	-2.7685	-7.6290	5.8629
C	-3.7403	-8.6271	5.8014
H	-4.4788	-10.4802	6.6521
H	-2.6752	-10.5877	8.3780
H	-0.9468	-8.8208	8.4889
H	-2.8186	-6.8182	5.1197
H	-4.5287	-8.5818	5.0311
C	1.0228	-8.1653	-1.6539
C	0.6711	-6.8709	-1.2772
C	0.1818	-6.6257	0.0052
C	0.0347	-7.6673	0.9257
C	0.3947	-8.9610	0.5399
C	0.8845	-9.2097	-0.7419
H	1.4109	-8.3618	-2.6676
H	0.7810	-6.0398	-1.9942
H	-0.0877	-5.5917	0.2804
H	0.3074	-9.8105	1.2361
H	1.1659	-10.2353	-1.0351

C	-1.6673	-11.3742	4.3665
C	-2.3906	-10.8343	3.3054
C	-2.0400	-9.5902	2.7829
C	-0.9611	-8.8700	3.3044
C	-0.2492	-9.4178	4.3746
C	-0.5964	-10.6615	4.9009
H	-1.9453	-12.3558	4.7858
H	-3.2444	-11.3901	2.8825
H	-2.6342	-9.1822	1.9476
H	0.5934	-8.8821	4.8385
H	-0.0271	-11.0797	5.7483

Molecule 14 MM3

MM3 energy = 90.679 kJ/mol

C	3.5882	-14.0823	-17.0266
C	4.2816	-13.1477	-17.8121
C	5.5763	-12.7458	-17.4711
C	6.1604	-13.2899	-16.3373
C	5.4836	-14.2079	-15.5588
C	4.1940	-14.6266	-15.8803
P	1.8790	-14.5999	-17.5032
H	3.8097	-12.7252	-18.7129
H	6.1260	-12.0143	-18.0850
O	7.3877	-13.0459	-15.8275
O	6.2517	-14.5866	-14.5112
C	2.1552	-17.4184	-13.3364
C	1.9273	-16.0535	-13.2479
C	2.5907	-15.1663	-14.0722
C	3.5091	-15.6032	-15.0249
C	3.7516	-16.9847	-15.1253

C	3.0749	-17.8806	-14.2822
H	1.6198	-18.1171	-12.6737
O	1.0914	-15.3957	-12.4148
O	2.2087	-13.8981	-13.7953
P	4.9590	-17.6233	-16.3705
H	3.2605	-18.9630	-14.3641
C	7.4860	-13.8801	-14.6683
C	1.2313	-14.0126	-12.7566
H	7.6852	-13.2594	-13.7692
H	8.3213	-14.6019	-14.7912
H	1.5536	-13.4319	-11.8664
H	0.2578	-13.6093	-13.1078
C	2.2835	-16.9214	-21.5004
C	3.4362	-16.5863	-20.7900
C	3.3366	-15.8832	-19.5881
C	2.0863	-15.5083	-19.0920
C	0.9343	-15.8566	-19.8027
C	1.0308	-16.5579	-21.0059
H	2.3629	-17.4821	-22.4468
H	4.4272	-16.8861	-21.1704
H	4.2532	-15.6427	-19.0272
H	-0.0564	-15.5776	-19.4081
H	0.1174	-16.8294	-21.5613
C	0.0567	-10.4726	-18.5774
C	0.0930	-10.9240	-17.2578
C	0.6459	-12.1717	-16.9639
C	1.1639	-12.9733	-17.9838
C	1.1262	-12.5158	-19.3032
C	0.5736	-11.2683	-19.6003
H	-0.3795	-9.4868	-18.8116

H	-0.3136	-10.2960	-16.4472
H	0.6768	-12.5281	-15.9214
H	1.5361	-13.1332	-20.1178
H	0.5469	-10.9116	-20.6440
C	2.4907	-20.0568	-19.4554
C	1.8204	-19.2803	-18.5101
C	2.5443	-18.5450	-17.5698
C	3.9402	-18.5846	-17.5666
C	4.6063	-19.3557	-18.5232
C	3.8851	-20.0936	-19.4635
H	1.9181	-20.6325	-20.2017
H	0.7183	-19.2367	-18.5112
H	2.0033	-17.9205	-16.8420
H	5.7083	-19.3783	-18.5359
H	4.4176	-20.6999	-20.2155
C	7.2235	-20.7312	-13.7778
C	7.5770	-19.3817	-13.7597
C	6.8789	-18.4686	-14.5521
C	5.8270	-18.8974	-15.3649
C	5.4764	-20.2496	-15.3780
C	6.1729	-21.1653	-14.5868
H	7.7736	-21.4542	-13.1521
H	8.4062	-19.0359	-13.1197
H	7.1563	-17.4020	-14.5367
H	4.6453	-20.6024	-16.0087
H	5.8914	-22.2320	-14.6006

Molecule 14 MMFF

MMFF energy = 637.6 kJ/mol

C	-5.8980	-0.7051	-0.1794

C	-5.3098	0.1206	0.8176
C	-3.9308	0.3422	0.8747
C	-3.1464	-0.2679	-0.0730
C	-3.6841	-1.0735	-1.0493
C	-5.0525	-1.3283	-1.1436
P	-7.7197	-0.9735	-0.2231
H	-5.9278	0.6151	1.5644
H	-3.4957	0.9748	1.6400
O	-1.7941	-0.1588	-0.1628
O	-2.7176	-1.5741	-1.8767
C	-6.5761	-3.7675	-4.3751
C	-6.5110	-2.3975	-4.4444
C	-6.0093	-1.6434	-3.4095
C	-5.5545	-2.2079	-2.2179
C	-5.6029	-3.6271	-2.1142
C	-6.1150	-4.3806	-3.2067
H	-6.9692	-4.3524	-5.1986
O	-6.9177	-1.6419	-5.4994
O	-6.0225	-0.3099	-3.7075
P	-4.9951	-4.4759	-0.5982
H	-6.1687	-5.4664	-3.1514
C	-1.5313	-0.9901	-1.3089
C	-6.6599	-0.3162	-4.9964
H	-0.8525	-1.7968	-1.0089
H	-1.0367	-0.3836	-2.0763
H	-7.6112	0.2241	-4.9234
H	-6.0134	0.2079	-5.7092
C	-9.0334	-0.3419	4.1833
C	-9.3467	0.6326	3.2399
C	-8.9624	0.4702	1.9044

C	-8.2531	-0.6663	1.4900
C	-7.9531	-1.6413	2.4588
C	-8.3371	-1.4810	3.7927
H	-9.3341	-0.2149	5.2197
H	-9.8938	1.5229	3.5393
H	-9.2285	1.2555	1.2017
H	-7.4118	-2.5446	2.1811
H	-8.0919	-2.2495	4.5208
C	-9.5074	2.5209	-2.6978
C	-10.2435	1.3716	-2.4290
C	-9.6762	0.3385	-1.6777
C	-8.3647	0.4344	-1.1771
C	-7.6401	1.5991	-1.4652
C	-8.2062	2.6341	-2.2174
H	-9.9457	3.3260	-3.2813
H	-11.2595	1.2746	-2.8025
H	-10.2742	-0.5507	-1.4841
H	-6.6183	1.7260	-1.1195
H	-7.6237	3.5274	-2.4280
C	-8.7040	-5.7936	1.8592
C	-7.4142	-5.9446	2.3562
C	-6.3186	-5.5446	1.5859
C	-6.4875	-4.9947	0.3004
C	-7.7971	-4.8460	-0.1751
C	-8.8954	-5.2430	0.5956
H	-9.5594	-6.0973	2.4566
H	-7.2563	-6.3686	3.3442
H	-5.3201	-5.6674	2.0035
H	-7.9991	-4.4065	-1.1474
H	-9.9021	-5.1113	0.2070

C	-3.0466	-8.3073	-2.3566
C	-2.4597	-7.0566	-2.5198
C	-3.0708	-5.9224	-1.9786
C	-4.2813	-6.0154	-1.2671
C	-4.8540	-7.2862	-1.1127
C	-4.2425	-8.4223	-1.6536
H	-2.5734	-9.1910	-2.7763
H	-1.5266	-6.9593	-3.0684
H	-2.5917	-4.9548	-2.1232
H	-5.7902	-7.4216	-0.5778
H	-4.7050	-9.3975	-1.5239

Molecule 14 OPLS-2005

OPLS-2005 energy = 637.6 kJ/mol

C	-3.7580	3.4031	0.1560
C	-3.2224	3.5432	1.4667
C	-2.6680	2.4438	2.1576
C	-2.6633	1.2037	1.5070
C	-3.1752	1.0543	0.2362
C	-3.7311	2.1306	-0.4802
P	-4.5205	4.8700	-0.7471
H	-3.2292	4.5060	1.9555
H	-2.2623	2.5518	3.1525
O	-2.1981	0.0116	1.9582
O	-3.0509	-0.2430	-0.1482
C	-5.1949	1.6310	-4.5264
C	-3.9213	2.1426	-4.2478
C	-3.4674	2.2761	-2.9539
C	-4.2533	1.9263	-1.8407
C	-5.5468	1.3915	-2.0899

C	-6.0020	1.2522	-3.4313
H	-5.5427	1.5294	-5.5436
O	-2.9709	2.5754	-5.1145
O	-2.2095	2.7866	-2.9697
P	-6.6218	0.8789	-0.6336
H	-6.9867	0.8576	-3.6329
C	-2.4235	-0.9046	0.9188
C	-1.8954	3.0036	-4.3186
H	-3.0617	-1.7140	1.2747
H	-1.4714	-1.3165	0.5822
H	-1.7176	4.0674	-4.4798
H	-0.9967	2.4439	-4.5790
C	-0.8103	6.7267	-2.9680
C	-0.6139	6.0411	-1.7547
C	-1.7129	5.4802	-1.0753
C	-3.0189	5.5950	-1.6010
C	-3.2026	6.2824	-2.8190
C	-2.1073	6.8481	-3.5004
H	0.0322	7.1597	-3.4877
H	0.3797	5.9442	-1.3418
H	-1.5450	4.9578	-0.1461
H	-4.1961	6.3757	-3.2330
H	-2.2638	7.3742	-4.4310
C	-5.3487	7.8627	2.7926
C	-4.3794	8.1919	1.8267
C	-4.1171	7.3097	0.7600
C	-4.8163	6.0868	0.6487
C	-5.7894	5.7725	1.6202
C	-6.0560	6.6508	2.6879
H	-5.5507	8.5395	3.6102

H	-3.8366	9.1230	1.9009
H	-3.3732	7.5857	0.0276
H	-6.3324	4.8419	1.5429
H	-6.8032	6.3931	3.4243
C	-9.1231	4.7754	0.2144
C	-8.5660	4.5819	-1.0632
C	-7.8172	3.4212	-1.3382
C	-7.6086	2.4449	-0.3382
C	-8.1727	2.6507	0.9383
C	-8.9294	3.8058	1.2156
H	-9.6970	5.6661	0.4256
H	-8.7139	5.3238	-1.8346
H	-7.4046	3.2899	-2.3266
H	-8.0250	1.9112	1.7122
H	-9.3563	3.9501	2.1975
C	-9.6959	-2.0012	-2.7373
C	-8.3445	-2.3658	-2.5931
C	-7.4438	-1.4890	-1.9580
C	-7.8766	-0.2397	-1.4645
C	-9.2372	0.1137	-1.6109
C	-10.1417	-0.7607	-2.2447
H	-10.3893	-2.6720	-3.2237
H	-7.9976	-3.3172	-2.9697
H	-6.4058	-1.7716	-1.8541
H	-9.6030	1.0593	-1.2391
H	-11.1789	-0.4779	-2.3514

Molecule 14 OPLS3e

OPLS3e energy = 274.209 kJ/mol

C	1.5802	1.2903	-0.2944

C	0.8611	2.0866	0.6193
C	-0.4969	1.8605	0.8743
C	-1.1122	0.8284	0.1817
C	-0.4283	0.0459	-0.7050
C	0.9285	0.2369	-0.9726
P	3.3887	1.6721	-0.6180
H	1.3643	2.8927	1.1298
H	-1.0419	2.4691	1.5791
O	-2.4004	0.4049	0.2641
O	-1.2741	-0.9005	-1.1991
C	3.0227	-2.3527	-3.7324
C	3.2166	-2.4152	-2.3606
C	2.5482	-1.5924	-1.4990
C	1.6328	-0.6365	-1.9432
C	1.4247	-0.5376	-3.3364
C	2.1200	-1.3957	-4.2117
H	3.5485	-3.0115	-4.4061
O	4.0177	-3.2576	-1.6574
O	2.9094	-1.9052	-0.2235
P	0.2581	0.7486	-4.0479
H	1.9598	-1.3109	-5.2751
C	-2.5558	-0.5142	-0.7835
C	4.0079	-2.7674	-0.3437
H	-3.0711	-0.0301	-1.6166
H	-3.1354	-1.3799	-0.4600
H	4.9262	-2.2043	-0.1604
H	3.9402	-3.5838	0.3767
C	3.3876	5.7865	-2.7896
C	2.1890	5.0866	-2.6692
C	2.1706	3.8584	-2.0125

C	3.3457	3.3316	-1.4709
C	4.5440	4.0319	-1.6150
C	4.5682	5.2628	-2.2667
H	3.4028	6.7360	-3.3053
H	1.2793	5.4809	-3.0978
H	1.2452	3.3055	-1.9546
H	5.4546	3.6090	-1.2170
H	5.4982	5.8019	-2.3728
C	4.7649	2.3537	3.7688
C	4.5547	3.4660	2.9559
C	4.1438	3.2877	1.6365
C	3.9453	2.0005	1.1305
C	4.1553	0.8947	1.9533
C	4.5653	1.0667	3.2733
H	5.0823	2.4900	4.7930
H	4.7066	4.4619	3.3459
H	3.9704	4.1517	1.0148
H	3.9888	-0.0969	1.5577
H	4.7239	0.2062	3.9069
C	3.0013	3.7585	-6.2973
C	3.5794	2.8462	-5.4172
C	2.7730	1.9315	-4.7441
C	1.3918	1.9227	-4.9533
C	0.8215	2.8541	-5.8218
C	1.6230	3.7685	-6.5015
H	3.6264	4.4726	-6.8140
H	4.6448	2.8565	-5.2396
H	3.2239	1.2518	-4.0370
H	-0.2494	2.8603	-5.9613
H	1.1765	4.4854	-7.1748

C	-1.8766	-1.9040	-7.2116
C	-0.8953	-1.0129	-7.6418
C	-0.2423	-0.2055	-6.7129
C	-0.5708	-0.2873	-5.3573
C	-1.5516	-1.1846	-4.9371
C	-2.2073	-1.9949	-5.8610
H	-2.3833	-2.5322	-7.9304
H	-0.6393	-0.9492	-8.6894
H	0.5251	0.4738	-7.0489
H	-1.7920	-1.2498	-3.8857
H	-2.9647	-2.6901	-5.5292

Molecule 15 AMBER

AMBER energy = -107.382 kJ/mol

C	2.4745	-6.4955	0.9909
C	3.6288	-5.9728	0.3666
C	4.6554	-5.4524	1.1797
C	4.5267	-5.4309	2.5787
C	3.3687	-5.9409	3.1850
C	2.3436	-6.4743	2.3894
H	1.6758	-6.9387	0.4134
N	3.7979	-5.9534	-1.0512
H	5.5583	-5.0588	0.7352
H	5.3198	-5.0249	3.1904
H	3.2658	-5.9259	4.2612
H	1.4543	-6.8750	2.8549
H	4.6248	-5.5042	-1.4078
S	2.5331	-5.8482	-2.0551
O	1.9479	-7.1703	-2.1265
O	3.1007	-5.3009	-3.2658

C	-0.6387	-2.9145	-0.5420
C	0.7070	-2.7316	-0.1743
C	1.6749	-3.6325	-0.6472
C	1.3191	-4.7166	-1.4693
C	-0.0313	-4.8873	-1.8252
C	-1.0119	-3.9905	-1.3660
H	-1.3823	-2.2149	-0.1831
H	2.7069	-3.4774	-0.3734
H	-0.3026	-5.7197	-2.4615
H	-2.0473	-4.1263	-1.6478
S	1.1825	-1.3977	0.8618
O	0.0275	-0.9115	1.5866
N	2.2676	-1.8886	1.9563
O	1.9163	-0.4467	0.0584
H	3.0207	-1.2337	2.0834
C	1.3395	-4.0839	5.4925
C	2.5197	-3.3298	5.3989
C	2.8096	-2.6194	4.2211
C	1.9265	-2.6415	3.1224
C	0.7468	-3.4119	3.2275
C	0.4552	-4.1256	4.4030
H	1.1103	-4.6269	6.3995
H	3.2039	-3.2916	6.2349
H	3.7227	-2.0443	4.1738
H	0.0352	-3.4653	2.4185
H	-0.4572	-4.7011	4.4694

Molecule 15 MM2

MM2 energy = 67.155 kJ/mol

C	-3.6529	4.2119	-0.3328

C	-3.5513	3.8863	1.0233
C	-2.5382	3.0066	1.4166
C	-1.6593	2.4625	0.4804
C	-1.7790	2.7896	-0.8682
C	-2.7778	3.6715	-1.2737
H	-4.4375	4.9063	-0.6776
N	-4.5039	4.4770	1.9089
H	-2.4031	2.7097	2.4679
H	-0.8659	1.7689	0.8068
H	-1.0841	2.3583	-1.6084
H	-2.8750	3.9411	-2.3389
H	-5.2416	5.0527	1.4986
S	-4.5892	4.2748	3.5359
O	-4.7305	2.9096	3.7465
O	-5.5027	5.2440	3.9458
C	-0.5713	5.6531	5.1081
C	-1.2688	6.4440	4.1924
C	-2.5093	6.0008	3.7263
C	-3.0554	4.7857	4.1486
C	-2.3429	4.0092	5.0650
C	-1.1069	4.4419	5.5411
H	0.4084	5.9689	5.5044
H	-3.0693	6.6222	3.0065
H	-2.7396	3.0460	5.4275
H	-0.5498	3.8227	6.2646
S	-0.6268	7.9512	3.6406
O	0.7533	7.9358	3.7946
N	-0.9026	7.9942	2.0232
O	-1.4754	8.9312	4.1519
H	-1.4015	8.8192	1.6849

C	0.3178	5.2666	-0.9850
C	-0.4572	6.3834	-1.2875
C	-0.8445	7.2567	-0.2725
C	-0.4706	7.0352	1.0567
C	0.2993	5.9047	1.3468
C	0.6910	5.0278	0.3356
H	0.6294	4.5739	-1.7849
H	-0.7621	6.5761	-2.3300
H	-1.4553	8.1366	-0.5360
H	0.6258	5.6726	2.3722
H	1.2998	4.1408	0.5800

Molecule 15 MMFF

MMFF energy = -150.809 kJ/mol

C	0.4430	1.4032	0.2846
C	1.8303	1.4163	0.4403
C	2.4597	2.5273	1.0006
C	1.7012	3.6248	1.4109
C	0.3149	3.6105	1.2609
C	-0.3142	2.5009	0.6971
H	-0.0633	0.5439	-0.1461
N	2.5770	0.2712	0.0468
H	3.5408	2.5550	1.1131
H	2.1935	4.4916	1.8447
H	-0.2751	4.4661	1.5806
H	-1.3952	2.4909	0.5801
H	2.1899	-0.3189	-0.6876
S	3.4083	-0.6167	1.2150
O	4.4110	0.2166	1.8420
O	3.7630	-1.8777	0.5968

C	0.1925	-1.5612	4.3178
C	0.2754	-2.3004	3.1331
C	1.2474	-2.0097	2.1727
C	2.1495	-0.9722	2.4211
C	2.0949	-0.2430	3.6137
C	1.1094	-0.5363	4.5580
H	-0.5735	-1.7821	5.0590
H	1.3009	-2.5806	1.2484
H	2.8066	0.5569	3.8099
H	1.0559	0.0348	5.4826
S	-0.8949	-3.6010	2.8093
O	-1.1311	-3.6044	1.3802
N	-0.0158	-4.9988	3.1519
O	-1.9881	-3.4675	3.7474
H	0.4613	-5.3689	2.3316
C	1.9616	-5.2984	6.8589
C	2.6920	-5.2707	5.6713
C	2.0296	-5.1848	4.4456
C	0.6353	-5.1234	4.4106
C	-0.0952	-5.1550	5.5979
C	0.5687	-5.2426	6.8225
H	2.4778	-5.3670	7.8135
H	3.7781	-5.3147	5.7007
H	2.6138	-5.1570	3.5301
H	-1.1819	-5.1228	5.5822
H	-0.0023	-5.2703	7.7473

Molecule 15 OPLS-2005

OPLS-2005 energy = -150.809 kJ/mol

C	1.3963	-1.6046	1.1537

C	2.3030	-0.5388	0.9620
C	2.9398	-0.3906	-0.2880
C	2.6672	-1.2896	-1.3373
C	1.7548	-2.3438	-1.1433
C	1.1200	-2.5006	0.1031
H	0.9159	-1.7592	2.1082
N	2.5963	0.3724	1.9801
H	3.6422	0.4130	-0.4544
H	3.1583	-1.1700	-2.2919
H	1.5439	-3.0335	-1.9482
H	0.4262	-3.3140	0.2577
H	3.3755	0.9917	1.8296
S	1.7659	0.6824	3.4571
O	2.3069	1.9654	3.9242
O	1.8854	-0.5458	4.2555
C	-2.5834	1.2537	2.0842
C	-1.5608	2.0988	1.6053
C	-0.2329	1.9421	2.0380
C	0.0691	0.9139	2.9471
C	-0.9401	0.0578	3.4345
C	-2.2723	0.2310	3.0045
H	-3.5986	1.3938	1.7400
H	0.5455	2.5919	1.6663
H	-0.6849	-0.7261	4.1341
H	-3.0511	-0.4204	3.3755
S	-1.9308	3.3685	0.4038
O	-1.2247	4.6021	0.7741
N	-1.1284	2.8191	-1.0175
O	-3.3668	3.3611	0.0905
H	-0.4574	3.4810	-1.3705

C	-1.1874	-0.9545	-2.9144
C	-2.2330	-0.5984	-2.0420
C	-2.2259	0.6590	-1.4080
C	-1.1679	1.5664	-1.6352
C	-0.1273	1.2068	-2.5169
C	-0.1367	-0.0490	-3.1544
H	-1.1943	-1.9185	-3.4029
H	-3.0472	-1.2859	-1.8650
H	-3.0504	0.9207	-0.7619
H	0.6891	1.8872	-2.7098
H	0.6654	-0.3192	-3.8254

Molecule 15 OPLS3e

OPLS3e energy = -310.569 kJ/mol

C	6.5784	0.2228	-2.8038
C	6.7562	-0.2467	-1.5016
C	8.0150	-0.1986	-0.9028
C	9.1053	0.2902	-1.6188
C	8.9259	0.7463	-2.9234
C	7.6654	0.7215	-3.5170
H	5.6009	0.1918	-3.2646
N	5.6830	-0.7784	-0.7845
H	8.1608	-0.5623	0.1035
H	10.0880	0.3061	-1.1696
H	9.7743	1.1188	-3.4799
H	7.5310	1.0796	-4.5268
H	5.7390	-0.7722	0.2180
S	4.8809	-2.2388	-1.3337
O	4.5288	-2.0543	-2.7437
O	3.8735	-2.5476	-0.3163

C	8.1097	-5.4754	-1.1491
C	8.1944	-4.4422	-2.0867
C	7.2051	-3.4591	-2.1390
C	6.1300	-3.5077	-1.2503
C	6.0435	-4.5358	-0.3073
C	7.0339	-5.5186	-0.2602
H	8.8768	-6.2380	-1.1046
H	7.2715	-2.6595	-2.8685
H	5.2095	-4.5773	0.3817
H	6.9677	-6.3156	0.4669
S	9.5556	-4.3615	-3.2347
O	10.4433	-5.4892	-2.9422
N	8.8459	-4.7055	-4.8018
O	10.0384	-2.9785	-3.2545
H	8.7076	-5.6944	-4.9079
C	5.9432	-2.1551	-6.3782
C	7.2414	-1.6834	-6.1939
C	8.2158	-2.5335	-5.6773
C	7.8885	-3.8500	-5.3495
C	6.5936	-4.3250	-5.5574
C	5.6153	-3.4726	-6.0640
H	5.1812	-1.4932	-6.7647
H	7.4912	-0.6627	-6.4424
H	9.2215	-2.1683	-5.5226
H	6.3323	-5.3416	-5.3037
H	4.6040	-3.8272	-6.2030

Molecule 16 AMBER

AMBER energy = -38.978 kJ/mol

C	-1.9231	-1.0024	0.0463

O	-0.7522	-0.1827	0.0530
N	-0.0983	-2.0578	-0.8906
C	-1.5410	-2.1112	-0.9474
C	-2.0881	-1.6029	1.4505
C	-3.1790	-0.1792	-0.2751
H	-1.9239	-3.0670	-0.5856
C	-2.0231	-1.8866	-2.4035
C	0.3655	-0.9103	-0.3687
O	1.4753	-0.4371	-0.1795
C	0.7343	-3.0966	-1.1152
O	0.2649	-4.2212	-1.2861
C	2.2531	-2.9386	-1.2575
H	2.7063	-2.4317	-0.4077
H	2.6887	-3.9384	-1.2789
C	3.2026	-1.0529	-5.0565
C	3.5494	-0.4192	-3.8518
C	3.2493	-1.0310	-2.6223
C	2.5942	-2.2777	-2.5802
C	2.2599	-2.9085	-3.7953
C	2.5586	-2.3008	-5.0271
H	3.4341	-0.5829	-6.0027
H	4.0478	0.5400	-3.8670
H	3.5222	-0.5321	-1.7025
H	1.7671	-3.8713	-3.7826
H	2.2942	-2.7956	-5.9511
C	-1.2875	-0.7553	-3.1437
C	-1.8889	-3.1622	-3.2458
H	-3.0883	-1.6576	-2.3830
H	-1.7695	-0.5830	-4.1063
H	-1.3126	0.1731	-2.5758

H	-0.2467	-1.0252	-3.3260
H	-2.3937	-3.0206	-4.2019
H	-0.8422	-3.3902	-3.4412
H	-2.3501	-4.0047	-2.7297
H	-2.9674	-2.2472	1.4787
H	-1.2109	-2.1915	1.7214
H	-2.2112	-0.8039	2.1824
H	-4.0478	-0.8351	-0.3365
H	-3.3458	0.5492	0.5189
H	-3.0709	0.3660	-1.2106

Molecule 16 MM2

MM2 energy = -122.554 kJ/mol

C	2.4102	0.9171	3.0058
O	2.5271	0.4603	1.6696
N	3.7695	2.3204	1.8320
C	3.5691	1.9312	3.2092
C	1.0510	1.6469	3.0872
C	2.3661	-0.2974	3.9447
H	3.2020	2.7799	3.8335
C	4.8610	1.3542	3.8407
C	3.2495	1.3643	0.9846
O	3.3783	1.3076	-0.2206
C	4.3336	3.5357	1.4668
O	4.5837	4.3674	2.3242
C	4.6328	3.8632	0.0086
H	3.6933	3.7810	-0.5841
H	4.9431	4.9314	-0.0653
C	7.8709	1.5229	-1.6271
C	6.7001	1.7038	-2.3610

C	5.6551	2.4614	-1.8338
C	5.7630	3.0417	-0.5673
C	6.9462	2.8646	0.1543
C	7.9935	2.1072	-0.3679
H	8.6985	0.9235	-2.0427
H	6.6007	1.2472	-3.3603
H	4.7369	2.5969	-2.4290
H	7.0626	3.3247	1.1490
H	8.9201	1.9713	0.2151
C	5.6265	0.3809	2.9248
C	5.8337	2.4461	4.3250
H	4.5633	0.8072	4.7671
H	6.4809	-0.0877	3.4652
H	4.9879	-0.4472	2.5466
H	6.0472	0.9032	2.0364
H	6.6596	2.0065	4.9309
H	6.3142	2.9805	3.4764
H	5.3212	3.1982	4.9679
H	0.8562	2.0147	4.1206
H	1.0088	2.5259	2.4035
H	0.2100	0.9723	2.8062
H	2.2907	0.0248	5.0082
H	1.4795	-0.9357	3.7272
H	3.2527	-0.9599	3.8405

Molecule 16 MM3

MM3 energy = 58.805 kJ/mol

C	3.5332	4.3606	1.1568
O	3.2597	3.2629	0.2686
N	3.4259	5.0780	-1.0211

C	3.9717	5.5383	0.2356
C	2.1970	4.7066	1.8350
C	4.5252	3.9089	2.2308
H	3.4825	6.4741	0.5921
C	5.4983	5.7611	0.1619
C	3.0918	3.7428	-0.9839
O	2.6778	3.0501	-1.8786
C	3.1227	5.9927	-2.0278
O	3.1421	7.1874	-1.8241
C	2.7852	5.4687	-3.4225
H	1.9977	4.6873	-3.3863
H	2.3385	6.2951	-4.0222
C	6.3611	4.1545	-5.4671
C	6.2046	5.4906	-5.1021
C	5.0450	5.9044	-4.4463
C	4.0272	4.9899	-4.1414
C	4.1943	3.6519	-4.5207
C	5.3534	3.2361	-5.1768
H	7.2770	3.8255	-5.9857
H	6.9980	6.2214	-5.3320
H	4.9340	6.9649	-4.1626
H	3.4066	2.9116	-4.3020
H	5.4714	2.1783	-5.4660
C	6.2395	4.6708	-0.6399
C	5.8565	7.1365	-0.4343
H	5.8969	5.7642	1.2043
H	6.0147	3.6446	-0.2799
H	5.9782	4.7002	-1.7204
H	7.3428	4.8006	-0.5724
H	5.6298	7.1975	-1.5201

H	5.3109	7.9629	0.0734
H	6.9428	7.3523	-0.3236
H	2.3105	5.5478	2.5547
H	1.4190	5.0077	1.0982
H	1.7877	3.8423	2.4042
H	4.7701	4.7337	2.9361
H	4.1065	3.0785	2.8421
H	5.4799	3.5312	1.8085

Molecule 16 MMFF

MMFF energy = 1.07 kJ/mol

C	2.5386	1.1014	1.0933
O	3.9356	1.3572	0.8761
N	3.2566	0.0416	-0.8307
C	2.0474	0.6312	-0.2859
C	2.4285	-0.0310	2.1328
C	1.8837	2.3546	1.6692
H	1.2725	-0.1354	-0.1565
C	1.4685	1.7317	-1.2308
C	4.3571	0.5454	-0.1476
O	5.5396	0.3601	-0.3781
C	3.2120	-1.0539	-1.6772
O	2.1642	-1.6856	-1.8542
C	4.4841	-1.4778	-2.3942
H	5.3311	-1.5337	-1.7068
H	4.3440	-2.5109	-2.7400
C	5.2779	1.0196	-5.8231
C	6.1353	1.0516	-4.7252
C	5.8857	0.2397	-3.6175
C	4.7732	-0.6108	-3.5962

C	3.9193	-0.6378	-4.7090
C	4.1705	0.1744	-5.8157
H	5.4746	1.6516	-6.6849
H	7.0008	1.7091	-4.7294
H	6.5669	0.2802	-2.7691
H	3.0490	-1.2922	-4.7180
H	3.5020	0.1462	-6.6722
C	2.4992	2.7394	-1.7557
C	0.7300	1.1155	-2.4271
H	0.7070	2.2885	-0.6711
H	1.9993	3.5325	-2.3229
H	3.0553	3.2204	-0.9476
H	3.2218	2.2649	-2.4280
H	-0.0143	0.3843	-2.0952
H	0.2001	1.8920	-2.9901
H	1.4167	0.6208	-3.1198
H	1.3822	-0.2721	2.3475
H	2.9284	-0.9453	1.7935
H	2.9202	0.2543	3.0701
H	2.3032	2.5891	2.6550
H	2.0733	3.2408	1.0578
H	0.8025	2.2255	1.7819

Molecule 16 MMFF

MMFF energy = -0.343 kJ/mol

C	0.5524	-4.0778	-0.9618
O	0.9319	-2.6975	-0.8320
N	0.1697	-2.9594	-2.9422
C	-0.3766	-4.0719	-2.1873
C	1.8316	-4.8901	-1.2425

C	-0.0481	-4.5579	0.3573
H	-0.2563	-5.0077	-2.7482
C	-1.9014	-3.8873	-1.8974
C	0.8714	-2.1254	-2.0790
O	1.3726	-1.0399	-2.3155
C	0.1150	-2.9197	-4.3258
O	-0.2171	-3.9139	-4.9816
C	0.4801	-1.6282	-5.0402
H	1.3887	-1.1842	-4.6281
H	0.7405	-1.8815	-6.0767
C	-2.8347	1.1354	-5.0970
C	-2.9083	-0.0586	-5.8110
C	-1.8330	-0.9478	-5.7972
C	-0.6725	-0.6523	-5.0662
C	-0.6089	0.5532	-4.3562
C	-1.6856	1.4414	-4.3709
H	-3.6715	1.8287	-5.1081
H	-3.8029	-0.2979	-6.3798
H	-1.9064	-1.8777	-6.3592
H	0.2796	0.8103	-3.7818
H	-1.6253	2.3731	-3.8144
C	-2.2982	-2.5021	-1.3714
C	-2.7567	-4.2074	-3.1311
H	-2.1902	-4.6290	-1.1427
H	-1.7347	-2.2225	-0.4782
H	-2.1444	-1.7241	-2.1266
H	-3.3602	-2.4885	-1.1020
H	-2.4951	-5.1851	-3.5485
H	-3.8184	-4.2386	-2.8615
H	-2.6421	-3.4530	-3.9145

H	2.5561	-4.7649	-0.4295
H	1.6085	-5.9568	-1.3492
H	2.3371	-4.5527	-2.1543
H	-0.8588	-3.9116	0.7043
H	-0.4212	-5.5839	0.2772
H	0.7041	-4.5304	1.1547

Molecule 16 OPLS-2005

OPLS-2005 energy = -0.343 kJ/mol

C	1.8689	1.6196	-2.5569
O	1.9769	0.8240	-3.7176
N	1.3839	2.7245	-4.5455
C	1.8759	3.0220	-3.1965
C	0.5094	1.3051	-1.8969
C	3.0050	1.2858	-1.5704
H	1.1594	3.6420	-2.6529
C	3.2520	3.7784	-3.2159
C	1.5771	1.4228	-4.8552
O	1.3895	0.8372	-5.9196
C	0.7573	3.6542	-5.3111
O	0.3223	4.6956	-4.8120
C	0.6052	3.5047	-6.8378
H	0.1692	2.5484	-7.1214
H	-0.1087	4.2522	-7.1851
C	4.3939	4.2460	-8.8415
C	3.7244	5.2975	-8.1872
C	2.4946	5.0580	-7.5448
C	1.9299	3.7629	-7.5461
C	2.6008	2.7154	-8.2136
C	3.8305	2.9558	-8.8559

H	5.3374	4.4294	-9.3349
H	4.1538	6.2888	-8.1782
H	1.9893	5.8721	-7.0433
H	2.1807	1.7192	-8.2312
H	4.3413	2.1481	-9.3600
C	4.2877	3.2236	-4.2181
C	3.0935	5.2924	-3.4399
H	3.6852	3.6989	-2.2192
H	4.4526	2.1540	-4.0961
H	5.2532	3.7136	-4.0900
H	3.9809	3.3906	-5.2517
H	4.0328	5.8146	-3.2558
H	2.7951	5.5264	-4.4613
H	2.3475	5.7207	-2.7696
H	0.3582	1.9112	-1.0031
H	-0.3281	1.5013	-2.5675
H	0.4482	0.2579	-1.5987
H	2.9807	1.9448	-0.7022
H	2.9131	0.2623	-1.2060
H	3.9910	1.3646	-2.0250

Molecule 16 OPLS3e

OPLS3e energy = -98.642 kJ/mol

C	0.4043	-0.3299	0.1106
O	1.5178	-0.3571	0.9759
N	2.3535	0.3905	-0.9242
C	0.9039	0.6387	-0.9784
C	0.1852	-1.7668	-0.4157
C	-0.8092	0.1163	0.9470
H	0.4978	0.2878	-1.9299

C	0.5254	2.1600	-0.8479
C	2.6842	-0.0519	0.3102
O	3.7796	-0.2251	0.8412
C	3.1406	0.4636	-2.0308
O	2.6529	0.5329	-3.1621
C	4.6797	0.5280	-1.9317
H	5.0655	-0.2145	-1.2355
H	5.1038	0.2468	-2.8966
C	5.9786	4.5089	-0.9153
C	6.1006	3.4829	0.0196
C	5.6893	2.1946	-0.3119
C	5.1566	1.9347	-1.5778
C	5.0367	2.9703	-2.5108
C	5.4483	4.2584	-2.1793
H	6.2980	5.5084	-0.6572
H	6.5108	3.6839	0.9989
H	5.7802	1.4030	0.4193
H	4.6175	2.7761	-3.4887
H	5.3549	5.0591	-2.8988
C	1.2629	2.9296	0.2722
C	0.7003	2.9092	-2.1829
H	-0.5426	2.2140	-0.6325
H	2.3307	3.0194	0.0645
H	0.8711	3.9417	0.3790
H	1.1616	2.4439	1.2423
H	0.2898	3.9181	-2.1287
H	1.7505	3.0043	-2.4614
H	0.1910	2.3967	-3.0001
H	-0.6842	-1.8170	-1.0713
H	1.0419	-2.1350	-0.9810

H	0.0220	-2.4661	0.4051
H	-1.6956	0.2375	0.3248
H	-1.0434	-0.6184	1.7180
H	-0.6323	1.0570	1.4658

Molecule 16 OPLS

OPLS energy = 1.084 kJ/mol

C	-0.9363	-2.8429	2.0625
O	0.0913	-2.9738	1.0695
N	-1.7482	-2.5091	-0.0747
C	-2.0910	-2.1708	1.2977
C	-1.3751	-4.2527	2.4907
C	-0.3749	-2.1462	3.3123
H	-3.0198	-2.6485	1.6100
C	-2.2607	-0.6321	1.4753
C	-0.4727	-2.9456	-0.2084
O	0.2164	-3.3216	-1.1448
C	-2.6504	-2.7467	-1.0648
O	-3.8419	-2.8744	-0.7813
C	-2.2930	-2.7189	-2.5599
H	-1.5462	-3.4594	-2.8386
H	-3.2050	-2.9631	-3.1050
C	-1.1503	1.2931	-3.7158
C	-2.4799	1.0099	-3.3718
C	-2.8497	-0.2930	-3.0071
C	-1.8905	-1.3181	-2.9793
C	-0.5619	-1.0343	-3.3334
C	-0.1925	0.2688	-3.6979
H	-0.8649	2.2966	-3.9976
H	-3.2213	1.7957	-3.3887

H	-3.8760	-0.5056	-2.7416
H	0.1834	-1.8177	-3.3194
H	0.8325	0.4819	-3.9652
C	-1.2093	0.2404	0.7676
C	-3.6463	-0.1498	1.0267
H	-2.2286	-0.3984	2.5379
H	-1.3385	1.2782	1.0742
H	-0.2012	-0.0755	1.0298
H	-1.3258	0.1819	-0.3142
H	-3.7742	0.8950	1.3099
H	-3.7528	-0.2325	-0.0539
H	-4.4230	-0.7399	1.5129
H	-2.1362	-4.1855	3.2687
H	-1.7836	-4.8053	1.6440
H	-0.5181	-4.8022	2.8805
H	0.4034	-2.7677	3.7552
H	0.0688	-1.1847	3.0613
H	-1.1680	-2.0007	4.0461

Molecule 17 MMFF

MMFF energy = 168.858 kJ/mol

C	-8.7954	-4.0081	-11.4706
C	-9.8611	-3.1870	-11.8759
C	-10.6809	-2.6077	-10.8964
C	-10.4587	-2.8603	-9.5346
C	-9.3976	-3.7033	-9.1604
C	-8.5437	-4.2575	-10.1162
H	-8.1486	-4.4439	-12.2320
C	-10.0915	-2.9026	-13.3408
H	-11.5071	-1.9613	-11.1979

C	-11.3671	-2.2778	-8.4780
H	-9.2863	-3.9408	-8.1050
N	-7.4577	-5.1042	-9.8148
F	-12.0039	-1.1454	-8.8878
F	-12.3467	-3.1442	-8.1032
F	-10.7062	-1.9383	-7.3364
F	-9.4094	-1.8045	-13.7652
F	-9.7003	-3.9252	-14.1505
F	-11.4000	-2.6714	-13.6381
H	-7.0951	-5.6547	-10.5868
C	-6.8112	-5.3838	-8.6415
S	-5.7958	-6.7119	-8.6315
N	-7.0197	-4.5262	-7.6048
H	-7.1287	-3.5663	-7.9229
C	-6.4077	-5.6296	-3.9749
C	-7.0237	-5.6461	-5.3725
C	-6.4133	-4.5524	-6.2628
C	-6.6212	-3.1560	-5.5852
C	-5.9664	-3.1445	-4.1917
C	-6.5296	-4.2614	-3.3119
H	-5.3487	-5.9083	-4.0419
H	-6.8965	-6.3850	-3.3492
H	-8.1095	-5.5018	-5.2979
H	-6.8790	-6.6388	-5.8095
H	-5.3412	-4.7483	-6.3848
N	-6.2302	-2.0514	-6.5029
H	-7.7078	-3.0567	-5.4389
H	-4.8810	-3.2771	-4.2758
H	-6.1370	-2.1860	-3.6896
H	-7.5854	-4.0554	-3.0960

H	-6.0049	-4.2711	-2.3498
C	-6.8028	-0.7692	-6.0873
C	-4.7858	-1.9180	-6.6957
H	-6.3972	-0.4156	-5.1338
H	-6.6067	-0.0018	-6.8448
H	-7.8924	-0.8410	-5.9969
H	-4.3390	-2.8508	-7.0522
H	-4.5777	-1.1686	-7.4682
H	-4.2660	-1.6087	-5.7827

Molecule 17 MMFF

MMFF energy = 172.39 kJ/mol

C	6.2567	-3.7274	-1.0632
C	4.8607	-3.7198	-0.9052
C	4.2472	-4.7892	-0.2342
C	5.0135	-5.8337	0.3044
C	6.4076	-5.8123	0.1326
C	7.0333	-4.7814	-0.5717
H	6.7331	-2.9048	-1.5963
C	4.0331	-2.5979	-1.4854
H	3.1620	-4.7984	-0.1161
C	4.3677	-6.9778	1.0499
H	6.9944	-6.5952	0.6093
N	8.4270	-4.6989	-0.7542
F	3.0638	-6.7411	1.3679
F	4.3687	-8.1326	0.3288
F	4.9885	-7.2642	2.2268
F	2.8957	-2.3551	-0.7776
F	4.7016	-1.4127	-1.5352
F	3.6348	-2.8592	-2.7599

H	8.8006	-3.7715	-0.9299
C	9.4085	-5.6466	-0.6541
S	10.9711	-5.1052	-0.4062
N	9.0357	-6.9443	-0.8339
H	8.0913	-7.1060	-1.1526
C	11.4863	-9.7242	-1.8015
C	10.6401	-8.4638	-1.9547
C	9.8471	-8.1614	-0.6729
C	8.9409	-9.3892	-0.3082
C	9.8155	-10.6488	-0.1390
C	10.6426	-10.9265	-1.3946
H	12.2636	-9.5516	-1.0469
H	12.0005	-9.9390	-2.7451
H	9.9493	-8.5846	-2.7994
H	11.2951	-7.6256	-2.2150
H	10.5604	-7.9849	0.1409
N	8.0150	-9.1036	0.8173
H	8.2992	-9.5533	-1.1872
H	10.4969	-10.5297	0.7121
H	9.1967	-11.5292	0.0645
H	9.9709	-11.1955	-2.2193
H	11.2940	-11.7906	-1.2212
C	8.6767	-8.9055	2.1063
C	6.9559	-10.1060	0.9404
H	9.1669	-9.8141	2.4713
H	9.4183	-8.1027	2.0606
H	7.9448	-8.5945	2.8610
H	6.4238	-10.2296	-0.0092
H	7.3333	-11.0834	1.2582
H	6.2085	-9.7804	1.6728

Molecule 17 OPLS-2005

OPLS-2005 energy = 172.39 kJ/mol

C	3.2522	-5.3529	-8.2373
C	3.9249	-5.7253	-9.4204
C	4.4163	-7.0401	-9.5548
C	4.2214	-7.9879	-8.5295
C	3.5493	-7.6044	-7.3495
C	3.0785	-6.2807	-7.1912
H	2.8840	-4.3415	-8.1360
C	4.1340	-4.7092	-10.5319
H	4.9419	-7.3285	-10.4537
C	4.7465	-9.4049	-8.6989
H	3.3939	-8.3384	-6.5704
N	2.3653	-5.8714	-6.0308
F	4.0859	-10.2642	-7.9230
F	4.6131	-9.7957	-9.9662
F	6.0384	-9.4318	-8.3761
F	5.2594	-4.0369	-10.2983
F	4.2291	-5.3141	-11.7158
F	3.1170	-3.8487	-10.5773
H	1.5159	-5.3448	-6.1834
C	2.6648	-6.0635	-4.7225
S	1.5602	-5.6590	-3.5510
N	3.9037	-6.5804	-4.5305
H	4.4309	-6.8553	-5.3449
C	5.6377	-6.4809	-1.1042
C	5.0576	-5.9262	-2.4170
C	4.4566	-7.0638	-3.2675
C	5.5555	-8.1100	-3.5738

C	6.0968	-8.7064	-2.2553
C	6.6712	-7.5920	-1.3627
H	4.8262	-6.8737	-0.4895
H	6.0931	-5.6739	-0.5289
H	5.8378	-5.4100	-2.9776
H	4.2992	-5.1777	-2.1860
H	3.6615	-7.5434	-2.6948
N	5.1038	-9.0979	-4.5680
H	6.3791	-7.5696	-4.0451
H	5.3037	-9.2233	-1.7145
H	6.8780	-9.4414	-2.4446
H	7.5539	-7.1633	-1.8393
H	7.0092	-8.0122	-0.4147
C	6.2103	-9.8761	-5.1176
C	4.0602	-10.0010	-4.0857
H	5.8624	-10.5154	-5.9301
H	6.9827	-9.2235	-5.5269
H	6.6706	-10.5217	-4.3692
H	3.7109	-10.6432	-4.8956
H	4.4189	-10.6476	-3.2840
H	3.1927	-9.4516	-3.7189

Molecule 17 OPLS3e

OPLS3e energy = 163.893 kJ/mol

C	-0.7596	-6.6410	-3.6602
C	0.6050	-6.5009	-3.9432
C	1.0935	-5.2524	-4.3479
C	0.2387	-4.1487	-4.4619
C	-1.1214	-4.3079	-4.1745
C	-1.6320	-5.5580	-3.7888

H	-1.1445	-7.6044	-3.3526
C	1.5527	-7.6938	-3.8113
H	2.1472	-5.1415	-4.5708
C	0.7689	-2.7778	-4.8852
H	-1.7634	-3.4383	-4.2316
N	-2.9877	-5.7841	-3.4784
F	2.0999	-2.7164	-4.8160
F	0.2677	-1.8209	-4.1032
F	0.4040	-2.5398	-6.1444
F	1.6457	-8.3004	-4.9934
F	2.7708	-7.3003	-3.4366
F	1.1038	-8.5746	-2.9155
H	-3.1488	-6.4941	-2.7790
C	-4.1104	-5.1040	-3.8389
S	-5.4761	-5.3750	-2.9305
N	-4.0177	-4.3031	-4.9420
H	-3.1166	-4.2248	-5.3886
C	-7.2237	-3.0224	-6.7578
C	-6.1646	-4.0348	-6.2813
C	-5.0406	-3.3728	-5.4423
C	-4.4100	-2.1508	-6.1762
C	-5.5045	-1.1493	-6.6242
C	-6.5957	-1.8199	-7.4811
H	-7.7949	-2.6703	-5.8964
H	-7.9440	-3.5180	-7.4110
H	-5.7239	-4.5258	-7.1505
H	-6.6575	-4.8202	-5.7065
H	-5.5162	-2.9502	-4.5559
N	-3.2563	-1.5672	-5.4649
H	-4.0041	-2.5739	-7.0988

H	-5.9619	-0.6864	-5.7496
H	-5.0598	-0.3394	-7.2011
H	-6.1655	-2.1492	-8.4288
H	-7.3692	-1.0938	-7.7365
C	-3.5917	-0.9044	-4.2012
C	-2.4352	-0.6930	-6.3052
H	-4.2118	-0.0196	-4.3540
H	-4.1222	-1.5765	-3.5252
H	-2.6864	-0.5879	-3.6798
H	-2.9562	0.2253	-6.5803
H	-1.5183	-0.4086	-5.7856
H	-2.1342	-1.2004	-7.2234

Molecule 18 AMBER

AMBER energy = 88.963 kJ/mol

C	-1.4187	-2.6476	3.9940
C	-1.9537	-2.9363	5.2015
C	0.7767	-2.5448	4.8832
C	-0.0817	-2.4691	3.8359
H	-3.0245	-3.0601	5.2743
O	0.4373	-2.2445	2.5253
C	4.9822	-1.8726	4.3604
C	4.3144	-2.8036	3.6429
C	2.9771	-2.9897	3.7939
C	2.2538	-2.2693	4.6875
H	6.0477	-1.7723	4.2114
O	2.3088	-3.9334	2.9550
C	-1.1983	-3.0890	6.3035
C	0.2393	-2.9097	6.1918
C	1.0283	-3.1065	7.3430

C	0.4526	-3.4270	8.5183
C	-0.9434	-3.5802	8.6274
C	-1.7456	-3.4199	7.5570
H	2.1034	-3.0096	7.2980
H	1.0743	-3.5706	9.3911
H	-1.3767	-3.8371	9.5841
H	-2.8123	-3.5527	7.6690
C	2.9337	-1.2162	5.4379
C	4.3643	-1.0625	5.2378
C	5.0488	-0.0703	5.9642
C	4.3801	0.7295	6.8174
C	2.9894	0.5970	6.9983
C	2.2864	-0.3391	6.3315
H	6.1144	0.0606	5.8398
H	4.9174	1.4895	7.3676
H	2.4755	1.2580	7.6824
H	1.2194	-0.4010	6.4898
P	1.3044	-3.3984	1.8212
O	2.2126	-2.6981	0.7094
H	2.7963	-2.1243	1.2067
O	0.4437	-4.4901	1.3162
C	-4.0513	-2.3692	0.5661
C	-4.0175	-3.5428	1.3376
C	-3.1630	-3.6284	2.4508
C	-2.3334	-2.5466	2.8041
C	-2.3761	-1.3743	2.0248
C	-3.2290	-1.2837	0.9110
H	-4.7066	-2.3021	-0.2913
H	-4.6456	-4.3819	1.0735
H	-3.1351	-4.5384	3.0332

H	-1.7448	-0.5368	2.2870
H	-3.2501	-0.3786	0.3206
C	6.5123	-5.1679	0.7543
C	6.5460	-3.7650	0.6912
C	5.8336	-3.0010	1.6322
C	5.0789	-3.6280	2.6430
C	5.0547	-5.0352	2.6988
C	5.7656	-5.8032	1.7604
H	7.0581	-5.7571	0.0304
H	7.1173	-3.2728	-0.0833
H	5.8610	-1.9225	1.5702
H	4.4823	-5.5290	3.4718
H	5.7361	-6.8824	1.8138

Molecule 18 MM2

MM2 energy = 196.264 kJ/mol

C	0.7038	0.0781	2.0837
C	0.4517	1.1726	1.2589
C	-0.7273	-1.2836	0.6944
C	0.1383	-1.1676	1.7911
H	0.9058	2.1484	1.4995
O	0.4775	-2.2454	2.5495
C	-2.7161	-4.9936	-0.1402
C	-1.3254	-4.9490	-0.0637
C	-0.6770	-3.7547	0.2681
C	-1.4406	-2.5958	0.4669
H	-3.2141	-5.9426	-0.4001
O	0.6734	-3.7593	0.4359
C	-0.3489	1.0420	0.1251
C	-0.9358	-0.1944	-0.1668

C	-1.7059	-0.2963	-1.3318
C	-1.9121	0.8002	-2.1672
C	-1.3412	2.0297	-1.8529
C	-0.5575	2.1447	-0.7076
H	-2.1661	-1.2497	-1.6362
H	-2.5251	0.6952	-3.0785
H	-1.5011	2.9016	-2.5097
H	-0.1011	3.1218	-0.4745
C	-2.8433	-2.6551	0.4441
C	-3.4815	-3.8600	0.1289
C	-4.8760	-3.9379	0.0823
C	-5.6580	-2.8209	0.3644
C	-5.0369	-1.6222	0.7021
C	-3.6458	-1.5470	0.7429
H	-5.3797	-4.8857	-0.1731
H	-6.7587	-2.8868	0.3298
H	-5.6472	-0.7342	0.9391
H	-3.2065	-0.5793	1.0328
P	1.2582	-3.4329	1.8660
O	1.1233	-4.7198	2.7617
H	0.1994	-4.8911	2.7952
O	2.6725	-3.0951	1.7306
C	3.3277	0.5394	5.4705
C	2.1077	-0.1183	5.6161
C	1.2586	-0.2663	4.5201
C	1.6161	0.2386	3.2672
C	2.8399	0.8995	3.1326
C	3.6924	1.0489	4.2258
H	4.0008	0.6570	6.3365
H	1.8131	-0.5199	6.6004

H	0.2928	-0.7792	4.6611
H	3.1524	1.3013	2.1544
H	4.6577	1.5691	4.1056
C	0.9495	-8.5458	-0.7353
C	1.3248	-7.3558	-1.3557
C	0.5876	-6.1922	-1.1393
C	-0.5309	-6.2021	-0.3020
C	-0.9009	-7.4010	0.3128
C	-0.1659	-8.5665	0.0998
H	1.5320	-9.4671	-0.9051
H	2.2050	-7.3355	-2.0205
H	0.8953	-5.2646	-1.6498
H	-1.7741	-7.4367	0.9854
H	-0.4658	-9.5060	0.5942

Molecule 18 MMFF

MMFF energy = 250.29 kJ/mol

C	0.7086	0.0737	2.0979
C	0.4871	1.1844	1.2765
C	-0.7818	-1.2469	0.6376
C	0.0949	-1.1324	1.7409
H	0.9646	2.1289	1.5374
O	0.3821	-2.2606	2.4738
C	-2.7455	-5.0103	-0.1115
C	-1.3472	-4.9540	-0.0893
C	-0.7558	-3.7145	0.1934
C	-1.5032	-2.5368	0.4173
H	-3.2275	-5.9620	-0.3347
O	0.6144	-3.6607	0.2978
C	-0.2972	1.1001	0.1161

C	-0.9309	-0.1216	-0.2286
C	-1.6703	-0.1471	-1.4371
C	-1.8057	0.9829	-2.2466
C	-1.1981	2.1737	-1.8772
C	-0.4468	2.2299	-0.7053
H	-2.1522	-1.0639	-1.7720
H	-2.3844	0.9258	-3.1649
H	-1.2994	3.0565	-2.5027
H	0.0326	3.1706	-0.4403
C	-2.9275	-2.6393	0.4629
C	-3.5372	-3.8872	0.1735
C	-4.9369	-4.0099	0.1835
C	-5.7529	-2.9253	0.4966
C	-5.1782	-1.7035	0.8123
C	-3.7883	-1.5651	0.8003
H	-5.4079	-4.9640	-0.0458
H	-6.8335	-3.0396	0.5041
H	-5.8051	-0.8543	1.0717
H	-3.3811	-0.5923	1.0694
P	1.2732	-3.3990	1.7499
O	0.9079	-4.7362	2.5682
H	1.5682	-5.4287	2.3845
O	2.7527	-3.1686	1.7277
C	3.2038	0.4604	5.5516
C	1.8535	0.7954	5.6242
C	1.0399	0.6660	4.4962
C	1.5660	0.2002	3.2814
C	2.9277	-0.1307	3.2244
C	3.7410	-0.0035	4.3528
H	3.8369	0.5563	6.4297

H	1.4312	1.1533	6.5594
H	-0.0141	0.9254	4.5693
H	3.3634	-0.5015	2.2982
H	4.7922	-0.2731	4.2934
C	0.9873	-8.4945	-0.7299
C	1.1205	-7.4072	-1.5901
C	0.3569	-6.2554	-1.3853
C	-0.5508	-6.1715	-0.3176
C	-0.6710	-7.2789	0.5388
C	0.0919	-8.4313	0.3349
H	1.5804	-9.3912	-0.8907
H	1.8181	-7.4534	-2.4226
H	0.4746	-5.4156	-2.0680
H	-1.3567	-7.2431	1.3836
H	-0.0139	-9.2782	1.0081

Molecule 18 OPLS-2005

OPLS-2005 energy = 250.29 kJ/mol

C	-1.8101	-2.7088	3.2585
C	-2.6135	-3.1950	4.3069
C	0.1657	-2.8398	4.7446
C	-0.4234	-2.5586	3.4862
H	-3.6750	-3.3146	4.1457
O	0.3891	-2.2029	2.4481
C	4.4155	-2.1986	5.2210
C	3.9422	-3.0567	4.2110
C	2.5458	-3.2244	4.0662
C	1.6197	-2.5772	4.9248
H	5.4804	-2.0649	5.3442
O	2.0797	-3.9863	3.0317

C	-2.0482	-3.5530	5.5441
C	-0.6560	-3.3921	5.7673
C	-0.1195	-3.8059	7.0116
C	-0.9506	-4.3358	8.0167
C	-2.3320	-4.4682	7.7914
C	-2.8789	-4.0798	6.5552
H	0.9380	-3.7253	7.2147
H	-0.5250	-4.6422	8.9615
H	-2.9705	-4.8750	8.5624
H	-3.9396	-4.1959	6.3850
C	2.1219	-1.6629	5.8941
C	3.5224	-1.4929	6.0466
C	4.0357	-0.6075	7.0174
C	3.1624	0.1327	7.8343
C	1.7725	-0.0066	7.6763
C	1.2575	-0.8932	6.7118
H	5.1027	-0.4850	7.1354
H	3.5584	0.8129	8.5745
H	1.0977	0.5680	8.2945
H	0.1849	-0.9684	6.6127
P	1.3404	-3.3170	1.7508
O	2.5061	-2.4223	1.0866
H	2.7217	-2.7615	0.2345
O	0.6811	-4.2620	0.8109
C	-3.5984	-1.7722	-0.5516
C	-2.7763	-0.8290	0.0922
C	-2.1920	-1.1372	1.3361
C	-2.4183	-2.3875	1.9541
C	-3.2466	-3.3241	1.2964
C	-3.8327	-3.0217	0.0518

H	-4.0448	-1.5399	-1.5077
H	-2.5913	0.1307	-0.3679
H	-1.5619	-0.4060	1.8217
H	-3.4241	-4.2915	1.7434
H	-4.4570	-3.7521	-0.4419
C	6.7433	-5.0287	1.5893
C	6.8379	-3.6428	1.8134
C	5.9218	-3.0039	2.6717
C	4.8995	-3.7358	3.3166
C	4.8190	-5.1272	3.0834
C	5.7317	-5.7713	2.2257
H	7.4445	-5.5213	0.9309
H	7.6119	-3.0682	1.3255
H	5.9994	-1.9376	2.8281
H	4.0466	-5.7084	3.5669
H	5.6539	-6.8357	2.0571

Molecule 18 OPLS3e

OPLS3e energy = 84.344 kJ/mol

C	-1.6695	-2.7014	3.8441
C	-2.3009	-3.0628	5.0317
C	0.5122	-3.0584	4.8911
C	-0.2702	-2.7444	3.7592
H	-3.3798	-3.0440	5.0877
O	0.3508	-2.4214	2.5765
C	4.8267	-2.7978	4.7364
C	4.1462	-3.6322	3.8516
C	2.7459	-3.6810	3.8932
C	2.0121	-2.9335	4.8331
H	5.9060	-2.7600	4.7127

O	2.0733	-4.4716	2.9981
C	-1.5651	-3.4937	6.1539
C	-0.1384	-3.5057	6.0909
C	0.5726	-3.9726	7.2355
C	-0.0996	-4.3808	8.3841
C	-1.4868	-4.3465	8.4372
C	-2.2217	-3.9110	7.3394
H	1.6517	-4.0158	7.2133
H	0.4617	-4.7295	9.2393
H	-1.9964	-4.6677	9.3345
H	-3.3014	-3.8964	7.3836
C	2.7101	-2.0230	5.6978
C	4.1374	-1.9764	5.6522
C	4.8422	-1.1023	6.5184
C	4.1535	-0.2742	7.3987
C	2.7656	-0.2895	7.4356
C	2.0470	-1.1414	6.6017
H	5.9219	-1.0751	6.4852
H	4.6991	0.3926	8.0511
H	2.2396	0.3665	8.1145
H	0.9679	-1.1281	6.6415
P	1.1990	-3.6407	1.9101
O	2.3392	-2.8551	1.0930
H	2.5381	-3.3421	0.2765
O	0.4138	-4.5128	1.0012
C	-4.1128	-1.4393	0.5652
C	-3.3225	-2.5789	0.4367
C	-2.5268	-2.9895	1.5029
C	-2.5114	-2.2646	2.6983
C	-3.3151	-1.1248	2.8153

C	-4.1135	-0.7100	1.7518
H	-4.7283	-1.1189	-0.2630
H	-3.3223	-3.1449	-0.4837
H	-1.9258	-3.8807	1.4013
H	-3.3101	-0.5527	3.7317
H	-4.7259	0.1749	1.8455
C	6.3775	-5.9391	1.0102
C	6.6325	-4.5772	1.1488
C	5.9058	-3.8309	2.0734
C	4.9223	-4.4376	2.8646
C	4.6785	-5.8077	2.7110
C	5.4017	-6.5579	1.7873
H	6.9384	-6.5203	0.2921
H	7.3871	-4.1007	0.5397
H	6.0981	-2.7716	2.1667
H	3.9184	-6.2910	3.3090
H	5.2027	-7.6140	1.6743

Molecule 19 AMBER

AMBER energy = 84.344 kJ/mol

C	-1.9339	1.0150	-5.1313
N	-1.0172	0.3778	-4.3898
C	0.4196	0.2712	-4.5890
C	0.7796	-0.6151	-5.8045
H	0.7890	1.2797	-4.7717
C	0.9948	-0.1865	-3.2473
H	0.2265	-1.5457	-5.6924
C	2.2713	-0.9902	-5.8390
N	0.3762	-0.0252	-7.1070
C	0.5496	-1.3316	-2.5323

C	2.0102	0.5857	-2.6553
C	2.7329	-1.0322	-7.3034
H	2.4259	-1.9563	-5.3555
H	2.8707	-0.2474	-5.3108
C	1.3339	1.0153	-7.5550
C	0.3616	-1.1078	-8.1178
C	-0.4681	-2.1978	-3.0009
C	1.1516	-1.6048	-1.2774
C	2.5402	0.2064	-1.4141
H	2.3900	1.4782	-3.1309
C	2.7273	0.4176	-7.8022
H	3.7361	-1.4555	-7.3832
C	1.7318	-1.8119	-8.1763
H	1.4076	1.8109	-6.8143
H	0.9727	1.4673	-8.4804
H	-0.4233	-1.8283	-7.8812
H	0.1324	-0.6840	-9.0972
C	-0.9106	-3.3027	-2.2573
H	-0.9634	-2.0526	-3.9430
C	0.7061	-2.7126	-0.5373
N	2.1248	-0.8643	-0.7394
H	3.3201	0.8115	-0.9699
H	3.4820	1.0025	-7.2732
H	2.9616	0.4403	-8.8682
H	2.0880	-1.7917	-9.2087
C	1.6412	-3.2876	-7.7660
C	-0.3132	-3.5518	-1.0092
O	-1.9065	-4.0803	-2.7920
H	1.1562	-2.9165	0.4247
H	1.0022	-3.8222	-8.4703

H	2.6355	-3.7357	-7.7943
H	1.2296	-3.4047	-6.7644
H	-0.6142	-4.3796	-0.3841
C	-2.3030	-5.2808	-2.1142
H	-3.0376	-5.8014	-2.7284
H	-1.4481	-5.9431	-1.9713
H	-2.7600	-5.0448	-1.1523
N	-3.2202	1.0357	-4.7236
O	-1.5888	1.5405	-6.2161
H	-1.3367	0.0162	-3.5035
H	-3.9141	1.4876	-5.3052
C	-3.5914	0.0442	-3.8019
C	-4.2378	-1.1220	-4.2460
C	-3.3002	0.1728	-2.4299
C	-4.5546	-2.1716	-3.3603
H	-4.4944	-1.2406	-5.2904
C	-3.5643	-0.8723	-1.5213
H	-2.8276	1.0732	-2.0580
C	-4.2114	-2.0300	-1.9998
H	-4.4371	-2.8294	-1.3099
C	-3.1337	-0.7371	-0.0632
C	-5.2320	-3.4281	-3.9000
F	-3.1368	-1.9416	0.6149
F	-1.8420	-0.2447	-0.0153
F	-3.9788	0.1478	0.5709
F	-5.4710	-4.3898	-2.9371
F	-6.4338	-3.0797	-4.4811
F	-4.4195	-3.9830	-4.8711
H	-0.5420	0.3987	-7.0116

Molecule 19 MM2

MM2 energy = 84.344 kJ/mol

C	3.2191	-2.4720	1.8856
N	1.8825	-2.5293	1.8653
C	1.0868	-3.6175	2.4412
C	1.6357	-5.0503	2.2159
H	1.1037	-3.4038	3.5332
C	-0.3529	-3.4598	1.9741
H	2.1745	-5.0213	1.2396
C	0.5786	-6.1686	2.1499
N	2.6049	-5.4543	3.3035
C	-0.7502	-3.4439	0.6261
C	-1.3557	-3.3085	2.9394
C	1.1062	-7.4450	2.8186
H	0.2889	-6.3674	1.0910
H	-0.3599	-5.8772	2.6741
C	1.9181	-5.8637	4.5791
C	3.4600	-6.5913	2.8098
C	0.1435	-3.5861	-0.4386
C	-2.1101	-3.2755	0.3294
C	-2.6789	-3.1531	2.5344
H	-1.1209	-3.3130	4.0167
C	1.1427	-7.1667	4.3286
H	0.4339	-8.3122	2.6042
C	2.5444	-7.7491	2.3682
H	1.2323	-5.0660	4.9520
H	2.6663	-6.0233	5.3944
H	4.1117	-6.2559	1.9662
H	4.1649	-6.9244	3.6109
C	-0.2346	-3.5582	-1.7839

H	1.2165	-3.7255	-0.2485
C	-2.5156	-3.2491	-1.0048
N	-3.0505	-3.1370	1.2614
H	-3.4953	-3.0334	3.2830
H	0.1067	-7.0858	4.7367
H	1.6190	-8.0236	4.8623
H	2.8752	-8.6791	2.8943
C	2.6478	-8.0539	0.8648
C	-1.5974	-3.3850	-2.0429
O	0.7418	-3.6951	-2.7333
H	-3.5838	-3.1156	-1.2483
H	3.6814	-8.3705	0.5934
H	1.9587	-8.8807	0.5765
H	2.3974	-7.1715	0.2349
H	-1.9840	-3.3517	-3.0738
C	0.3898	-3.5987	-4.0916
H	1.3334	-3.7043	-4.6723
H	-0.2959	-4.4343	-4.3617
H	-0.0497	-2.5954	-4.2971
N	3.8947	-1.4357	1.3753
O	3.8526	-3.4142	2.4204
H	1.4306	-1.6571	1.5863
H	4.8918	-1.4624	1.6000
C	3.4694	-0.3961	0.6310
C	2.5379	-0.5460	-0.3974
C	4.0066	0.8719	0.8601
C	2.1171	0.5416	-1.1652
H	2.1290	-1.5418	-0.6273
C	3.6092	1.9778	0.1046
H	4.7545	1.0050	1.6598

C	2.6586	1.8017	-0.9037
H	2.3352	2.6654	-1.5048
C	4.2182	3.3243	0.4105
C	1.0962	0.3058	-2.2505
F	3.8913	3.7067	1.7078
F	5.6049	3.2568	0.3211
F	3.7858	4.3263	-0.4498
F	-0.0174	-0.3378	-1.7152
F	0.6571	1.4755	-2.8568
F	1.6274	-0.5189	-3.2365
H	3.2417	-4.6442	3.5174

Molecule 19 MM3

MM3 energy = 84.344 kJ/mol

C	-0.1933	-0.8819	1.3876
N	-0.8505	-0.0929	0.4673
C	-2.2554	0.2861	0.6070
C	-3.3052	-0.8588	0.5647
H	-2.3772	0.8061	1.5866
C	-2.5401	1.3076	-0.4845
H	-3.1652	-1.4001	-0.3994
C	-4.7507	-0.2685	0.5488
N	-3.2559	-1.9094	1.6504
C	-2.4094	1.0534	-1.8690
C	-2.9605	2.5938	-0.1070
C	-5.6827	-1.0786	1.4648
H	-5.1467	-0.2442	-0.4913
H	-4.7479	0.7863	0.9026
C	-3.6925	-1.3626	2.9788
C	-4.1538	-3.0514	1.2759

C	-1.9599	-0.1762	-2.3777
C	-2.7407	2.0742	-2.7844
C	-3.2596	3.5502	-1.0729
H	-3.0663	2.8738	0.9542
C	-5.1709	-0.9046	2.9049
H	-6.7297	-0.7020	1.3845
C	-5.6242	-2.5774	1.1141
H	-3.0552	-0.5061	3.2938
H	-3.5851	-2.1364	3.7741
H	-3.8031	-3.5290	0.3325
H	-4.1075	-3.8494	2.0534
C	-1.8529	-0.4113	-3.7494
H	-1.6586	-0.9971	-1.7132
C	-2.6313	1.8358	-4.1552
N	-3.1623	3.2923	-2.3874
H	-3.5950	4.5625	-0.7884
H	-5.2593	0.1606	3.2192
H	-5.7967	-1.4980	3.6099
H	-6.2596	-3.1315	1.8480
C	-6.1696	-2.8806	-0.2914
C	-2.1945	0.6048	-4.6423
O	-1.4043	-1.6481	-4.1210
H	-2.8943	2.6446	-4.8574
H	-6.1984	-3.9763	-0.4853
H	-7.2080	-2.5002	-0.4154
H	-5.5504	-2.4237	-1.0937
H	-2.1195	0.4593	-5.7320
C	-1.1893	-1.9412	-5.4943
H	-0.4368	-1.2517	-5.9320
H	-0.8009	-2.9768	-5.5869

H	-2.1404	-1.8788	-6.0641
N	1.1695	-1.0663	1.2407
O	-0.7849	-1.3673	2.3194
H	-0.2553	0.4883	-0.1438
H	1.6672	-1.4987	2.0301
C	2.0815	-0.7024	0.2551
C	1.7857	-0.8474	-1.1029
C	3.3360	-0.2029	0.6188
C	2.7064	-0.4853	-2.0986
H	0.8135	-1.2735	-1.4028
C	4.2871	0.1662	-0.3451
H	3.5778	-0.0883	1.6899
C	3.9532	0.0190	-1.7000
H	4.6957	0.3087	-2.4660
C	5.6496	0.7154	0.0422
C	2.3422	-0.6489	-3.5632
F	6.6105	-0.0818	-0.4632
F	5.7955	1.9482	-0.4810
F	5.7986	0.7872	1.3792
F	1.2797	0.1360	-3.8309
F	3.3507	-0.3147	-4.3884
F	1.9855	-1.9280	-3.7963
H	-2.3049	-2.2800	1.7339

Molecule 19 MMFF

MMFF energy = 84.344 kJ/mol

C	0.2247	0.1798	0.0147
N	-0.8926	0.9245	-0.2693
C	-2.0702	0.9880	0.6128
C	-2.8239	-0.3886	0.8187

H	-1.6736	1.2997	1.5866
C	-2.9948	2.0913	0.1004
H	-2.8907	-0.8817	-0.1573
C	-4.2402	-0.3042	1.4299
N	-2.0495	-1.3865	1.6978
C	-3.6247	2.0737	-1.1803
C	-3.2399	3.1776	0.9556
C	-4.4722	-1.4653	2.4209
H	-4.9880	-0.3279	0.6287
H	-4.3887	0.6432	1.9609
C	-2.1084	-1.0682	3.1740
C	-2.5516	-2.7916	1.4431
C	-3.4318	1.0781	-2.1714
C	-4.5026	3.1393	-1.4845
C	-4.1077	4.1680	0.5451
H	-2.7747	3.2630	1.9334
C	-3.5597	-1.2008	3.6338
H	-5.5201	-1.4913	2.7398
C	-4.0489	-2.8250	1.8049
H	-1.7201	-0.0566	3.3196
H	-1.4478	-1.7717	3.6917
H	-2.3449	-3.0330	0.3951
H	-1.9641	-3.4646	2.0772
C	-4.0960	1.1104	-3.4011
H	-2.7378	0.2574	-2.0215
C	-5.1544	3.1551	-2.7088
N	-4.7403	4.1513	-0.6402
H	-4.3295	5.0252	1.1760
H	-3.8667	-0.2869	4.1562
H	-3.6480	-2.0156	4.3625

H	-4.1941	-3.6065	2.5632
C	-4.9083	-3.2189	0.6038
C	-4.9668	2.1545	-3.6666
O	-3.7997	0.0691	-4.2340
H	-5.8321	3.9787	-2.9298
H	-4.6474	-4.2268	0.2631
H	-5.9698	-3.2211	0.8720
H	-4.7725	-2.5392	-0.2429
H	-5.5111	2.2343	-4.6023
C	-4.4245	0.0739	-5.5147
H	-5.5129	0.0001	-5.4213
H	-4.1349	0.9586	-6.0913
H	-4.0754	-0.8087	-6.0592
N	1.2223	0.3856	-0.9030
O	0.2410	-0.5945	0.9646
H	-0.6927	1.7844	-0.7680
H	0.9682	0.9043	-1.7338
C	2.5221	-0.1636	-0.8949
C	3.0472	-0.9025	0.1666
C	3.3243	0.0691	-2.0185
C	4.3508	-1.4296	0.1106
H	2.4689	-1.0848	1.0685
C	4.6367	-0.4304	-2.0936
H	2.9401	0.6464	-2.8594
C	5.1380	-1.1862	-1.0241
H	6.1532	-1.5857	-1.0748
C	5.4740	-0.1857	-3.3287
C	4.8886	-2.2196	1.2818
F	6.8108	-0.2113	-3.0831
F	5.2430	-1.1167	-4.2924

F	5.2176	1.0222	-3.9022
F	3.9730	-3.0995	1.7766
F	5.9906	-2.9556	0.9785
F	5.2375	-1.4182	2.3239
H	-1.0450	-1.3407	1.4214

Molecule 19 MMFF

MMFF energy = 84.344 kJ/mol

C	0.4865	-0.4808	-0.3590
N	-0.2242	0.1291	-1.3584
C	-1.6259	0.5607	-1.2465
C	-2.6626	-0.5932	-0.9318
H	-1.6330	1.2492	-0.3931
C	-1.9884	1.3552	-2.4998
H	-2.4066	-1.4541	-1.5589
C	-4.1521	-0.2453	-1.1479
N	-2.5642	-1.1286	0.5078
C	-2.0130	0.8026	-3.8157
C	-2.3252	2.7079	-2.3322
C	-5.0211	-0.8693	-0.0344
H	-4.4740	-0.6038	-2.1325
H	-4.3114	0.8392	-1.1489
C	-3.1771	-0.2116	1.5409
C	-3.1922	-2.5036	0.5883
C	-1.6444	-0.5250	-4.1508
C	-2.4290	1.6519	-4.8666
C	-2.7044	3.4434	-3.4357
H	-2.3029	3.1953	-1.3617
C	-4.6778	-0.1200	1.2674
H	-6.0847	-0.7472	-0.2670

C	-4.6715	-2.3655	0.1808
H	-2.6839	0.7614	1.4683
H	-2.9650	-0.6355	2.5281
H	-2.6153	-3.1702	-0.0615
H	-3.0848	-2.8480	1.6226
C	-1.7087	-1.0093	-5.4607
H	-1.2740	-1.2169	-3.4015
C	-2.4884	1.1545	-6.1602
N	-2.7717	2.9324	-4.6767
H	-2.9769	4.4925	-3.3517
H	-4.9770	0.9327	1.1974
H	-5.2378	-0.5431	2.1101
H	-5.2852	-2.7468	1.0085
C	-5.0009	-3.2272	-1.0380
C	-2.1392	-0.1631	-6.4693
O	-1.3241	-2.3122	-5.6033
H	-2.8150	1.8171	-6.9607
H	-4.8379	-4.2863	-0.8109
H	-6.0501	-3.1047	-1.3259
H	-4.3790	-2.9759	-1.9025
H	-2.2103	-0.4811	-7.5047
C	-1.3413	-2.8456	-6.9246
H	-2.3572	-2.8511	-7.3330
H	-0.6536	-2.2997	-7.5789
H	-0.9977	-3.8830	-6.8699
N	1.7921	-0.7065	-0.7102
O	-0.0616	-0.7971	0.6911
H	0.3006	0.4899	-2.1458
H	2.0669	-0.4374	-1.6452
C	2.8065	-1.2961	0.0751

C	4.0605	-1.4687	-0.5241
C	2.6317	-1.7056	1.3981
C	5.1346	-2.0403	0.1810
H	4.2252	-1.1579	-1.5558
C	3.6836	-2.2981	2.1213
H	1.6820	-1.5761	1.9094
C	4.9325	-2.4579	1.5041
H	5.7570	-2.9101	2.0596
C	3.4625	-2.7207	3.5558
C	6.4721	-2.2347	-0.4969
F	2.2353	-3.2816	3.7489
F	4.3662	-3.6373	3.9937
F	3.5331	-1.6707	4.4170
F	7.5155	-2.2593	0.3742
F	6.5250	-3.4021	-1.1921
F	6.7508	-1.2489	-1.3937
H	-1.5522	-1.2157	0.7453

Molecule 19 OPLS-2005

OPLS-2005 energy = 84.344 kJ/mol

C	-0.9021	-0.7353	-5.6757
N	-0.6994	-1.8526	-4.9775
C	-0.8167	-3.2600	-5.3519
C	-2.1653	-3.6598	-6.0422
H	-0.0015	-3.4449	-6.0518
C	-0.5259	-4.0925	-4.1009
H	-2.9543	-3.2748	-5.3956
C	-2.4283	-5.1914	-6.1566
N	-2.3819	-3.0352	-7.3854
C	-1.1676	-3.8961	-2.8417

C	0.4851	-5.0696	-4.1811
C	-3.0998	-5.5085	-7.5149
H	-3.0425	-5.5343	-5.3221
H	-1.4985	-5.7576	-6.0838
C	-1.5938	-3.7112	-8.4566
C	-3.8310	-3.0826	-7.7246
C	-2.1999	-2.9450	-2.6381
C	-0.7404	-4.6895	-1.7368
C	0.8176	-5.8112	-3.0372
H	1.0325	-5.2632	-5.0932
C	-2.0522	-5.1863	-8.6027
H	-3.3977	-6.5576	-7.5699
C	-4.3134	-4.5654	-7.7466
H	-0.5273	-3.6599	-8.2364
H	-1.7262	-3.1786	-9.4002
H	-4.4019	-2.4869	-7.0099
H	-3.9949	-2.6145	-8.6972
C	-2.7736	-2.7336	-1.3735
H	-2.5869	-2.3294	-3.4296
C	-1.3355	-4.4850	-0.4800
N	0.2169	-5.6245	-1.8539
H	1.5908	-6.5659	-3.0705
H	-1.1959	-5.8589	-8.5241
H	-2.4727	-5.3570	-9.5958
H	-4.7056	-4.7726	-8.7449
C	-5.4739	-4.8280	-6.7723
C	-2.3336	-3.5154	-0.2832
O	-3.7315	-1.7500	-1.2957
H	-0.9983	-5.0839	0.3531
H	-5.7914	-5.8712	-6.8133

H	-5.2098	-4.6059	-5.7382
H	-6.3438	-4.2196	-7.0244
H	-2.7407	-3.3934	0.7099
C	-4.3618	-1.5278	-0.0402
H	-3.6443	-1.2028	0.7147
H	-4.8776	-2.4218	0.3131
H	-5.1069	-0.7390	-0.1452
N	-0.8490	0.3989	-4.9633
O	-1.2175	-0.7597	-6.8604
H	-0.4477	-1.7153	-4.0084
H	-1.0283	1.2299	-5.5121
C	-1.0345	0.5259	-3.5570
C	-2.1553	1.2314	-3.0747
C	-0.1192	-0.0335	-2.6369
C	-2.3968	1.3320	-1.6878
H	-2.8616	1.6849	-3.7562
C	-0.3595	0.0490	-1.2487
H	0.7715	-0.5437	-2.9769
C	-1.4943	0.7410	-0.7791
H	-1.6752	0.8113	0.2838
C	0.5999	-0.6182	-0.2778
C	-3.6339	2.0657	-1.1973
F	-0.0037	-0.8947	0.8777
F	1.0502	-1.7575	-0.8050
F	1.6296	0.1944	-0.0541
F	-4.6940	1.6662	-1.8997
F	-3.8652	1.8298	0.0939
F	-3.4632	3.3733	-1.3786
H	-2.0642	-2.0591	-7.3248

Molecule 19 OPLS3e

OPLS3e energy = 84.344 kJ/mol

C	0.6092	1.5319	-0.5341
N	-0.6025	0.9598	-0.4625
C	-1.9202	1.5603	-0.7289
C	-2.3173	2.7640	0.2043
H	-1.8795	1.9176	-1.7587
C	-2.9738	0.4400	-0.7093
H	-1.9719	2.5369	1.2145
C	-3.8287	3.1663	0.2837
N	-1.5109	4.0414	-0.1812
C	-3.2307	-0.3580	0.4616
C	-3.7265	0.1911	-1.8626
C	-4.0485	4.7013	0.1568
H	-4.2686	2.7754	1.2029
H	-4.3947	2.6649	-0.5026
C	-2.0503	4.7206	-1.4736
C	-1.6243	5.0694	0.9815
C	-2.5392	-0.2237	1.6970
C	-4.2506	-1.3585	0.3716
C	-4.6939	-0.8074	-1.8491
H	-3.5676	0.7562	-2.7705
C	-3.5367	5.1144	-1.2499
H	-5.1015	4.9680	0.2910
C	-3.1366	5.4145	1.2079
H	-1.9181	4.0297	-2.3078
H	-1.4154	5.5890	-1.6698
H	-1.1396	4.6268	1.8564
H	-1.0332	5.9430	0.6932
C	-2.8307	-1.0301	2.7900

H	-1.7504	0.4970	1.8127
C	-4.5396	-2.1686	1.4940
N	-4.9650	-1.5702	-0.7666
H	-5.2815	-1.0211	-2.7319
H	-4.1569	4.6578	-2.0245
H	-3.6570	6.1907	-1.3935
H	-3.2459	6.4934	1.0663
C	-3.5821	5.1484	2.6625
C	-3.8341	-1.9978	2.6829
O	-2.0772	-0.7996	3.9204
H	-5.3098	-2.9244	1.4153
H	-3.0111	5.7544	3.3685
H	-4.6337	5.3995	2.8112
H	-3.4493	4.1067	2.9569
H	-4.0756	-2.6359	3.5186
C	-2.2267	-1.6619	5.0452
H	-1.5193	-1.3680	5.8210
H	-3.2283	-1.5955	5.4728
H	-2.0148	-2.7012	4.7877
N	1.6772	0.7203	-0.4535
O	0.7649	2.7349	-0.7059
H	-0.5904	-0.0480	-0.5153
H	2.5496	1.1728	-0.6802
C	1.7315	-0.6660	-0.2229
C	1.0030	-1.2913	0.7990
C	2.5937	-1.4246	-1.0171
C	1.1057	-2.6729	1.0024
H	0.3665	-0.7097	1.4522
C	2.7078	-2.8070	-0.8282
H	3.1728	-0.9469	-1.7970

C	1.9572	-3.4238	0.1810
H	2.0409	-4.4937	0.3297
C	3.6455	-3.6234	-1.7183
C	0.3004	-3.3403	2.1175
F	3.9612	-4.7934	-1.1617
F	3.0358	-3.8550	-2.8795
F	4.7723	-2.9497	-1.9513
F	-0.9709	-3.4355	1.7325
F	0.7494	-4.5649	2.3944
F	0.3610	-2.6117	3.2321
H	-0.5344	3.7464	-0.3234

Molecule 19 OPLS

OPLS energy = 84.344 kJ/mol

C	-0.1655	-1.4813	-1.2370
N	-1.1236	-0.5719	-1.0366
C	-2.5123	-0.7577	-0.6355
C	-3.3381	-1.4755	-1.7337
H	-2.5049	-1.3825	0.2546
C	-3.0219	0.6173	-0.1998
H	-3.0764	-0.9749	-2.6637
C	-4.8685	-1.3643	-1.6065
N	-2.9519	-2.9014	-1.8776
C	-2.9495	1.7899	-0.9762
C	-3.5761	0.7152	1.0720
C	-5.4896	-2.6935	-2.0756
H	-5.2271	-0.5326	-2.2130
H	-5.1643	-1.1923	-0.5715
C	-3.6014	-3.7777	-0.8707
C	-3.3603	-3.3569	-3.2257

C	-2.4070	1.8244	-2.2815
C	-3.4320	2.9776	-0.4148
C	-4.0342	1.9437	1.5191
H	-3.6550	-0.1417	1.7251
C	-5.1303	-3.7346	-1.0060
H	-6.5731	-2.6025	-2.1535
C	-4.8832	-3.1925	-3.4063
H	-3.3266	-3.4772	0.1390
H	-3.2634	-4.8047	-1.0110
H	-2.8219	-2.7966	-3.9910
H	-3.1011	-4.4090	-3.3470
C	-2.3045	3.0192	-3.0045
H	-2.0270	0.9534	-2.7833
C	-3.3502	4.1721	-1.1470
N	-3.9702	3.0589	0.7991
H	-4.4631	2.0059	2.5103
H	-5.5761	-3.4648	-0.0483
H	-5.5025	-4.7163	-1.3000
H	-5.3045	-4.1747	-3.6229
C	-5.2307	-2.2953	-4.6010
C	-2.7857	4.2064	-2.4296
O	-1.7173	2.9422	-4.2401
H	-3.7204	5.0850	-0.7031
H	-4.8596	-2.7575	-5.5159
H	-6.3132	-2.1895	-4.6725
H	-4.7800	-1.3088	-4.4969
H	-2.7362	5.1574	-2.9380
C	-1.5469	4.1430	-5.0207
H	-1.0655	3.8788	-5.9620
H	-2.5134	4.5982	-5.2384

H	-0.9082	4.8496	-4.4892
N	1.0750	-1.0860	-1.5584
O	-0.4395	-2.7001	-1.1848
H	-0.8160	0.3917	-1.0580
H	1.7398	-1.8017	-1.8281
C	1.2380	0.2719	-1.9788
C	1.1132	1.3379	-1.0701
C	1.5292	0.5388	-3.3238
C	1.2082	2.6699	-1.5154
H	0.9379	1.1279	-0.0235
C	1.6491	1.8630	-3.7803
H	1.6470	-0.2732	-4.0279
C	1.4882	2.9275	-2.8714
H	1.5797	3.9493	-3.2112
C	1.9402	2.1050	-5.2551
C	1.0043	3.8153	-0.5317
F	1.9691	3.4406	-5.5989
F	3.1567	1.5287	-5.5539
F	0.9674	1.4657	-5.9978
F	0.0706	3.4487	0.4182
F	2.2046	4.0782	0.0884
F	0.5392	4.9498	-1.1644
H	-1.9336	-2.9604	-1.7471

Molecule 20 AMBER

AMBER energy = 128.896 kJ/mol

C	-2.4257	1.5988	0.3392
N	-1.2851	1.0153	-0.4010
C	-1.6775	1.0612	-1.8323
C	-2.9972	1.8309	-1.9341

C	-3.6364	1.5352	-0.5849
H	-2.6153	1.0593	1.2690
H	-2.2093	2.6427	0.5704
C	-0.5374	1.6753	-2.6546
H	-1.8623	0.0414	-2.1779
H	-2.8350	2.9037	-2.0251
H	-3.6162	1.4787	-2.7612
H	-4.0561	0.5275	-0.5830
H	-4.4033	2.2641	-0.3174
H	-0.8967	2.0716	-3.6057
H	0.1652	0.8672	-2.8670
N	0.1693	2.6894	-1.8431
C	-0.3958	4.0550	-1.8818
C	0.7646	5.0551	-1.7781
C	2.0093	4.1896	-1.5689
C	1.5851	2.8694	-2.2016
H	-1.0858	4.1957	-1.0490
H	-0.9221	4.2381	-2.8200
H	0.8566	5.6043	-2.7171
H	0.6270	5.7575	-0.9542
H	2.8959	4.6143	-2.0428
H	2.1844	4.0466	-0.5011
H	2.2009	2.0424	-1.8446
H	1.6806	2.9557	-3.2858
H	0.0943	2.3422	-0.8900
H	-1.2352	0.0373	-0.1209

Molecule 20 MM2

MM2 energy = 244.586 kJ/mol

C	4.4545	-5.3481	9.8579

N	3.1674	-5.9909	10.0224
C	2.3329	-5.4841	8.9522
C	2.6856	-3.9898	8.9000
C	4.1830	-3.9643	9.2453
H	4.9834	-5.2885	10.8381
H	5.0766	-5.9753	9.1771
C	2.6388	-6.1950	7.6224
H	1.2563	-5.6227	9.2141
H	2.4544	-3.5218	7.9142
H	2.1100	-3.4444	9.6865
H	4.4173	-3.1391	9.9595
H	4.8026	-3.8139	8.3290
H	3.6391	-5.8783	7.2407
H	1.8907	-5.8903	6.8502
N	2.6287	-7.6747	7.8503
C	2.9831	-8.4287	6.6079
C	2.6853	-9.8624	7.0668
C	1.5520	-9.7280	8.0987
C	1.2873	-8.2203	8.2263
H	4.0479	-8.2860	6.2985
H	2.3179	-8.1512	5.7525
H	2.3998	-10.5146	6.2072
H	3.5919	-10.3045	7.5461
H	0.6302	-10.2713	7.7810
H	1.8746	-10.1485	9.0816
H	0.9663	-7.9362	9.2580
H	0.4971	-7.9006	7.5026
H	3.3209	-7.9219	8.6085
H	2.7732	-5.7077	10.9256

Molecule 20 MM3

MM3 energy = 183.766 kJ/mol

C	3.4965	2.0766	-1.6016
N	2.6392	2.9130	-0.7616
C	1.9571	1.9876	0.1457
C	1.6262	0.7714	-0.7451
C	2.7881	0.7095	-1.7599
H	3.6887	2.5627	-2.5809
H	4.4823	1.9454	-1.1074
C	2.8798	1.6228	1.3257
H	1.0267	2.4554	0.5360
H	1.5257	-0.1676	-0.1574
H	0.6645	0.9431	-1.2816
H	2.4044	0.5670	-2.7953
H	3.4853	-0.1273	-1.5284
H	3.7045	0.9655	0.9657
H	2.2975	1.0106	2.0528
N	3.4626	2.8207	2.0106
C	4.3240	2.4557	3.1728
C	4.5077	3.7869	3.9361
C	3.2620	4.6331	3.5693
C	2.4503	3.7525	2.5897
H	5.2975	2.0080	2.8710
H	3.8042	1.7227	3.8322
H	4.5814	3.6174	5.0338
H	5.4374	4.3028	3.6033
H	2.6617	4.8905	4.4708
H	3.5710	5.5840	3.0779
H	1.9252	4.3657	1.8238
H	1.6781	3.1852	3.1591

H	4.0368	3.3403	1.3343
H	1.9445	3.3441	-1.3778

Molecule 20 MMFF

MMFF energy = 121.986 kJ/mol

C	-0.5815	0.8568	0.7907
N	-1.5657	0.5063	-0.2451
C	-0.7618	0.0839	-1.4252
C	0.5475	-0.4627	-0.8467
C	0.4556	-0.2350	0.6534
H	-0.1453	1.8412	0.5843
H	-1.0371	0.8737	1.7844
C	-1.5611	-0.9530	-2.2490
H	-0.5347	0.9512	-2.0577
H	0.7315	-1.5164	-1.0747
H	1.3907	0.1104	-1.2514
H	1.4192	0.0459	1.0887
H	0.1142	-1.1508	1.1505
H	-0.9405	-1.6244	-2.8498
H	-2.2614	-0.4219	-2.9038
N	-2.4063	-1.7720	-1.2963
C	-3.7764	-2.1285	-1.7967
C	-4.1487	-3.3256	-0.9493
C	-2.8446	-4.0952	-0.8766
C	-1.7803	-3.0200	-0.7244
H	-3.6898	-2.3933	-2.8561
H	-4.4370	-1.2680	-1.6612
H	-4.4603	-3.0059	0.0522
H	-4.9586	-3.9131	-1.3904
H	-2.8261	-4.8148	-0.0530

H	-2.6828	-4.6499	-1.8089
H	-0.8669	-3.2668	-1.2719
H	-1.5539	-2.8129	0.3256
H	-2.5260	-1.1116	-0.4980
H	-2.0915	1.3581	-0.4723

Molecule 20 OPLS-2005

OPLS-2005 energy = 121.986 kJ/mol

C	0.1599	-0.5996	1.1323
N	-1.2267	-0.5566	0.6835
C	-1.2621	0.3809	-0.4424
C	0.1586	0.3925	-1.0325
C	0.9533	-0.5712	-0.1647
H	0.3838	0.2858	1.7320
H	0.3733	-1.4804	1.7412
C	-2.3758	-0.0685	-1.4072
H	-1.4828	1.3831	-0.0665
H	0.2089	0.1300	-2.0889
H	0.5774	1.3968	-0.9426
H	1.9849	-0.2477	-0.0140
H	0.9829	-1.5661	-0.6098
H	-2.3335	0.4623	-2.3600
H	-3.3355	0.1926	-0.9570
N	-2.3010	-1.5383	-1.5790
C	-1.4728	-2.0410	-2.7030
C	-2.2672	-3.1794	-3.3581
C	-3.3129	-3.5678	-2.3156
C	-3.5984	-2.2460	-1.6070
H	-0.5274	-2.4123	-2.3049
H	-1.2384	-1.2678	-3.4365

H	-2.7650	-2.8129	-4.2580
H	-1.6362	-4.0191	-3.6549
H	-4.2056	-4.0160	-2.7558
H	-2.8918	-4.2895	-1.6130
H	-4.0067	-2.3866	-0.6047
H	-4.3227	-1.6719	-2.1886
H	-1.8271	-1.7964	-0.7127
H	-1.7908	-0.2211	1.4631

Molecule 20 OPLS3e

OPLS3e energy = 346.337 kJ/mol

C	-1.1627	-0.9544	-1.4446
N	0.0115	-1.1183	-0.5963
C	1.0999	-1.5161	-1.4868
C	0.4224	-2.4292	-2.5172
C	-1.0693	-2.0962	-2.4496
H	-2.0742	-1.0264	-0.8446
H	-1.1848	0.0175	-1.9440
C	1.8213	-0.2865	-2.0975
H	1.8047	-2.0954	-0.8859
H	0.8162	-2.2687	-3.5225
H	0.6123	-3.4801	-2.2898
H	-1.6561	-2.9547	-2.1163
H	-1.4695	-1.8013	-3.4214
H	1.1703	0.2232	-2.8115
H	2.7616	-0.5357	-2.5941
N	2.1055	0.7521	-0.9838
C	2.4586	2.1732	-1.4738
C	3.2156	2.8132	-0.3013
C	3.5364	1.6947	0.7020

C	3.2400	0.3757	-0.0108
H	1.5405	2.6872	-1.7737
H	3.1009	2.0621	-2.3526
H	4.1298	3.2926	-0.6614
H	2.6227	3.6050	0.1645
H	4.5745	1.7380	1.0423
H	2.9164	1.7839	1.5980
H	2.9052	-0.4406	0.6363
H	4.0815	0.0165	-0.6101
H	1.2333	0.7582	-0.4535
H	-0.1828	-1.8877	0.0413

Molecule 20 OPLS

OPLS energy = 8.465 kJ/mol

C	-5.6127	-2.8397	0.8425
N	-5.8900	-3.2820	2.2249
C	-6.0892	-4.7423	2.0347
C	-6.5338	-5.0173	0.5909
C	-6.5379	-3.6383	-0.0656
H	-5.7779	-1.7681	0.7326
H	-4.5782	-3.0758	0.5901
C	-4.7796	-5.4235	2.4231
H	-6.8594	-5.0711	2.7327
H	-5.8336	-5.6743	0.0753
H	-7.5336	-5.4509	0.5653
H	-7.5418	-3.2157	-0.0168
H	-6.1824	-3.6687	-1.0957
H	-4.1247	-5.4966	1.5543
H	-4.9426	-6.4129	2.8507
N	-4.1874	-4.4723	3.3687

C	-2.7335	-4.2936	3.2808
C	-2.2896	-3.6252	4.5840
C	-3.4648	-3.8248	5.5498
C	-4.4145	-4.7483	4.7923
H	-2.4581	-3.6957	2.4117
H	-2.2582	-5.2735	3.2201
H	-2.1229	-2.5594	4.4262
H	-1.3833	-4.0981	4.9628
H	-3.9541	-2.8655	5.7201
H	-3.1476	-4.2635	6.4959
H	-5.4520	-4.5765	5.0795
H	-4.1356	-5.7797	5.0116
H	-4.6974	-3.6201	3.0903
H	-6.8364	-2.9146	2.3198

Coordinates of Lowest Energy Conformers (DFT)

Created using ESIgen v0.0.5

ESIgen is scientific software, funded by public research grants and published as:

```
J Rodriguez-Guerra, P Gomez-Orellana, JD Marechal.
J. Chem. Inf. Model., 2018, 58 (3), pp 561564.
DOI: 10.1021/acs.jcim.7b00714.
```

If you make use of ESIgen in scientific publications, please cite us in the main text! References only mentioned in SI documents are not indexed by citation engines.

Molecule 1

Datum	Value
M06-2X/6-31G(d)-IEFPCM(Benzene) Energy	-2180.964623
M06-2X/def2-TZVPP-IEFPCM(Benzene)//M06-2X/6-31G(d)-IEFPCM(Benzene) Free Energy (Quasiharmonic)	-2181.228809
M06-2x/def2-TZVPP-IEFPCM(Benzene) Energy	-2181.719512
Number of Imaginary Frequencies	0

Frequencies (Top 3 out of 204)

```
 1.      21.4998 cm-1
 2.      23.6435 cm-1
 3.      32.1959 cm-1
```

M06-2X/6-31G(d)-IEFPCM(Benzene) Molecular Geometry in Cartesian Coordinates

```
C         -2.545846       1.570976      -1.296698
C         -1.207270       1.597914      -0.958940
C         -0.690671       0.524544      -0.183795
C         -1.552920      -0.498801       0.188530
H         -2.945898       2.363504      -1.923416
O         -0.992089      -1.540208       0.924014
C          1.503941      -0.647287      -0.092323
C          0.738000       0.462118       0.251720
```

```
C          1.353905        1.510901        0.985964
C          2.691543        1.385139        1.301947
O          0.841742       -1.670377       -0.773882
H          3.165867        2.158258        1.900363
C         -2.931670       -0.548759       -0.125098
C         -3.410332        0.531338       -0.909976
C         -4.772186        0.547474       -1.365228
C         -5.610494       -0.469751       -1.071357
H         -5.099656        1.388130       -1.969937
H         -6.634608       -0.470522       -1.434177
C          3.465584        0.276781        0.916388
C          2.887744       -0.785268        0.172926
C          5.606460       -0.852436        1.004246
C          4.841348        0.213524        1.322442
H          6.642983       -0.910287        1.324894
H          5.240876        1.038739        1.904724
C         -5.180391       -1.570529       -0.254626
C         -3.855430       -1.616704        0.260869
C         -3.540498       -2.691305        1.131843
C         -4.462252       -3.677672        1.422341
C         -5.751453       -3.645905        0.869398
C         -6.100488       -2.595295        0.052321
H         -2.580427       -2.733362        1.623045
H         -4.182465       -4.478798        2.099365
H         -6.467603       -4.428279        1.098999
H         -7.101069       -2.530930       -0.366925
C          3.732474       -1.908662       -0.238877
C          5.081588       -1.929308        0.212084
C          5.934219       -2.996841       -0.138437
C          5.494209       -4.024903       -0.939950
C          4.178411       -3.993666       -1.424942
C          3.320748       -2.966500       -1.086893
H          6.955408       -2.983372        0.233102
H          6.158460       -4.841380       -1.204531
H          3.823495       -4.784125       -2.078656
H          2.328717       -2.972363       -1.508618
C          1.138399        4.910771       -2.340311
C         -0.109452        5.119912       -1.758455
C         -0.860203        4.037602       -1.312128
C         -0.375083        2.731480       -1.439366
C          0.874645        2.529173       -2.035823
C          1.626046        3.612382       -2.480479
H          1.728639        5.754242       -2.685229
H         -0.492980        6.128281       -1.635533
H         -1.812803        4.203291       -0.816009
H          1.255302        1.519426       -2.158592
H          2.592683        3.440433       -2.943902
C         -0.697135        5.041183        2.295809
C         -1.288344        3.791549        2.474799
C         -0.632223        2.638885        2.054651
C          0.623871        2.721599        1.443238
C          1.213453        3.979584        1.277484
C          0.558407        5.131314        1.699958
H         -1.212847        5.939327        2.621693
```

```
H          -2.261057        3.712127        2.950452
H          -1.091927        1.667056        2.208685
H           2.171365        4.053417        0.769551
H           1.022447        6.101060        1.547315
P          -0.117124       -2.615746        0.103204
O          -1.033232       -3.145071       -1.087389
O           0.472036       -3.620786        0.991676
H          -1.497626       -3.965461       -0.851603
```

Molecule 2

Datum	Value
M06-2X/6-31G(d)-IEFPCM(Benzene) Energy	-1303.023633
M06-2X/def2-TZVPP-IEFPCM(Benzene)//M06-2X/6-31G(d)-IEFPCM(Benzene) Free Energy (Quasiharmonic)	-1303.093491
M06-2x/def2-TZVPP-IEFPCM(Benzene) Energy	-1303.518854
Number of Imaginary Frequencies	0

Frequencies (Top 3 out of 162)

```
1.      19.2312 cm-1
2.      32.1134 cm-1
3.      44.5627 cm-1
```

M06-2X/6-31G(d)-IEFPCM(Benzene) Molecular Geometry in Cartesian Coordinates

```
C          -0.298241       -0.878954        0.414654
C          -0.407688        0.481131        0.721855
C          -1.615876        1.138750        0.527431
C          -2.691104        0.386972        0.047101
C          -2.545250       -0.981491       -0.261117
C          -1.335612       -1.646018       -0.088335
O           0.979385       -1.254008        0.691695
N           0.831643        0.933468        1.176602
H          -1.736370        2.196575        0.733298
H          -3.390014       -1.524947       -0.670935
H          -1.204776       -2.690588       -0.343820
C           1.592809       -0.106701        1.110743
C           3.044444       -0.209991        1.494808
C           3.747367        1.136097        1.239438
C           4.126333        1.440921       -0.223946
C           4.941840        0.274463       -0.786191
```

```
C          4.129619      -1.024280      -0.734216
C          3.768346      -1.339451       0.734421
H          4.682019       1.120331       1.817062
H          3.131873       1.947806       1.643670
C          4.945899       2.733269      -0.241463
C          2.909874       1.644565      -1.141883
H          5.237019       0.490634      -1.822557
H          5.866869       0.147374      -0.206321
C          4.921625      -2.191869      -1.318528
H          4.712164      -1.530219       1.263189
H          3.181585      -2.263183       0.777388
C          3.063804      -0.522786       3.004398
H          5.815876       2.646102       0.417388
H          5.305070       2.955036      -1.251887
H          4.344558       3.582579       0.102207
H          5.194853      -1.992839      -2.359439
H          5.840742      -2.344550      -0.743146
H          4.327836      -3.107875      -1.295222
C          2.830269      -0.864596      -1.528135
O          2.257120      -1.816493      -2.039060
H          3.232766       2.207706      -2.028142
N          2.304337       0.390261      -1.576871
H          2.148210       2.237583      -0.623477
H          1.438359       0.448228      -2.101446
H          2.566154       0.273510       3.565692
H          2.551072      -1.467616       3.211312
H          4.097596      -0.606350       3.353791
C         -3.973048       1.119919      -0.207289
O         -3.952883       2.311060      -0.464456
C         -7.796518      -0.808651      -0.007986
C         -7.589685       0.281995      -0.852914
C         -6.341115       0.887944      -0.909170
C         -5.281967       0.392881      -0.141359
C         -5.495773      -0.696290       0.709074
C         -6.753113      -1.290352       0.778761
H         -8.773775      -1.279005       0.040961
H         -8.404602       0.661026      -1.461429
H         -6.164374       1.749654      -1.544873
H         -4.687158      -1.064699       1.333002
H         -6.917819      -2.127721       1.449262
```

Molecule 3

Datum	Value
M06-2X/6-31G(d)-IEFPCM(Benzene) Energy	-1498.475207
M06-2X/def2-TZVPP-IEFPCM(Benzene)//M06-2X/6-31G(d)-IEFPCM(Benzene) Free Energy (Quasiharmonic)	-1498.773075
M06-2x/def2-TZVPP-IEFPCM(Benzene) Energy	-1499.107093

Datum	Value
Number of Imaginary Frequencies	0

Frequencies (Top 3 out of 138)

```
1.       9.6107 cm-1
2.      11.9448 cm-1
3.      20.1168 cm-1
```

M06-2X/6-31G(d)-IEFPCM(Benzene) Molecular Geometry in Cartesian Coordinates

```
C         -1.754693        0.972496        0.104739
C         -3.127134        1.170507        0.231478
C         -4.042249        0.136679        0.098147
C         -3.547070       -1.135954       -0.172279
C         -2.187109       -1.365916       -0.303041
C         -1.270906       -0.312227       -0.167466
H         -1.064662        1.797985        0.213660
C         -3.598318        2.571149        0.510754
H         -5.105728        0.310536        0.208425
C         -4.507751       -2.272178       -0.382174
H         -1.830620       -2.372357       -0.503200
N          0.082446       -0.621464       -0.298236
F         -5.620403       -2.121157        0.348668
F         -4.891509       -2.362317       -1.665289
F         -3.963351       -3.453787       -0.054731
F         -3.325970        3.392541       -0.514358
F         -4.919443        2.626249        0.727301
F         -2.991181        3.086817        1.589872
H          0.289341       -1.578955       -0.549351
C          1.159814        0.258170       -0.261069
O          1.038852        1.465024       -0.116118
N          2.363829       -0.370214       -0.432066
H          2.454975       -1.330440       -0.104257
C          5.199086        2.089624       -1.150669
C          3.887529        1.339708       -1.395299
C          3.591902        0.387496       -0.233758
C          4.743401       -0.618841       -0.080499
C          6.046533        0.138272        0.211293
C          6.360937        1.119276       -0.923397
H          5.086604        2.731531       -0.266423
H          5.414893        2.751973       -1.995223
H          3.955017        0.749927       -2.319412
H          3.052891        2.036424       -1.505924
H          3.504538        0.993720        0.681222
N          4.382311       -1.689795        0.856515
H          4.853513       -1.114965       -1.057137
```

```
H          5.945329       0.693615       1.153332
H          6.871675      -0.571097       0.341928
H          6.543954       0.552329      -1.846442
H          7.281577       1.668842      -0.701046
C          5.249162      -2.852064       0.740340
C          4.281754      -1.260058       2.244920
H          6.276875      -2.667265       1.095158
H          4.833647      -3.671524       1.334777
H          5.296817      -3.173941      -0.303970
H          3.875063      -2.082551       2.840719
H          5.252928      -0.974157       2.680840
H          3.597832      -0.411469       2.332493
```

Molecule 4

Datum	Value
M06-2X/6-31G(d)-IEFPCM(Benzene) Energy	-1797.873822
M06-2X/def2-TZVPP-IEFPCM(Benzene)//M06-2X/6-31G(d)-IEFPCM(Benzene) Free Energy (Quasiharmonic)	-1798.054859
M06-2x/def2-TZVPP-IEFPCM(Benzene) Energy	-1798.274101
Number of Imaginary Frequencies	0

Frequencies (Top 3 out of 99)

```
1.      21.6076 cm-1
2.      31.0106 cm-1
3.      38.0793 cm-1
```

M06-2X/6-31G(d)-IEFPCM(Benzene) Molecular Geometry in Cartesian Coordinates

```
C          1.998878       0.606733       0.152625
C          2.606972       0.021759      -0.962203
C          3.027353      -1.304383      -0.933755
C          2.835329      -2.049689       0.224870
C          2.241140      -1.492327       1.351624
C          1.827099      -0.164788       1.308201
H          2.738550       0.609239      -1.865996
H          3.493149      -1.759447      -1.801007
Cl         3.350947      -3.715810       0.265582
H          2.107351      -2.088840       2.247277
H          1.366474       0.281797       2.185724
C          0.662701       4.669540      -0.033080
```

```
C          2.023658      4.386081     -0.065093
C          2.442338      3.060445     -0.001269
C          1.522465      2.018057      0.095806
C          0.143777      2.318161      0.110058
C         -0.280588      3.649363      0.052410
H          0.319390      5.698338     -0.078124
H          2.753254      5.185851     -0.135847
H          3.500793      2.816304     -0.017288
N         -0.748752      1.227613      0.164606
H         -1.337254      3.872252      0.062433
H         -0.305802      0.321450      0.245905
C         -2.095187      1.230554     -0.026263
O         -2.773883      2.235861     -0.163985
C         -4.247329     -2.437934      0.180820
C         -4.844749     -1.250859      0.586411
C         -4.098342     -0.086219      0.492805
C         -2.783128     -0.118799      0.021435
C         -2.309200     -1.379705     -0.373244
N         -3.004349     -2.496799     -0.301161
H         -4.779956     -3.383399      0.231762
H         -5.862289     -1.241018      0.958760
H         -4.509789      0.877124      0.774498
Cl        -0.707430     -1.592906     -1.067539
```

Molecule 5

Datum	Value
M06-2X/6-31G(d)-IEFPCM(Benzene) Energy	-883.94268
M06-2X/def2-TZVPP-IEFPCM(Benzene)//M06-2X/6-31G(d)-IEFPCM(Benzene) Free Energy (Quasiharmonic)	-883.922771
M06-2x/def2-TZVPP-IEFPCM(Benzene) Energy	-884.277462
Number of Imaginary Frequencies	0

Frequencies (Top 3 out of 126)

```
1.      48.1785 cm-1
2.      50.0198 cm-1
3.      68.8991 cm-1
```

M06-2X/6-31G(d)-IEFPCM(Benzene) Molecular Geometry in Cartesian Coordinates

```
O          0.610460      -1.844676       2.041293
C          0.112077      -0.635385       1.497240
C          1.364424      -0.006929       0.846761
H         -0.164201       0.052195       2.307082
C         -1.112084      -0.860763       0.624490
H          2.018737       0.230317       1.689718
C          1.212021       1.212266      -0.069333
N          2.154199      -1.050300       0.078430
C         -2.054277       0.185625       0.378815
C         -1.375455      -2.106618       0.109860
C          2.337324       1.169173      -1.121401
H          1.250949       2.122434       0.534701
H          0.244552       1.201397      -0.580900
C          1.662908      -1.221048      -1.338728
C          3.602815      -0.630835       0.056024
C         -1.941467       1.504532       0.898508
C         -3.187873      -0.131257      -0.425558
C         -2.535005      -2.308879      -0.679459
H         -0.708138      -2.940837       0.302027
C          1.998383       0.058572      -2.125597
H          2.407542       2.132788      -1.631684
C          3.694027       0.822976      -0.476582
H          0.591885      -1.408329      -1.283465
H          2.155901      -2.109132      -1.737365
H          3.984767      -0.729472       1.074888
H          4.120547      -1.347686      -0.584163
C         -2.895724       2.450891       0.623023
H         -1.095070       1.775164       1.521792
C         -4.154073       0.871537      -0.699336
N         -3.406533      -1.371052      -0.950470
H         -2.732625      -3.295461      -1.093209
H          1.140548       0.337988      -2.741843
H          2.845854      -0.114641      -2.794661
H          4.441669       0.828744      -1.276438
C          4.143733       1.822024       0.589149
C         -4.011946       2.134661      -0.188073
H         -5.000334       0.591812      -1.317577
H          5.140245       1.566536       0.959853
H          4.191793       2.829120       0.165643
H          3.468735       1.853865       1.450564
H         -4.755944       2.896176      -0.396779
H         -0.076657      -2.265697       2.580539
H          2.050063      -1.934478       0.593778
H         -2.797010       3.450804       1.032625
```

Molecule 6

Datum	Value
M06-2X/6-31G(d)-IEFPCM(Benzene) Energy	-1492.681468

Datum	Value
M06-2X/def2-TZVPP-IEFPCM(Benzene)//M06-2X/6-31G(d)-IEFPCM(Benzene) Free Energy (Quasiharmonic)	-1492.813681
M06-2x/def2-TZVPP-IEFPCM(Benzene) Energy	-1493.256712
Number of Imaginary Frequencies	0

Frequencies (Top 3 out of 174)

```
1.      18.4210 cm-1
2.      25.1140 cm-1
3.      35.5808 cm-1
```

M06-2X/6-31G(d)-IEFPCM(Benzene) Molecular Geometry in Cartesian Coordinates

```
C         -2.219125       -1.931945       -1.344873
C         -3.461562       -1.541371       -0.924411
C         -3.665345       -0.277074       -0.296578
C         -2.581983        0.560612       -0.130054
H         -2.082067       -2.897021       -1.825015
H         -4.315631       -2.196160       -1.059258
H         -2.709632        1.522622        0.362717
C         -1.281990        0.180426       -0.555227
C         -1.090354       -1.087352       -1.164515
C          0.212899       -1.473952       -1.571699
C          1.288690       -0.650414       -1.347799
C          1.114064        0.624585       -0.726181
C         -0.154656        1.019246       -0.369693
H          0.327874       -2.423406       -2.083865
O          2.577487       -0.944758       -1.711990
H         -0.294002        1.992442        0.095588
C          4.335815        3.450211       -0.263929
C          3.239647        3.727117       -1.032100
C          2.213720        2.765685       -1.152239
C          2.264472        1.546869       -0.508744
H          5.149143        4.163895       -0.171884
H          3.157903        4.668757       -1.564078
H          1.362224        2.974701       -1.792674
C          3.386056        1.250690        0.337775
C          4.435519        2.217049        0.431948
C          5.585942        1.957673        1.221552
C          5.728236        0.785878        1.915170
C          4.697751       -0.171503        1.855668
C          3.560884        0.053791        1.106357
H          6.360710        2.717761        1.262232
H          6.610071        0.593793        2.515464
```

```
H          4.787717      -1.094176       2.423231
C          2.524441      -1.003528       1.252369
N          1.459115      -0.736582       1.985829
H          0.666207      -1.365386       2.051915
N          2.727575      -2.216218       0.767148
H          1.347760       0.190281       2.375906
H          2.023117      -2.924683       0.962965
C          3.688076      -2.551359      -0.299427
C          4.137905      -3.996659      -0.156175
C          3.028885      -2.285111      -1.656054
H          4.534971      -1.873128      -0.184772
H          3.292085      -4.685336      -0.255628
H          4.858337      -4.235254      -0.942091
H          4.615364      -4.162442       0.811854
H          2.222166      -3.003736      -1.828288
H          3.770989      -2.407164      -2.448082
C         -7.562666       0.906811       1.047145
C         -6.846508       1.747405       0.208751
C         -5.582088       1.356066      -0.233990
C         -5.020305       0.128864       0.155959
C         -5.780522      -0.703155       0.994646
C         -7.041339      -0.321744       1.444307
H         -8.545769       1.206026       1.396015
H         -7.242492       2.700079      -0.123049
O         -4.932468       2.215680      -1.062087
O         -5.213776      -1.887061       1.363040
H         -7.603817      -0.978709       2.101862
H         -4.142928       1.780094      -1.421610
H         -5.834422      -2.368012       1.930263
```

Molecule 7

Datum	Value
M06-2X/6-31G(d)-IEFPCM(Benzene) Energy	-727.928662
M06-2X/def2-TZVPP-IEFPCM(Benzene)//M06-2X/6-31G(d)-IEFPCM(Benzene) Free Energy (Quasiharmonic)	-728.037398
M06-2x/def2-TZVPP-IEFPCM(Benzene) Energy	-728.221096
Number of Imaginary Frequencies	0

Frequencies (Top 3 out of 78)

```
1.      17.5103 cm-1
2.      20.5245 cm-1
3.      27.9426 cm-1
```

M06-2X/6-31G(d)-IEFPCM(Benzene) Molecular Geometry in Cartesian Coordinates

```
C	-5.822875	-0.064207	-0.419431
C	-5.090563	1.111173	-0.580212
C	-3.720174	1.114902	-0.352117
C	-3.073367	-0.065881	0.040524
C	-3.813848	-1.246217	0.200799
C	-5.183862	-1.240414	-0.029581
H	-6.893400	-0.063661	-0.598635
H	-5.588804	2.025977	-0.884234
H	-3.139789	2.023396	-0.474794
C	-1.661784	-0.068700	0.278017
H	-3.304621	-2.154987	0.503939
H	-5.754729	-2.154829	0.094635
C	-0.467791	-0.089572	0.482882
C	0.966614	-0.074447	0.724843
O	1.568865	-1.069707	1.072223
C	1.649773	1.264973	0.529656
H	1.435943	1.608143	-0.488285
H	1.189087	1.987164	1.213447
C	3.145886	1.155937	0.764085
H	3.617490	2.143380	0.713974
H	3.367213	0.736823	1.748241
C	3.789990	0.280687	-0.287361
O	3.291003	-0.003640	-1.350078
O	5.010642	-0.120723	0.088200
C	5.696539	-0.940054	-0.860549
H	6.656204	-1.179832	-0.406333
H	5.839025	-0.398661	-1.798141
H	5.125082	-1.849443	-1.055910
```

Molecule 8

Datum	Value
M06-2X/6-31G(d)-IEFPCM(Benzene) Energy	-950.351209
M06-2X/def2-TZVPP-IEFPCM(Benzene)//M06-2X/6-31G(d)-IEFPCM(Benzene) Free Energy (Quasiharmonic)	-950.463779
M06-2x/def2-TZVPP-IEFPCM(Benzene) Energy	-950.728979
Number of Imaginary Frequencies	0

Frequencies (Top 3 out of 105)

```
1.      33.0433 cm-1
2.      43.3943 cm-1
3.      57.2034 cm-1
```

M06-2X/6-31G(d)-IEFPCM(Benzene) Molecular Geometry in Cartesian Coordinates

```
N         -1.245673       -1.686462        1.312226
C          0.446421       -1.330380       -0.437737
N         -0.672536       -1.555021       -1.337181
C         -2.380778       -1.519796        0.422260
H          1.188927       -2.118676       -0.612086
C          1.119159        0.044881       -0.689733
H         -3.072332       -2.352689        0.583017
C         -3.135873       -0.197289        0.683294
O          0.907114       -1.402418        1.905459
C          0.052407       -1.464980        1.026664
O         -2.862483       -1.631975       -1.899671
C         -1.991199       -1.581659       -1.045637
H         -1.440528       -1.756315        2.306002
H         -0.466086       -1.571775       -2.330408
H         -3.495079       -0.205297        1.718304
H         -4.010785       -0.195748        0.026716
C         -0.648505        3.249855       -0.050187
C         -0.741037        2.716835        1.233848
C         -1.548330        1.605637        1.470882
C         -2.276249        1.016874        0.432985
C         -2.168771        1.554525       -0.854710
C         -1.362128        2.664108       -1.094337
H         -0.022183        4.116571       -0.236218
H         -0.186261        3.165474        2.051920
H         -1.622250        1.196349        2.475822
H         -2.727710        1.098120       -1.668457
H         -1.294924        3.074950       -2.097055
H          0.620217        0.802950       -0.074355
H          0.933540        0.323405       -1.731759
C          2.598291        0.052857       -0.477493
C          3.630692        0.462016       -1.284587
N          4.840905        0.295527       -0.657078
C          4.543726       -0.214461        0.517040
N          3.206154       -0.376529        0.677426
H          3.561141        0.872033       -2.283504
H          5.253640       -0.487523        1.285460
H          2.694713       -0.783418        1.457219
```

Molecule 9

Datum	Value
M06-2X/6-31G(d)-IEFPCM(Benzene) Energy	-1148.035621
M06-2X/def2-TZVPP-IEFPCM(Benzene)//M06-2X/6-31G(d)-IEFPCM(Benzene) Free Energy (Quasiharmonic)	-1148.048613
M06-2x/def2-TZVPP-IEFPCM(Benzene) Energy	-1148.46153
Number of Imaginary Frequencies	0

Frequencies (Top 3 out of 153)

```
1.      37.7852 cm-1
2.      44.9524 cm-1
3.      48.8673 cm-1
```

M06-2X/6-31G(d)-IEFPCM(Benzene) Molecular Geometry in Cartesian Coordinates

```
C          2.262781        0.062138        0.762956
C          2.244333       -0.300829       -0.748146
C          3.563982        0.058821       -1.435655
C          4.793577       -0.487842       -0.709342
C          4.818764        0.019762        0.731529
C          3.542136       -0.401838        1.460861
H          1.413289       -0.425654        1.250381
N          2.087365        1.507819        0.922930
H          1.449631        0.269387       -1.246439
N          1.950733       -1.713983       -0.892300
H          3.628860        1.153072       -1.471668
H          3.525241       -0.283624       -2.475732
H          5.699038       -0.184043       -1.242396
H          4.781215       -1.585873       -0.706012
H          5.688914       -0.370526        1.266819
H          4.922821        1.114397        0.726433
H          3.532163       -0.048908        2.498114
H          3.499675       -1.495776        1.500201
H          2.841528        2.047888        1.328075
H          2.556347       -2.254904       -1.494839
N         -0.109260        1.505995        0.171024
C          0.960040        2.166295        0.646056
C          0.869301        3.583596        0.840663
C         -0.274363        4.234083        0.512147
H         -0.350700        5.307067        0.663038
N         -0.202081       -1.464853       -0.065461
C          0.708601       -2.231495       -0.644163
C          0.463974       -3.600304       -1.000056
C         -0.746348       -4.144590       -0.714877
H         -0.968261       -5.173546       -0.984190
```

```
C          -1.732187       -3.374251       -0.030004
C          -1.400479       -2.036082        0.297344
C          -2.327521       -1.273723        1.045876
C          -3.540240       -1.812346        1.413164
C          -3.882148       -3.133142        1.055723
C          -2.985943       -3.901673        0.350009
H          -2.058630       -0.265013        1.346656
H          -4.237630       -1.215936        1.992691
H          -4.842613       -3.542015        1.349858
H          -3.223097       -4.928251        0.084526
C          -1.386477        3.538498       -0.060246
C          -1.269644        2.147869       -0.245161
C          -2.295004        1.417322       -0.862583
C          -3.441010        2.079409       -1.257792
C          -3.587262        3.463885       -1.056263
C          -2.569352        4.184814       -0.470317
H          -2.175228        0.352361       -1.032318
H          -4.236809        1.519411       -1.737211
H          -4.496611        3.961870       -1.373127
H          -2.657417        5.257004       -0.323475
H           1.244222       -4.171423       -1.492419
H           1.729332        4.102550        1.248761
H          -0.089646        0.465381        0.080578
```

Molecule 10

Datum	Value
M06-2X/6-31G(d)-IEFPCM(Benzene) Energy	-2423.147963
M06-2X/def2-TZVPP-IEFPCM(Benzene)//M06-2X/6-31G(d)-IEFPCM(Benzene) Free Energy (Quasiharmonic)	-2423.746317
M06-2x/def2-TZVPP-IEFPCM(Benzene) Energy	-2424.002433
Number of Imaginary Frequencies	0

Frequencies (Top 3 out of 126)

```
1.      16.9762 cm-1
2.      22.9091 cm-1
3.      31.4360 cm-1
```

M06-2X/6-31G(d)-IEFPCM(Benzene) Molecular Geometry in Cartesian Coordinates

```
C          1.746047      -1.854846      -2.047011
C          2.883559      -2.417426      -2.618457
C          3.892125      -2.931127      -1.805777
C          3.758287      -2.882318      -0.420489
C          2.620256      -2.319122       0.150214
C          1.608873      -1.803342      -0.659355
H          0.951725      -1.473148      -2.683766
H          2.979224      -2.460180      -3.698805
H          4.777144      -3.373885      -2.251783
H          4.537251      -3.287284       0.217649
H          2.516849      -2.277232       1.231667
C          0.405983      -1.126198      -0.032064
N         -0.837283      -1.431695      -0.747694
H          0.298549      -1.475329       0.999564
C          0.622662       0.407640      -0.014452
H          0.804833       0.718109      -1.048356
N         -0.591871       1.102281       0.458292
C          4.013560       1.464290       2.416192
C          2.886946       0.889013       3.001462
C          1.791734       0.549583       2.213680
C          1.812112       0.790865       0.836742
C          2.940122       1.367828       0.257001
C          4.039405       1.701050       1.044553
H          4.870141       1.724860       3.029432
H          2.862955       0.699115       4.069611
H          0.922600       0.084421       2.677288
H          2.961127       1.547279      -0.814396
H          4.915910       2.145860       0.584646
H         -1.213853      -0.748974      -1.408462
H         -0.617480       1.418598       1.424868
S         -1.943349      -2.437966      -0.114348
O         -1.257606      -3.454078       0.655759
C         -2.824198      -1.409604       1.136589
O         -2.930860      -2.733947      -1.127924
S         -1.512671       1.985441      -0.552973
O         -2.676204       2.452985       0.162688
C         -0.506646       3.489500      -0.912613
O         -1.580794       1.267526      -1.815793
F         -3.776827      -2.120397       1.715844
F         -1.957367      -1.000838       2.065256
F         -3.357684      -0.348298       0.545988
F         -1.158481       4.272052      -1.752257
F         -0.275354       4.135121       0.220540
F          0.653731       3.123413      -1.451075
```

Molecule 11

Datum	Value
M06-2X/6-31G(d)-IEFPCM(Benzene) Energy	-1975.589694

Datum	Value
M06-2X/def2-TZVPP-IEFPCM(Benzene)//M06-2X/6-31G(d)-IEFPCM(Benzene) Free Energy (Quasiharmonic)	-1975.675621
M06-2x/def2-TZVPP-IEFPCM(Benzene) Energy	-1976.31528
Number of Imaginary Frequencies	0

Frequencies (Top 3 out of 249)

```
1.      12.4506 cm-1
2.      14.7270 cm-1
3.      19.0868 cm-1
```

M06-2X/6-31G(d)-IEFPCM(Benzene) Molecular Geometry in Cartesian Coordinates

```
C         -0.672869        2.521706        0.255846
C         -1.487598        1.437261       -0.036459
C         -2.835450        1.398082        0.440193
C         -3.328383        2.469625        1.148243
C         -0.906591        0.300943       -0.861483
H         -4.357413        2.449353        1.499624
C         -2.511907        3.570231        1.500798
C         -1.152291        3.588092        1.085295
C         -0.322381        4.649938        1.542446
C         -0.831211        5.653069        2.327123
C         -2.196684        5.653659        2.702759
C         -3.014584        4.630588        2.302706
H          0.727679        4.656619        1.271246
H         -0.180569        6.451541        2.669684
H         -2.585989        6.457695        3.319301
H         -4.058922        4.603619        2.602099
C          3.383979        2.443870       -1.157298
C          0.730282        2.520150       -0.258399
C          1.533729        1.425763        0.020330
C          2.882007        1.371780       -0.455918
H          4.409947        2.414595       -1.516786
C          0.933687        0.275640        0.802602
C          2.580138        3.558680       -1.495008
C          1.222195        3.591225       -1.074820
C          0.405365        4.668676       -1.517776
C          0.925201        5.672828       -2.294176
C          2.289006        5.658301       -2.674970
C          3.094212        4.619999       -2.288306
H         -0.643311        4.687033       -1.241865
H          0.284547        6.483928       -2.625689
H          2.687279        6.463217       -3.284592
```

```
H        4.137112    4.582240   -2.591591
H        1.717859   -0.390238    1.162231
H        0.389024    0.661490    1.675701
N        0.054564   -0.490634   -0.076031
H       -0.401436    0.720873   -1.738720
H       -1.692742   -0.368061   -1.207465
N        0.197895   -2.489220    1.234314
C       -0.142962   -1.840658    0.180107
N       -0.781237   -2.516407   -0.856409
H        0.491193   -1.855711    1.976292
H       -0.442939   -2.286764   -1.784103
H       -0.788260   -3.514227   -0.681986
C       -5.473565   -1.957434   -0.169561
C       -4.224000   -2.128152    0.437813
C       -3.366498   -1.051619    0.623755
C       -3.729187    0.232279    0.203299
C       -4.980606    0.407523   -0.396966
C       -5.838464   -0.670082   -0.581584
H       -3.927663   -3.110700    0.793811
H       -2.413607   -1.208629    1.123240
H       -5.271156    1.396734   -0.740358
H       -6.791919   -0.517370   -1.079376
C        5.486570   -2.017205    0.137118
C        5.902265   -0.720439    0.459645
C        5.054998    0.366614    0.280148
C        3.762868    0.194417   -0.227524
C        3.348669   -1.102301   -0.558292
C        4.194915   -2.188036   -0.376266
H        6.889050   -0.565656    0.886742
H        5.387195    1.362573    0.560179
H        2.357329   -1.253935   -0.978347
H        3.857660   -3.180888   -0.659433
C       -8.109025   -5.293145   -0.758518
C       -8.624061   -4.046112   -0.411746
C       -7.769723   -2.964929   -0.220199
C       -6.385474   -3.110027   -0.372835
C       -5.879933   -4.368240   -0.721913
C       -6.733377   -5.450243   -0.912443
H       -8.775258   -6.137123   -0.907415
H       -9.694072   -3.916072   -0.281223
H       -8.175746   -2.001561    0.075440
H       -4.810293   -4.490845   -0.867450
H       -6.323362   -6.416647   -1.189517
C        8.090527   -5.378532    0.713512
C        6.726521   -5.496030    0.971218
C        5.882451   -4.405960    0.784507
C        6.387112   -3.179187    0.336083
C        7.759573   -3.073303    0.080094
C        8.604148   -4.162820    0.267420
H        8.749176   -6.228901    0.859777
H        6.318630   -6.437044    1.327406
H        4.824023   -4.496631    1.012200
H        8.162240   -2.135362   -0.292169
H        9.664597   -4.064228    0.056386
```

Molecule 12

Datum	Value
M06-2X/6-31G(d)-IEFPCM(Benzene) Energy	-1875.782391
M06-2X/def2-TZVPP-IEFPCM(Benzene)//M06-2X/6-31G(d)-IEFPCM(Benzene) Free Energy (Quasiharmonic)	-1876.177197
M06-2x/def2-TZVPP-IEFPCM(Benzene) Energy	-1876.435464
Number of Imaginary Frequencies	0

Frequencies (Top 3 out of 120)

```
1.      14.2333 cm-1
2.      18.6158 cm-1
3.      22.3755 cm-1
```

M06-2X/6-31G(d)-IEFPCM(Benzene) Molecular Geometry in Cartesian Coordinates

```
C         -2.956761        1.005409       -0.002001
C         -3.109731        0.217358       -1.345636
C         -2.936622       -1.262360       -0.967983
N         -1.570809        1.396289        0.221504
H         -3.557879        1.916680        0.008798
H         -4.137494        0.381175       -1.697950
O         -2.174817        0.568383       -2.335609
H         -3.516367       -1.926353       -1.614347
H         -1.881387       -1.549193       -1.071263
C         -3.382007       -0.015116        1.025438
C         -3.368678       -1.300124        0.477287
C         -3.714936       -2.394933        1.262779
C         -4.064785       -2.185338        2.596099
C         -4.069411       -0.899506        3.141323
C         -3.725341        0.198992        2.355856
H         -3.714071       -3.397715        0.845165
H         -4.339971       -3.031669        3.217997
H         -4.348130       -0.755740        4.180396
H         -3.729906        1.203254        2.770533
H         -2.273219        1.518650       -2.522700
H         -0.979529        0.713121        0.683416
C         -0.981798        2.441325       -0.380142
S         -1.820207        3.697260       -1.134967
N          0.386012        2.464075       -0.321512
```

```
H          0.800191       3.331251      -0.642211
C          2.924316      -0.895592      -0.166203
C          3.266848       0.288910       0.477854
C          2.422305       1.393137       0.451805
C          1.222155       1.323601      -0.255124
C          0.877999       0.152112      -0.935966
C          1.723493      -0.948827      -0.864669
H          3.587021      -1.753210      -0.136111
C          4.538771       0.351505       1.278932
H          2.692140       2.306058       0.973077
H         -0.033794       0.118528      -1.532099
C          1.293052      -2.222236      -1.539791
F          5.018527       1.600115       1.347977
F          4.343032      -0.066441       2.538258
F          5.491805      -0.425927       0.749613
F          0.415300      -2.897091      -0.776025
F          0.683233      -1.980450      -2.705929
F          2.325283      -3.040264      -1.773613
```

Molecule 13

Datum	Value
M06-2X/6-31G(d)-IEFPCM(Benzene) Energy	-1301.564263
M06-2X/def2-TZVPP-IEFPCM(Benzene)//M06-2X/6-31G(d)-IEFPCM(Benzene) Free Energy (Quasiharmonic)	-1301.680432
M06-2x/def2-TZVPP-IEFPCM(Benzene) Energy	-1302.064522
Number of Imaginary Frequencies	0

Frequencies (Top 3 out of 153)

```
1.      15.7141 cm-1
2.      20.4551 cm-1
3.      31.2945 cm-1
```

M06-2X/6-31G(d)-IEFPCM(Benzene) Molecular Geometry in Cartesian Coordinates

```
C          2.471945       0.259078       0.529964
C          5.069732       0.080649       1.620634
C          4.167776       0.962099       2.144387
C          2.861582       1.048533       1.598805
H          6.069141       0.006906       2.040762
H          4.413111       1.605999       2.982094
```

```
O          2.037996       1.945508       2.191588
C          3.419031      -0.658212      -0.035534
C          4.726184      -0.753996       0.522553
C          5.656417      -1.670616      -0.029619
C          5.315556      -2.464972      -1.094728
C          4.021939      -2.365868      -1.657241
C          3.098265      -1.488588      -1.144855
H          6.648637      -1.729019       0.410288
H          6.033872      -3.163963      -1.510570
H          3.758249      -2.987385      -2.507397
H          2.113041      -1.409279      -1.594279
C         -1.598528       0.485454      -0.864667
C          1.078085       0.362102       0.012419
C          0.209677      -0.699583       0.183491
C         -1.142937      -0.653470      -0.260220
H         -2.633335       0.541590      -1.194412
O          0.574921      -1.862564       0.780883
C         -2.008797      -1.879475      -0.035321
C         -0.754625       1.610962      -1.066019
C          0.600662       1.549821      -0.635266
C          1.434686       2.678548      -0.860681
C          0.940591       3.805268      -1.471120
C         -0.408668       3.867549      -1.889072
C         -1.234134       2.789434      -1.691767
H          2.475613       2.634729      -0.554683
H          1.593681       4.656251      -1.637730
H         -0.785201       4.765767      -2.367610
H         -2.272397       2.820211      -2.011643
H          1.176229       1.934856       1.742006
H          1.506859      -1.803296       1.052117
N         -3.366680      -1.757395      -0.512951
H         -1.546697      -2.739249      -0.531700
H         -2.013290      -2.136526       1.030466
C         -3.648416      -2.105636      -1.892579
C         -6.289056       0.295404       1.579814
C         -5.014410       0.221469       2.134046
C         -4.009974      -0.445524       1.449526
C         -4.304426      -1.049789       0.213916
C         -5.626445      -0.896724      -0.257573
N         -6.585792      -0.250644       0.399776
H         -7.094157       0.813917       2.093552
H         -3.007567      -0.478490       1.861249
H         -5.918048      -1.325152      -1.212049
H         -2.746926      -2.527398      -2.338392
H         -3.946026      -1.229863      -2.485203
H         -4.447111      -2.853367      -1.963099
H         -4.803975       0.691976       3.088933
```

Molecule 14

Datum	Value
M06-2X/6-31G(d)-IEFPCM(Benzene) Energy	-2447.697008
M06-2X/def2-TZVPP-IEFPCM(Benzene)//M06-2X/6-31G(d)-IEFPCM(Benzene) Free Energy (Quasiharmonic)	-2447.922458
M06-2x/def2-TZVPP-IEFPCM(Benzene) Energy	-2448.430075
Number of Imaginary Frequencies	0

Frequencies (Top 3 out of 210)

```
1.      19.6435 cm-1
2.      23.4111 cm-1
3.      24.9294 cm-1
```

M06-2X/6-31G(d)-IEFPCM(Benzene) Molecular Geometry in Cartesian Coordinates

```
C        -1.232644       -0.060537        1.207299
C        -1.557595        0.024881        2.560160
C        -0.892695       -0.733334        3.540898
C         0.096085       -1.581830        3.102895
C         0.408756       -1.699772        1.750056
C        -0.211384       -0.949862        0.777717
P        -1.984977        1.008062       -0.099752
H        -2.335809        0.710988        2.878414
H        -1.143151       -0.649746        4.592081
O         0.889498       -2.433929        3.819539
O         1.379593       -2.649005        1.591695
C         0.895913       -1.202846       -3.410583
C        -0.094653       -1.983987       -2.864549
C        -0.410448       -1.918452       -1.508916
C         0.208733       -1.045257       -0.644968
C         1.231590       -0.222196       -1.188148
C         1.559117       -0.319525       -2.539552
H         1.149011       -1.262114       -4.462783
O        -0.886194       -2.925100       -3.461685
O        -1.382034       -2.837262       -1.226089
P         1.981800        1.012797       -0.035211
H         2.338648        0.316884       -2.945741
C         1.908094       -2.831074        2.900106
C        -1.905491       -3.198649       -2.498853
H         2.143593       -3.883107        3.053499
H         2.786758       -2.185098        3.027108
H        -2.135340       -4.263067       -2.508112
H        -2.787178       -2.581137       -2.714610
C        -4.405279        3.986499        2.514628
C        -5.073581        2.905031        1.947520
```

```
C        -4.381675     1.983421     1.162447
C        -3.012591     2.140523     0.929312
C        -2.351891     3.240421     1.493551
C        -3.039342     4.151423     2.288594
H        -4.946270     4.701332     3.127075
H        -6.137938     2.773410     2.118206
H        -4.912407     1.139700     0.730793
H        -1.287917     3.376682     1.309660
H        -2.512061     4.994640     2.724340
C        -5.297888    -1.650755    -1.983026
C        -4.798478    -1.990023    -0.725757
C        -3.791693    -1.224594    -0.141749
C        -3.267115    -0.112888    -0.810268
C        -3.759741     0.205013    -2.081804
C        -4.775730    -0.551883    -2.662524
H        -6.087405    -2.244847    -2.433482
H        -5.197545    -2.849876    -0.195921
H        -3.406747    -1.492725     0.838475
H        -3.346480     1.056053    -2.617926
H        -5.153675    -0.287265    -3.645353
C         4.409615     3.599944    -3.031597
C         5.076021     2.607033    -2.318919
C         4.382115     1.804204    -1.414282
C         3.012963     1.993637    -1.206892
C         2.354328     3.005612    -1.918748
C         3.043758     3.796501    -2.831887
H         4.952007     4.221737    -3.737246
H         6.140325     2.451565    -2.468613
H         4.911266     1.027665    -0.869442
H         1.290331     3.167313    -1.756945
H         2.517955     4.571575    -3.381184
C         5.297977    -1.360739     2.191419
C         4.796144    -1.870803     0.994178
C         3.786876    -1.194338     0.312599
C         3.262546    -0.001469     0.822533
C         3.756955     0.488863     2.037290
C         4.775276    -0.179568     2.714450
H         6.089433    -1.886065     2.717555
H         5.195335    -2.795012     0.586977
H         3.399866    -1.595424    -0.620282
H         3.343639     1.405276     2.451816
H         5.154801     0.218392     3.650604
```

Molecule 15

Datum	Value
M06-2X/6-31G(d)-IEFPCM(Benzene) Energy	-1901.688914

Datum	Value
M06-2X/def2-TZVPP-IEFPCM(Benzene)//M06-2X/6-31G(d)-IEFPCM(Benzene) Free Energy (Quasiharmonic)	-1902.012794
M06-2x/def2-TZVPP-IEFPCM(Benzene) Energy	-1902.288859
Number of Imaginary Frequencies	0

Frequencies (Top 3 out of 120)

```
1.      23.3413 cm-1
2.      25.9318 cm-1
3.      41.0793 cm-1
```

M06-2X/6-31G(d)-IEFPCM(Benzene) Molecular Geometry in Cartesian Coordinates

```
C          1.581926       1.397060       0.441081
C          2.555724       0.652950      -0.224933
C          3.851727       0.562378       0.278542
C          4.171446       1.232210       1.456511
C          3.211213       1.999069       2.114623
C          1.917589       2.083872       1.602865
H          0.566200       1.423533       0.053378
N          2.219390      -0.054659      -1.424982
H          4.586800      -0.028932      -0.256480
H          5.179138       1.164923       1.853711
H          3.471408       2.528808       3.025464
H          1.161197       2.673926       2.111756
H          1.443550       0.364741      -1.938815
S          1.934516      -1.701617      -1.265776
O          3.150678      -2.311102      -0.761675
O          1.322845      -2.110826      -2.518110
C         -1.164064      -1.545091       2.052734
C         -1.531145      -1.389883       0.716509
C         -0.613229      -1.522986      -0.317589
C          0.708689      -1.786484       0.024595
C          1.110276      -1.939501       1.347714
C          0.160074      -1.836648       2.363009
H         -1.915192      -1.451688       2.830896
H         -0.918327      -1.437982      -1.356201
H          2.154028      -2.136316       1.570617
H          0.457041      -1.971894       3.397026
S         -3.198070      -0.909092       0.322422
O         -4.074339      -1.337576       1.398149
N         -3.211778       0.759950       0.424180
O         -3.436864      -1.276962      -1.060397
H         -3.465303       1.053158       1.364015
```

```
C          -0.553929       3.368191       -1.549630
C          -1.015834       2.226172       -2.200243
C          -1.895606       1.347486       -1.569821
C          -2.295754       1.602567       -0.253873
C          -1.825237       2.742282        0.407166
C          -0.973331       3.626479       -0.246255
H           0.120488       4.051869       -2.053671
H          -0.715840       2.024566       -3.224375
H          -2.298403       0.493491       -2.102776
H          -2.139710       2.940125        1.428550
H          -0.625267       4.512985        0.274424
```

Molecule 16

Datum	Value
M06-2X/6-31G(d)-IEFPCM(Benzene) Energy	-902.4516
M06-2X/def2-TZVPP-IEFPCM(Benzene)//M06-2X/6-31G(d)-IEFPCM(Benzene) Free Energy (Quasiharmonic)	-902.493978
M06-2x/def2-TZVPP-IEFPCM(Benzene) Energy	-902.805544
Number of Imaginary Frequencies	0

Frequencies (Top 3 out of 117)

```
  1.      25.7823 cm-1
  2.      30.5588 cm-1
  3.      66.2014 cm-1
```

M06-2X/6-31G(d)-IEFPCM(Benzene) Molecular Geometry in Cartesian Coordinates

```
C           2.891208      -0.096075       -0.313005
O           2.158444      -1.095046       -1.075985
N           0.787993      -0.447885        0.550050
C           1.762499       0.645153        0.442815
C           3.791860      -0.841238        0.667227
C           3.712805       0.725509       -1.287719
H           2.099380       0.918267        1.446725
C           1.160798       1.880996       -0.255547
C           1.004046      -1.395257       -0.454252
O           0.320676      -2.339531       -0.752522
C          -0.131266      -0.514895        1.600587
O          -0.013616       0.240118        2.543781
C          -1.317283      -1.445604        1.451218
```

```
H          -1.025324       -2.419096        1.060567
H          -1.740807       -1.554802        2.452124
C          -4.061219        0.581390       -1.195477
C          -3.469512       -0.613599       -1.595794
C          -2.592290       -1.284794       -0.744922
C          -2.305343       -0.765490        0.518237
C          -2.906111        0.433036        0.916826
C          -3.778481        1.104166        0.065596
H          -4.741472        1.103075       -1.861556
H          -3.687360       -1.026824       -2.575930
H          -2.116396       -2.206625       -1.062354
H          -2.677246        0.839322        1.899812
H          -4.238180        2.033817        0.386895
C           0.383852        1.530269       -1.529564
C           0.260607        2.683985        0.688010
H           2.014122        2.519590       -0.519893
H          -0.523205        0.966411       -1.279160
H           0.070279        2.447314       -2.037068
H           0.965214        0.933044       -2.238045
H          -0.010622        3.634967        0.218679
H          -0.665235        2.141655        0.901519
H           0.754808        2.894015        1.640976
H           4.408741       -0.130378        1.224382
H           3.196260       -1.417292        1.382461
H           4.446670       -1.526821        0.123543
H           4.256898        1.506015       -0.747931
H           4.442347        0.076326       -1.779052
H           3.094131        1.193577       -2.054657
```

Molecule 17

Datum	Value
M06-2X/6-31G(d)-IEFPCM(Benzene) Energy	-1821.423564
M06-2X/def2-TZVPP-IEFPCM(Benzene)//M06-2X/6-31G(d)-IEFPCM(Benzene) Free Energy (Quasiharmonic)	-1821.71716
M06-2x/def2-TZVPP-IEFPCM(Benzene) Energy	-1822.04961
Number of Imaginary Frequencies	0

Frequencies (Top 3 out of 138)

```
1.      11.3526 cm-1
2.      14.2536 cm-1
3.      19.0910 cm-1
```

M06-2X/6-31G(d)-IEFPCM(Benzene) Molecular Geometry in Cartesian Coordinates

```
C          -2.503757       -1.550354       -0.042578
C          -3.490346       -0.603589        0.183607
C          -3.216891        0.760047        0.102384
C          -1.927001        1.147714       -0.227804
C          -0.921567        0.219402       -0.483619
C          -1.204663       -1.145889       -0.377732
H          -2.735275       -2.608059        0.034505
C          -4.869527       -1.046524        0.589357
H          -3.993924        1.494910        0.279029
C          -1.567065        2.605674       -0.296721
H           0.066171        0.575544       -0.765831
N          -0.261049       -2.145294       -0.661487
F          -2.646572        3.386217       -0.415047
F          -0.761352        2.864475       -1.340759
F          -0.908441        2.998609        0.805110
F          -5.017312       -1.019388        1.922243
F          -5.811263       -0.244694        0.074865
F          -5.131323       -2.296639        0.187053
H          -0.609981       -2.991979       -1.092905
C           1.112859       -2.092744       -0.608348
S           2.013060       -3.227514       -1.467192
N           1.643471       -1.131560        0.154713
H           1.025759       -0.510437        0.664672
C           5.319037       -1.245572        1.183030
C           3.838676       -1.631084        1.210127
C           3.057143       -0.785656        0.200471
C           3.191318        0.708554        0.530198
C           4.678743        1.093277        0.461251
C           5.503041        0.251346        1.441612
H           5.735248       -1.498920        0.198749
H           5.873405       -1.832751        1.922032
H           3.421537       -1.463265        2.212488
H           3.701724       -2.688076        0.964248
H           3.467113       -0.986297       -0.796612
N           2.263068        1.519003       -0.266955
H           2.866579        0.839645        1.575857
H           5.050795        0.934875       -0.559796
H           4.800284        2.157617        0.687913
H           5.182810        0.482094        2.466937
H           6.560926        0.524727        1.371634
C           2.480219        1.445762       -1.706482
C           2.221269        2.905334        0.177226
H           3.449800        1.862064       -2.025559
H           2.413502        0.409553       -2.052969
H           1.688855        2.015665       -2.203036
H           3.150372        3.458975       -0.035556
H           1.399217        3.417776       -0.326838
H           2.035117        2.940125        1.255135
```

Molecule 18

Datum	Value
M06-2X/6-31G(d)-IEFPCM(Benzene) Energy	-1873.796713
M06-2X/def2-TZVPP-IEFPCM(Benzene)//M06-2X/6-31G(d)-IEFPCM(Benzene) Free Energy (Quasiharmonic)	-1874.04489
M06-2x/def2-TZVPP-IEFPCM(Benzene) Energy	-1874.445949
Number of Imaginary Frequencies	0

Frequencies (Top 3 out of 168)

```
1.      27.2736 cm-1
2.      31.0990 cm-1
3.      41.1355 cm-1
```

M06-2X/6-31G(d)-IEFPCM(Benzene) Molecular Geometry in Cartesian Coordinates

```
C         -2.894948       -0.333003        0.088533
C         -3.588854        0.697064        0.678896
C         -0.819593        0.984812        0.286853
C         -1.488940       -0.171063       -0.061943
H         -4.663837        0.601568        0.809245
O         -0.788396       -1.201882       -0.685755
C          3.359790        1.314568       -0.696324
C          2.907029        0.224160        0.006496
C          1.520631        0.184091        0.315291
C          0.617646        1.148511       -0.064945
H          4.418078        1.394740       -0.932106
O          1.046686       -0.918908        1.019494
C         -2.943429        1.859374        1.166559
C         -1.541299        2.008013        0.985241
C         -0.905368        3.155259        1.533033
C         -1.632793        4.110410        2.196645
C         -3.033682        3.975517        2.348835
C         -3.671217        2.871249        1.848033
H          0.169272        3.264060        1.433167
H         -1.128630        4.975365        2.615828
H         -3.595528        4.742048        2.872809
H         -4.743199        2.743814        1.971770
C          1.091064        2.248102       -0.856817
C          2.482800        2.334587       -1.143742
C          2.971699        3.426317       -1.909666
C          2.116736        4.377525       -2.400602
```

```
C          0.727681       4.268605      -2.154967
C          0.226974       3.234130      -1.405267
H          4.038673       3.482647      -2.107390
H          2.497825       5.205678      -2.989390
H          0.050045       5.008170      -2.569505
H         -0.841171       3.158035      -1.236312
P          0.290499      -2.046884       0.155166
O          1.257410      -2.428127      -1.051254
H          1.828305      -3.183548      -0.831841
O         -0.175387      -3.146136       1.007710
C         -5.103370      -3.711066      -1.359342
C         -5.482298      -2.415458      -1.700882
C         -4.747142      -1.329908      -1.234418
C         -3.623642      -1.524176      -0.422492
C         -3.249309      -2.828664      -0.082332
C         -3.987806      -3.911958      -0.549264
H         -5.674204      -4.559987      -1.722875
H         -6.347129      -2.247823      -2.335459
H         -5.032286      -0.319188      -1.513473
H         -2.391683      -3.000052       0.560450
H         -3.689769      -4.918645      -0.272873
C          5.577578      -2.975147       1.016669
C          4.694659      -2.482570       1.975907
C          3.817769      -1.449749       1.659412
C          3.815822      -0.890554       0.375140
C          4.704467      -1.393888      -0.582457
C          5.580339      -2.427919      -0.264087
H          6.257772      -3.783527       1.265940
H          4.688452      -2.902563       2.976810
H          3.135032      -1.068658       2.411414
H          4.690345      -0.981430      -1.587487
H          6.259669      -2.810038      -1.019755
```

Molecule 19

Datum	Value
M06-2X/6-31G(d)-IEFPCM(Benzene) Energy	-2052.059482
M06-2X/def2-TZVPP-IEFPCM(Benzene)//M06-2X/6-31G(d)-IEFPCM(Benzene) Free Energy (Quasiharmonic)	-2052.385869
M06-2x/def2-TZVPP-IEFPCM(Benzene) Energy	-2052.887823
Number of Imaginary Frequencies	0

Frequencies (Top 3 out of 201)

```
1.      13.2809 cm-1
2.      19.1588 cm-1
```

```
3.      31.0801 cm-1
```

M06-2X/6-31G(d)-IEFPCM(Benzene) Molecular Geometry in Cartesian Coordinates

```
C        -0.284736       -2.446503       -0.371227
N        -0.584721       -1.293457        0.286205
C        -1.893094       -1.076370        0.907641
C        -3.009271       -0.878674       -0.153148
H        -2.144264       -1.956370        1.515296
C        -1.779469        0.110468        1.845147
H        -2.536576       -0.483657       -1.055121
C        -4.197952        0.006753        0.256233
N        -3.585467       -2.209220       -0.593656
C        -1.342795        1.392279        1.381883
C        -2.089351       -0.036357        3.171421
C        -5.468396       -0.527065       -0.426541
H        -3.977966        1.041307       -0.019420
H        -4.334203       -0.008086        1.343431
C        -4.612624       -2.724723        0.373426
C        -4.203552       -2.035560       -1.952577
C        -0.993180        1.679560        0.042666
C        -1.250070        2.429639        2.355190
C        -1.976995        1.073518        4.048595
H        -2.412530       -0.995347        3.565533
C        -5.864308       -1.832613        0.279576
H        -6.274329        0.206916       -0.348288
C        -5.198546       -0.848160       -1.909966
H        -4.161727       -2.705535        1.367541
H        -4.804768       -3.765649        0.108672
H        -3.382463       -1.872436       -2.655506
H        -4.686488       -2.983260       -2.198611
C        -0.541282        2.932438       -0.320778
H        -1.039791        0.922798       -0.732014
C        -0.797463        3.709682        1.948114
N        -1.572819        2.258292        3.667803
H        -2.227799        0.952598        5.099787
H        -6.245296       -1.632068        1.283864
H        -6.657153       -2.339728       -0.278047
H        -6.141022       -1.194335       -2.347127
C        -4.721563        0.358975       -2.718173
C        -0.444008        3.964826        0.648755
O        -0.206755        3.087605       -1.618367
H        -0.735401        4.481449        2.707973
H        -4.603566        0.094079       -3.772734
H        -5.453328        1.169575       -2.654879
H        -3.761531        0.751178       -2.364393
H        -0.092271        4.950670        0.369710
C         0.377002        4.316638       -2.023240
H         0.641948        4.186568       -3.071229
H        -0.334959        5.142454       -1.924273
```

```
H        1.279989     4.529135    -1.442989
N        1.014667    -2.627463    -0.755881
O       -1.136504    -3.305020    -0.636209
H        0.179417    -0.738371     0.655029
H        1.197763    -3.553052    -1.124900
C        2.105596    -1.742143    -0.584447
C        2.025293    -0.410336    -0.999490
C        3.293394    -2.225388    -0.035476
C        3.123282     0.427149    -0.830904
H        1.124544    -0.032024    -1.474277
C        4.387750    -1.378540     0.093718
H        3.358704    -3.258520     0.292503
C        4.313728    -0.044561    -0.290328
H        5.168830     0.611479    -0.177059
C        5.672622    -1.947488     0.636509
C        2.998852     1.871521    -1.238270
F        5.440834    -2.730655     1.698725
F        6.288808    -2.703419    -0.281571
F        6.522774    -0.986758     1.009023
F        2.334360     2.581633    -0.309691
F        4.192953     2.447987    -1.398984
F        2.320366     1.999184    -2.386142
H       -2.773342    -2.865053    -0.651260
```

Molecule 20

Datum	Value
M06-2X/6-31G(d)-IEFPCM(Benzene) Energy	-463.502032
M06-2X/def2-TZVPP-IEFPCM(Benzene)//M06-2X/6-31G(d)-IEFPCM(Benzene) Free Energy (Quasiharmonic)	-463.424068
M06-2x/def2-TZVPP-IEFPCM(Benzene) Energy	-463.679629
Number of Imaginary Frequencies	0

Frequencies (Top 3 out of 84)

```
1.      43.2558 cm-1
2.      66.2532 cm-1
3.      83.6919 cm-1
```

M06-2X/6-31G(d)-IEFPCM(Benzene) Molecular Geometry in Cartesian Coordinates

```
C          2.503808       1.243212      -0.400936
N          1.368457       0.479364      -0.959092
C          1.264415      -0.733545      -0.121759
C          2.630308      -0.944563       0.584574
C          3.520099       0.180981       0.025540
H          2.877040       1.954897      -1.139210
H          2.153943       1.813525       0.467162
C          0.145758      -0.476705       0.893448
H          0.992286      -1.586459      -0.750617
H          2.518409      -0.848051       1.670001
H          3.037892      -1.937041       0.386489
H          4.071144      -0.171397      -0.852031
H          4.244956       0.547282       0.755425
H          0.448102       0.297198       1.605865
H         -0.150699      -1.373156       1.442824
N         -1.036178       0.058588       0.160314
C         -2.049995       0.784135       0.994594
C         -3.244547       0.868983       0.058686
C         -3.274265      -0.525535      -0.587105
C         -1.799551      -0.964610      -0.656888
H         -1.627613       1.737690       1.313339
H         -2.258698       0.161399       1.868769
H         -4.164759       1.107576       0.592879
H         -3.074147       1.646941      -0.691773
H         -3.835305      -1.219719       0.042251
H         -3.737694      -0.517519      -1.573846
H         -1.385472      -0.961016      -1.665555
H         -1.626781      -1.943879      -0.207466
H         -0.587658       0.724958      -0.502630
H          1.602938       0.206995      -1.911357
```
